# An optimized protocol for stepwise optimization of real-time RT-PCR analysis

**DOI:** 10.1038/s41438-021-00616-w

**Published:** 2021-08-01

**Authors:** Fangzhou Zhao, Nathan A. Maren, Pawel Z. Kosentka, Ying-Yu Liao, Hongyan Lu, James R. Duduit, Debao Huang, Hamid Ashrafi, Tuanjie Zhao, Alejandra I. Huerta, Thomas G. Ranney, Wusheng Liu

**Affiliations:** 1 Soybean Research Institute, Nanjing Agricultural University, 210095 Nanjing, China; 2 Department of Horticultural Science, North Carolina State University, Raleigh, NC 27607 USA; 3 Mountain Crop Improvement Lab, Department of Horticultural Science, Mountain Horticultural Crops Research and Extension Center, North Carolina State University, Mills River, NC 28759 USA; 4 Department of Entomology and Plant Pathology, North Carolina State University, Raleigh, NC 27607 USA; 5 College of Biosystems Engineering and Food Science, Zhejiang University, 310058 Hangzhou, China

**Keywords:** Gene expression analysis, Plant molecular biology, Transcription

## Abstract

Computational tool-assisted primer design for real-time reverse transcription (RT) PCR (qPCR) analysis largely ignores the sequence similarities between sequences of homologous genes in a plant genome. It can lead to false confidence in the quality of the designed primers, which sometimes results in skipping the optimization steps for qPCR. However, the optimization of qPCR parameters plays an essential role in the efficiency, specificity, and sensitivity of each gene’s primers. Here, we proposed an optimized approach to sequentially optimizing primer sequences, annealing temperatures, primer concentrations, and cDNA concentration range for each reference (and target) gene. Our approach started with a sequence-specific primer design that should be based on the single-nucleotide polymorphisms (SNPs) present in all the homologous sequences for each of the reference (and target) genes under study. By combining the efficiency calibrated and standard curve methods with the 2^−ΔΔCt^ method, the standard cDNA concentration curve with a logarithmic scale was obtained for each primer pair for each gene. As a result, an *R*^2^ ≥ 0.9999 and the efficiency (*E*) = 100 ± 5% should be achieved for the best primer pair of each gene, which serve as the prerequisite for using the 2^−ΔΔCt^ method for data analysis. We applied our newly developed approach to identify the best reference genes in different tissues and at various inflorescence developmental stages of *Tripidium ravennae*, an ornamental and biomass grass, and validated their utility under varying abiotic stress conditions. We also applied this approach to test the expression stability of six reference genes in soybean under biotic stress treatment with *Xanthomonas axonopodis* pv. *glycines* (Xag). Thus, these case studies demonstrated the effectiveness of our optimized protocol for qPCR analysis.

## Introduction

Real-time reverse transcription PCR (i.e., real-time RT-PCR or qPCR) has become a powerful and widely used method for quantifying gene expression levels^1^. Relative quantification of gene expression permits the measurement of relative changes in gene expression without knowing the absolute quantity of the reference (or internal control) genes^2^. The accuracy of qPCR analysis highly depends on (i) the specificity of sequence-specific primers, (ii) the optimization of qPCR amplification conditions, and (iii) the accuracy of transcript normalization using stably expressed reference genes. To date, primer design considerations primarily focus on target specificity, primer GC content, self-dimerization, and secondary structure formation^3,4^. Multiple computational tools have been developed for online primer design for qPCR analysis; examples are Primer3Plus^5^, Primique^6^, BatchPrimer3^7^, QuantPrime^8^, PrimerBank^9^, primer-BLAST^10^, MRPrimerW^11^, Oli2go^12^, qPrimerDB^13^, and MRPrimerW2^14^. Primer3Plus, Primique, BatchPrimer3, and QuantPrime were developed based on the Primer3 core algorithm and provide a user-friendly interface with rich filtering constraints, allowing primer ranking. However, these tools are unable to test off-target primer binding. On the contrary, PrimerBank, primer-BLAST, and MRPrimerW were developed to take advantage of the whole-genome sequence data to test off-target binding. Also, qPrimerDB and MRPrimerW2 are the most comprehensive predesigned primer databases that contain genome sequences for 9 and 516 organisms, respectively^13,14^.

A significant drawback of using these tools for primer design is that they largely ignore homologous genes and their sequence similarities for a gene of interest in a plant genome. Highly similar (or identical) homologous genes often exist in a genome of interest due to genome and gene duplication. Single-nucleotide polymorphisms (SNPs) are the only nucleotides that can discern the differences among these homologous gene sequences and can be used to design robust and sequence-specific qPCR primers for each gene. To the best of our knowledge, the SYBR Taq DNA polymerase can differentiate the SNPs in the last one or two nucleotides at the 3’-end of each primer between any two homologous sequences. However, this can only be achieved under optimized qPCR conditions. Considering that qPCR amplicons are commonly 85–125 bp in length, we posit that it is critical to obtain all the homologous sequences of a gene of interest from a plant genome, conduct sequence alignment, and design sequence-specific primers based on the SNPs present among these homologs.

Another major drawback of using these tools for primer design is that they create a false sense of confidence in the quality of the designed primers for users, which can result in the omission of the necessary optimization of qPCR conditions. To date, three methods have been frequently used for qPCR data analysis for relative quantification of gene expression levels: the 2^−ΔΔCt^ method^15^, the efficiency calibrated method^16,17^, and the standard curve method^16,18,19^. Ct stands for the threshold cycle, i.e., the entry cycle of the exponential phase of PCR amplification, where the amount of PCR product begins to across the basal phase of PCR amplification^1^. The 2^−ΔΔCt^ method is fast, efficient, and has been widely used for data analysis. It requires an equal PCR amplification efficiency for both the reference and target genes. The efficiency calibrated method is similar to the 2^−ΔΔCt^ method, except for the primer efficiencies of each gene that are factored into an equation, providing more accurate quantification of gene expression. The standard curve method uses a serial dilution of the input cDNA for the relative quantification of each of the reference and target genes. Optimization of qPCR conditions plays a vital role in the efficiency, specificity, and sensitivity of the designed primers, and is essential for developing a reliable and robust assay. Thus, optimizing qPCR conditions for each reference and target gene should be performed when using the methods mentioned above for data analysis^20–23^.

Testing the stability of a set of reference genes under study is a prerequisite for a reliable and robust analysis of relative gene expression using qPCR. Ideal reference genes should be consistently and steadily expressed in all tissues and are insensitive to environmental conditions. Commonly used reference genes in plants are housekeeping genes such as elongation factor 1 alpha (*EF1α*), actin (*Act*), ubiquitin (*UBI*), glyceraldehyde-3-phosphate dehydrogenase (*GAPDH*), ribosomal protein L18 (*RPL*), tubulin (*TUB*), ribosomal protein S (*RPS*), 18S ribosomal RNA (*18S*), cyclophilin (*CYC*), and TIP41-like family protein (*TIP41*)^24,25^. These genes are often constitutively expressed and required for basic cellular functions. However, their expression levels can change from tissue to tissue or under various experimental conditions and should be experimentally tested and validated for each plant species^1,26,27^. Based on statistical data from the Internal Control Genes (ICG) database (http://icg.big.ac.cn/index.php/Main_Page), *EF1α*, *Act*, *UBI*, and *18S* ranked as the most frequently used reference genes in different plant tissues, at various developmental stages, under salinity stress, and water-deficit stress, respectively^25^.

In this study, we developed an optimized approach for both primer design and stepwise optimization of qPCR conditions for relative gene expression analysis to achieve the goal of *R*^2^ ≥ 0.99 and efficiency = 100 ± 5% in qPCR analysis. Once these two conditions are met, the 2^−ΔΔCt^ method can be reliably and accurately used for data analysis. As case studies, we applied our improved method to identify the most suitable reference genes in *Tripidium ravennae* (L.) H. Scholz (2*n* = 2*x* = 20), an ornamental and bioenergy grass (Poaceae)^28–30^, over a variety of developmental conditions, and validated their utility under varying abiotic (salinity and water-deficit) stress conditions. We also applied our optimized approach to test the expression stability of six reference genes in soybean (*Glycine max* (L.) Merr.; 2*n* = 4 χ = 40) under biotic stress treatment with the phytopathogenic bacteria *Xanthomonas axonopodis* pv. *glycines* (Xag). Our results demonstrated that our optimized protocol is highly effective for qPCR analysis.

## Results

### Selection of candidate reference genes in *T. ravennae*

RNA-Seq data generated through high-throughput Illumina and PacBio sequencing^30^ permitted for the identification of eight gene sequences that showed the most stable digital expression levels in five different tissues of *T. ravennae*: the root, vegetative meristem, inflorescence, flower, and seed tissues (Fig. S1). The sequences corresponded to *EF1α*, Ubi4-like polyubiquitin (*Ubi4*), histone H3.3 isoform X1 (*H3.3*), translationally controlled tumor protein homolog (*TCTPH*), fructose-bisphosphate aldolase (*Aldolase*), BI1-like protein (*BI1*), autophage-related protein 8C (*8C*), and aconitate hydratase (*AH*), respectively (Table S1). *EF1α, Ubi4*, and *H3.3* are housekeeping genes and have been widely used as reference genes in many plant species^31–33^. The other five genes are new candidate reference genes that have not been used for studying gene expression levels in any plant species to date.

The PacBio (i.e., cDNA) sequences of *Ubi4*, *Aldolase*, *BI1*, and *8C* showed relatively stable reads per kilobase of transcript per million mapped reads (RPKM) values ranging from 148.52 to 355.34 in the sampled tissues, except for *Aldolase* in the root (Fig. S1). Comparable digital expression patterns were observed for *EF1α*, *H3.3*, and *TCTPH*, except that their RPKM values were higher (from 240.52 to 629.94) in the vegetative meristem, inflorescence, flower, and seed. Similarly, the RPKM values of *EF1α* and *TCTPH* were higher in the root (1054.89 and 725.26, respectively). *AH* showed the most variation (from 423.27 to 926.13) in its RPKM values in different tissues. These eight genes were selected as the candidate reference genes for further analysis.

### Validation of the accuracy of the PacBio sequences of the eight candidate reference genes in *T. ravennae*

To validate the accuracy of the PacBio sequences of the eight candidate reference genes, we compared the PacBio sequence of each gene with its most similar genomic copy. If SNPs were identified for any genes, we conducted PCR for the amplification of both the PacBio sequence and its most similar genomic copy simultaneously for that gene, followed by Sanger sequencing of the PCR products without cloning. The BlastP search for the homologous sequences of each of these eight genes (i.e., *EF1α, Ubi4, H3.3*, *TCTPH*, *Aldolase*, *BI1*, *8C*, and *AH*) in the reference genome assembly of *T. ravennae*^29^ returned 7, 12, 8, 3, 6, 5, 4, and 3 homologous genomic sequences with high amino acid sequence similarities, respectively (Figs. S2–S9). By comparing PacBio sequences with their respective genomic DNA sequences (Figs. S10–S17), we found the PacBio sequences for *EF1α*, *H3.3*, and *TCTPH* were identical in sequence to their respective genomic copies. However, SNPs were identified between each cDNA sequence and its most similar genomic copy for *Ubi4, Aldolase*, *BI1*, *8C*, and *AH* (Figs. S10–S17).

Four pairs of sequence-specific primers were designed for each of the five PacBio sequences (*Ubi4, Aldolase*, *BI1*, *8C*, and *AH*) to PCR amplify both the PacBio sequence and its most similar genomic copy simultaneously. Each PCR amplicon contained SNPs between each PacBio sequence and its most similar genomic copy (Tables S1 and S2 and Figs. S10–S17). Our preliminary data showed that a 1:10 dilution of cDNA was appropriate for use since the average Ct values varied from 20 to 31 for all the candidate reference genes (data not shown). With the leaf cDNA (1:10 dilution) being used as the PCR templates, Sanger sequencing without cloning revealed that each PCR product was identical in nucleotide sequence to its respective PacBio sequence (Fig. S18). Therefore, these five PacBio sequences were accurate in nucleotide sequence and used as the candidate reference genes for further qPCR analysis.

### Optimization of qPCR conditions

We optimized qPCR conditions by optimizing primer annealing temperature and primer concentration and identifying the optimal template cDNA concentration range and the best primer pair for each gene. To achieve this, four pairs of sequence-specific primers were designed for qPCR for each of the eight candidate reference genes based on cDNA sequence alignment (Table 1, Table S1, and Figs. S10–S17). The primers were 20 or 21 bp in length with one (or two) SNP(s) present in the last nucleotide position(s) at the 3’-end. When possible, we avoided the presence of 5 or more consecutive nucleotides of the same type (e.g., AAAAA or GGGGG) in any primers, and had a C or G in the last nucleotide position at either or both ends of each primer with a 45–55% GC content. PCR amplicons were 85–125 bp in length for most primer pairs and contained SNPs between each gene and its most similar homolog in the genome.

**Fig. 1:**
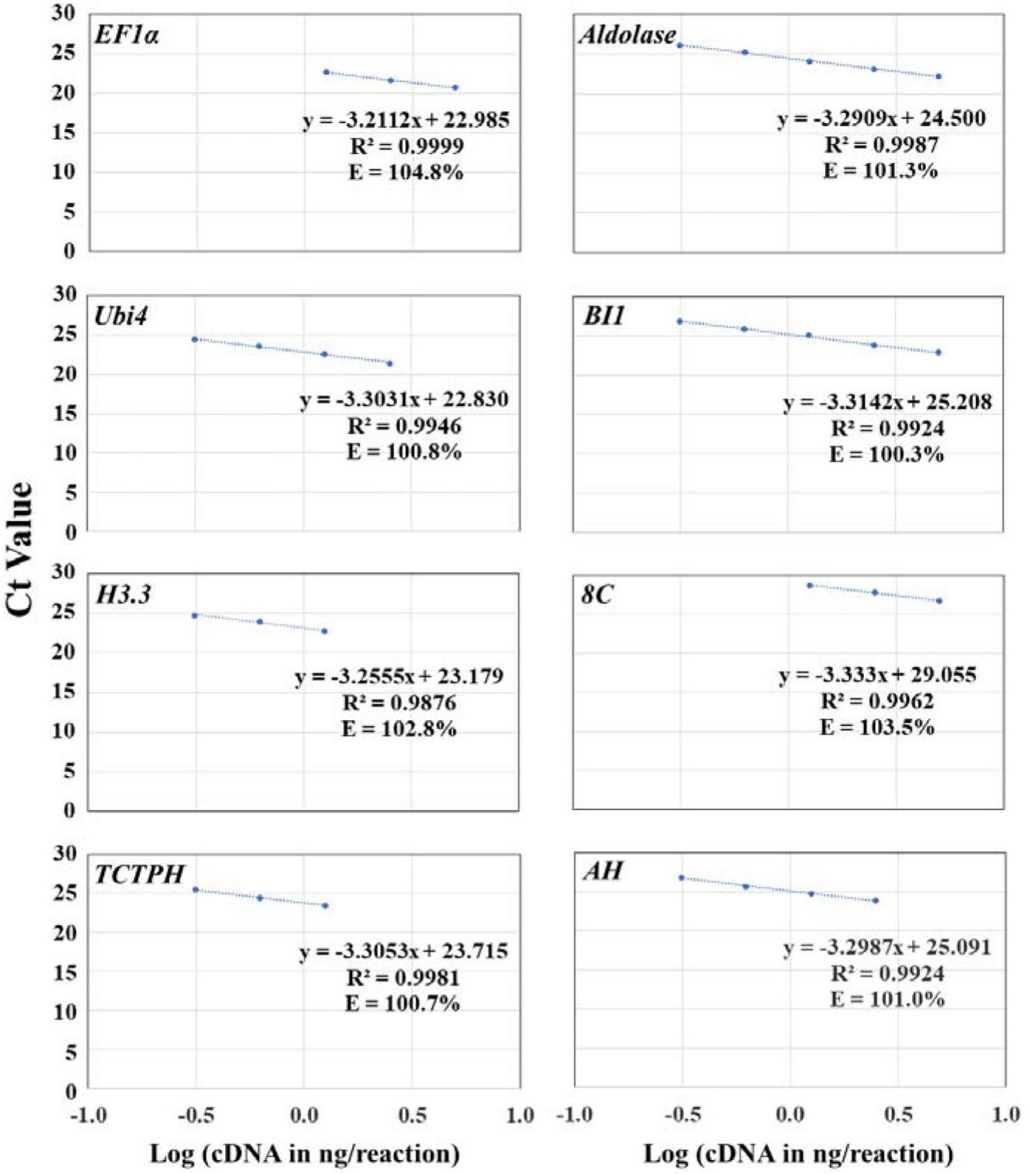
The plot of the averaged Ct values from three technical replicates against the Log (cDNA in ng/reaction) for optimizing qPCR conditions for the best primer pair of each of the eight candidate reference genes in *T. ravennae*. The PCR efficiency (E; %) for each primer pair was calculated as E = (10^-1/A^ − 1) × 100. The cDNA concentration in 1:10, 1:20, 1:40, 1:80, and 1:160 dilutions was 5, 2.5, 1.25, 0.625, 0.3125 ng/µl, respectively, while the Log (cDNA in ng/reaction) for the 1:10, 1:20, 1:40, 1:80, and 1:160 dilutions were 0.69897, 0.39794, 0.09691, −0.20412, and −0.50515, respectively. The data from the lowest (or highest) one (or two) cDNA concentration might have been omitted in order to obtain *R*^2^ ≥ 0.99 and *E* = 100 ± 5% for the data from the remaining four (or three) consecutive cDNA concentrations for the best primer pair for each candidate gene. This served as the prerequisite for using the 2^−ΔΔCt^ method for data analysis

1. *Optimization of primer annealing temperature*: Using the diluted leaf cDNA (1:10 dilution) as the templates and primer concentration of 350 mM per primer per reaction, we conducted gradient qPCR (56, 58, 60.6, and 62.7 °C) to identify the optimal annealing temperature for each primer pair for each gene. All the PCR reactions at 60.6 °C had the lowest Ct values for each primer pair for each gene (Table 1 and Fig. S19). Thus, the annealing temperature at 60.6 °C provided an optimal temperature for each primer pair for each gene.
2. *Optimization of primer concentration*: Using the same leaf cDNA (1:10 dilution) as the templates and 60.6 °C as the optimal annealing temperature, we used different primer concentrations (200, 250, 300, 350, and 400 mM) to determine the optimal primer concentration for each primer pair for each gene. We found that primer concentrations of 350 and 400 mM per primer per reaction had the lowest Ct values (Table 1). The exceptions came from a few primer pairs of *EF1α*, *Ubi4*, *H3.3*, and *Aldolase*, which had the lowest Ct values at the primer concentrations of 250 or 300 mM. The primer concentration with the lowest Ct value for each primer pair was chosen as the optimal primer concentration for that primer pair (Table 1).
3. *Identification of the optimal cDNA concentration range and its associated best primer pair*: Using 60.6 °C as the optimal annealing temperature and the optimal primer concentration for each primer pair as detailed in Table 1, we used serial dilutions of the same leaf cDNA (1:10, 1:20, 1:40, 1:80, and 1:160 dilutions) as the templates to run qPCR and obtain the standard concentration curve with a logarithmic scale for each primer pair. When the efficiency of each primer pair was factored into an equation, as described in the efficiency calibrated method (Fig. 1), we found that different primer pairs had different optimal cDNA concentration ranges for each gene that gave rise to the largest *R*^2^ and optimal efficiencies (100 ± 5%) (Table 1 and Fig. 1). The optimal cDNA concentration range means that the qPCR amplification is at the exponential stage under the conditions of the optimal primer annealing temperature, primer concentration, and cDNA concentration range. Thus, a twofold difference in gene expression results in the difference of one qPCR cycle. As shown in Table 1 and Fig. 1, most *R*^2^ were between 0.98 and 1.0, and most efficiencies were between 100 and 110%. Primer pairs EF1α-qPCR-F1/EF1α-qPCR-R2, Ubi4-F2/Ubi4-R2, H3.3-qPCR-F2/H3.3-qPCR-R2, TCTPH-qPCR-F1/TCTPH-qPCR-R1, Aldolase-F2/Aldolase-R1, BI1-qPCR-F1/BI1-R2, 8C-F2/8C-qPCR-R2, and AH-F1/AH-qPCR-R2 gave rise to the best *R*^2^ (0.9924–0.9999) and efficiencies (100.3–104.8%) (Table 1). The only exception came from the *R*^2^ of the primer pair H3.3-qPCR-F2/H3.3-qPCR-R2 of *H3.3*, which was 0.9876, despite the optimal efficiency at 102.85% for *H3.3*. Taken together, these primer pairs were the best for their respective candidate reference genes. The PCR amplicons from these best primer pairs were 86–121 bp in length, except that the PCR amplicon from the best primer pair BI1-qPCR-F1/ BI1-R2 for *BI1* was 142 bp in length (Table 1).

**Table 1:** The optimized qPCR conditions for the eight candidate reference genes in *T. ravennae*

Gene	Primer pair^a^	Amplicon length (bp)	Optimal Tm (°C)^b^	Optimal primer concentration (mM)	cDNA dilution	*R*^2^	*E* (%)^c^
*EF1α*	EF1α-qPCR-F1/EF1α-qPCR-R1	84	60.6	350	1/10–1/40	0.9996	107.4
	**EF1α-qPCR-F1/ EF1α-qPCR-R2**	**102**	**60.6**	**350**	**1/10–1/40**	**0.9999**	**104.8**
	EF1α-qPCR-F2/EF1α-qPCR-R1	81	60.6	300	1/10–1/160	0.9994	110.3
	EF1α-qPCR-F2/EF1α-qPCR-R2	99	60.6	250	1/10–1/160	0.9992	114.5
*Ubi4*	Ubi4-F1/Ubi4-R1	86	60.6	350	1/10–1/160	0.9898	105.2
	Ubi4-F1/Ubi4-R2	88	60.6	300	1/20–1/160	0.9887	101.6
	Ubi4-F2/Ubi4-R1	84	60.6	350	1/20–1/160	0.9921	98.6
	**Ubi4-F2/Ubi4-R2**	**86**	**60.6**	**350**	**1/20–1/160**	**0.9946**	**100.8**
*H3.3*	H3.3-qPCR-F1/H3.3-qPCR-R1	115	60.6	250	1/20–1/80	0.9929	110.2
	H3.3-qPCR-F1/H3.3-qPCR-R2	121	60.6	350	1/40–1/160	0.9992	96.9
	H3.3-qPCR-F2/H3.3-qPCR-R1	108	60.6	350	1/10–1/40	0.9547	114.2
	**H3.3-qPCR-F2/H3.3-qPCR-R2**	**114**	**60.6**	**250**	**1/40–1/160**	**0.9876**	**102.9**
*TCTPH*	**TCTPH-qPCR-F1/TCTPH-qPCR-R1**	**86**	**60.6**	**350**	**1/40–1/160**	**0.9981**	**100.7**
	TCTPH-qPCR-F1/TCTPH-qPCR-R2	94	60.6	350	1/10–1/160	0.9880	101.7
	TCTPH-qPCR-F2/TCTPH-qPCR-R1	82	60.6	400	1/10–1/80	0.9780	103.4
	TCTPH-qPCR-F2/TCTPH-qPCR-R2	90	60.6	400	1/10–1/80	0.9998	109.1
*Aldolase*	Aldolase-F1/Aldolase-R1	119	60.6	350	1/10–1/160	0.9975	103.5
	Aldolase-F1/Aldolase-R2	120	60.6	300	1/40–1/160	0.9894	108.1
	**Aldolase-F2/Aldolase-R1**	**112**	**60.6**	**400**	**1/10–1/160**	**0.9987**	**101.3**
	Aldolase-F2/Aldolase-R2	113	60.6	350	1/10–1/80	0.9944	102.5
*BI1*	BI1-qPCR-F1/BI1-R1	139	60.6	400	1/10-1//40	0.9116	74.5
	**BI1-qPCR-F1/BI1-R2**	**142**	**60.6**	**400**	**1/10–1/160**	**0.9924**	**100.3**
	BI1-qPCR-F2/ BI1-R1	133	60.6	400	1/10–1/160	0.9900	109.6
	BI1-qPCR-F2/BI1-R2	136	60.6	400	1/10–1/160	0.9696	103.2
*8C*	8C-F1/8C-qPCR-R1	118	60.6	400	1/10–1/80	0.9988	104.6
	8C-F1/8C-qPCR-R2	122	60.6	400	1/20–1/160	0.9998	122.4
	8C-F2/8C-qPCR-R1	117	60.6	400	1/20 –1/80	0.9958	109.9
	**8C-F2/8C-qPCR-R2**	**121**	**60.6**	**400**	**1/10–1/80**	**0.9962**	**103.5**
*AH*	AH-F1/AH-qPCR-R1	80	60.6	400	1/10–1/160	0.9753	105.3
	**AH-F1/AH-qPCR-R2**	**86**	**60.6**	**400**	**1/20–1/160**	**0.9924**	**101.0**
	AH-F2/AH-qPCR-R1	74	60.6	400	1/20–1/160	0.9726	102.8
	AH-F2/AH-qPCR-R1	80	60.6	400	1/20–1/160	0.9993	101.3

^a^The best primer pair for each gene is in bold. ^b^Tm, annealing temperature. ^c^*E* (%), efficiency

As a result, the qPCR conditions were optimized as shown in Table 1 and used for further qPCR analysis in *T. ravennae*.

### Transcript abundance of the eight candidate reference genes across different tissues and inflorescence developmental stages of *T. ravennae*

**Fig. 2:**
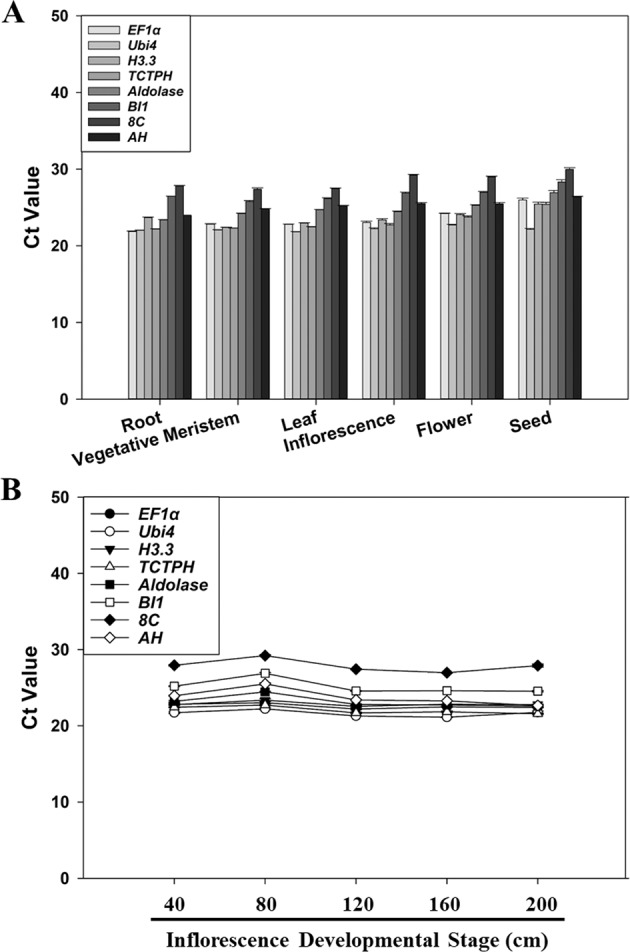
Transcript abundance of the eight candidate reference genes in different tissues (A) and at different inflorescence developmental stages (B) of *T. ravennae* measured by qPCR. RNA was extracted from the root, vegetative meristem, leaf, inflorescence, flower, and immature seed, and from the developmental stages of the inflorescence of 40, 80, 120, 160, and 200 cm in length. Ct values indicate the mean values of transcript abundance from three independent replicates ± standard errors (vertical bars)

We examined the expression stability of each of the eight candidate reference genes across different tissues and inflorescence developmental stages of *T. ravennae*. Under the optimized qPCR conditions (Table 1), the transcript abundance of each candidate reference gene was quantified in the root, vegetative meristem, leaf, inflorescence, flower, and immature seed of *T. ravennae*. The mean Ct values from three biological replicates varied from 22.17 to 28.47 for the eight genes under study (Fig. 2A and Table S2). Gene expression stability, evaluated across the different developmental stages, revealed a ranking of the stability of gene transcript abundance in terms of the same amounts of total RNA per reaction was *Ubi4* > *BI1* > *8C* > *AH* > *H3.3* > *TCTPH* > *Aldolase* > *EF1α* as determined by the coefficient of variation (CV, %) of each gene’s Ct values (Table S2).

Transcript abundance of the eight genes was also measured at different inflorescence developmental stages of *T. ravennae* (i.e., 40, 80, 120, 160, and 200 cm in length). The average Ct values of the eight genes ranged from 21.63 to 27.89 (Fig. 2B and Table S2). The coefficient of variation (CV, %) of each gene’s Ct values across different inflorescence developmental stages demonstrated the stability ranking of transcript abundance in terms of the same amounts of total RNA per reaction was *H3.3* > *EF1α* > *Ubi4* > *Aldolase* > *AH* > *TCTPH* > *8C* > *BI1* (Table S2).

### Transcript abundance of the eight candidate reference genes in *T. ravennae* under salinity stress treatment

**Fig. 3:**
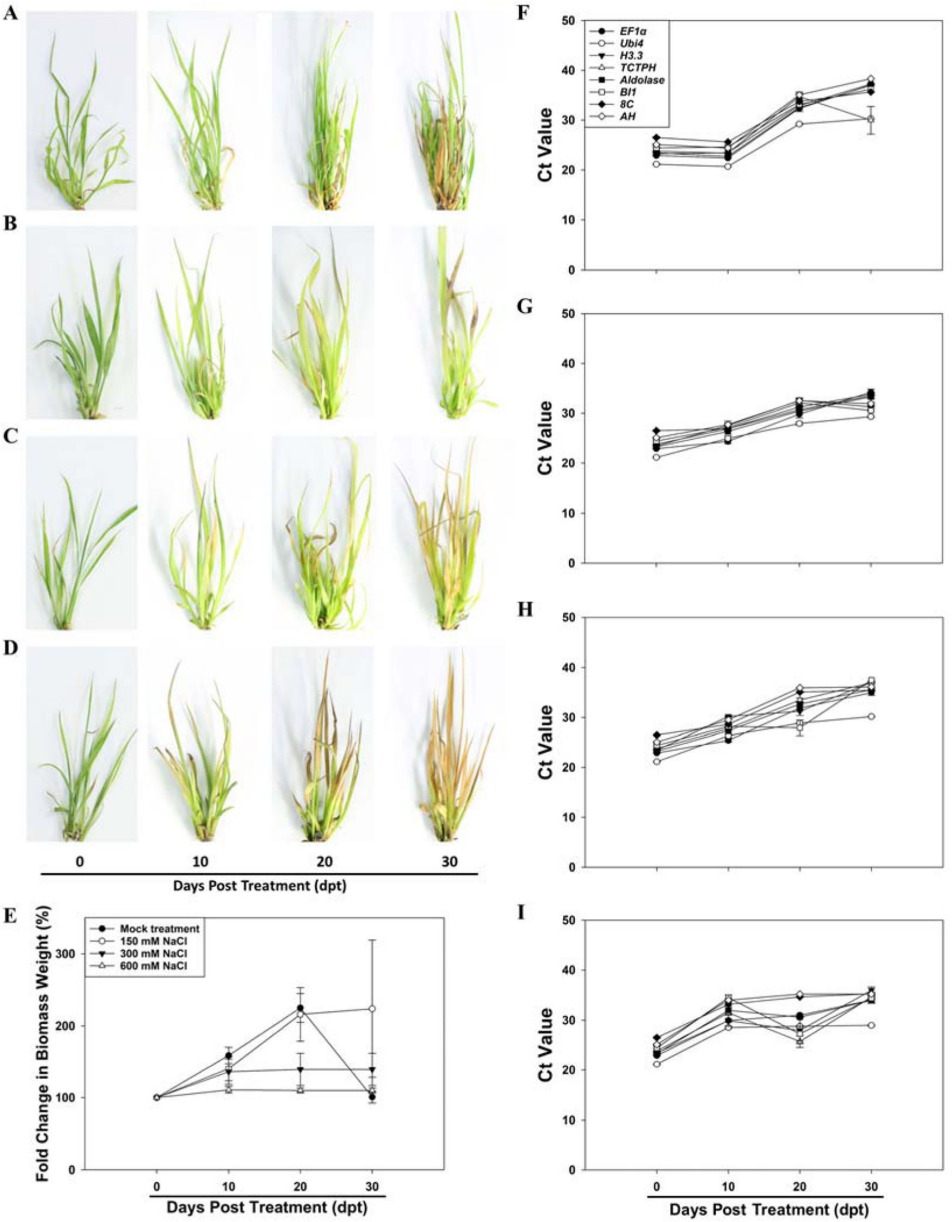
Representative images of the phenotype, fold change in fresh biomass weight, and transcript abundance of the eight candidate reference genes measured by qPCR in the leaves of *T. ravennae* seedlings under salinity stress. Images were taken with a Canon EOS Rebel T6 digital camera and a 35 mm macro lens at 0, 10, 20, and 30 dpt with 0 (**A**), 150 (**B**), 300 (**C**) and 600 (**D**) mM NaCl. Fold change in fresh biomass weight (**E**) was calculated by dividing the fresh biomass weight at each time point by the fresh biomass weight at 0 dpt. RNA was extracted from the leaves at 0, 10, 20, and 30 days post treatment (dpt) with 0 (**F**), 150 (**G**), 300 (**H**), and 600 (**I**) mM NaCl. Ct values indicate the mean values of transcript abundance from three independent replicates ± standard errors (vertical bars)

We also examined the expression stability of each of the eight candidate reference genes in *T. ravennae* under salinity stress treatment. Transcript abundance of each gene was measured in the leaves of the *T. ravennae* clones treated with 0, 150, 300, and 600 mM NaCl for 0, 10, 20, and 30 days (Fig. 3A–E). Under the mock treatment (negative control; 0 mM NaCl), the clones’ phenotypic analysis found that all the clones were healthy at each time point except for 30 days post treatment (dpt) where ~30% of the leaves exhibited a chlorosis phenotype (Fig. 3A). Growth performance analysis revealed that fresh biomass steadily and significantly increased from 0 to 20 dpt by 2.3-folds (*P* < 0.05, Fig. 3E). Growth from 20 to 30 dpt resulted in a significant decrease in fresh biomass weight (*P* < 0.05, Fig. 3E), a possible outcome of growing the plants in the test tube. The outcome led to the exclusion of the data collected at 30 dpt from further data analysis.

Phenotypic analysis of the clones treated with 150, 300, and 600 mM NaCl revealed that the severity of the salinity stress phenotype was correlated with the NaCl concentration and treatment duration (Fig. 3B–D). The clones treated with 150 and 600 mM NaCl showed the least and most growth stress, respectively (Fig. 3B, D). Analysis of plant growth revealed that clones’ fresh weight at 10 and 20 dpt with 150 mM NaCl and 10 dpt with 300 mM NaCl were similar to that of the mock treatment (*P* > 0.05, Fig. 3B). However, the fresh biomass weight at 10 and 20 dpt with 600 mM NaCl decreased significantly compared to that of the mock treatment (*P* < 0.05, Fig. 3B). Together, we concluded that the clones were tolerant to the treatments with 150 mM NaCl for 20 days and 300 mM NaCl for 10 days, and were highly stressed by 600 mM NaCl for 10 and 20 days.

Under the mock treatment, transcript abundance of each gene at 20 dpt significantly decreased when compared to that at 0 dpt (*P* < 0.05) even though there was an insignificant difference between 10 and 0 dpt (Fig. 3F). This noncontinuous change in transcript levels suggests that the plants at 20 dpt were suffering from an abiotic factor not related to the treatment and possibly related to the growth condition of the plants in the test tubes, and thus, data collected at 20 dpt were excluded from further analysis. The transcript abundance at 10 dpt is inversely correlated to NaCl concentrations (Fig. 3G–I). There was an insignificant difference in each gene’s transcript abundance at 10 and 0 dpt with 150 mM NaCl (*P* > 0.05, Fig. 3G). However, there was a significant difference in each gene’s transcript abundance at both 0 and 10 dpt with 300 or 600 mM NaCl (*P* < 0.05, Fig. 3H, I).

Time-course analysis of the average Ct values for each gene under the mock treatment at 0 and 10 dpt showed that the coefficient of variation (CV, %) of the Ct values of the eight genes at both time points was ranked as *BI1* > *Aldolase* > *TCTPH* > *Ubi4* > *EF1α* > *AH* > *H3.3* > *8C*. However, the salinity stress treatments at 10 dpt changed the ranking of the coefficient of variation (CV, %) of the Ct values (Table S3). We found that the *8C* gene had the lowest coefficient of variation of the Ct values under the treatments with 150, 300, and 600 mM NaCl, and *EF1α* had the second-lowest coefficient of variation of the Ct values under the treatments with 150 and 300 mM NaCl, and the third-lowest coefficient of variation of the Ct values under the treatments with 600 mM NaCl (Table S3).

### Transcript abundance of the eight candidate reference genes in *T. ravennae* under water-deficit treatment

**Fig. 4:**
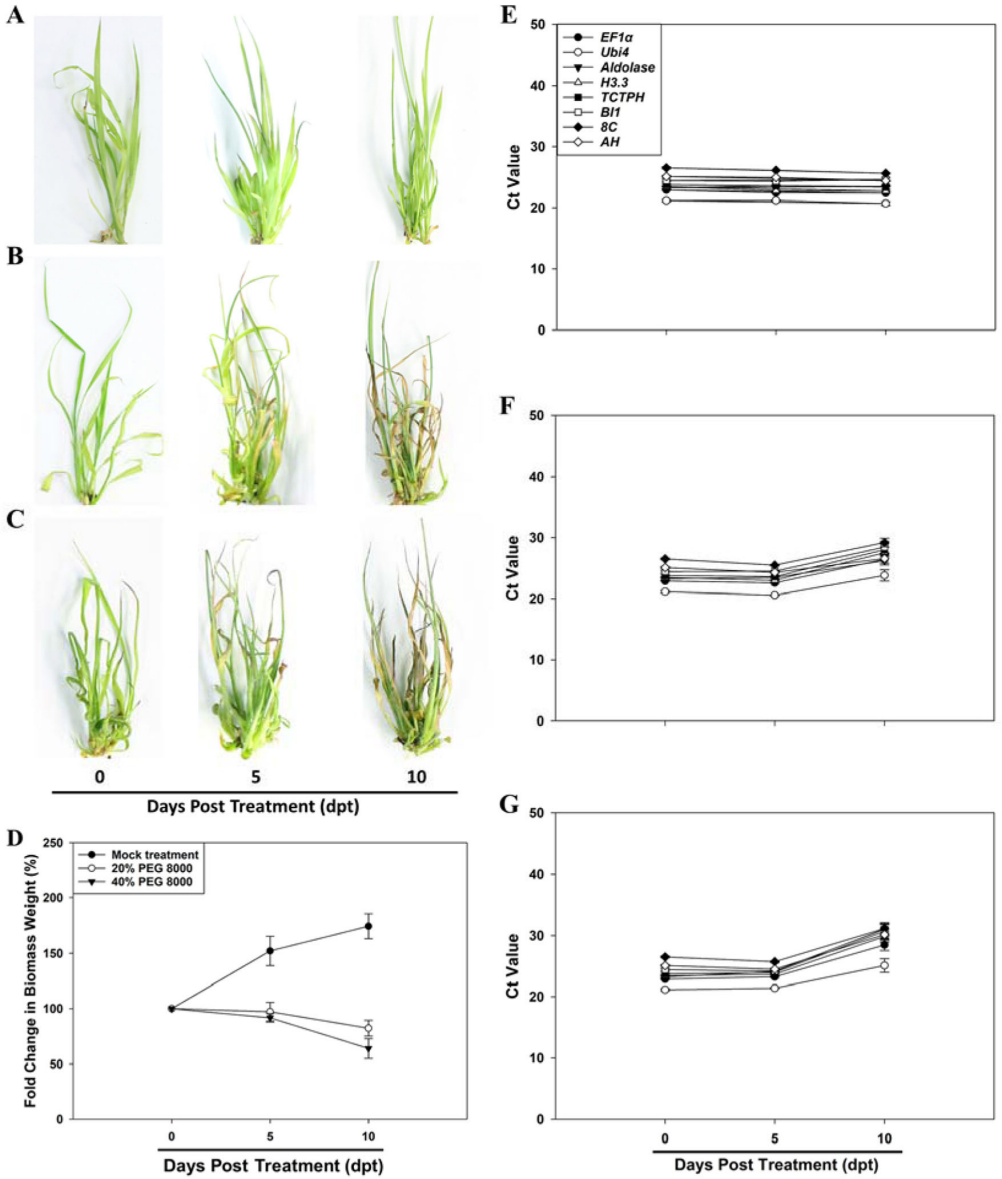
Representative images of the phenotype, fold change in fresh biomass weight, and transcript abundance of the eight candidate reference genes measured by qPCR in the leaves of *T. ravennae* seedlings under water-deficit stress. Images were taken with a Canon EOS Rebel T6 digital camera and a 35 mm macro lens at 0, 5, and 10 dpt with 0 (**A**), 20% (**B**) and 40% (**C**) PEG 8000. Fold change in fresh biomass weight (**D**) was calculated by dividing the fresh biomass weight at each time point by the fresh biomass weight at 0 dpt. RNA was extracted from the leaves at 0, 5 and 10 h post treatment (dpt) with 0 (**E**), 20% (**F**), and 40% (**G**) PEG 8000. Ct values indicate the mean values of transcript abundance from three independent replicates ± standard errors (vertical bars)

Next, we studied the expression stability of each of the eight candidate reference genes in *T. ravennae* under water-deficit treatment. Transcript abundance of the eight candidate reference genes was quantified in the leaves of the *T. ravennae* clones treated with 0%, 20%, and 40% PEG 8000 for 0, 5, and 10 days (Fig. 4A–D). The phenotypic analysis found that the clones under the mock treatment (0% PEG 8000) were healthy at each time point (Fig. 4A), but the clones treated with 20% and 40% PEG 8000 exhibited enhanced stress phenotype severity as PEG 8000 concentration increased (Fig. 4B, C). The severity of the stress phenotype at 5 dpt was apparently milder than that at 10 dpt with 20% and 40% PEG 8000. Growth performance analysis revealed that the clones’ fresh weight under the mock treatment steadily and significantly increased from 0 to 10 dpt by 1.59 times (*P* < 0.05, Fig. 4D). However, the fresh biomass weight under the 20% and 40% PEG 8000 treatments significantly decreased at 5 and 10 dpt when compared to that at 0 dpt (*P* < 0.05, Fig. 4D).

Under the mock treatment, each gene’s transcript abundance at 5 and 10 dpt was insignificantly different from that at 0 dpt (*P* > 0.05, Fig. 4E). The transcript abundance of each gene at 5 dpt with 20% and 40% PEG 8000 was also insignificantly different from that at 0 dpt (*P* > 0.05, Fig. 4F, G). However, the transcript abundance of each gene at 10 dpt with 20% and 40% PEG 8000 significantly decreased compared to that of 0 dpt (*P* < 0.05, Fig. 4F, G).

Time-course analysis (i.e., 0, 5, and 10 dpt) of the average Ct values under the mock treatment revealed that the coefficient of variation (CV, %) of the Ct values of the eight genes at the three time points was ranked as *BI1* > *Aldolase* > *TCTPH* > *Ubi4* > *EF1α* > *AH* > *H3.3* > *8C*. However, the water-deficit stress treatments at 5 and 10 dpt changed the ranking of the coefficient of variation (CV, %) of the Ct values of each gene (Table S4). We found that *AH* had the lowest coefficient of variation of the Ct values at the three time points of treatments with 20% PEG 8000, followed by *Aldolase*, *8C*, *Ubi4*, *BI1*, *EF1α*, *TCTPH*, and *H3.3*. *Ubi4* had the lowest coefficient of variation of the Ct values at the three time points of treatments with 40% PEG 8000, followed by *8C*, *AH*, *EF1α*, *H3.3*, *BI1*, *TCTPH*, and *Aldolase* (Table S4).

### Analysis of the expression stability of the eight candidate reference genes in *T. ravennae*

Since RNA components and their concentrations vary from sample to sample^34^, it is critical to analyze the expression stability of the candidate reference genes under different experimental conditions using statistical algorithms such as RefFinder^35^, which were designed to identify a set of reference genes with similar expression patterns in different samples. The comprehensive ranking by RefFinder showed that the stability rankings of the genes were *BI1* > *TCTPH* > *AH* > *Aldolase* > *8C* > *H3.3* > *EF1α* > *Ubi4* in different tissues, and *EF1α* > *Aldolase* > *H3.3* > *TCTPH* > *Ubi4* > *8C* > *BI1* > *AH* at different inflorescence developmental stages (Table 2). The stability rankings under salinity and water-deficit stresses were *TCTPH* > *Aldolase* > *EF1α* > *AH* > *8C* > *Ubi4* > *BI1* > *H3.3*, and *EF1α* > *8C* > *BI1* > *H3.3* > *TCTPH* > *AH* > *Ubi4* > *Aldolase*, respectively (Table 2). When all the samples were analyzed in combination, the stability ranking was *Aldolase* > *EF1α* > *Ubi4* > *8C* > *H3.3* > *AH* > *BI1* > *TCTPH* (Table 2).

**Table 2:** Ranking of expression stability of the eight candidate reference genes in *T. ravennae* with Ct values being calculated using RefFinder

Ranking order	Tissues		Inflorescence developmental stages		Salinity treatment		Water-deficit treatment		Total	
	Gene	Stability value	Gene	Stability value	Gene	Stability value	Gene	Stability value	Gene	Stability value
1	*BI1*	0.170	*EF1α*	2.210	*TCTPH*	1.570	*EF1α*	2.000	*Aldolase*	1.570
2	*TCTPH*	0.250	*Aldolase*	2.240	*Aldolase*	1.680	*8C*	3.080	*EF1α*	1.860
3	*AH*	0.300	*H3.3*	2.340	*EF1α*	2.830	*BI1*	3.220	*BI1*	3.220
4	*Aldolase*	0.320	*TCTPH*	2.630	*AH*	3.710	*H3.3*	3.440	*AH*	4.230
5	*8C*	0.330	*Ubi4*	3.460	*8C*	4.560	*TCTPH*	3.560	*Ubi4*	4.330
6	*H3.3*	0.340	*8C*	5.730	*Ubi4*	5.440	*AH*	4.740	*8C*	4.760
7	*EF1α*	0.450	*BI1*	7.000	*BI1*	6.190	*Ubi4*	4.760	*H3.3*	6.240
8	*Ubi4*	0.650	*AH*	8.000	*H3.3*	8.000	*Aldolase*	6.190	*TCTPH*	6.650

*H3.3* and *AH* did not rank high among the most stably expressed genes under any conditions and should not be used as reference genes for qPCR analysis in *T. ravennae* under these conditions tested in this work.

### Optimal numbers of reference genes in *T. ravennae*

**Fig. 5:**
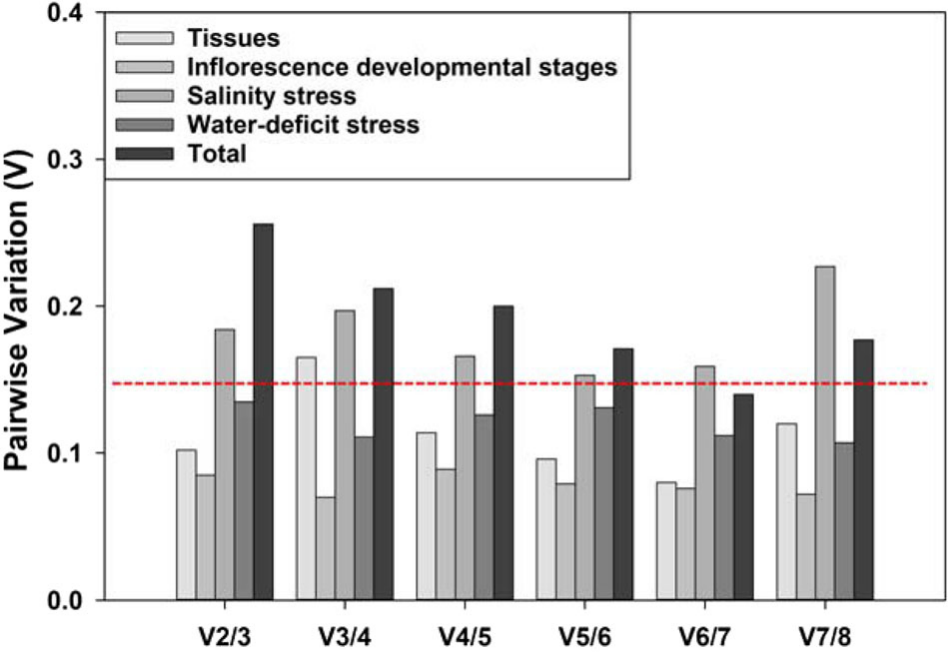
The optimal number of reference genes for qPCR normalization in *T. ravennae* detected by geNorm. The pairwise variation (V_n/n+1_) of the eight candidate reference genes under various conditions were calculated by the geNorm software, and the optimal number of reference genes was determined by the lowest number of genes with V_n_/V_n+1_ smaller than the threshold of 0.15

To identify how many genes should be used as the reference genes for qPCR analysis, the reference genes that showed similar expression patterns in different samples should be identified. The geNorm method was used to calculate the expression pairwise variation (V) of each candidate gene against all the other candidate genes under different experimental conditions. As shown in Fig. 5, the V of V_2/3_ was smaller than the threshold of 0.15 for all the eight genes in all the tissues, at various inflorescence developmental stages, and under water-deficit stress. Thus, the use of the two most stably expressed reference genes ensures accurate normalization of qPCR data; the addition of one more reference gene did not make a significant difference. Under salinity stress or when all samples analyzed in combination, however, all the V-values were larger than the 0.15 threshold except V_6/7_ (0.14) for all samples in combination (Fig. 5). Since the use of six or more reference genes in qPCR is unpractical, we propose to use the two most stable genes as reference genes for salinity stress treatment in this study.

### Relative expression levels of *H3.3* and *AH* in *T. ravennae*

**Fig. 6:**
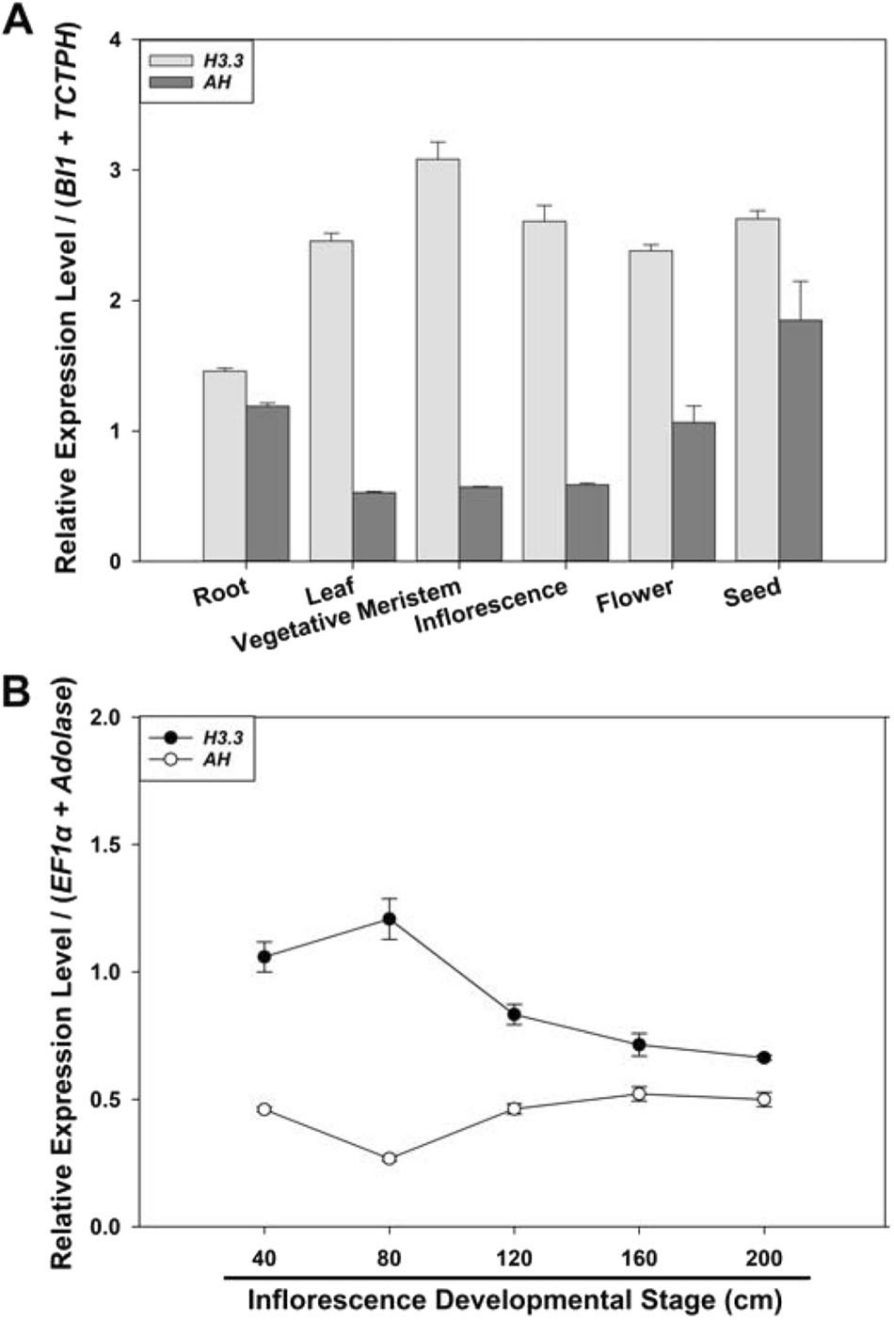
Relative expression levels of *H3.3* and *AH* in different tissues (A) and at various inflorescence developmental stages (B) of *T. ravennae* measured by qPCR. The two best reference genes *BI1* and *TCTPH* in different tissues (**A**) and *EF1α* and *Aldolase* at various inflorescence developmental stages (**B**) were used as the reference genes. Data analysis was conducted using the 2^−ΔΔCt^ method. Relative expression levels indicate the mean values of three independent replicates ± standard errors (vertical bars)

**Fig. 7:**
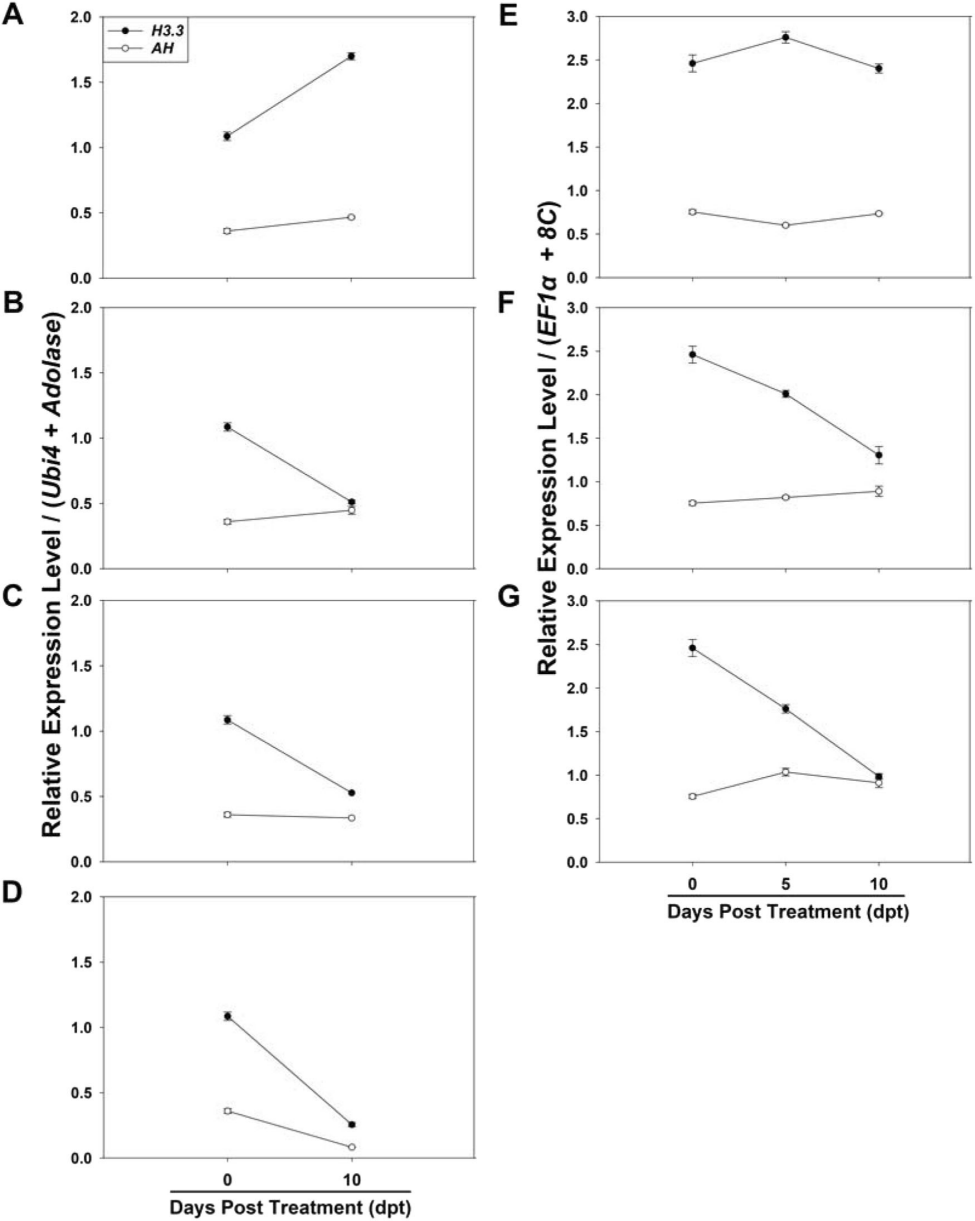
Relative expression levels of *H3.3* and *AH* in *T. ravennae* under salinity stress (A–D) and water-deficit stress (E–G) measured by qPCR. The two best reference genes *Ubi4* and *Aldolase* under salinity stress (**A**–**D**) and *EF1α* and *8C* under water-deficit stress (**E**–**G**) were used as the reference genes. Data analysis was conducted using the 2^−ΔΔCt^ method. Relative expression levels indicate the mean values of three independent replicates ± standard errors (vertical bars)

Since *H3.3* and *AH* did not rank among the best reference genes in this study, we proposed they should not be used as reference genes for qPCR analysis in *T. ravennae*. To better understand their poor expression stability, we chose to quantify the relative expression levels of both genes in *T. ravennae*. *BI1* and *TCTPH*, *EF1α* and *Aldolase*, *Ubi4* and *Aldolase*, and *EF1α* and *8C* were used as the reference genes in different tissues, at various inflorescence developmental stages, under salinity stress, and under water-deficit stress, respectively (Table 2). Thus, the geometric mean (which was calculated by multiplying the Ct values of the two reference genes and then taking a square root) of the two reference genes was used as the Ct of the reference genes for each experimental condition. Using the optimal annealing temperature (60.6 °C) and primer concentration (250, 300, 350, or 400 mM) for the best primer pair of each gene and 1:40 cDNA dilution (Log (cDNA in ng/reaction) = 0.09691) for all the samples (Table 1 and Fig. 1), the relative expression levels of both genes were quantified in different tissues, at different inflorescence stages, and under the salinity and water-deficit stress treatments (Figs. 6 and 7). These data showed that both genes had variable relative expression levels under different conditions in this study.

### Analysis of the expression stability of six candidate reference genes in soybean under biotic stress treatment with Xag

We confirmed our improved approach for expression stability of six candidate reference genes in soybean under biotic stress treatment with Xag, the causal agent of bacterial leaf pustule (BLP) disease and one of the most destructive bacterial diseases of susceptible soybean varieties. Soybean cv. Williams 82 (W82) was used as a BLP-resistant variety^36,37^ and cv. Jack as a BLP-susceptible variety. The candidate reference genes *Actin* (*Glyma.08G1822000*), *cons4* (*Glyma.12G020500*), *cons6* (*Glyma.12G051100*)*, Tubulin* (*Glyma.08G014200*), *FtsZ homolog 2-1* (*FtsZ2-1*; *Glyma.19G194800*), and *Tubulin beta chain* (*TUBB; Glyma.04G023900*) were selected from Kim et al.^38^ and Libault et al.^39^ that studied their expression stability at 12 hpt with Xag treatment. The cDNA and protein sequences of all the homologous sequences of each candidate reference gene were downloaded from the soybean whole-genome sequence database in Phytozome. Following protein sequence alignment (Figs. S20–S25) and then cDNA sequence alignment (Figs. S26–S31), the SNPs were identified between each candidate reference gene and its most similar homolog in the soybean genome and used for primer design for subsequent qPCR analysis. Using the diluted W82 leaf cDNA (1:10 dilution) as the templates, optimization of qPCR conditions was conducted as described above and summarized in Table S5. We found the optimal annealing temperatures were 58 or 60.6 °C, and the optimal primer concentrations were 250, 300, 350, or 400 mM for the four primer pairs of each gene (Table S5). Using the optimal annealing temperatures and the optimal primer concentrations for each primer pair and the serially diluted W82 leaf cDNA (1:10, 1:20, 1:40, 1:80, and 1:160 dilutions) as the templates, we obtained the standard concentration curve with a logarithmic scale for each primer pair for each gene (Fig. S32). We found the best primer pairs for the six candidate genes were Gmcons4-qPCR-F2/Gmcons4-qPCR-R2, GmActin-qPCR-F2/GmActin-qPCR-R2, Gmcons6-qPCR-F1/Gmcons6-qPCR-R1, GmTubulin-qPCR-F1/GmTubulin-qPCR-R1, Gm023900-qPCR-F1/Gm023900-qPCR-R1, and Gm194800-qPCR-F1/Gm194800-qPCR-R2, which gave rise to the best *R*^2^ (0.9852–0.9975) and efficiencies (100.46–104.23%) (Tables S1 and S5). The PCR amplicons from these best primer pairs were 88–124 bp in length.

**Fig. 8:**
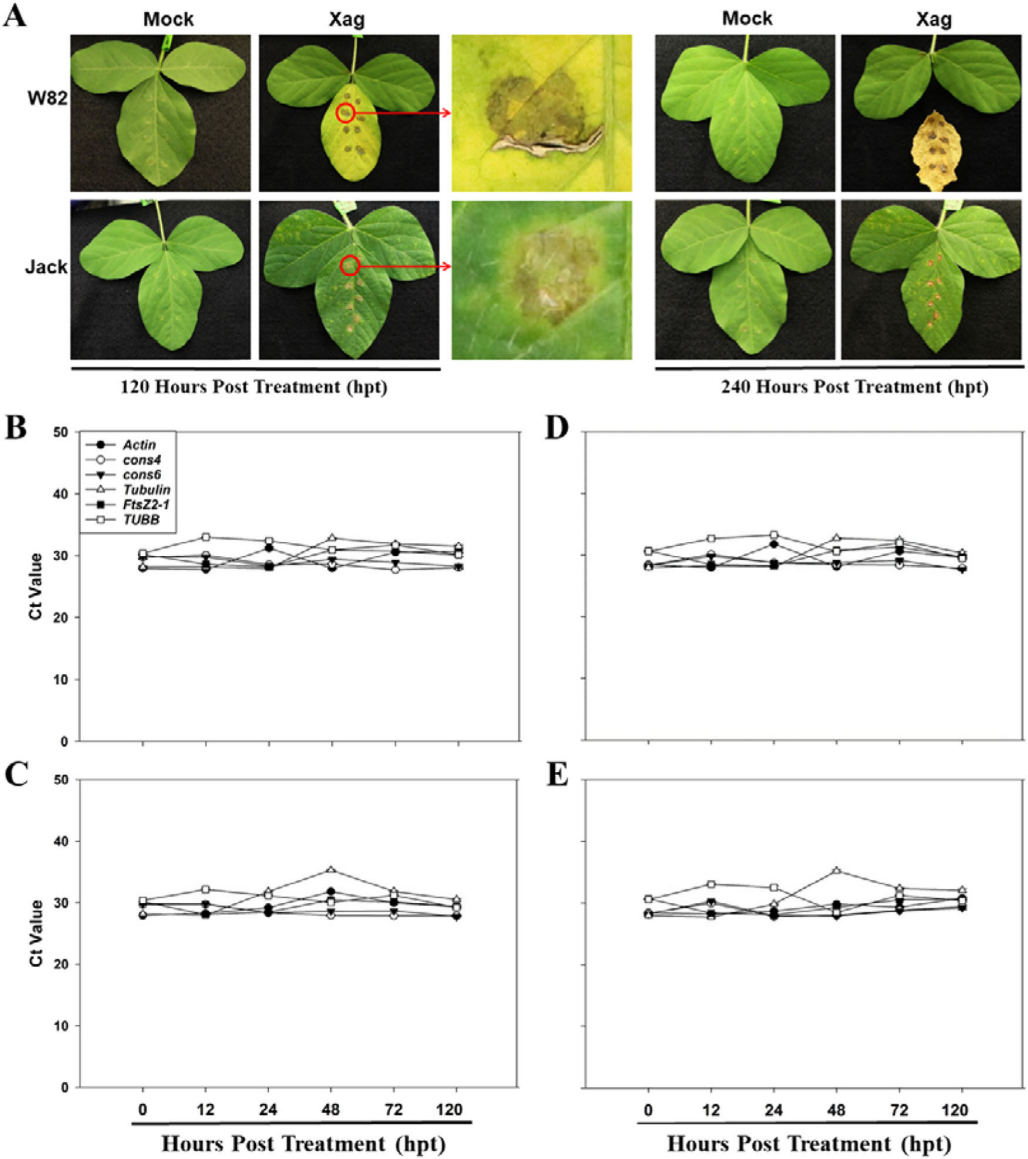
Disease symptoms (A) and transcript abundance (B) of the six candidate reference genes in the leaves of soybean cvs. W82 (Xag-resistant) and Jack (Xag-susceptible) under Xag strain EB08 treatment. Disease symptoms were assessed at 120 and 240 h post treatment (hpt) with Xag strain EB08 (**A**) which was used as the virulent pathogen with 10 mM MgCl_2_ being used as the mock treatment. RNA was extracted from soybean leaves at 0, 12, 24, 48, 72, and 120 hpt, and transcript abundance was quantified by qPCR in W82 under the mock (**B**) and Xag (**C**) treatments, and in Jack under the mock (**D**) and Xag (**E**) treatments. Ct values indicate the mean values of transcript abundance from three independent replicates ± standard errors (vertical bars)

To test the expression stability of these six candidate reference genes under Xag treatment, the leaves of soybean cvs. W82 and Jack were inoculated with pathogenic Xag strain EB08^40^ by leaf infiltration and subsequent spray inoculation. Symptoms were assessed daily and there was no symptoms observed on the leaves treated with the mock (10 mM MgCl_2_ buffer) treatment (Fig. 8A). In Jack treated with Xag, chlorosis was observed on the leaf surface at 72 hpt, and raised centers on the lower leaf surfaces were observed at 120 hpt. Pustules then started to appear with small yellow haloes surrounding each lesion, and lesions coalesced into larger necrotic areas by 240 hpt (Fig. 8A). However, in W82 inoculated with Xag, minimal chlorosis was observed on the leaf surface at 72 hpt, and large necrotic lesions developed after that. The BLP symptoms on the leaves of W82 were restricted to the leaf infiltration sites at 120 hpt. At 240 hpt, the inoculated leaves had become chlorotic and fell (Fig. 8A).

Transcript abundance of the six candidate reference genes was evaluated in the leaves of soybean cvs. W82 and Jack inoculated with Xag at 0, 12, 24, 48, 72, and 120 hpt. Under the mock treatment, the average Ct values for the six genes in the three biological replicates of W82 across the six time points varied from 28.80 to 31.44 (Fig. 8B and Table S6). The coefficient of variation (CV, %) of the Ct values of the six genes across the six time points showed similar stability of gene transcript abundance in terms of the same amounts of total RNA per reaction ranking the expression stability as *cons6* > *cons4* > *TUBB* > *FtsZ2-1* > *Actin* > *Tubulin*. Similarly, the stability of gene transcript abundance in Jack under the mock treatment was ranked as *cons6* > *cons4* > *FtsZ2-1* > *TUBB* > *Actin* > *Tubulin* (Fig. 8D and Table S6). Time-course analysis of transcript abundance of the six genes in W82 and Jack under Xag treatment revealed that the treatment slightly changed the ranking of the coefficient of variation (CV, %) of the Ct values of the six genes (Fig. 8C, E and Table S6). The stability of gene transcript abundance in W82 and Jack under Xag treatment in terms of the same amounts of total RNA per reaction was ranked as *cons6* > *cons4* > *FtsZ2-1* > *TUBB* > *Actin* > *Tubulin*, and *cons4* > *cons6* > *Actin* > *FtsZ2-1* > *TUBB* > *Tubulin*, respectively (Table S6).

To confirm that biotic stress did not affect the expression patterns of the six gens in question, we monitored the bacterial populations on both Jack and W82 treated with Xag for the duration of the experiment. As expected, the bacterial population increased significantly from none detected at 0 hpt to 5.6 Log (CFU/g) at 12 hpt (Fig. S33). The bacterial populations remained steady through the duration of the experiment averaging a mean bacterial population of 5.5 CFU/g of tissue in both soybean cultivars (Fig. S33). No bacteria was detected in any soybean plants treated with the mock (10 mM MgCl_2_ buffer).

RefFinder ranked the stability of the six genes in both soybean cultivars treated with Xag as *cons6* > *cons4* > *FtsZ2-1* > *TUBB* > *Actin* > *Tubulin* for W82, and *cons6* > *cons4* > *FtsZ2-1* > *Actin* > *TUBB* > *Tubulin* for Jack (Table S7). When all the samples were analyzed together, the stability rankings of the six genes were *cons6* > *cons4* > *FtsZ2-1* > *TUBB* > *Actin* > *Tubulin* (Table S8). As a result, *cons6* and *cons4* were ranked as the most stably expressed genes, while *Tubulin* was ranked as the least stably expressed genes under all the experimental conditions. Since all the V-values were larger than the threshold of 0.15 (Fig. S34), we recommended *cons6* and *cons4* be used as the reference genes in soybean under Xag treatment.

## Discussion

qPCR assays are easy to implement in a laboratory but more complex than perceived by many scientists^41^. Although the Minimum Information for Publication of Quantitative Real-time PCR Experiments (MIQE) guidelines and other methodology articles have been published to help scientists improve the quality of qPCR gene expression results, the accuracy and repeatability of qPCR results in many publications remain unvalidated^26,42,43^. This challenge lies in data reproducibility when publications do not provide detailed information about sample quality, RNA quality and integrity, primer specificity, optimized qPCR conditions and cDNA concentration, and amplification efficiencies. Poorly performed qPCR reactions could lead to misinterpreted results, which are difficult or even impossible to reproduce^41^. Hence, we proposed a systematic and comprehensively optimized protocol of qPCR analysis and used it to identify the optimal reference genes in *T. ravennae* and soybean under various experimental conditions as case studies.

Our optimized protocol for qPCR analysis starts with a stepwise SNP-based primer design. Considering that 65% of annotated genes in plant genomes are multi-copied due to repeated whole-genome duplication and gene duplication^44,45^, it is necessary to obtain all the homologous sequences of each reference and target gene of interest in a plant genome. The homologous sequences of each gene can be downloaded from public whole-genome sequence databases, including NCBI Genomes (https://www.ncbi.nlm.nih.gov/genome/), Phytozome^46^, PlantGDB^47^, Ensembl Plants^48^, Sol Genomics Network^49^, and Brassica Information Portal^50^. In case that the whole-genome sequences of a plant species have not been publically available, the cDNA sequences of all the homologous sequences of each gene can be obtained from the plant genome of interest by using transcriptomics data or rapid amplification of cDNA ends (RACE). cDNA sequence alignment of all these homologous sequences permits SNP-based sequence-specific primer design. Primer specificity can be experimentally validated by PCR, Sanger sequencing without cloning, and melting curves as shown in Figs. S18 and S19.

**Fig. 9:**
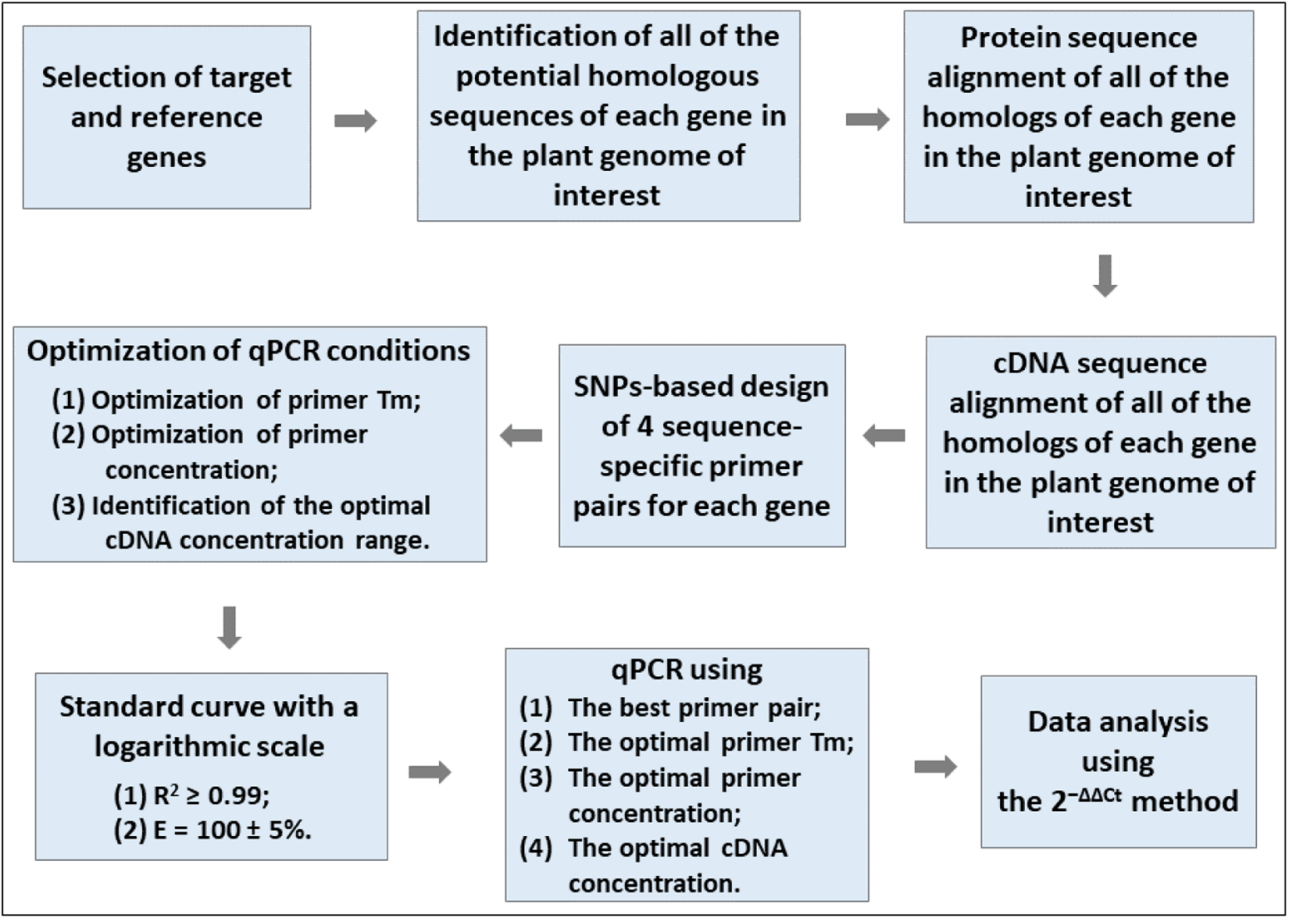
Guideline for performing qPCR analysis in this study. Tm, annealing temperature

To date, the 2^−ΔΔCt^ method^15^ has been the most widely used means for data analysis for relative quantification of gene expression with the prerequisite that both the reference and target genes have an equal qPCR amplification efficiency. Many factors could affect qPCR amplification efficiency, e.g., primer sequence, primer annealing temperature, primer concentration, and cDNA (template) concentration^26,51^. However, optimization of these factors has often been overlooked in publications^22,52,53^. A number of skilled commercial providers claimed that the optimization of qPCR conditions could be skipped when using their particular master mixes^50^. Here, we proposed an optimized method by combining the efficiency calibrated^16,17^ and standard curve methods^16,18,19^ with the 2^−ΔΔCt^ method^15^ to sequentially optimize qPCR parameters for each reference and target gene using *T. ravennae* as a model (Fig. 9). By doing this, the standard cDNA concentration curve with a logarithmic scale for each gene was obtained, and *R*^2^ ≥ 0.99 and *E* = 100 ± 5% were achieved for the best primer pair of each of the eight candidate reference genes except *H3.3* (Table 1) and served as the prerequisite for using the 2^−ΔΔCt^ method for data analysis.

We applied our optimized protocol to identify a set of reference genes in *T. ravennae* under different experimental conditions. *T. ravennae* is native to regions from central Europe to North Africa and from East India to Western China. It is a valuable ornamental grass in the landscape with utility in riparian buffer zones due to its attractiveness, broad adaptability, water-deficit tolerance, disease and pest resistance, and low input requirements^28^. It is also a perennial bioenergy feedstock, which forms dense pithy clumps of up to four meters in height and produces biomass of up to 10–14 dry Mg/ha per year^54^. Besides, the whole-genome and transcriptome of *T. ravennae* have been sequenced by Illumina and PacBio technologies^29,30^. Thus, the identification of stable reference genes in *T. ravennae* is of particular interest for studying gene expression under the different conditions of developmental stages or environmental stresses. We found that *T. ravennae* was tolerant to the salinity treatments with 150 and 300 mM NaCl for up to 20 days (Fig. 4B, C, E) and could withstand water deficit when stimulated with 20% PEG 8000 for up to 5 days (Fig. 6B, D). Neither treatment resulted in insignificant changes in fresh biomass weight. Thus, *T. ravennae* is confined to be salt and drought-tolerant.

Our methods identified *BI1* and *TCTPH* to be the most stable reference genes in qPCR analysis when tested in different tissues of *T. ravennae* (Table 2). The *BI1* gene encodes for BI1-like protein which has been shown to play a role in the negative regulation of programmed cell death and apoptosis, positive regulation of leaf senescence^55,56^. *BI1* can also be induced by water-deficit stress, heat shock, mycotoxin exposure, and plant pathogens^57^. *TCTPH* has been shown to be relatively stable in orchard grass under salinity treatment^58^, even though it is responsive to a wide range of stimuli, such as mercury, heat, cold, water-deficit, salinity, abscisic acid, ethylene, and pathogens^59,60^. We also found that *EF1α* and *Aldolase* were the most stably expressed reference genes at different inflorescence developmental stages, and *Ubi4* and *Aldolase* were the most stable reference genes under salinity stress (Table 2). *EF1α* has been reported as a reference gene in 81 species and used to measure transcript abundance in different tissues^61,62^. *Aldolase* encodes a critical enzyme in the Calvin–Benson cycle (*CBC*), is sensitive to temperature, and can regulate the growth and chilling tolerance in tomato seedlings^63^. *Ubi4* has been shown to be unstable across different tissues and under abiotic stresses^64^. Here, we report that *EF1α* and *8C* are the most stable reference genes under water-deficit stress (Table 2). *8C* is an autophagy-related ubiquitin-like modifier gene conferring tolerance to nitrogen starvation^65^. When all the samples were analyzed all together, *Aldolase* and *EF1α* were the most stable genes. However, *H3.3* and *AH* should not be used as reference genes for qPCR analysis in *T. ravennae* under these experimental conditions. As a result, our results indicated that neither the housekeeping genes nor the newly discovered genes have consistent stable expression levels under different conditions. We highly recommend that different pairs of genes be used for qPCR analysis under different conditions.

We also applied our optimized protocol to test the expression stability of a set of reference genes in two soybean varieties under biotic stress with Xag, the foliar bacterial pathogen causing BLP disease in soybean^40^. BLP is a worldwide disease, causing a significant reduction in soybean production and quality—especially under high temperature and humidity^66–68^. Williams 82 (W82) is BLP-resistant^36,37^ and we confirmed that Jack was BLP-susceptible in the present study (Fig. 8A). It was reported that *Actin*, *Tubulin*, *cons4*, and *cons6* could be used as reference genes in soybean at 12 hpt with Xag treatment^38,39^. Time-course analysis of the six candidate reference genes in W82 and Jack at 0, 12, 24, 48, 72, and 120 hpt with Xag revealed that *cons6* and *cons4* were the most stably expressed reference genes, while *Tubulin* was the least stable under all the experimental conditions. *cons6*, an F‐box protein family gene, was identified as the most stable gene in soybean under the infection with *Heterodera glycine*^69^. In contrast, *cons4*, an ATP‐binding cassette transporter gene, was identified as one of the most stable expressed genes in soybean infected with *Phytophthora sojae*^70^. Our data support the use of *cons6* and *cons4* a pair of reference genes for studying the Xag-soybean pathosystem.

As a result, a guideline for performing qPCR analysis is summarized in Fig. 9: (i) selection of target and reference genes; (ii) identification of all the homologous sequences in a plant genome of interest; (iii) SNP-based sequence-specific primer design; (iv) optimization of primer annealing temperature; (v) optimization of primer concentration; (vi) identification of the optimal cDNA concentration range and the best primer pair to achieve *R*^2^ ≥ 0.99 and efficiency (*E*) = 100 ± 5%; (vii) qPCR analysis using the optimal annealing temperature, primer concentration, and cDNA concentration for the best primer pair; (viii) data analysis using the 2^−ΔΔCt^ method. In the best interest of science and data reproducibility, we recommend that all these pieces of information be disclosed whenever qPCR results are published.

## Materials and methods

### Plant materials and growth conditions

Seed of *T. ravennae* collected from a single landscape plant at the JC Raulston Arboretum (4415 Beryl Road Raleigh, NC 27606-1457, USA) in the fall of 2018 was used for clone propagation. Caryopses were removed from mature inflorescences, sterilized for 18–21 min with periodic agitation in a 20% commercial bleach solution (6.15% sodium hypochlorite) containing a drop of Tween 20, and rinsed with sterilized water three times. Disinfected seeds were placed on culture media containing the complete Murashige and Skoog (MS) vitamins and one-half of the basal salinity formulation with an additional 30 g L^−1^ sucrose, 5 µM benzylaminopurine (BAP), and 100 mg L^‒1^ 2-(N-Morpholino) ethanesulfonic acid (MES). Media were adjusted to a pH of 5.75 and solidified with 6.5 g L^‒1^ agar (Phytotechnology Laboratories; Shawnee Mission, KS, USA) before autoclaving. Eight-week-old clones were transferred to MS media containing 30 g L^−1^ sucrose, 20 µM BAP, and 100 mg L^‒1^ MES for further experimentation. Shoots were maintained by transferring to fresh media once per 4 weeks and grown at 23 ± 2 °C with a 16-h photoperiod of 110 μmol m^‒2^ s^‒1^ provided by cool white fluorescent lamps.

Seeds of soybean cvs. W82 and Jack were germinated and grown in 1-Gal pots in a growth chamber at 25–28 °C with a 16-hour photoperiod of 520 μmol m^‒2^ s^‒1^.

### Salinity and water-deficit stress assays and growth performance analysis in *T. ravennae*

The *T. ravennae* clones grown on solid MS media in PYREX^®^ 55-ml test tubes (25 × 150 mm; Corning, AZ, USA)^71^ were transferred to liquid MS media (pH 5.75) containing 30 g L^−1^ sucrose, 100 mg L^−1^ MES, and 4 µM trans-zeatin plus different concentrations of NaCl or polyethylene glycol (PEG) 8000 (PhytoTech; Lenexa, KS, USA). NaCl was used to simulate varying degrees of salinity stress at the concentrations of 0 (control), 150, 300, and 600 mM at the time course of 0, 10, 20, and 30 dpt, while PEG 8000 was used to simulate different levels of water-deficit stress at the concentrations of 0 (control), 20% and 40% (w/v) at the time course of 0, 5, and 10 dpt. These concentrations and duration of treatments of NaCl and PEG 8000 were confirmed by a preliminary experiment with *T. ravennae* clones. The experiment was conducted as a completely randomized design consisting of three biological replicates per treatment. At each time point for each treatment, time-course analysis of phenotypic changes of the clones was conducted, fresh biomass weight was measured, and leaf samples were collected for total RNA extraction.

### Biotic stress treatment in soybean with Xag

Pathogenic Xag strain EB08^40^ was used for all biotic stress experiments in the present study. For inoculation experiments, EB08 was struck onto nutrient agar (NA) medium (BBL, Becton Dickinson and Co., Cockeysville, MD) from a 30% glycerol solution stored at −80 °C and incubated at 28 °C for 24 h. A single colony was then transferred to nutrient broth (BBL, Becton Dickinson and Co., Cockeysville, MD) where it was allowed to grow at 28 °C for 24 h with shaking at 225 rpm. The overnight bacteria culture was washed two times with water by centrifugation at 4000 × *g* for 10 min. The final pellet was resuspended in 10 mM MgCl_2_ buffer and diluted to a final concentration of 1 × 10^8^ CFU/mL (OD_600_ = 0.3–0.5). Using a 1-ml syringe without a needle, bacteria suspensions were leaf infiltrated at eight different locations on the abaxial side of the first trifoliate leaves of 1-month-old soybean plants. The same soybean plants were also spray inoculated using an atomizer. The inoculated plants were wrapped in plastic bags in the dark overnight. In total, 10 mM MgCl_2_ buffer was used as the mock treatment. Three biological replications were used for each treatment. One set of leaf samples were collected for RNA isolation and a second set for bacterial population counts to confirm pathogen infection (with the necrotic leaf tissues being removed) at 0, 12, 24, 48, 72, and 120 hpt. Immediately after tissue collection, all samples were placed in liquid nitrogen and stored in −80 °C until further processing.

Bacterial population sizes in soybean tissues were determined by homogenizing approximately 20 mg of leaf tissue in an OMNI bead ruptor elite bead homogenizer (OMNI International, Kennesaw, Georgia, USA). Samples were ground at room temperature in 2.0 ml screw-cap tubes with 1-ml sterile DI water and two 2.4 mm OMNI metal beads. Tubes containing samples were placed in the homogenizer and run on a speed cycle of 3.4 m/s for 30 s with a dwell of 5 s between cycles. Each cycle was repeated three times. Homogenate was serially diluted and plated in triplicates on NA media, and colonies were counted after 48 h of incubation at 28 °C. Each experiment consisted of three biological replicates per treatment.

### RNA isolation and cDNA synthesis

Total RNA was extracted from six tissues (root, vegetative meristem, leaf, inflorescence, flower, and immature seed) and five inflorescence developmental stages (the inflorescence of 40, 80, 120, 160, and 200 cm in length) of *T. ravennae*, using the TRIzol^®^ reagent (Molecular Research Center; Cincinnati, OH, USA) according to the manufacturer’s instructions. Total RNA was also extracted from the leaf samples of *T. ravennae* collected from the salinity and water-deficit treatments and from the leaf samples of soybean collected from Xag treatments. About 100 mg of each sample with three biological replicates were used for total RNA extraction. RNA samples were treated with DNase I (New England Biolabs; Ipswich, MA, USA) to remove genomic DNA contaminations, followed by the cleanup with the GeneJET RNA Cleanup and Concentration Micro Kit (Thermo Fisher; Waltham, MA, USA). The total RNA concentration and quality of each sample were assessed using a Nanodrop ND-1000 spectrophotometer (NanoDrop Technologies; Wilmington, DE, USA) and gel electrophoresis. The RNA with 260/280 ratio between 1.9 and 2.1, 260/230 ratio greater than 2.0, and clear bands for 28S and 18S after running the RNA on an agarose gel were used for cDNA Synthesis.

Synthesis of cDNA was performed by RT-PCR from 1000 ng of total RNA using the High Capacity cDNA Reverse Transcription Kit (Thermo Fisher; Waltham, MA, USA). Specifically, 1000 ng of total RNA was mixed with 2 µl of 10× RT Buffer, 2.0 µl 10× RT Random Primers, 0.8 µl of 25× dNTP Mix (100 mM), and 1 µl MultiScribe^®^ Reverse Transcriptase (50 units/µl) in a total volume of 20 µl. The mixture was incubated for 10 min at 25 °C, 120 min at 37 °C, and 5 s at 85 °C. The resulting cDNA was stored at −20 °C.

### Selection of candidate reference genes based on their digital expression profiles in *T. ravennae*

Candidate reference genes were selected from a tissue-specific Illumina RNA-Seq dataset of *T. ravennae*^30^ based on their relatively stable normalized read accounts (reads per Kb per million mapped (RPKMs)) in the root, vegetative meristem, inflorescence, flower, and seed. The RNA-Seq dataset was obtained from a de novo assembly of all the expression dataset of transcripts, followed by filtering the transcripts with a minimum expression value of 200 transcripts per million (TPM), a mean value of less than 2000 TPM, and a coefficient of variation (CV, %) <0.35. Once selected, each candidate reference gene was used as the query sequence to blast against a tissue-specific Pacific BioSciences (PacBio) Iso-Seq dataset of *T. ravennae*^30^ (Protocol # 101-070-200 version 6) in the same tissues as above, and their full-length transcript sequence reads were obtained.

### Identification of the homologous sequences of each candidate reference gene in the genomes of *T. ravennae* and soybean

Using GMAP^72^, the full-length PacBio sequence of each candidate reference gene was mapped to a primary reference genome assembly generated from the whole-genome sequencing of *T. ravennae*^29^. Subsequently, the PacBio sequence of each candidate reference gene was used as the query sequence to search against the cDNA and deduced protein sequence data sets of *T. ravennae*^29,30^ using BLASTN and BLASTX, respectively. The returned protein sequences of all the potentially homologous sequences of each PacBio sequence were used for protein sequence alignment using ClustalX 2.0. The sequences that lacked sequence homology were filtered out. The remaining homologs of each PacBio sequence were used for cDNA sequence alignment using their returned cDNA sequences and ClustalX 2.0. SNPs were identified between each PacBio sequence and its most similar homolog in the genome and used for gene-specific primer design for each gene.

The protein sequence of each candidate reference gene in soybean was obtained from the soybean genome in the Phytozome database^46^ (v12.1; https://phytozome.igi.doe.gov/pz/portal.html) and used as the query sequence to search against the soybean whole-genome sequence in Phytozome using TBLASTN. By doing this, the cDNA and deduced protein sequences of all the potential homologous sequences of each candidate reference gene were downloaded from the soybean whole-genome sequence database in Phytozome. The deduced protein sequences of all the returned homologous sequences of each gene were used for protein sequence alignment using ClustalX 2.0 (http://www.clustal.org/). After the homologous sequences that lacked sequence homology were removed, the remaining homologous sequences of each gene were used for both protein sequence alignment and cDNA sequence alignment by using their cDNA (including 5’- and 3’-UTR) sequences (Fig. S1). The cDNA sequence alignment could be adjusted manually according to the protein sequence alignment. The SNPs present in each gene’s cDNA alignment with its homologs were used for gene-specific primer design for each gene.

### Validation of the accuracy of the *T. ravennae* PacBio sequences by PCR and Sanger sequencing without cloning

Two forward and two reverse primers, which formed four primer pairs, were designed for each candidate reference gene in order to PCR amplify both the PacBio sequence and its most similar homolog simultaneously with SNPs present between the PacBio sequence and its most similar homolog between the locations of the primer pairs (Figs. S10–S17, S26–S31). By doing this, the Sanger sequencing of the PCR products without cloning would indicate whether the PacBio sequence, its most similar homolog, or both copies (by showing double peaks on the SNPs) were PCR amplified. Gradient PCR with different annealing temperatures (54.6, 56, 58, 60.6, and 62.7 °C) was conducted on clear plastic 96-well plates with optical film and Bio-Rad CFX96 real-time PCR thermocycler (Bio-Rad; Hercules, CA, USA) for each primer pair for each PacBio sequence, the diluted leaf cDNA (1:10 dilution) as the templates, and the SYBR Select Master Mix kit (Thermo Fisher; Waltham, MA, USA). The primer concentration of 350 mM per primer per reaction was used. Under these conditions, the Ct values were expected to be 15 < Ct < 32. In case the Ct values would be smaller than 15 cycles or larger than 32 cycles, the dilution factors of the leaf cDNA would be adjusted accordingly (primer concentration could also be adjusted thereafter). Confirmation of a single PCR product per primer pair was conducted by gel electrophoresis, followed by PCR product purification using the QIAquick Gel Extraction Kit (Qiagen, Hilden, North Rhine-Westphalia, Germany) and Sanger sequencing without cloning. The accuracy of each PacBio sequence was analyzed by aligning the sequencing results with their respective PacBio sequence using Sequencher (Gene Codes, Ann Arbor, MI, USA).

### Primer design for qPCR

The SNPs identified between each PacBio sequence and its most similar homolog in the *T. ravennae* genome were used for primer design for qPCR. The SNPs identified between each of the soybean candidate reference genes and its most similar homologs in the soybean genome were used for qPCR primer design. With the SNPs being located on the last position (or more positions including the last one) at the 3’-end of all the primers, two forward primers of 20–23 bp in length were designed next to each other for each candidate reference gene. Similarly, two reverse primers were designed next to each other for the same candidate gene with the PCR amplicons being 85 – 125 bp in length (including the length of the two primers) (Table S1). The PCR amplicons could be longer than 125 bp in length if sequence homology prevents the design of sequence-specific primers for PCR amplicons of 85–125 bp in length. Thus, four sequence-specific primer pairs were designed for each candidate reference gene. Exceptions were *Ubi4*, *Aldolase*, *BI1*, *8C*, and *AH*; all or half of their primers used for PCR validation were also used for qPCR.

### Optimization of qPCR conditions

After the validation of primer specificity by PCR, gel electrophoresis, and Sanger sequencing, qPCR conditions were optimized for each of the four primer pairs for each candidate reference gene. All the qPCR reactions had three technical replicates. First, we used gradient PCR (56, 58, 60.6, and 62.7 °C) to test the optimal annealing temperature for each primer pair by using the diluted cDNA (1:10 dilution) as the templates and primer concentration of 350 mM per primer per reaction. The optimal annealing temperature for each primer pair was the one that had the lowest Ct value. Second, we used different primer concentrations (250, 300, 350, and 400 mM per primer per reaction for each primer) to determine the optimal primer concentration for each primer pair by using the diluted cDNA (1:10 dilution) as templates and the optimal annealing temperature. The optimal primer concentration for each primer pair was the one that had the lowest Ct value. Lastly, we used the optimal annealing temperature and primer concentration for each primer pair, and serial dilutions of the cDNA (1:10, 1:20, 1:40, 1:80, and 1:160 dilutions) to obtain the standard cDNA concentration curve with a logarithmic scale. Serial dilution means a stepwise dilution of cDNA using the same dilution factor (specifically, the original cDNA was diluted by a ratio of 1 to 10 to make the 1:10 dilution, which was diluted by a ratio of 1 to 2 to make the 1:20 dilution; the 1:20 dilution was used to make the 1:40 dilution, which was used to make the 1:80 dilution, and the 1:80 dilution was used to make the 1:160 dilution). Each step of dilution should be mixed very well by gently pipetting about 30 times while spinning down should be avoided. Thus, the initial cDNA concentration could be calculated by dividing 1000 ng RNA by 20 µl cDNA (i.e., 50 ng/µl), and the serially diluted cDNA concentrations could be calculated by dividing the initial cDNA concentration by the dilution factors (i.e., 5, 2.5, 1.25, 0.625, 0.3125 ng/µl).

The averaged Ct values from three technical replicates were plotted against the Log (cDNA in ng/reaction), which were 0.69897, 0.39794, 0.09691, −0.20412, and −0.50515, respectively. The standard curve equation *y* = A*x* + B and *R*^2^ were obtained in a spreadsheet for each primer pair. The PCR efficiency (E, %) for each primer pair was calculated as *E* = (10^−1/A^ − 1) × 100. The outliers from the lowest (or highest) one (or two) cDNA concentration might have been removed in order to obtain *R*^2^ ≥ 0.99 and *E* = 100 ± 5% for the data from the remaining four (or three) consecutive cDNA concentrations for each primer pair for each candidate gene. This served as the prerequisite for using the 2^−ΔΔCt^ method for data analysis.

### qPCR

The primer pair that had the best *R*^2^ and *E* for each candidate gene was used for qPCR analysis in order to quantify the transcript abundance of each gene in different tissues or under different abiotic and biotic stress treatments. For each qPCR reaction, 10 µl PCR reaction volume was used, which contained 5 µl of SYBR Select Master Mix, 0.3 µl of each primer, 1 µl of diluted cDNA, and 3.4 µl of ddH_2_O (all the components except SYBR Select Master Mix should be mixed and spun down for three times in a master tube for every three technical replicates, and aliquoted into three wells on a clear plastic 96-well plate (Bio-Rad, Hercules, CA, USA), followed by the addition of SYBR Select Master Mix, and mixing by gentle pipetting for at least 30 times). PCR cycling was performed as follows: 2 min at 95 °C followed by 39 rounds of 5 s at 95 °C, 30 s at the optimal annealing temperature and finally, 1 cycle of 5 s at 65 °C. A melting curve (65–95 °C; at increments of 0.5 °C) was generated to verify the specificity of primer amplification. Three biological replicates were used with three technical replicates for each tissue sample to monitor possible sampling error and experimental error.

### Data analysis for transcript abundance of the candidate reference genes

Raw fluorescence Ct values were acquired from qPCR experiments and analyzed by Bio-Rad CFX Manager 3.1 (Bio-Rad, Hercules, CA, USA) and in a spreadsheet. The average Ct value from the three technical replicates per biological replicate for each candidate gene was calculated for each biological replicate of each tissue sample. The coefficient of variation (CV, %) of the Ct values of each candidate gene was calculated for different samples individually and in combination.

### Analysis of the stability of expression of the candidate reference genes

To analyze the stability of expression of the candidate reference genes, we used RefFinder, a web-based tool that integrates Delta Ct^73^, NormFinder^74^, BestKeeper^75^, and geNorm^76^ together to obtain a comprehensive stability ranking of all the candidate reference genes^35^. Mean Ct values were used as the input, and data analysis was conducted using the web-based software package of qbase + . RefFinder runs each of the algorithms, assigns a weight to each gene based on its rankings by each method, and calculates the geometric mean of its weights as the overall final ranking score.

### Identification of the optimal number of reference genes in *T. ravennae* and soybean

The pairwise variation (V_n/n+1_) of the candidate reference genes under various conditions was calculated by geNorm^76^, and the optimal number of reference genes was determined by the lowest number of genes with V_n_/V_n+1_ smaller than the threshold of 0.15. This indicates the use of the *n* most stable candidate genes as the reference genes is adequate to ensure the accurate normalization of qPCR data, and the addition of one more candidate gene will not make a significant difference.

## Supplementary information

An optimized protocol for stepwise optimization of real-time RT-PCR analysis
