# Supplementary material for: An optimized protocol for stepwise optimization of real-time RT-PCR analysis
Source: Hortic Res. 2021 Aug 1;8:179. doi: 10.1038/s41438-021-00616-w (PMC8325682; doi:10.1038/s41438-021-00616-w)
Supplement: Supplementary file 1 — An optimized protocol for stepwise optimization of real-time RT-PCR analysis [file 41438_2021_616_MOESM1_ESM.doc]

**Supplemental Information – Zhao et al. An optimized protocol for real-time RT-PCR analysis**

**Table S1. Primer sequences used in the present study.**

| **Gene** | **Function** | **Primer Name^a^** | **Primer Sequence (5′–3′)** | **Purpose** |
| --- | --- | --- | --- | --- |
| *EF1α* | Elongation Factor 1 Alpha | **EF1α-qPCR-F1** | **ACCTGATCTACAAGCTTGGC** | qPCR |
|  |  | EF1α-qPCR-F2 | TGATCTACAAGCTTGGCGGT | qPCR |
|  |  | EF1α-qPCR-R1 | ATGACCGCTTGTTCATCTCC | qPCR |
|  |  | **EF1α-qPCR-R2** | **GCACCCACGCATACTTGAAT** | qPCR |
| *Ubi4* | Ubi4-like- Polyubiquitin | Ubi4-F1 | GATCTTCGTTAAGACCCTCAC | PCR, Sequencing; qPCR |
|  |  | **Ubi4-F2** | **TCTTCGTTAAGACCCTCACT** | PCR, Sequencing; qPCR |
|  |  | Ubi4-R1 | GGATCTTAGCCTTGACATTGT | PCR, Sequencing; qPCR |
|  |  | **Ubi4-R2** | **CTGGATCTTAGCCTTGACATT** | PCR, Sequencing; qPCR |
| *H3.3* | Histone H3.3 isoform X1 | H3.3-qPCR-F1 | TTCCAGAGGCTTGTCAGGGAA | qPCR |
|  |  | **H3.3-qPCR-F2** | **GGCTTGTCAGGGAAATTGCA** | qPCR |
|  |  | H3.3-qPCR-R1 | CAAACAGGCCAACAAGGTAT | qPCR |
|  |  | **H3.3-qPCR-R2** | **TGTCCTCAAACAGGCCAACA** | qPCR |
| *TCTPH* | Translationally-controlled tumor protein homolog | **TCTPH-qPCR-F1** | **TCCGACTCCTTCCAGTACAA** | qPCR |
|  |  | TCTPH-qPCR-F2 | ACTCCTTCCAGTACAAGGAG | qPCR |
|  |  | **TCTPH-qPCR-R1** | **ACATCGACAGGTCCTTGGAC** | qPCR |
|  |  | TCTPH-qPCR-R2 | CAATATCCACATCGACAGGT | qPCR |
| *Aldolase* | Fructose-bisphosphate Aldolase | Aldolase-F1 | AATGCCATGAACAAGCTCAG | PCR, Sequencing; qPCR |
|  |  | **Aldolase-F2** | **TGAACAAGCTCAGCACCAAG** | PCR, Sequencing; qPCR |

^a^The best primer pair for qPCR of each gene.

**Table S1. (Cont.)**

| **Gene** | **Function** | **Primer Name^a^** | **Primer Sequence (5′–3′)** | **Purpose** |
| --- | --- | --- | --- | --- |
|  |  | **Aldolase-R1** | **GCCTTCTCAATGTTCTCCAC** | PCR, Sequencing; qPCR |
|  |  | Aldolase-R2 | AGCCTTCTCAATGTTCTCCA | PCR, Sequencing; qPCR |
| *BI1* | BI1-like protein | BI1-F1 | CTGGAGGCTTTAGTATTGACT | PCR, Sequencing |
|  |  | BI1-F2 | GCTTTAGTATTGACTGCTGG | PCR, Sequencing |
|  |  | BI1-R1 | CTGAGAAGACCAAAGCTCCT | PCR, Sequencing; qPCR |
|  |  | **BI1-R2** | **AGCCTGAGAAGACCAAAGCT** | PCR, Sequencing; qPCR |
|  |  | **BI1-qPCR-F1** | **TGTCCTTGTCCTAACTAGCT** | qPCR |
|  |  | BI1-qPCR-F2 | CCTTGTCCTAACTAGCTTTC | qPCR |
| *8C* | Autophagy  -related protein 8C | 8C-F1 | GTGATTGTTGAGAAGGCTGA | PCR, Sequencing; qPCR |
|  |  | **8C-F2** | **TGATTGTTGAGAAGGCTGAG** | PCR, Sequencing; qPCR |
|  |  | 8C-R1 | AGATAGCCTTCTCAGCACTG | PCR, Sequencing |
|  |  | 8C-R2 | GATGAAGATAGCCTTCTCAGC | PCR, Sequencing |
|  |  | 8C-qPCR-R1 | GCTTGATTCTCTTCCTAACG | qPCR |
|  |  | **8C-qPCR-R2** | **CTGAGCTTGATTCTCTTCCTA** | qPCR |
| *AH* | Aconitate  hydratase | **AH-F1** | **TGAGATACAAATCTGAGGGC** | PCR, Sequencing; qPCR |
|  |  | AH-F2 | TACAAATCTGAGGGCCATGC | PCR, Sequencing; qPCR |
|  |  | AH-R1 | AAGTTGCTTCTGTGGATACG | PCR, Sequencing |
|  |  | AH-R2 | CCAAGTTGCTTCTGTGGATA | PCR, Sequencing |
|  |  | AH-qPCR-R1 | CTTTAACTCCCAGGAGCATC | qPCR |

^a^The best primer pair for qPCR of each gene.

**Table S1. (Cont.)**

| **Gene** | **Function** | | | **Primer Name^a^** | | **Primer Sequence (5′–3′)** | | **Purpose** |  |
| --- | --- | --- | --- | --- | --- | --- | --- | --- | --- |
|  | |  | **AH-qPCR-R2** | | **TCACAGCTTTAACTCCCAGG** | | qPCR | | |
| *Actin* | Cell cytoskeleton and movement | | | GmActin-qPCR-F1 | | GTCTCTGTATGCCAGTGGAA | | qPCR |  |
|  |  |  |  | **GmActin-qPCR-F2** | | **CTCTGTATGCCAGTGGAAGA** | | qPCR |  |
|  |  |  |  | GmActin-qPCR-R1 | | ACCCTTCATATATGGGAACT | | qPCR |  |
|  |  |  |  | **GmActin-qPCR-R2** | | **GCATACCCTTCATATATGGGA** | | qPCR |  |
| *cons4* | ATP-binding cassette transporter | | | **Gmcons4-qPCR-F1** | | **TCCATGGATCTTGCTCCATT** | | qPCR |  |
|  |  |  |  | Gmcons4-qPCR-F2 | | GGATCTTGCTCCATTGATGAT | | qPCR |  |
|  |  |  |  | Gmcons4-qPCR-R1 | | ACTCTCTTCCATCCTCTTGG | | qPCR |  |
|  |  |  |  | **Gmcons4-qPCR-R2** | | **TGCAGAACACAACCAAATCA** | | qPCR |  |
| *cons6* | F-box protein family | | | **Gmcons6-qPCR-F1** | | **AAGTTAGGAGCCCAAGACAT** | | qPCR |  |
|  |  |  |  | Gmcons6-qPCR-F2 | | TTAGGAGCCCAAGACATTGCG | | qPCR |  |
|  |  |  |  | **Gmcons6-qPCR-R1** | | **AGCGAGTTCATTGAAGCAGA** | | qPCR |  |
|  |  |  |  | Gmcons6-qPCR-R2 | | GTTGTGTCAAAGCGAGTTCA | | qPCR |  |
| *Tubulin* | Microtubule | | | **GmTubulin-qPCR-F1** | | **GCCACTGGAAATTACGTAGGT** | | qPCR |  |
|  |  |  |  | GmTubulin-qPCR-F2 | | TGGAAATTACGTAGGTAACTT | | qPCR |  |
|  |  |  |  | **GmTunlin-qPCR-R1** | | **TCGAGGAACATACCGTCCC** | | qPCR |  |
|  |  |  |  | GmTunlin-qPCR-R2 | | TAAGCACAGCTCGAGGAACA | | qPCR |  |
| *FtsZ2-1* | Cell division | | | **Gm194800-qPCR-F1** | | **AGAATCACGGTATCAGATCG** | | qPCR |  |
|  |  |  |  | Gm194800-qPCR-F2 | | CGGTATCAGATCGTGCTTCT | | qPCR |  |

^a^The best primer pair for qPCR of each gene.

**Table S1. (Cont.)**

| **Gene** | **Function** | **Primer Name^a^** | **Primer Sequence (5′–3′)** | **Purpose** |
| --- | --- | --- | --- | --- |
|  |  | Gm194800-qPCR-R1 | ATTCGCGGAACACTTCACTG | qPCR |
|  |  | **Gm194800-qPCR-R2** | **TGATGCTGTGGGAATTCGCG** | qPCR |
| *TUBB* | Microtubule | **Gm023900-qPCR-F1** | **ACAACGAGGAAGGCAACAGCA** | qPCR |
|  |  | Gm023900-qPCR-F2 | AACGAGGAAGGCAACAGCAG | qPCR |
|  |  | **Gm023900-qPCR-R1** | **AGATCCATAAGCACGGCACGG** | qPCR |
|  |  | Gm023900-qPCR-R2 | TCGAGATCCATAAGCACGGCA | qPCR |

^a^The best primer pair for qPCR of each gene.

**Table S2. Transcript abundance of the eight candidate reference genes in different tissues and at different inflorescence stages of *T. ravennae*.**

| **Gene** | **Tissues** | | |  | **Inflorescence Developmental Stages** | | |
| --- | --- | --- | --- | --- | --- | --- | --- |
|  | **Ct** | **SD** | **CV (%)** |  | **Ct** | **SD** | **CV (%)** |
| *EF1α* | 23.46 | 1.45 | 6.16 |  | 22.59 | 0.34 | 1.49 |
| *Ubi4* | 22.17 | 0.31 | 1.39 |  | 21.63 | 0.44 | 2.01 |
| *H3.3* | 23.66 | 1.05 | 4.43 |  | 22.87 | 0.30 | 1.32 |
| *TCTPH* | 24.83 | 1.20 | 4.85 |  | 23.17 | 0.76 | 3.28 |
| *Aldolase* | 23.15 | 1.24 | 5.37 |  | 22.06 | 0.50 | 2.25 |
| *BI1* | 25.22 | 0.80 | 3.16 |  | 23.76 | 1.07 | 4.51 |
| *8C* | 26.77 | 0.88 | 3.30 |  | 25.16 | 1.01 | 4.00 |
| *AH* | 28.47 | 1.07 | 3.77 |  | 27.89 | 0.84 | 3.01 |

Ct, the average Ct value of each candidate reference gene in different tissues at various floral developmental stages. SD, the standard deviation. CV, coefficient of variation. Three biological replicates per sample were used.

**Table S3. Transcript abundance of the eight candidate reference genes in *T. ravennae* under salinity treatments.**

| **Gene** | **Control** | | |  | **150 mM NaCl** | | |  | **300 mM NaCl** | | |  | **600 mM NaCl** | | |
| --- | --- | --- | --- | --- | --- | --- | --- | --- | --- | --- | --- | --- | --- | --- | --- |
|  | **Ct** | **SD** | **CV (%)** |  | **Ct** | **SD** | **CV (%)** |  | **Ct** | **SD** | **CV (%)** |  | **Ct** | **SD** | **CV (%)** |
| *EF1α* | 22.65 | 0.36 | 1.58 |  | 23.62 | 1.02 | 4.31 |  | 24.16 | 1.79 | 7.40 |  | 26.43 | 4.99 | 18.87 |
| *Ubi4* | 20.93 | 0.32 | 1.52 |  | 23.05 | 2.69 | 11.66 |  | 23.76 | 3.69 | 15.52 |  | 24.85 | 5.23 | 21.06 |
| *H3.3* | 23.08 | 0.51 | 2.22 |  | 25.49 | 2.90 | 11.36 |  | 26.83 | 4.79 | 17.85 |  | 26.67 | 4.57 | 17.15 |
| *TCTPH* | 23.56 | 0.29 | 1.24 |  | 25.32 | 2.20 | 8.68 |  | 25.78 | 2.85 | 11.04 |  | 27.64 | 5.47 | 19.80 |
| *Aldolase* | 23.35 | 0.14 | 0.62 |  | 24.80 | 2.20 | 8.86 |  | 25.30 | 2.91 | 11.49 |  | 27.61 | 6.17 | 22.35 |
| *BI1* | 24.52 | 0.13 | 0.54 |  | 25.78 | 1.92 | 7.46 |  | 26.32 | 2.68 | 10.20 |  | 29.47 | 7.14 | 24.21 |
| *8C* | 26.07 | 0.63 | 2.40 |  | 26.70 | 0.27 | 0.99 |  | 27.56 | 1.49 | 5.40 |  | 29.85 | 4.73 | 15.83 |
| *AH* | 24.76 | 0.48 | 1.95 |  | 26.40 | 1.84 | 6.96 |  | 27.31 | 3.13 | 11.45 |  | 29.57 | 6.33 | 21.39 |

Ct, the average Ct value of each candidate reference gene under salinity stress treatment. SD, the standard deviation. CV, coefficient of variation. Three biological replicates per sample were used.

**Table S4. Transcript abundance of the eight candidate reference genes in *T. ravennae* under water-deficit treatments.**

| **Gene** | **Control** | | |  | **20% PEG 8000** | | |  | **40% PEG 8000** | | |
| --- | --- | --- | --- | --- | --- | --- | --- | --- | --- | --- | --- |
|  | **Ct** | **SD** | **CV (%)** |  | **Ct** | **SD** | **CV (%)** |  | **Ct** | **SD** | **CV (%)** |
| *EF1α* | 22.58 | 0.28 | 1.24 |  | 23.97 | 2.14 | 8.92 |  | 24.88 | 3.11 | 12.51 |
| *Ubi4* | 21.01 | 0.27 | 1.27 |  | 21.85 | 1.72 | 7.86 |  | 22.56 | 2.22 | 9.83 |
| *H3.3* | 22.97 | 0.40 | 1.76 |  | 24.66 | 2.49 | 10.11 |  | 25.66 | 3.64 | 14.17 |
| *TCTPH* | 23.56 | 0.21 | 0.88 |  | 25.13 | 2.51 | 9.98 |  | 26.15 | 3.94 | 15.07 |
| *Aldolase* | 23.43 | 0.18 | 0.75 |  | 24.31 | 1.66 | 6.83 |  | 26.16 | 4.29 | 16.40 |
| *BI1* | 24.45 | 0.15 | 0.61 |  | 25.79 | 2.29 | 8.89 |  | 26.55 | 3.87 | 14.58 |
| *8C* | 26.08 | 0.44 | 1.70 |  | 27.06 | 1.92 | 7.08 |  | 27.80 | 2.95 | 10.59 |
| *AH* | 24.82 | 0.36 | 1.45 |  | 25.32 | 1.15 | 4.53 |  | 26.59 | 3.11 | 11.70 |

Ct, the average Ct value of each candidate reference gene under water-deficit stress treatment. SD, the standard deviation. CV, coefficient of variation. Three biological replicates per sample were used.

**Table S5. The optimized qPCR conditions for the six candidate reference genes in soybean under Xag treatment**.

| **Gene** | **Primer Pair^a^** | **Amplicon Length (bp)** | **Optimal Tm (°C)^b^** | **Optimal Primer Concentration (mM)** | **cDNA Dilution** | **R^2^** | **E (%)^c^** |
| --- | --- | --- | --- | --- | --- | --- | --- |
| *Actin* | GmActin-qPCR-F1/ GmActin-qPCR-R1 | 86 | 58.0 | 400 | 1/20 - 1/160 | 0.9912 | 110.3 |
|  | GmActin-qPCR-F1/ GmActin-qPCR-R2 | 90 | 58.0 | 350 | 1/20 - 1/160 | 0.9928 | 106.8 |
|  | GmActin-qPCR-F2/ GmActin-qPCR-R1 | 84 | 58.0 | 350 | 1/20 - 1/160 | 0.9863 | 102.5 |
|  | **GmActin-qPCR-F2/ GmActin-qPCR-R2** | **88** | **58.0** | **300** | **1/40 - 1/160** | **0.9902** | **103.6** |
| *cons4* | Gmcons4-qPCR-F1/ Gmcons4-qPCR-R1 | 95 | 60.6 | 350 | 1/10 - 1/80 | 0.9175 | 102.1 |
|  | Gmcons4-qPCR-F1/ Gmcons4-qPCR-R2 | 129 | 60.6 | 350 | 1/10 - 1/40 | 0.9941 | 106.9 |
|  | Gmcons4-qPCR-F2/ Gmcons4-qPCR-R1 | 90 | 60.6 | 400 | 1/20 - 1/160 | 0.9789 | 118.1 |
|  | **Gmcons4-qPCR-F2/ Gmcons4-qPCR-R2** | **124** | **60.6** | **350** | **1/10 - 1/160** | **0.9852** | **102.8** |
| *cons6* | **Gmcons6-qPCR-F1/ Gmcons6-qPCR-R1** | **108** | **60.6** | **350** | **1/10 - 1/160** | **0.9912** | **104.2** |
|  | Gmcons6-qPCR-F1/ Gmcons6-qPCR-R2 | 118 | 60.6 | 350 | 1/10 - 1/80 | 0.9502 | 104.9 |
|  | Gmcons6-qPCR-F2/ Gmcons6-qPCR-R1 | 105 | 60.6 | 300 | 1/10 - 1/80 | 0.9079 | 102.0 |
|  | Gmcons6-qPCR-F2/ Gmcons6-qPCR-R2 | 115 | 60.6 | 300 | 1/10 - 1/80 | 0.9855 | 98.8 |

^a^The best primer pair for each gene was in bold. ^b^Tm, annealing temperature. ^c^E (%), efficiency.

**Table S5. (Cont.)**

| **Gene** | **Primer Pair^a^** | **Amplicon Length (bp)** | **Optimal Tm (°C)^b^** | **Optimal Primer Concentration (mM)** | **cDNA Dilution** | **R^2^** | **E (%)^c^** |
| --- | --- | --- | --- | --- | --- | --- | --- |
| *Tubulin* | **GmTubulin-qPCR-F1/ GmTunlin-qPCR-R1** | **93** | **58.0** | **300** | **1/20 - 1/160** | **0.9929** | **100.7** |
|  | GmTubulin-qPCR-F1/ GmTunlin-qPCR-R2 | 103 | 58.0 | 300 | 1/20 - 1/160 | 0.9868 | 102.8 |
|  | GmTubulin-qPCR-F2/ GmTunlin-qPCR-R1 | 88 | 58.0 | 300 | 1/10 - 1/40 | 0.9761 | 93.3 |
|  | GmTubulin-qPCR-F2/ GmTunlin-qPCR-R2 | 98 | 58.0 | 400 | 1/10 - 1/80 | 0.9997 | 84.8 |
| *FtsZ2-1* | Gm194800-qPCR-F1/ Gm194800-qPCR-R1 | 104 | 60.6 | 400 | 1/40 - 1/160 | 0.9993 | 94.3 |
|  | **Gm194800-qPCR-F1/ Gm194800-qPCR-R2** | **117** | **60.6** | **400** | 1/40 - 1/160 | **0.9975** | **100.5** |
|  | Gm194800-qPCR-F2/ Gm194800-qPCR-R1 | 97 | 60.6 | 350 | 1/40 - 1/160 | 0.9995 | 100.0 |
|  | Gm194800-qPCR-F2/ Gm194800-qPCR-R2 | 110 | 60.6 | 400 | 1/10 - 1/80 | 0.9974 | 102.2 |
| *TUBB* | **Gm023900-qPCR-F1/ Gm023900-qPCR-R1** | **103** | **60.6** | **400** | **1/20 - 1/80** | **0.9972** | **101.9** |
|  | Gm023900-qPCR-F1/ Gm023900-qPCR-F2 | 106 | 60.6 | 400 | 1/40 - 1/160 | 0.9998 | 91.1 |
|  | Gm023900-qPCR-F2/ Gm023900-qPCR-R1 | 101 | 60.6 | 250 | 1/10 - 1/40 | 0.9973 | 108.8 |
|  | Gm023900-qPCR-F2/ Gm023900-qPCR-R2 | 104 | 60.6 | 250 | 1/20 - 1/160 | 0.9894 | 98.5 |

^a^The best primer pair for each gene was in bold. ^b^Tm, annealing temperature. ^c^E (%), efficiency.

**Table S6. Transcript abundance of the six candidate reference genes in soybean under Xag treatments**.

| **Gene** | **W82 / Mock** | | |  | **W82 / Xag** | | |  | **Jack / Mock** | | |  | **Jack / Xag** | | |
| --- | --- | --- | --- | --- | --- | --- | --- | --- | --- | --- | --- | --- | --- | --- | --- |
|  | **Ct** | **SD** | **CV (%)** |  | **Ct** | **SD** | **CV (%)** |  | **Ct** | **SD** | **CV (%)** |  | **Ct** | **SD** | **CV (%)** |
| *Actin* | 29.36 | 1.63 | 5.54 |  | 29.43 | 1.39 | 4.71 |  | 29.42 | 1.55 | 5.25 |  | 29.20 | 0.98 | 3.35 |
| *cons4* | 28.80 | 0.92 | 3.21 |  | 28.59 | 0.91 | 3.18 |  | 28.66 | 0.77 | 2.67 |  | 28.73 | 0.85 | 2.95 |
| *cons6* | 29.08 | 0.70 | 2.41 |  | 28.84 | 0.82 | 2.84 |  | 28.73 | 0.73 | 2.53 |  | 28.72 | 0.92 | 3.19 |
| *Tubulin* | 30.10 | 2.23 | 7.40 |  | 30.94 | 2.73 | 8.82 |  | 29.97 | 2.20 | 7.34 |  | 30.85 | 2.90 | 9.40 |
| *FtsZ2-1* | 29.80 | 1.12 | 3.76 |  | 29.46 | 0.98 | 3.33 |  | 29.87 | 1.25 | 4.19 |  | 29.60 | 1.19 | 4.01 |
| *TUBB* | 31.44 | 1.14 | 3.63 |  | 30.70 | 1.03 | 3.34 |  | 31.42 | 1.47 | 4.68 |  | 31.04 | 1.63 | 5.25 |

Ct, the average Ct value of each candidate reference gene under salinity stress treatment. SD, the standard deviation. CV, coefficient of variation. Three biological replicates per sample were used.

**Table S7.** **Ranking of expression stability of the six candidate reference genes in soybean with Ct** **values being calculated using RefFinder.**

| **Ranking Order** | **W82** | |  | **Jack** | |  | **Total** | |
| --- | --- | --- | --- | --- | --- | --- | --- | --- |
|  | **Genes** | **Stability Value** |  | **Genes** | **Stability Value** |  | **Genes** | **Stability Value** |
| 1 | *cons6* | 1.190 |  | *cons6* | 1.000 |  | *cons6* | 1.000 |
| 2 | *cons4* | 2.060 |  | *cons4* | 1.680 |  | *cons4* | 1.860 |
| 3 | *FtsZ2-1* | 2.380 |  | *FtsZ2-1* | 3.660 |  | *FtsZ2-1* | 2.910 |
| 4 | *TUBB* | 3.460 |  | *Actin* | 3.720 |  | *TUBB* | 4.160 |
| 5 | *Actin* | 5.000 |  | *TUBB* | 4.400 |  | *Actin* | 4.470 |
| 6 | *Tubulin* | 6.000 |  | *Tubulin* | 6.000 |  | *Tubulin* | 6.000 |

**Figure S1. The digital expression levels of the eight candidate reference genes in different tissues of *T. ravennae* obtained from tissue-specific Illumina data^30^**. RNA was extracted from the root, vegetative meristem, inflorescence, flower, and seed. Normalized reads of each gene per Kb per million mapped reads (RPKM).

**
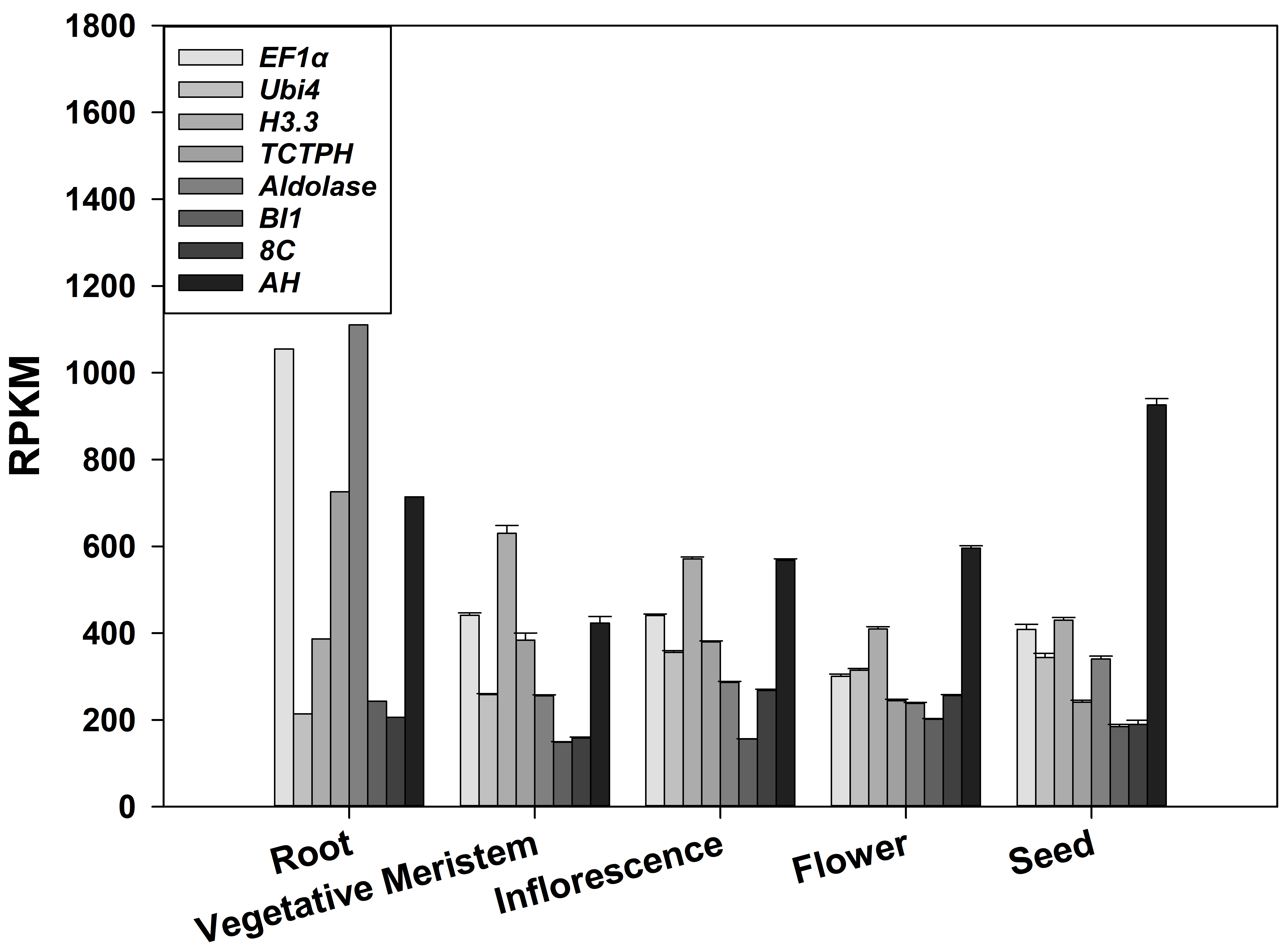
**

**Figure S2.** **Comparison of the deduced amino acid sequence of the PacBio sequence of *EF1α* and its homologs in the *T. ravennae* genome.**

The deduced amino acid sequence of the PacBio sequence of *EF1α* was used as the query sequence to BlastX against the protein sequence data set of *T. ravennae*^29^. The returned homologous sequences were used for protein sequence alignment with the PacBio sequence of *EF1α* using ClustalX 2.0.

**
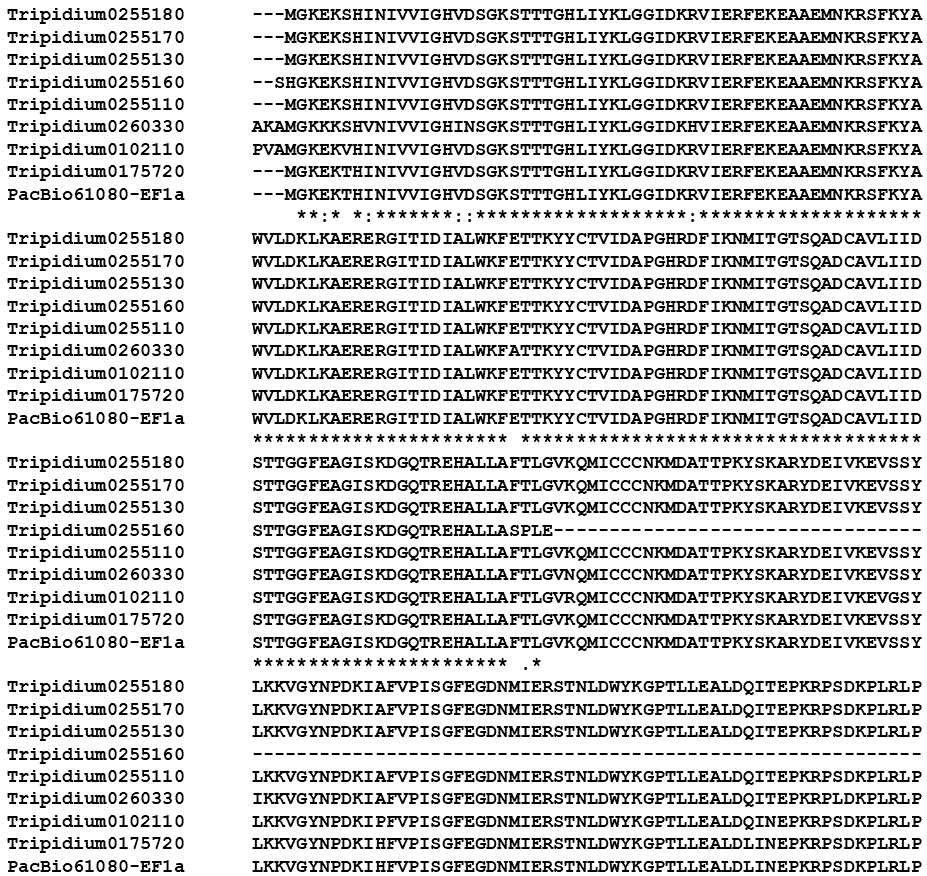
**

**Figure S2. (Cont.)**

**
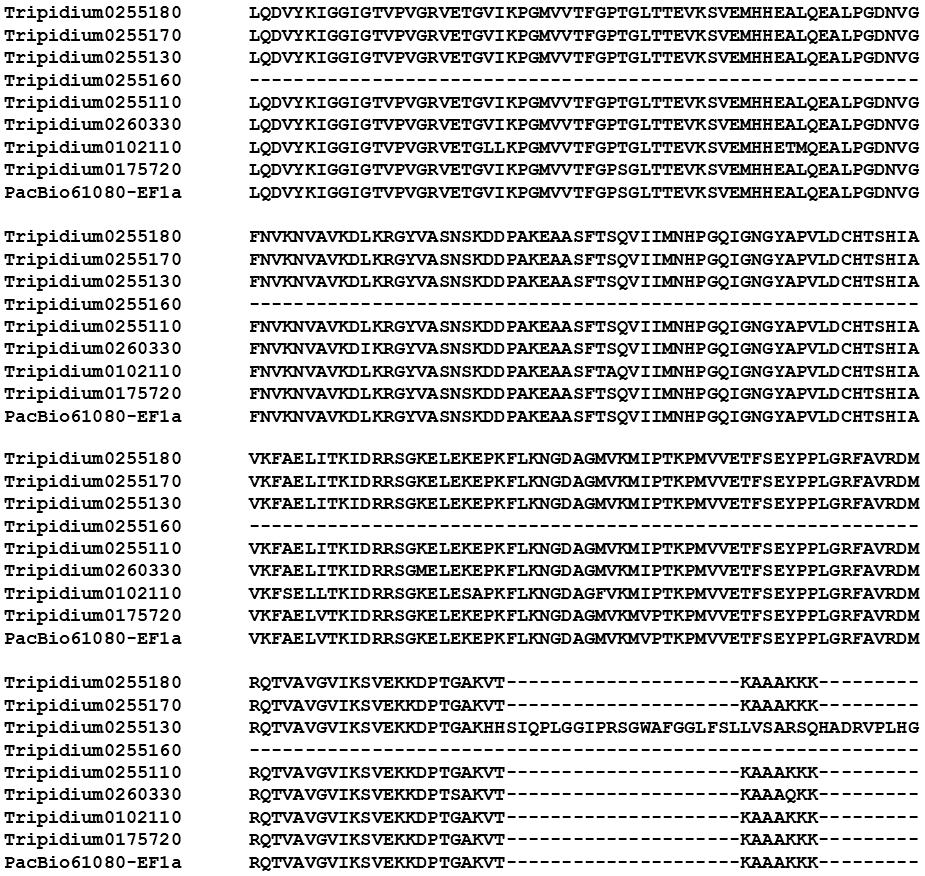
**

**Figure S3.** **Comparison of the deduced amino acid sequence of the PacBio sequence of *Ubi4* and its homologs in the *T. ravennae* genome.**

The deduced amino acid sequence of the PacBio sequence of *Ubi4* was used as the query sequence to BlastX against the protein sequence data set of *T. ravennae*^29^. The returned homologous sequences were used for protein sequence alignment with the PacBio sequence of *Ubi4* using ClustalX 2.0.

**
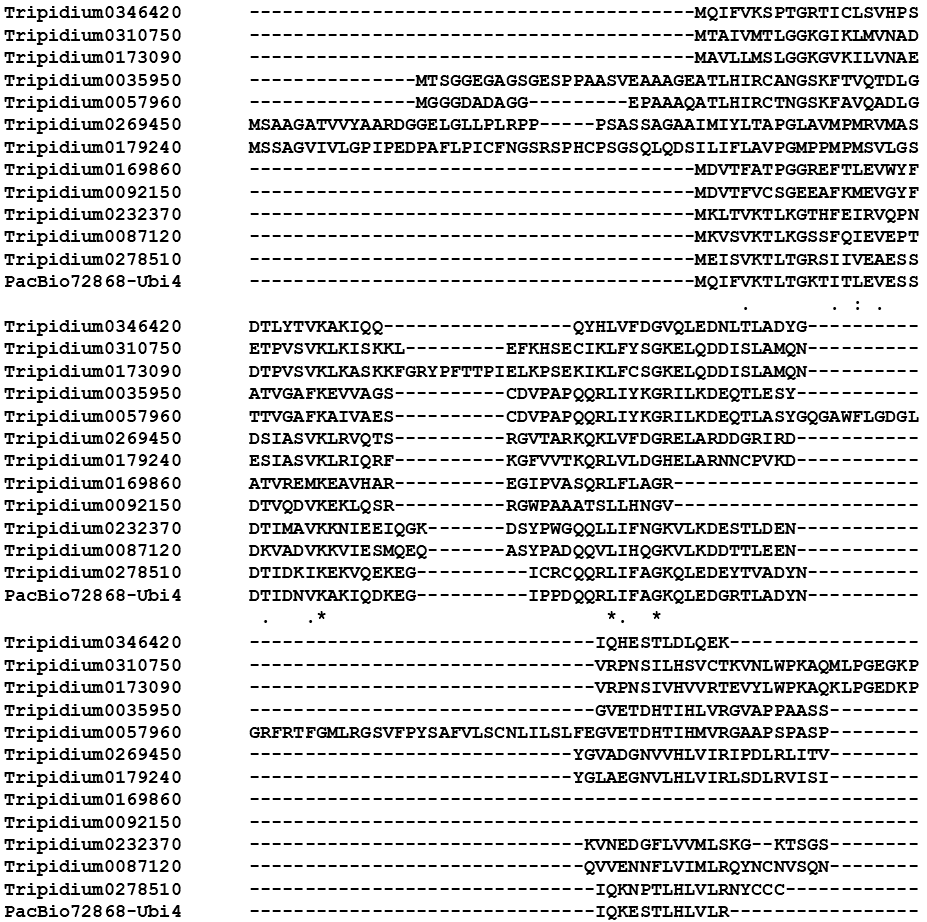
**

**Figure S3. (Cont.)**

**
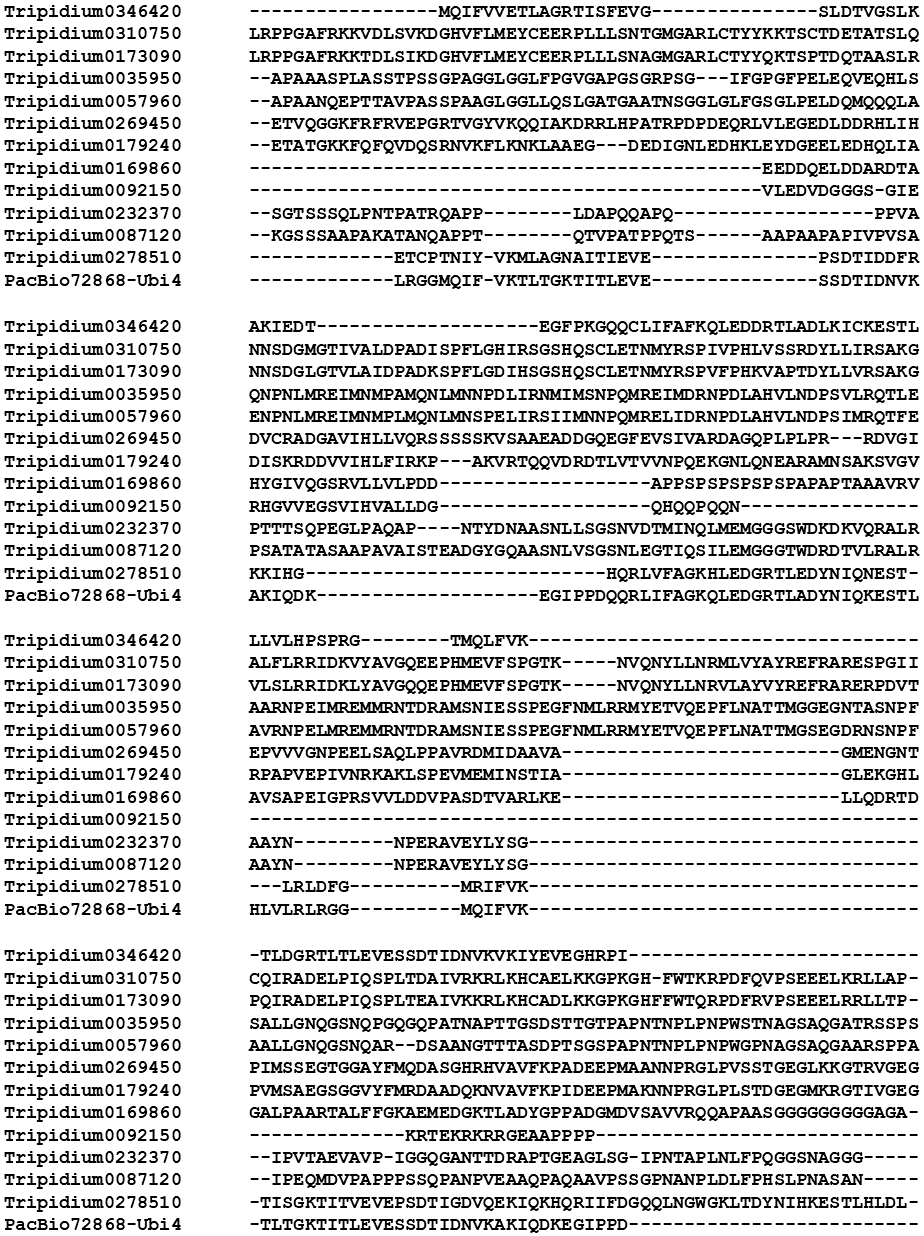
**

**Figure S3. (Cont.)**

**
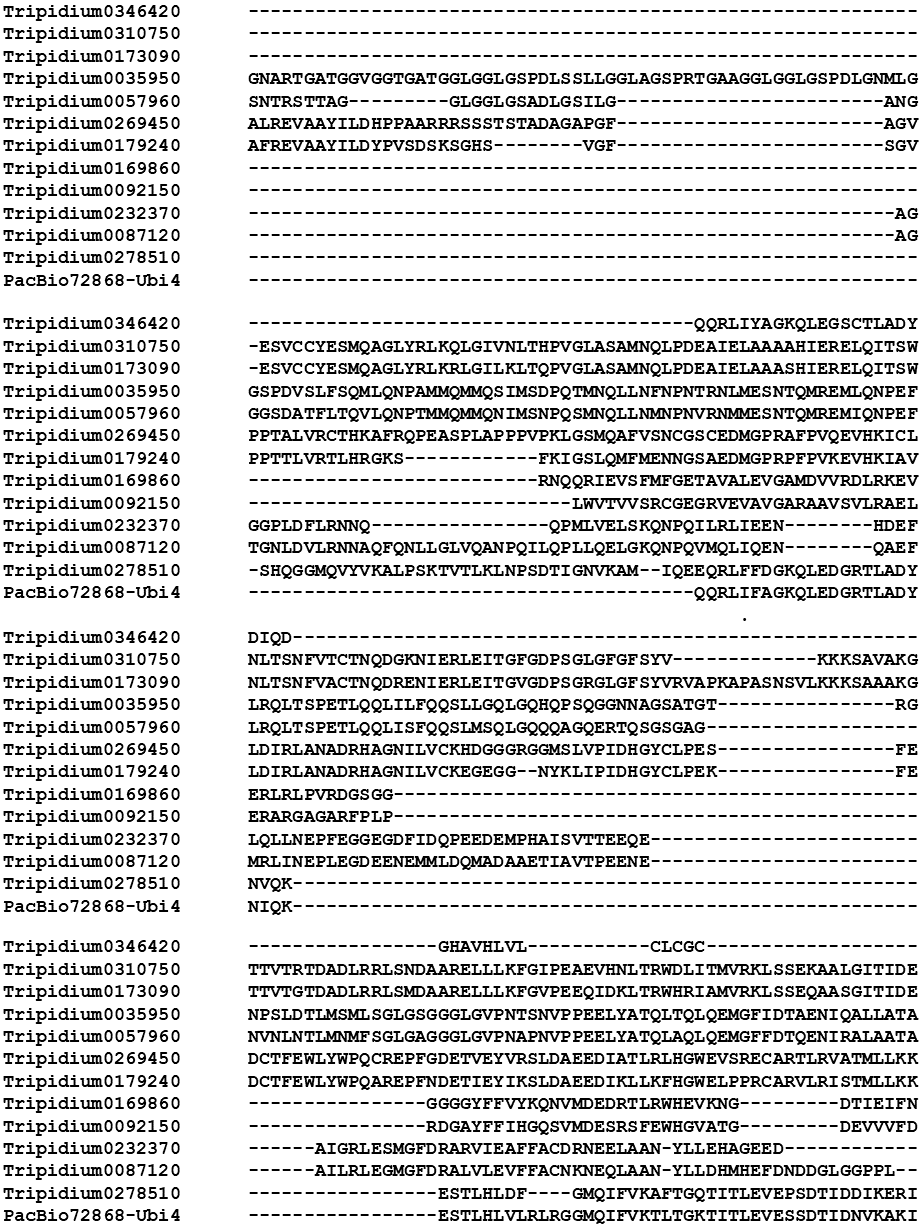
**

**Figure S3. (Cont.)**

**
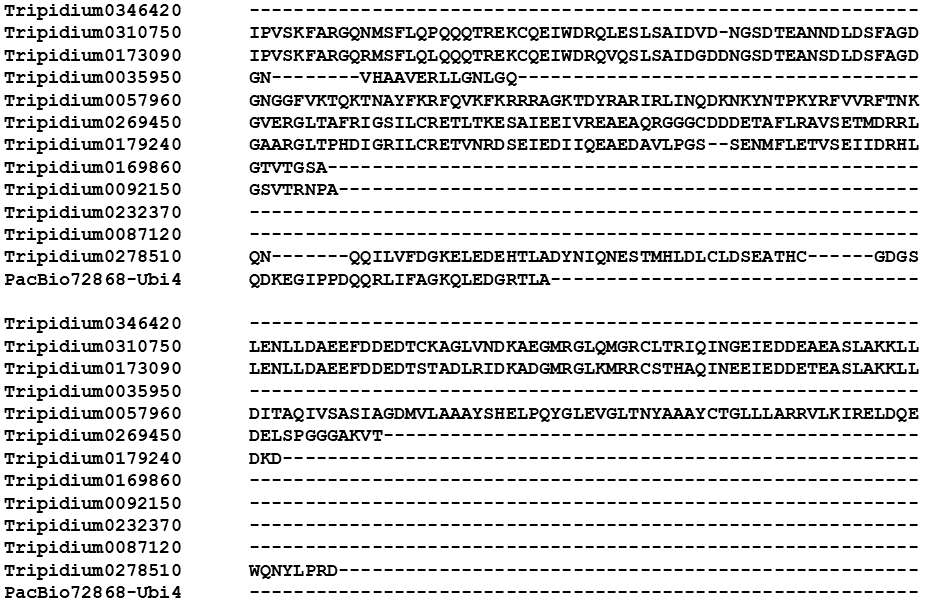
**

**Figure S4.** **Comparison of the deduced amino acid sequence of the PacBio sequence of *H3.3* and its homologs in the *T. ravennae* genome.**

The deduced amino acid sequence of the PacBio sequence of *H3.3* was used as the query sequence to BlastX against the protein sequence data set of *T. ravennae*^29^. The returned homologous sequences were used for protein sequence alignment with the PacBio sequence of *H3.3* using ClustalX 2.0.


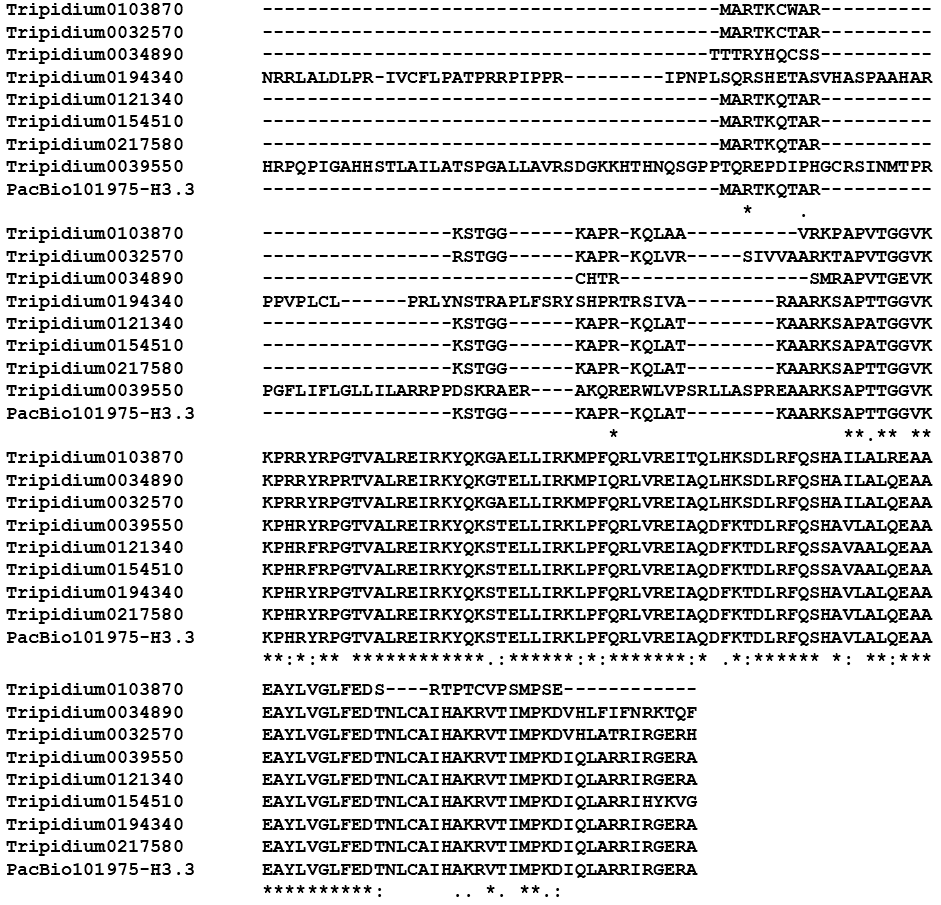


**Figure S5.** **Comparison of the deduced amino acid sequence of the PacBio sequence of *TCTPH* and its homologs in the *T. ravennae* genome.**

The deduced amino acid sequence of the PacBio sequence of *TCTPH* was used as the query sequence to BlastX against the protein sequence data set of *T. ravennae*^29^. The returned homologous sequences were used for protein sequence alignment with the PacBio sequence of *TCTPH* using ClustalX 2.0.

**
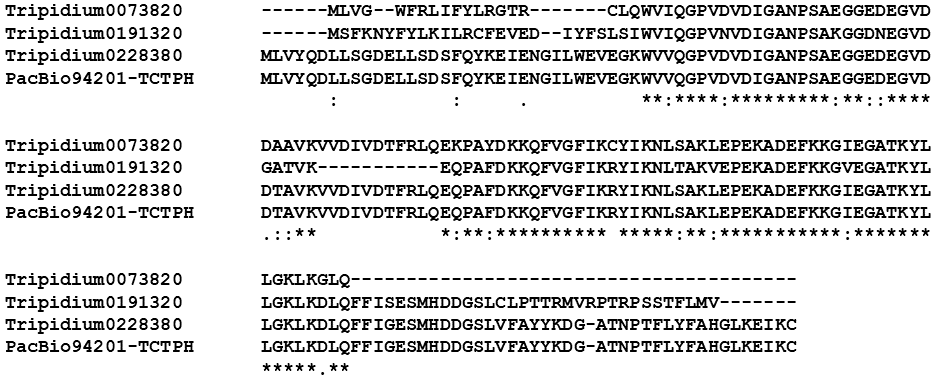
**

**Figure S6.** **Comparison of the deduced amino acid sequence of the PacBio sequence of *Aldolase* and its homologs in the *T. ravennae* genome.**

The deduced amino acid sequence of the PacBio sequence of *Aldolase* was used as the query sequence to BlastX against the protein sequence data set of *T. ravennae*^29^. The returned homologous sequences were used for protein sequence alignment with the PacBio sequence of *Aldolase* using ClustalX 2.0.

**
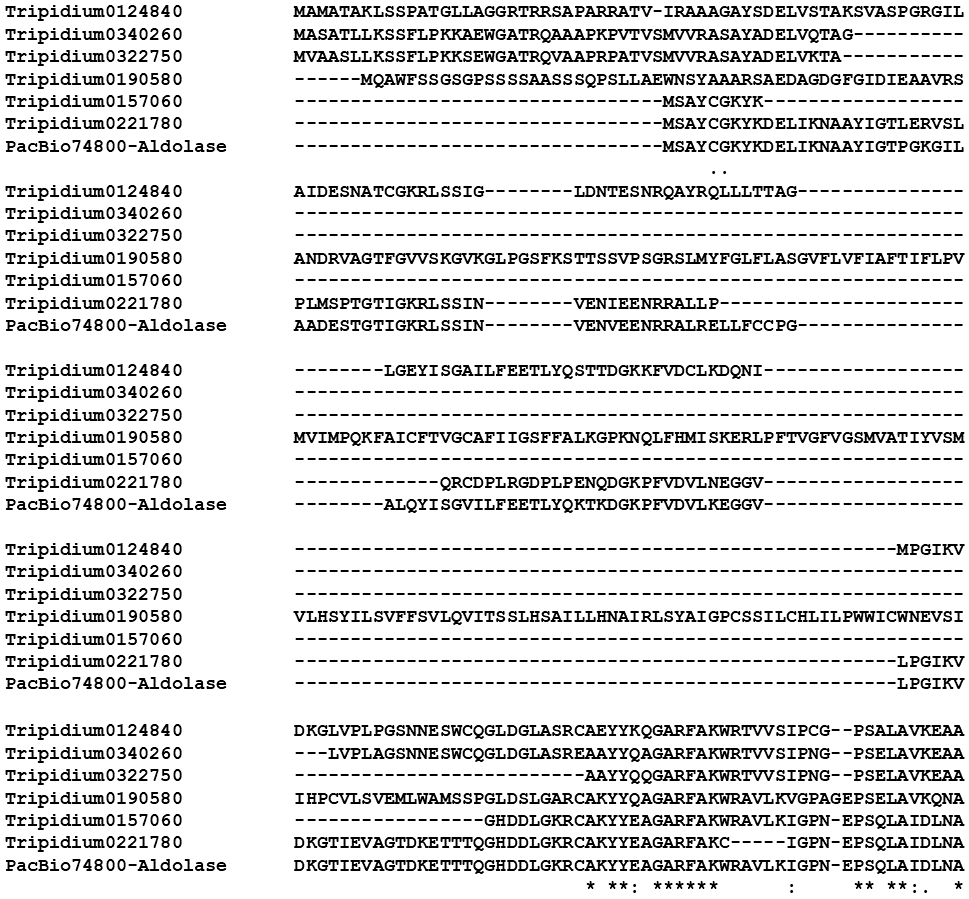
**

**Figure S6. (Cont.)**

**
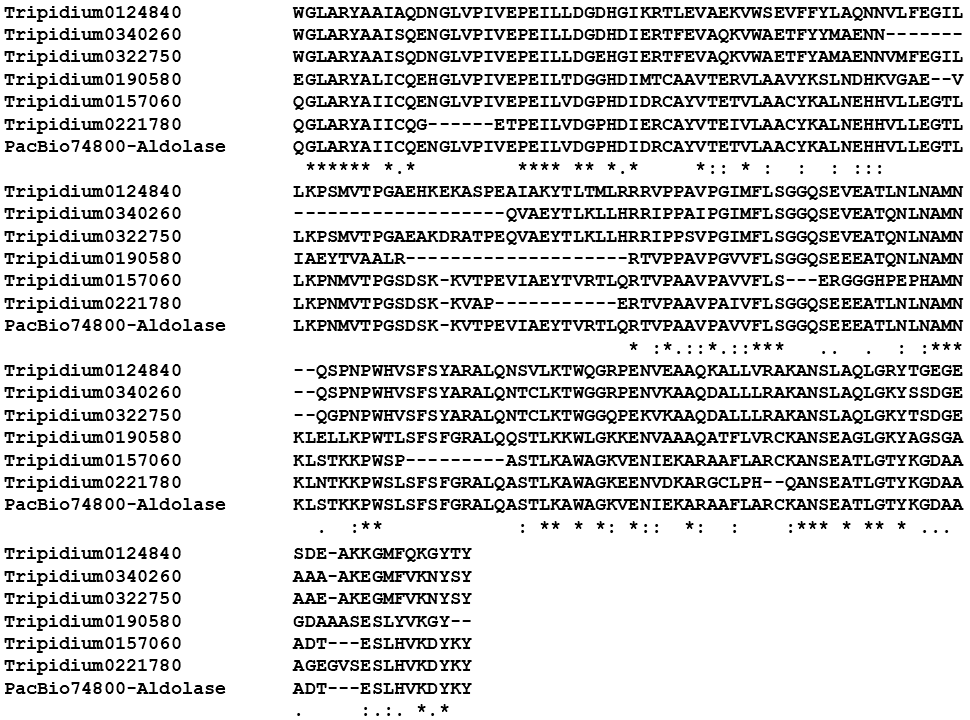
**

**Figure S7.** **Comparison of the deduced amino acid sequence of the PacBio sequence of *BI1* and its homologs in the *T. ravennae* genome.**

The deduced amino acid sequence of the PacBio sequence of *BI1* was used as the query sequence to BlastX against the protein sequence data set of *T. ravennae*^29^. The returned homologous sequences were used for protein sequence alignment with the PacBio sequence of *BI1* using ClustalX 2.0.

**
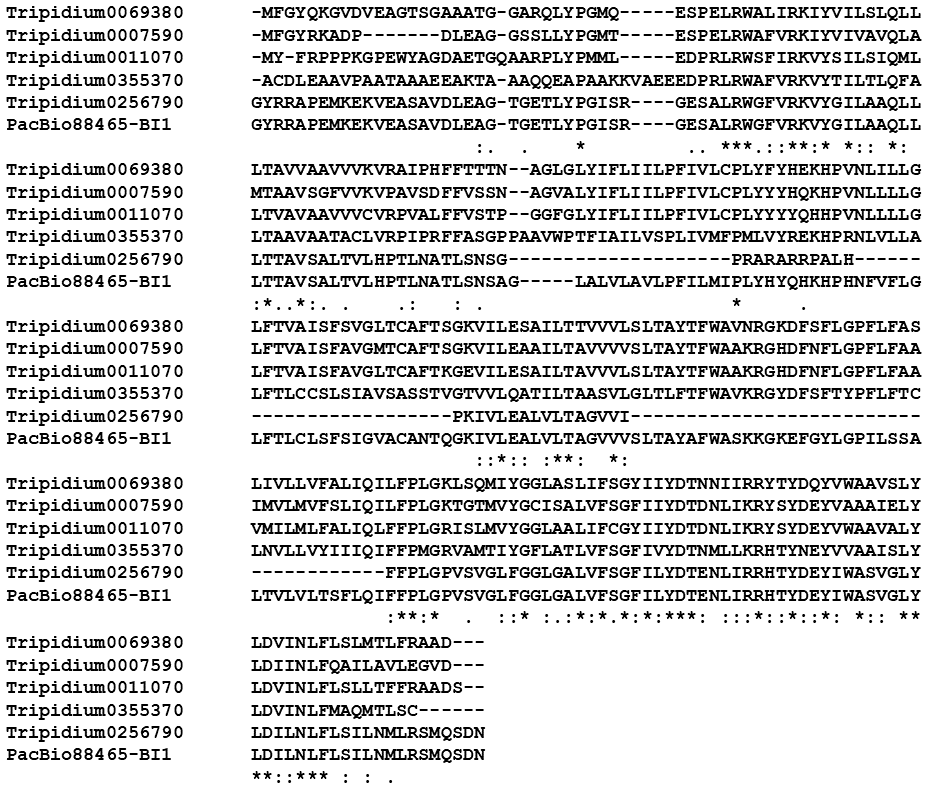
**

**Figure S8.** **Comparison of the deduced amino acid sequence of the PacBio sequence of *8C* and its homologs in the *T. ravennae* genome.**

The deduced amino acid sequence of the PacBio sequence of *8C* was used as the query sequence to BlastX against the protein sequence data set of *T. ravennae*^29^. The returned homologous sequences were used for protein sequence alignment with the PacBio sequence of *8C* using ClustalX 2.0.

**
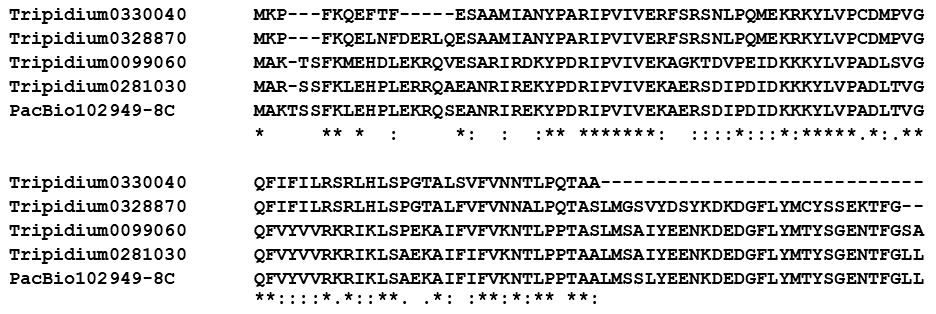
**

**Figure S9.** **Comparison of the deduced amino acid sequence of the PacBio sequence of *AH* and its homologs in the *T. ravennae* genome.**

The deduced amino acid sequence of the PacBio sequence of *AH* was used as the query sequence to BlastX against the protein sequence data set of *T. ravennae*^29^. The returned homologous sequences were used for protein sequence alignment with the PacBio sequence of *AH* using ClustalX 2.0.

**
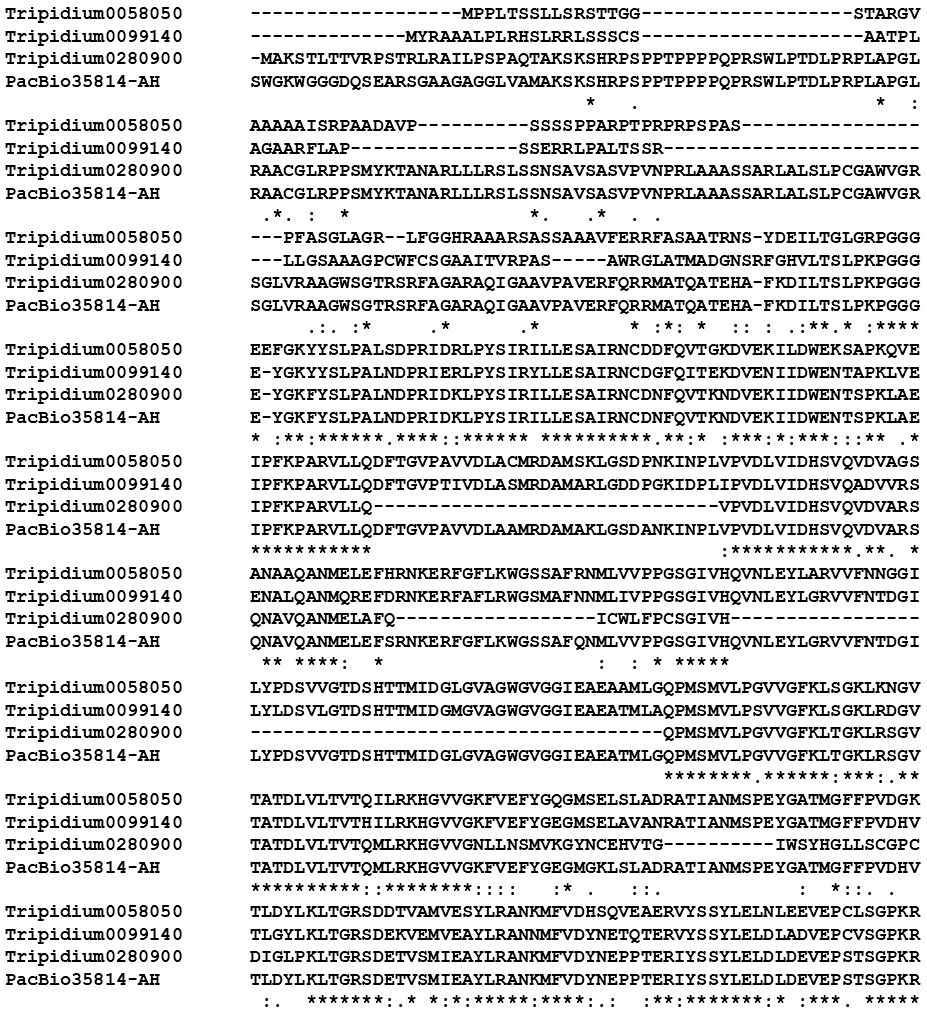
**

**Figure S9. (Cont.)**

**
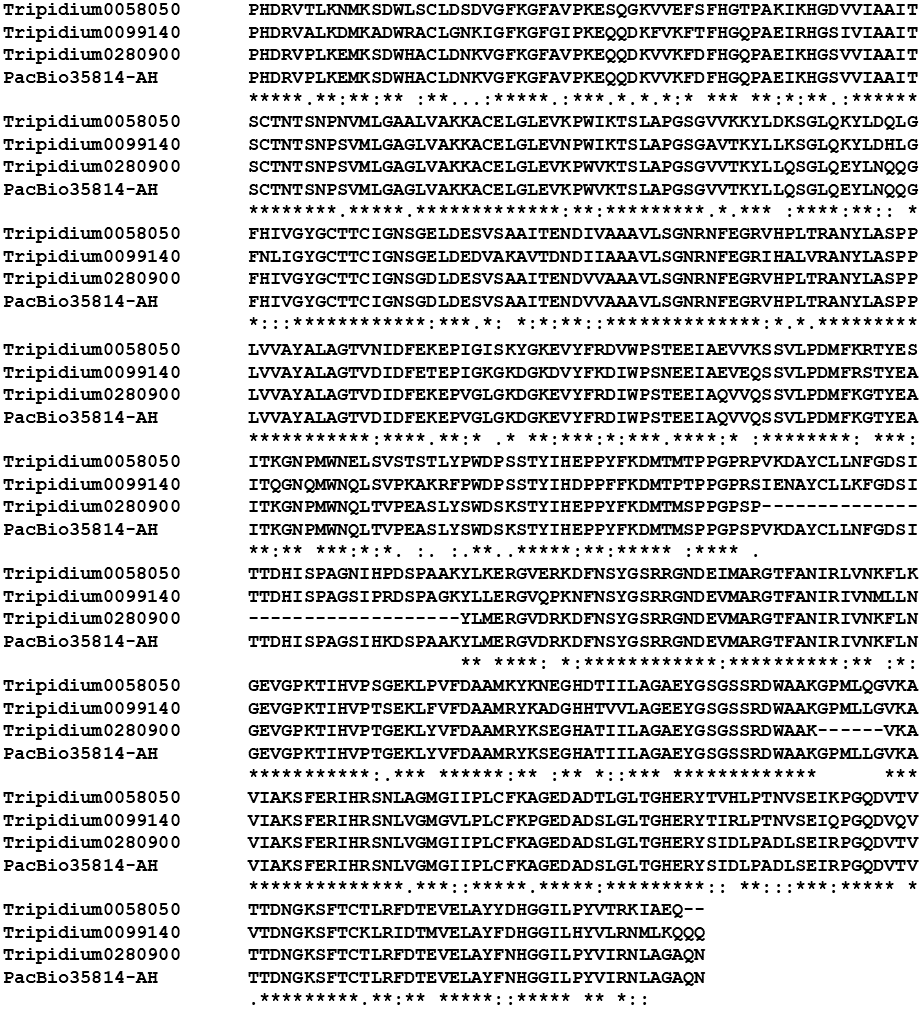
**

**Figure S10.** **Comparison of the cDNA sequence of the PacBio sequence of *EF1α* and its homologs in the *T. ravennae* genome.** The cDNA sequence of the PacBio sequence of *EF1α* was used as the query sequence to BlastN against the cDNA sequence data set of *T. ravennae*^30^. The returned homologous sequences were used for cDNA sequence alignment with the PacBio sequence of *EF1α* using ClustalX 2.0. Red font color, the identical nucleotides between the PacBio sequence of and its most similar homolog but different from the other homologs; Green font color, the SNPs between the PacBio sequence of and its most similar homolog.

**
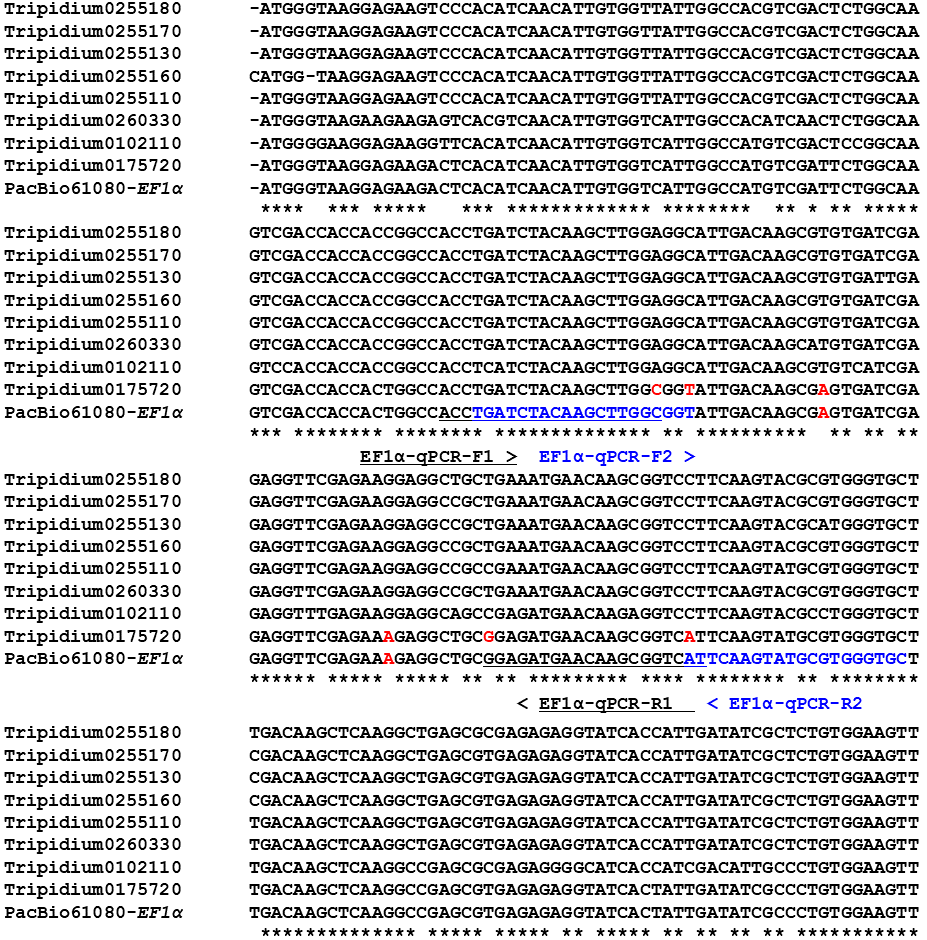
**

**Figure S10. (Cont.)**

**
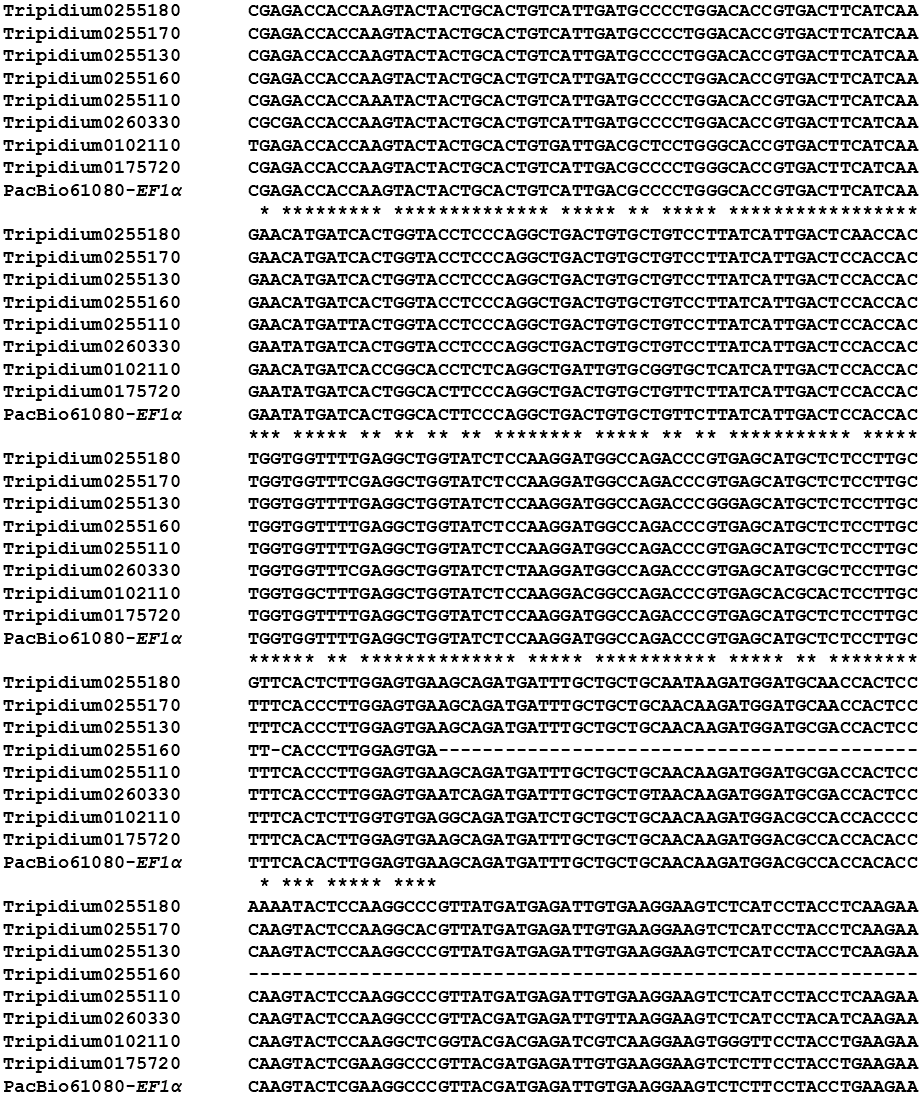
**

**Figure S10. (Cont.)**

**
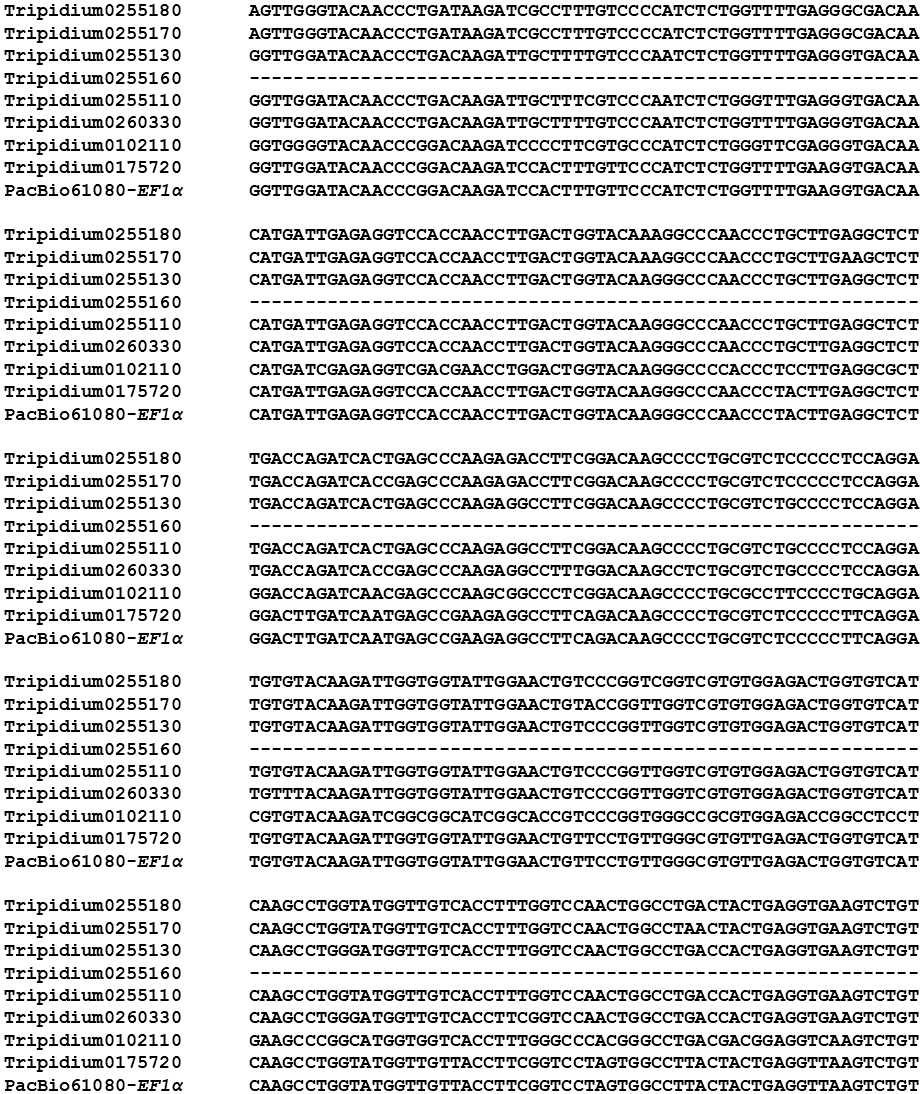
**

**Figure S10. (Cont.)**

**
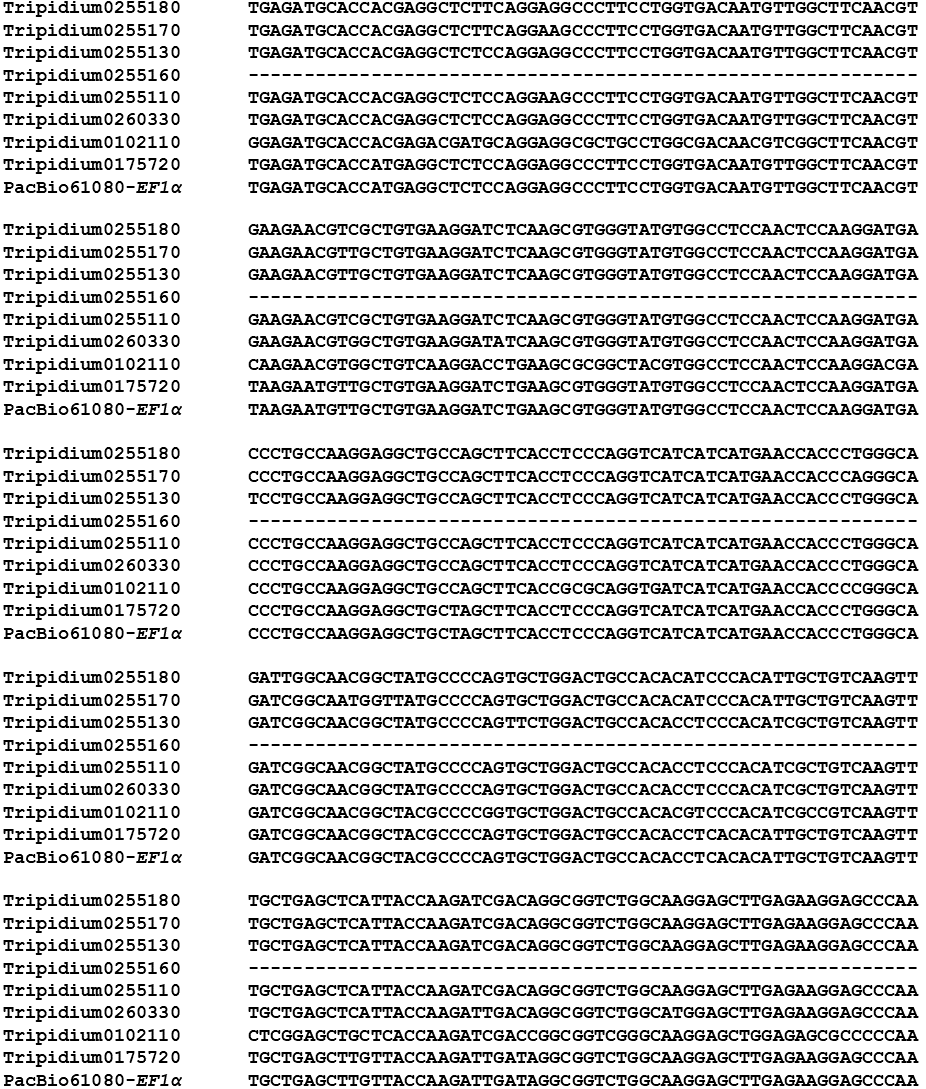
**

**Figure S10. (Cont.)**

**
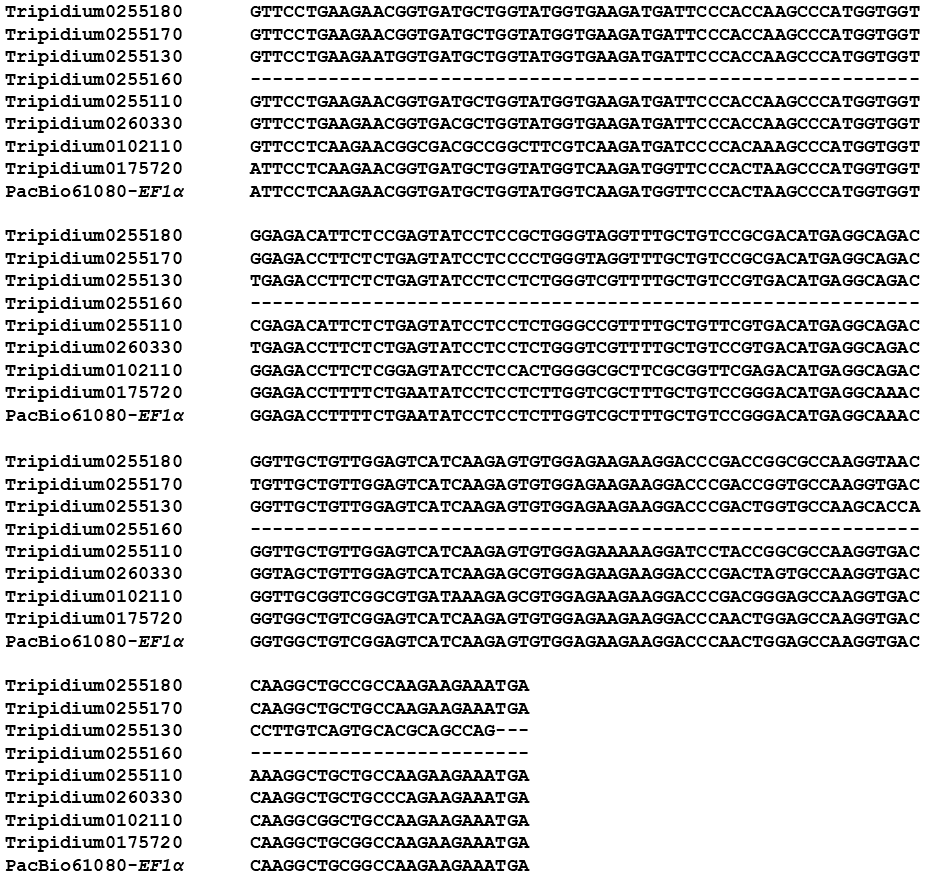
**

**Figure S11.** **Comparison of the cDNA sequence of the PacBio sequence of *Ubi4*** **and its homologs in the *T. ravennae* genome.** The cDNA sequence of the PacBio sequence of *Ubi4* was used as the query sequence to BlastN against the cDNA sequence data set of *T. ravennae*^30^. The returned homologous sequences were used for cDNA sequence alignment with the PacBio sequence of *Ubi4* using ClustalX 2.0. Red font color, the identical nucleotides between the PacBio sequence of and its most similar homolog but different from the other homologs; Green font color, the SNPs between the PacBio sequence of and its most similar homolog.

**
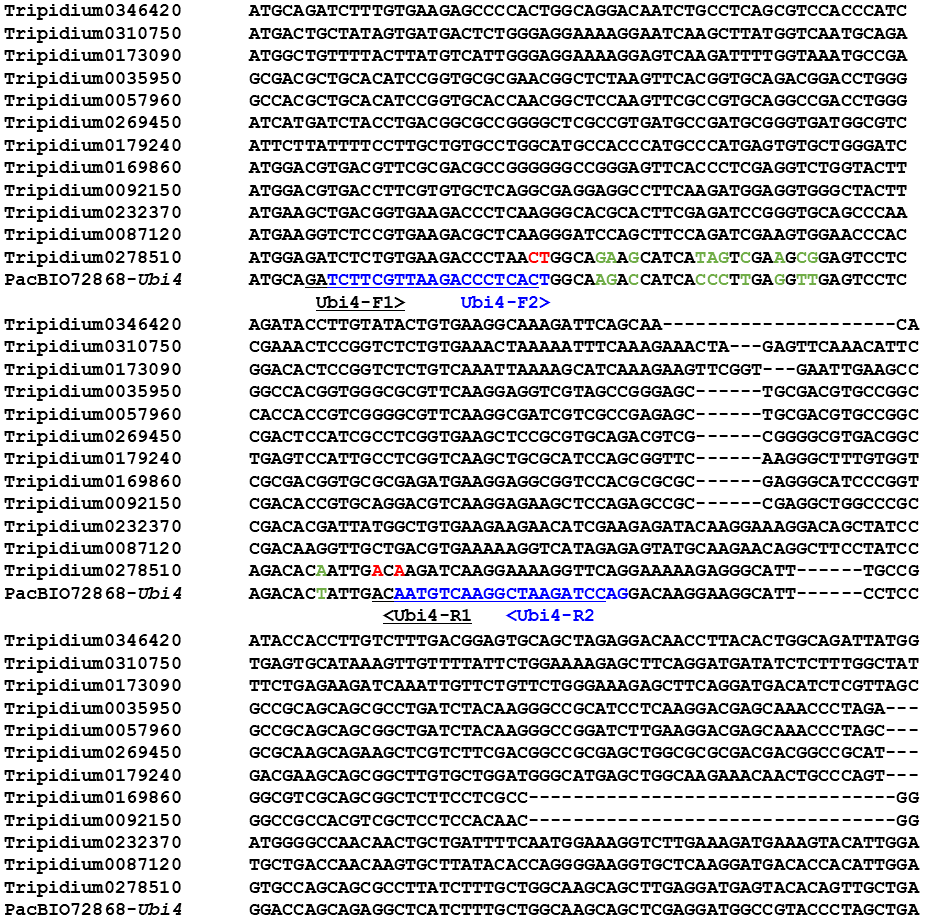
**

**Figure S11. (Cont.)**

**
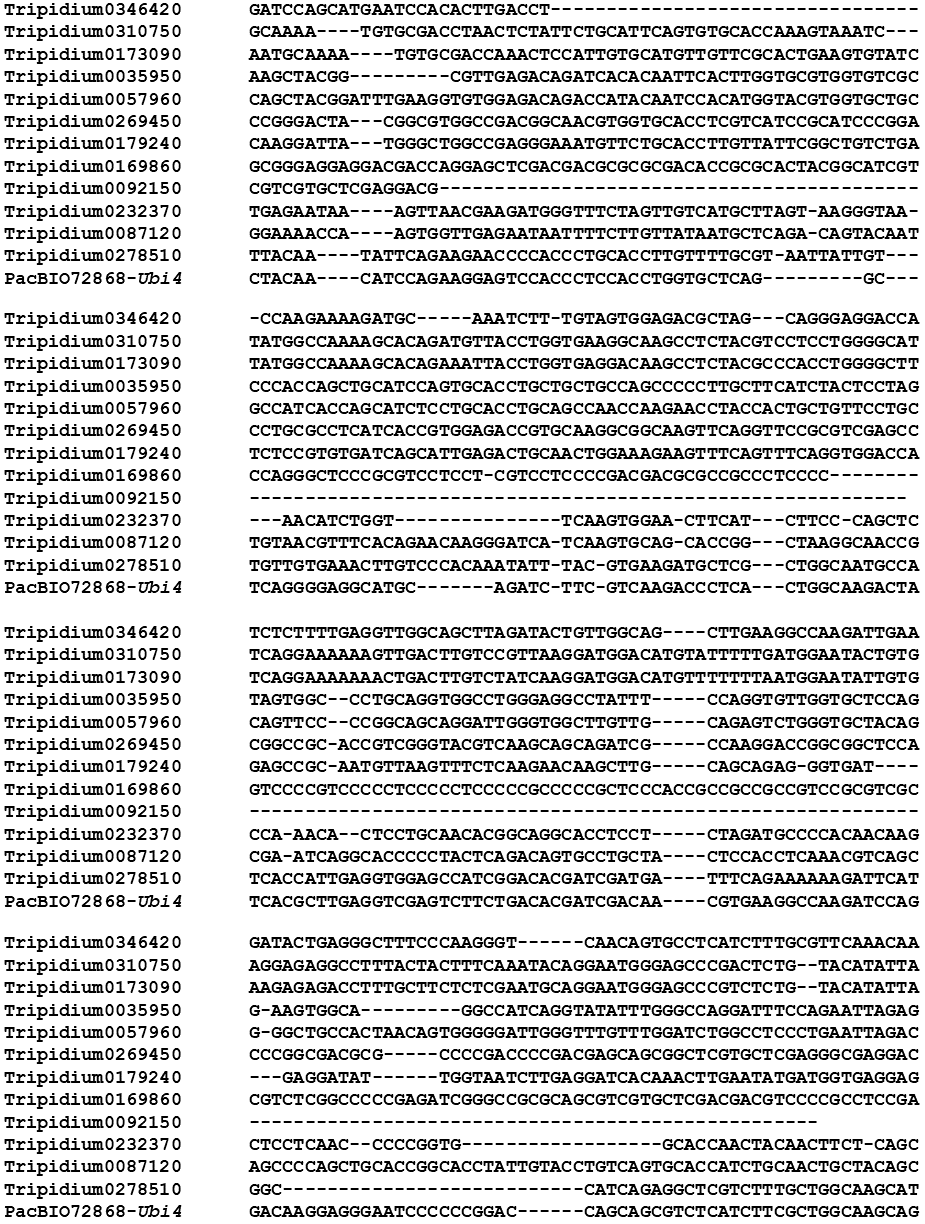
**

**Figure S11. (Cont.)**

**
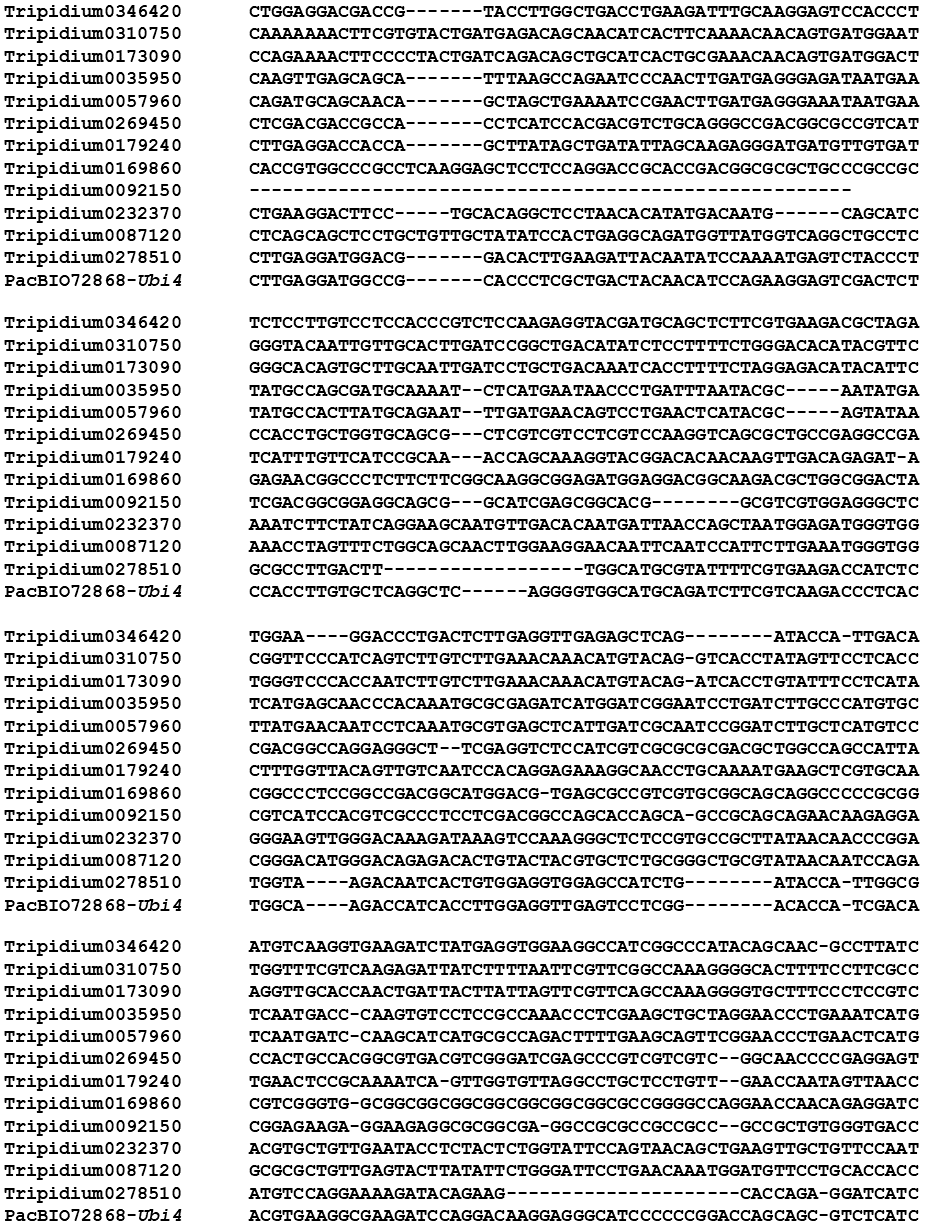
**

**Figure S11. (Cont.)**

**
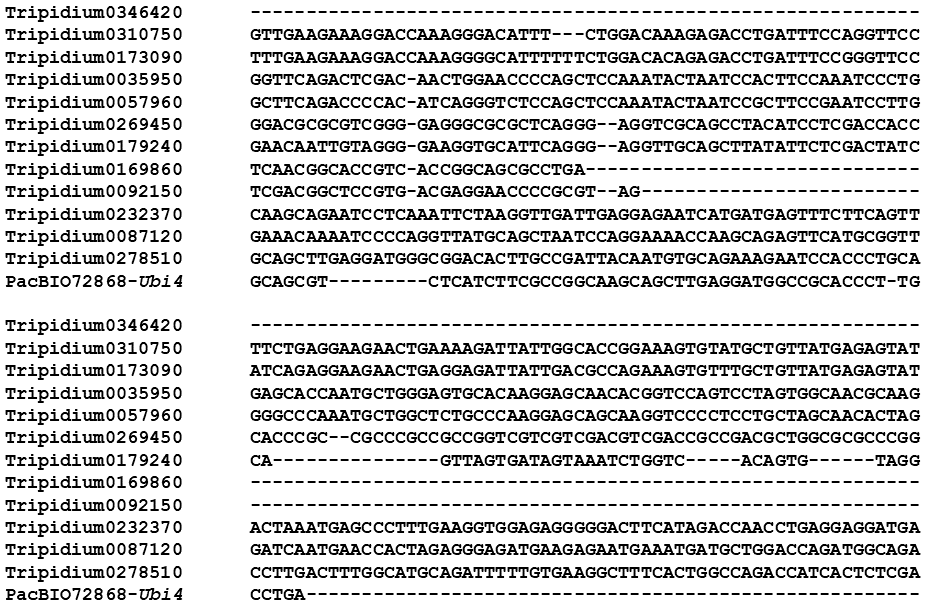
**

**Figure S12.** **Comparison of the cDNA sequence of the PacBio sequence of *H3.3* and its homologs in the *T. ravennae* genome.** The cDNA sequence of the PacBio sequence of *H3.3* was used as the query sequence to BlastN against the cDNA sequence data set of *T. ravennae*^30^. The returned homologous sequences were used for cDNA sequence alignment with the PacBio sequence of *H3.3* using ClustalX 2.0. Red font color, the identical nucleotides between the PacBio sequence of and its most similar homolog but different from the other homologs; Green font color, the SNPs between the PacBio sequence of and its most similar homolog.

**
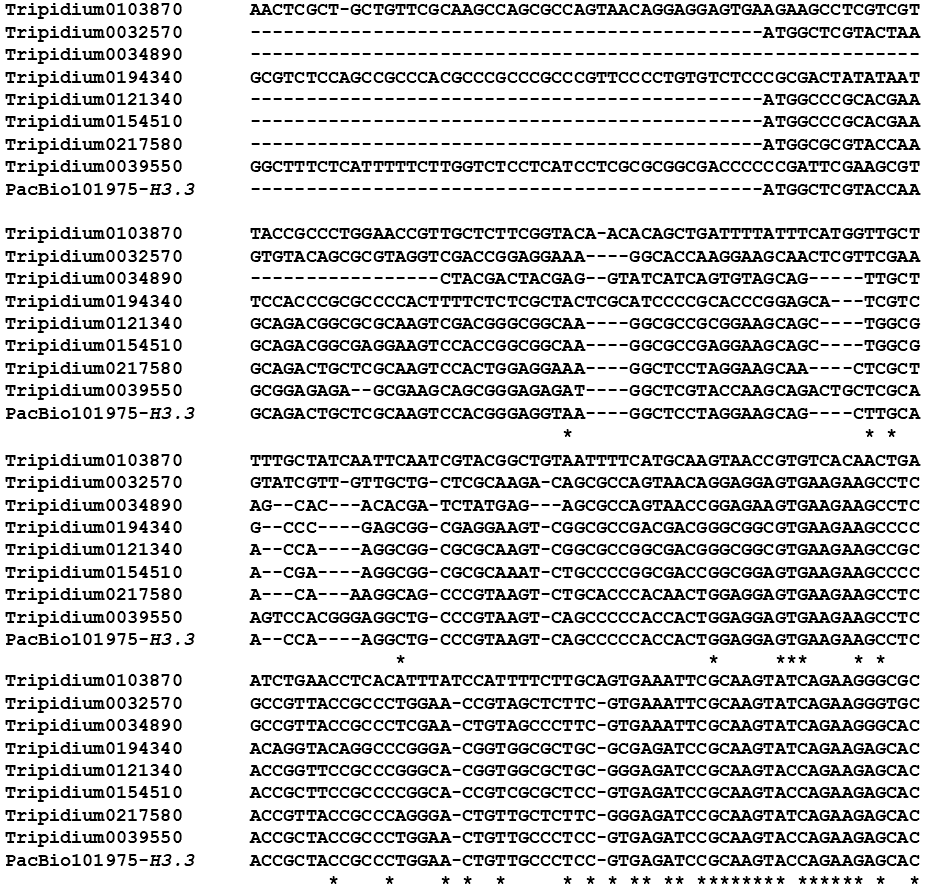
**

**Figure S12. (Cont.)**

**
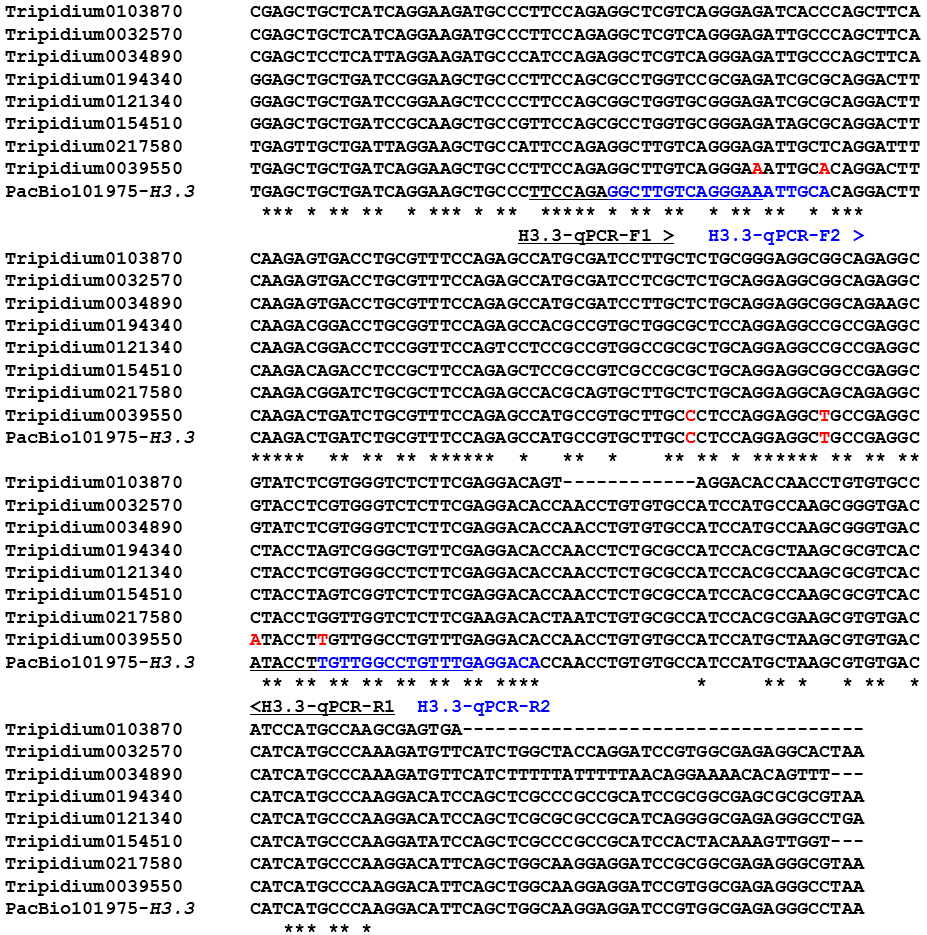
**

**Figure S13.** **Comparison of the cDNA sequence of the PacBio sequence of *TCTPH* and its homologs in the *T. ravennae* genome.** The cDNA sequence of the PacBio sequence of *TCTPH* was used as the query sequence to BlastN against the cDNA sequence data set of *T. ravennae*^30^. The returned homologous sequences were used for cDNA sequence alignment with the PacBio sequence of *TCTPH* using ClustalX 2.0. Red font color, the identical nucleotides between the PacBio sequence of and its most similar homolog but different from the other homologs; Green font color, the SNPs between the PacBio sequence of and its most similar homolog.

**
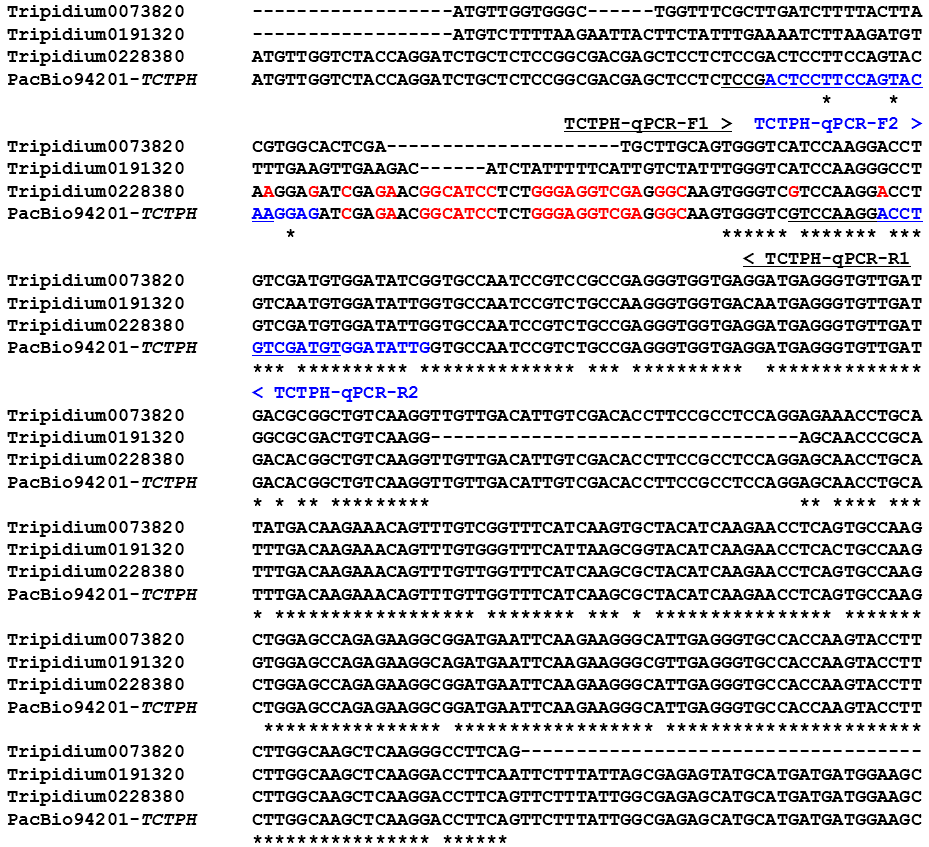
**

**Figure S13. (Cont.)**

**
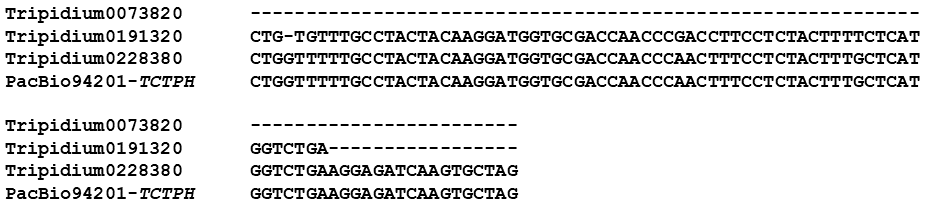
**

**Figure S14.** **Comparison of the cDNA sequence of the PacBio sequence of *Aldolase* and its homologs in the *T. ravennae* genome.** The cDNA sequence of the PacBio sequence of *Aldolase* was used as the query sequence to BlastN against the cDNA sequence data set of *T. ravennae*^30^. The returned homologous sequences were used for cDNA sequence alignment with the PacBio sequence of *Aldolase* using ClustalX 2.0. Red font color, the identical nucleotides between the PacBio sequence of and its most similar homolog but different from the other homologs; Green font color, the SNPs between the PacBio sequence of and its most similar homolog.

**
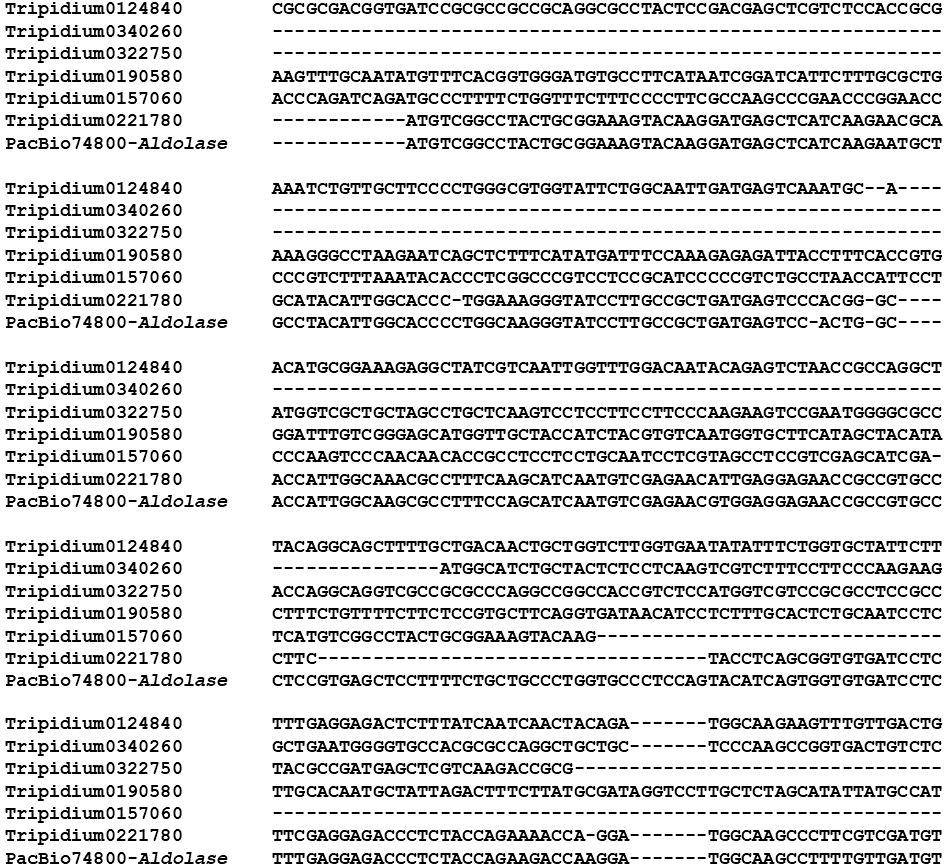
**

**Figure S14. (Cont.)**

**
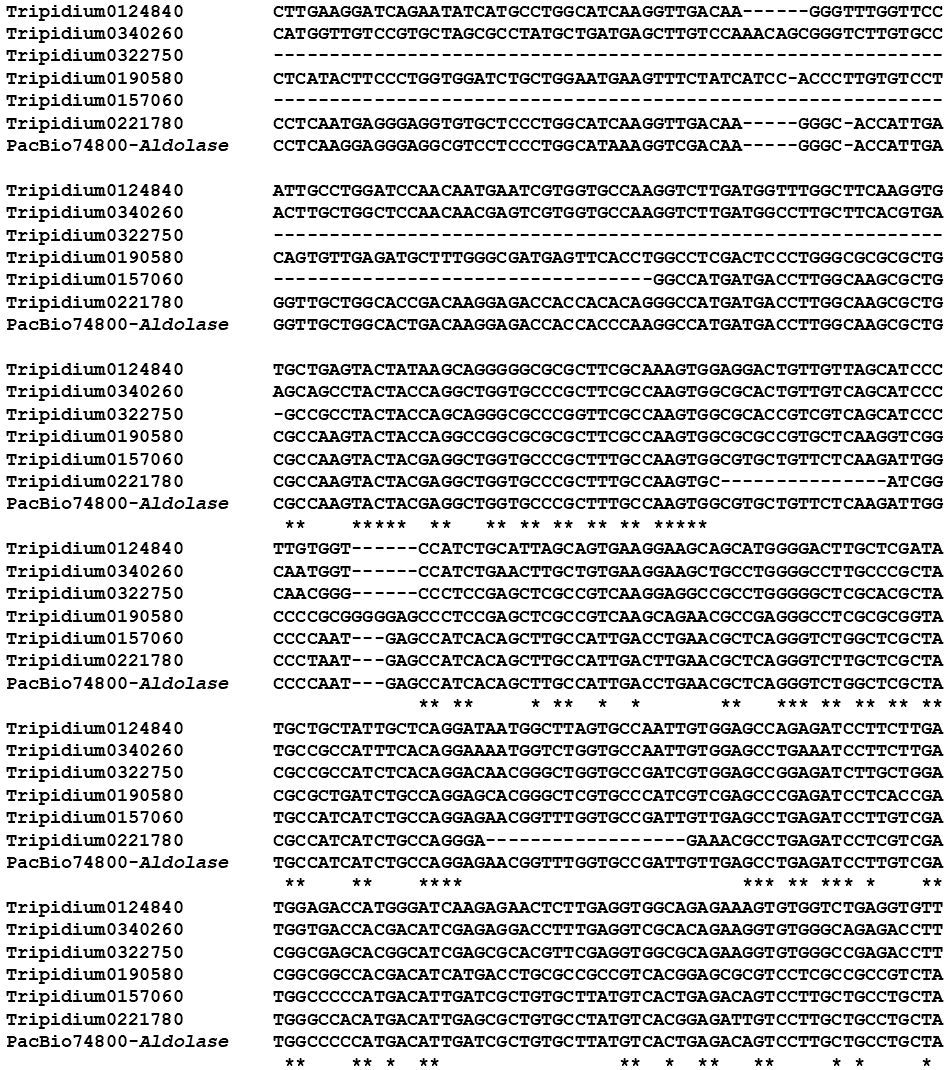
**

**Figure S14. (Cont.)**

**
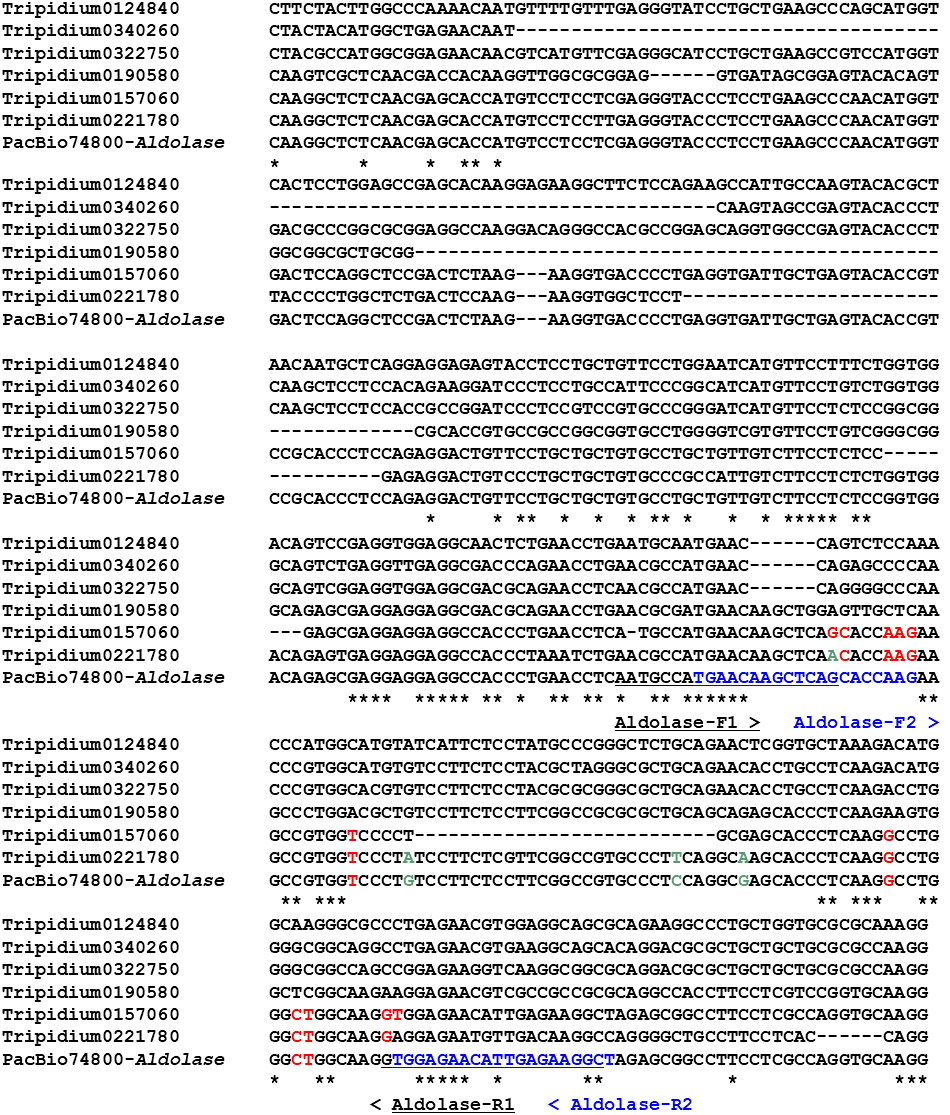
**

**Figure S14. (Cont.)**

**
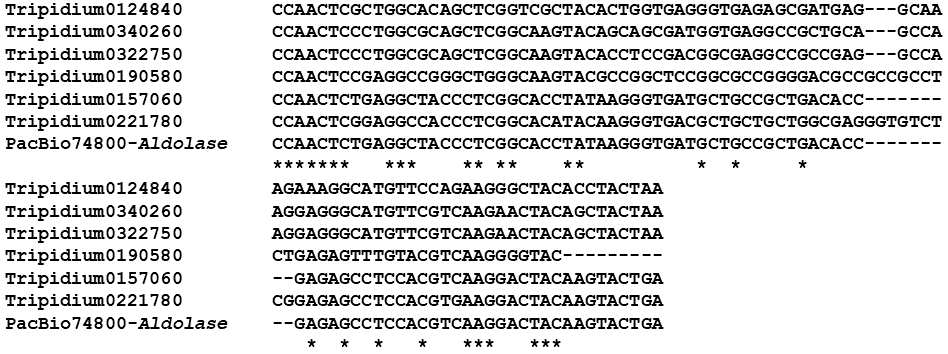
**

**Figure S15.** **Comparison of the cDNA sequence of the PacBio sequence of *BI1* and its homologs in the *T. ravennae* genome.** The cDNA sequence of the PacBio sequence of *BI1* was used as the query sequence to BlastN against the cDNA sequence data set of *T. ravennae*^30^. The returned homologous sequences were used for cDNA sequence alignment with the PacBio sequence of *BI1* using ClustalX 2.0. Red font color, the identical nucleotides between the PacBio sequence of and its most similar homolog but different from the other homologs; Green font color, the SNPs between the PacBio sequence of and its most similar homolog.

**
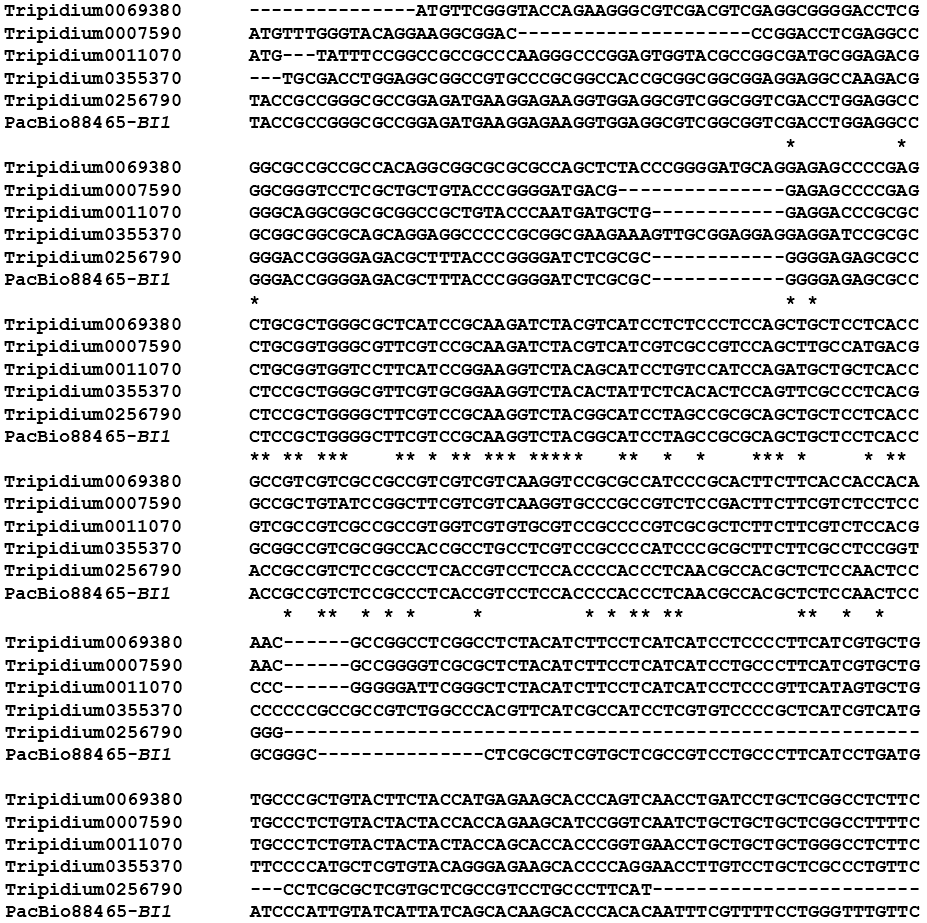
**

**Figure S15. (Cont.)**

**
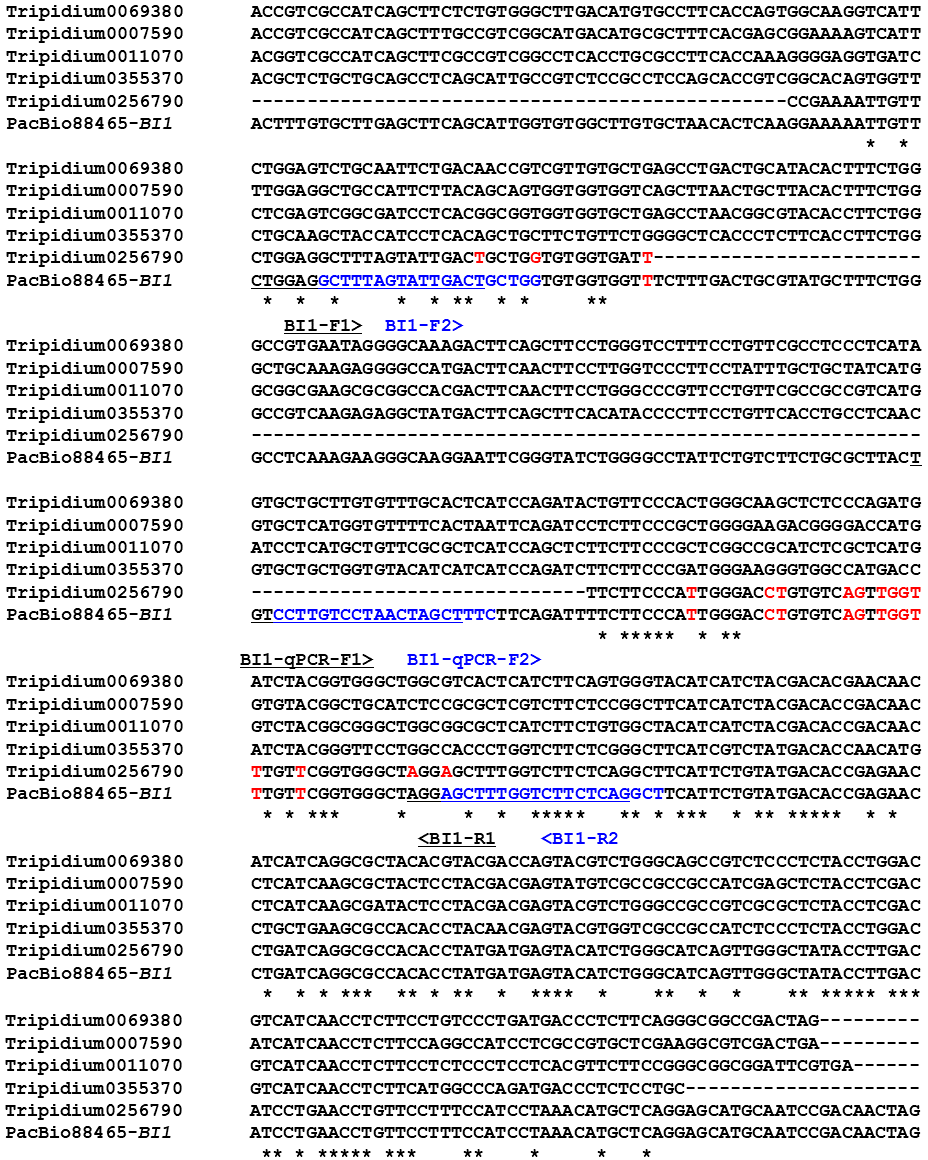
**

**Figure S16.** **Comparison of the cDNA sequence of the PacBio sequence of *8C* and its homologs in the *T. ravennae* genome.** The cDNA sequence of the PacBio sequence of *8C* was used as the query sequence to BlastN against the cDNA sequence data set of *T. ravennae*^30^. The returned homologous sequences were used for cDNA sequence alignment with the PacBio sequence of *8C* using ClustalX 2.0. Red font color, the identical nucleotides between the PacBio sequence of and its most similar homolog but different from the other homologs; Green font color, the SNPs between the PacBio sequence of and its most similar homolog.

**
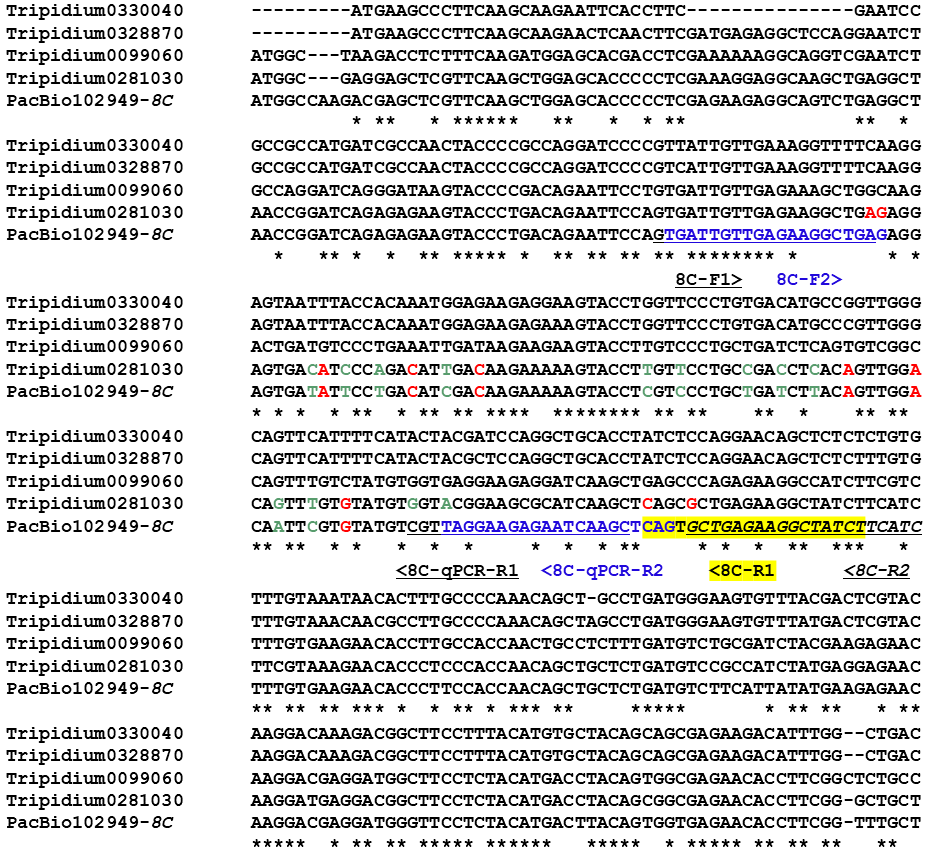
**

**Figure S16. (Cont.)**

**
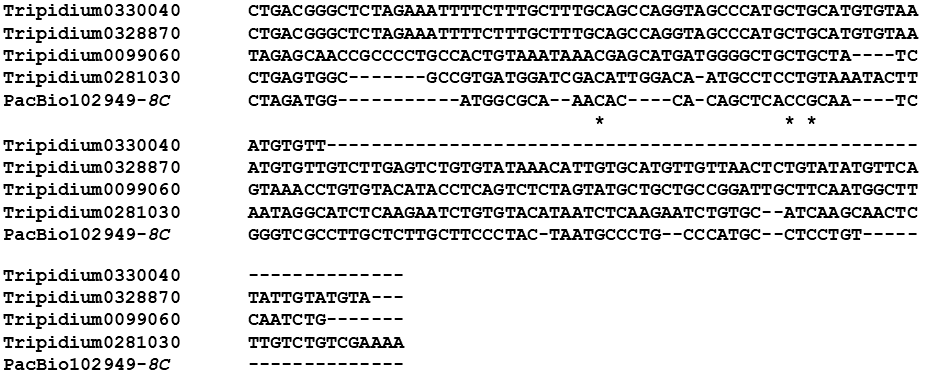
**

**Figure S17.** **Comparison of the cDNA sequence of the PacBio sequence of *AH* and its homologs in the *T. ravennae* genome.** The cDNA sequence of the PacBio sequence of *AH* was used as the query sequence to BlastN against the cDNA sequence data set of *T. ravennae*^30^. The returned homologous sequences were used for cDNA sequence alignment with the PacBio sequence of *AH* using ClustalX 2.0. Red font color, the identical nucleotides between the PacBio sequence of and its most similar homolog but different from the other homologs; Green font color, the SNPs between the PacBio sequence of and its most similar homolog.


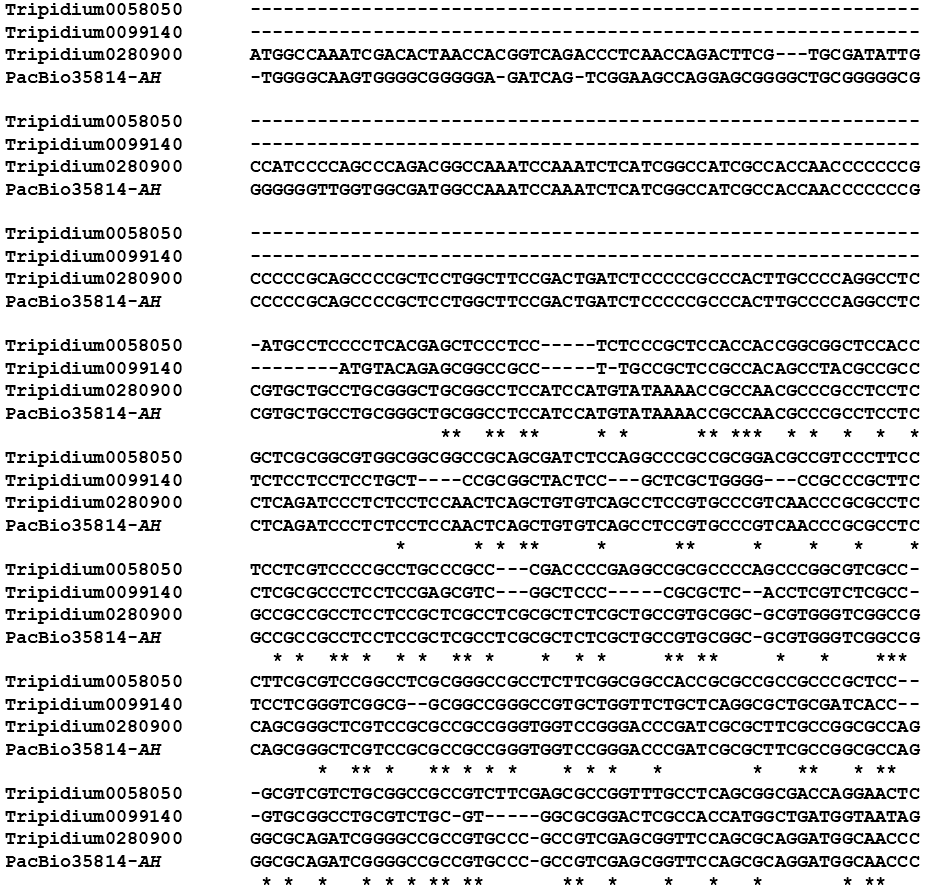


**Figure S17. (Cont.)**

**
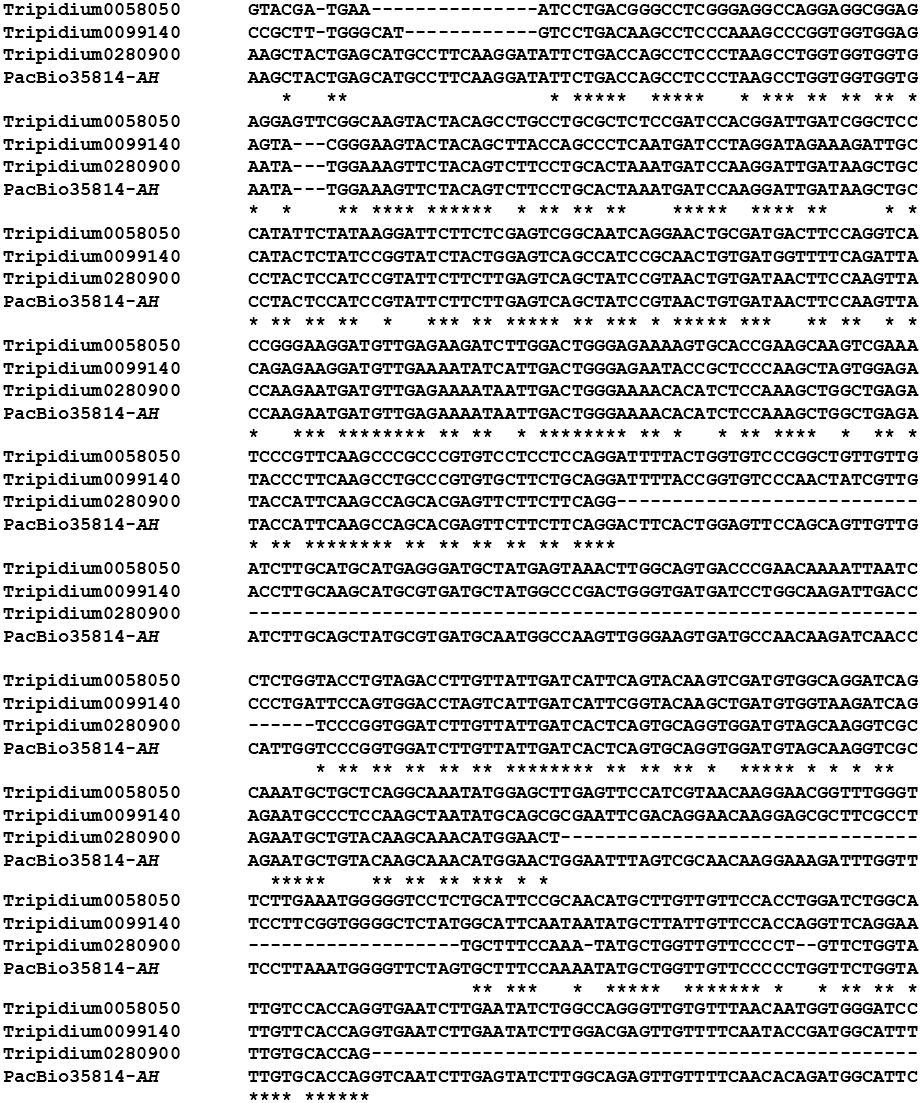
**

**Figure S17. (Cont.)**

**
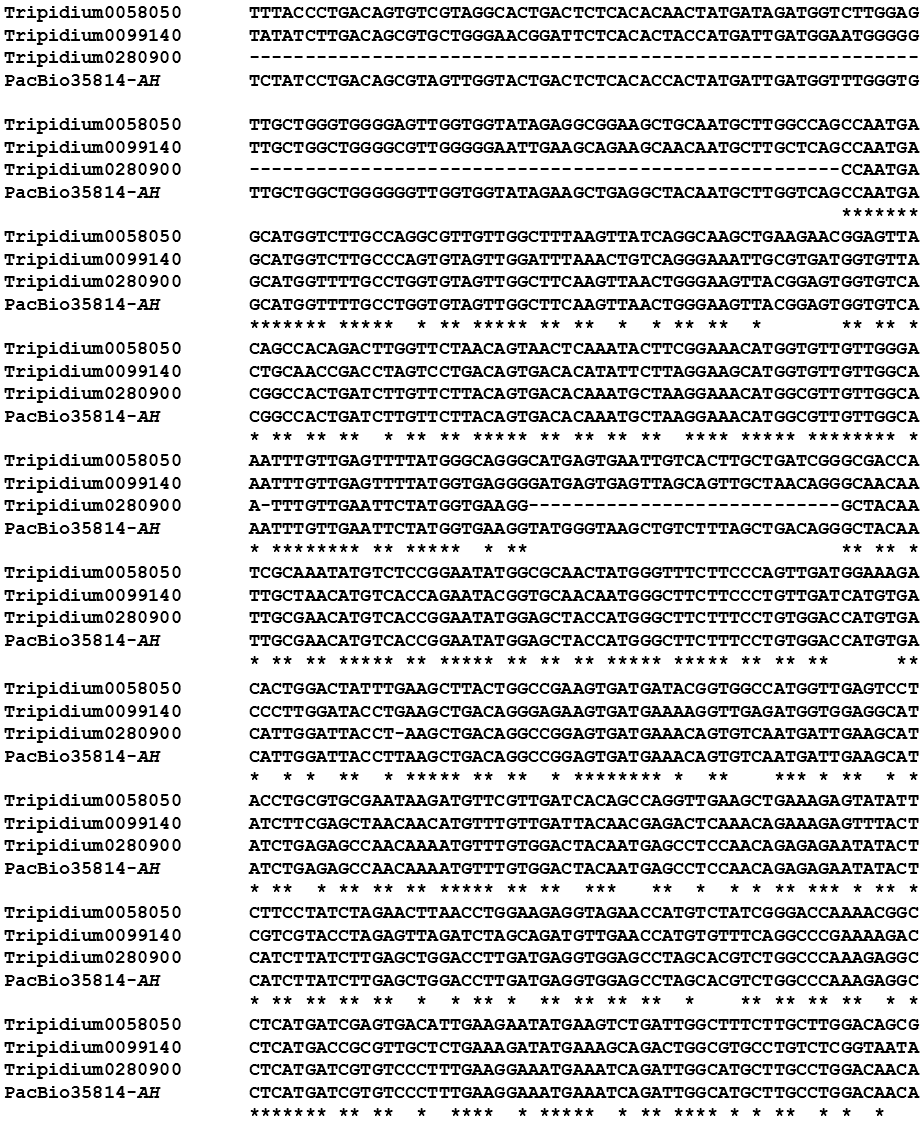
**

**Figure S17. (Cont.)**

**
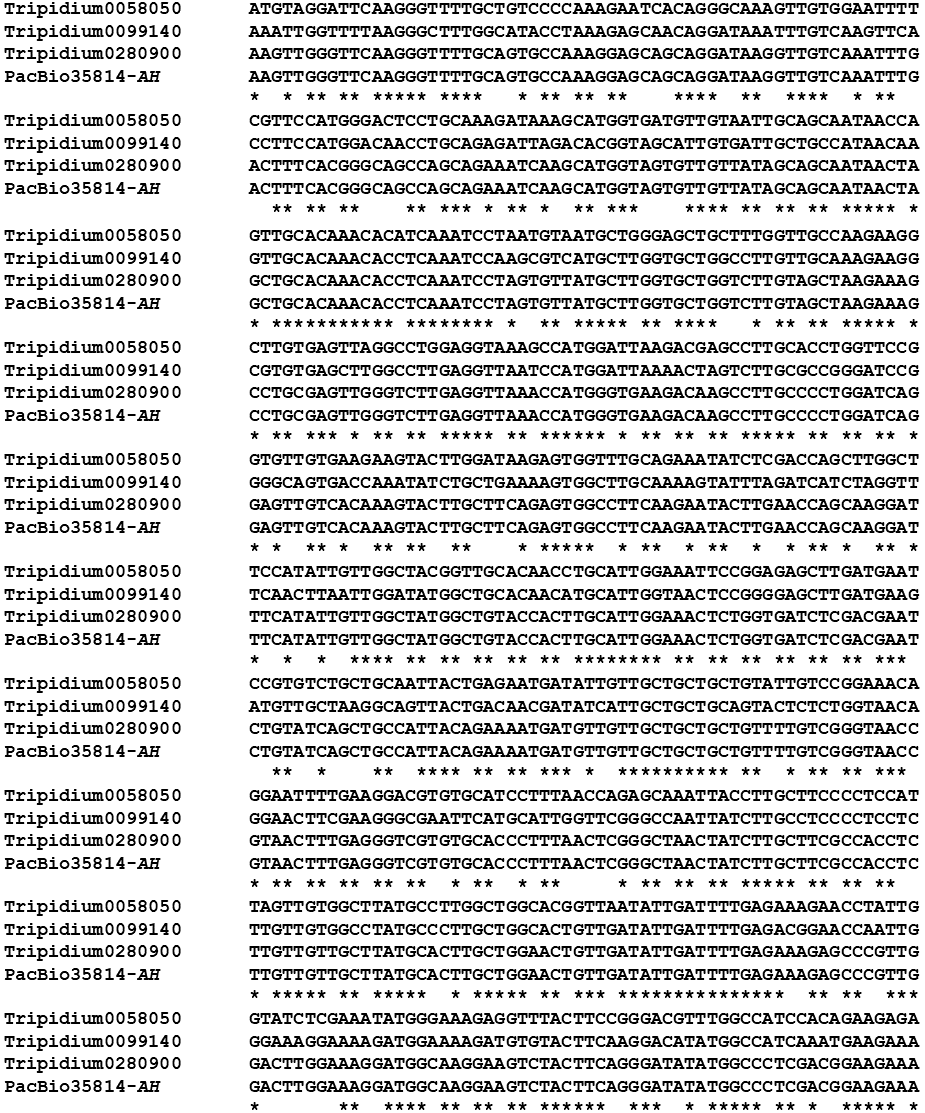
**

**Figure S17. (Cont.)**

**
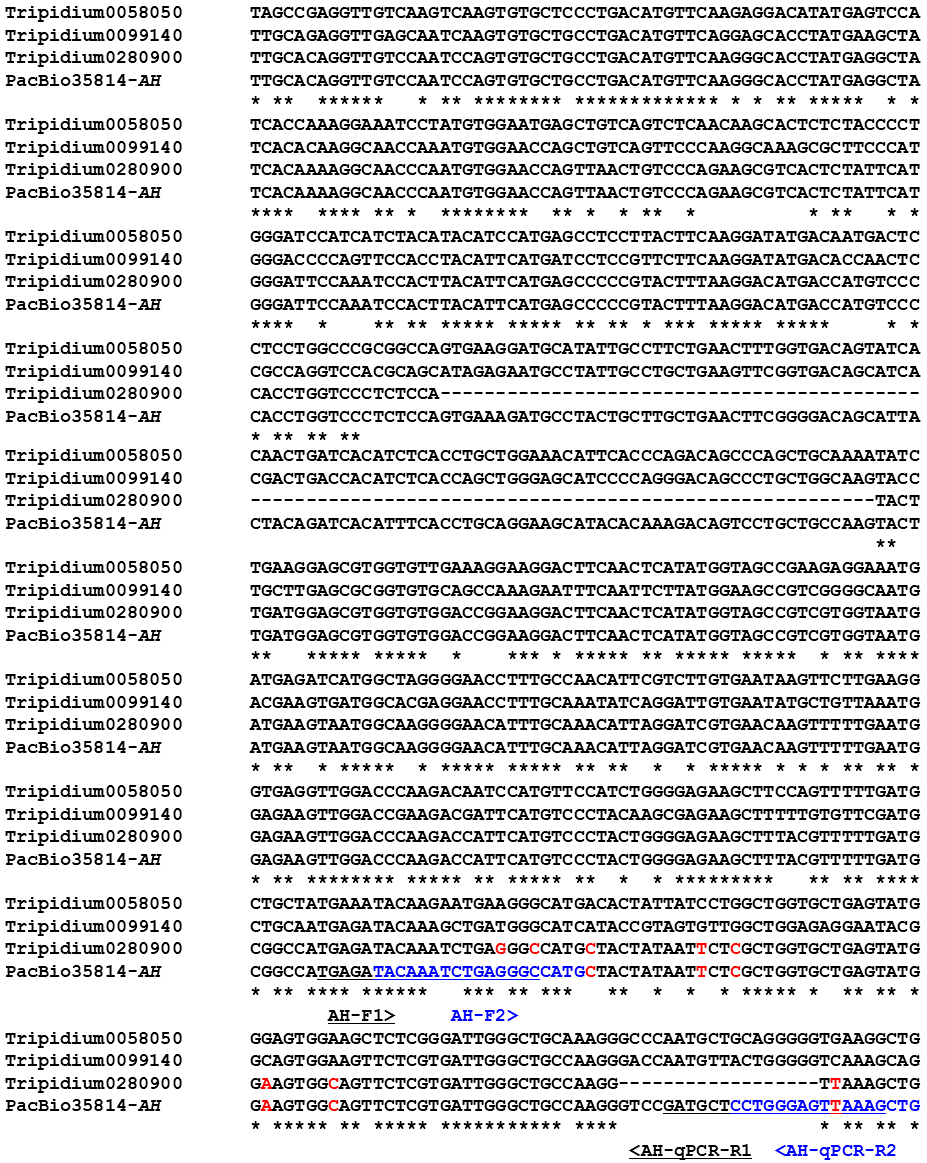
**

**Figure S17. (Cont.)**

**
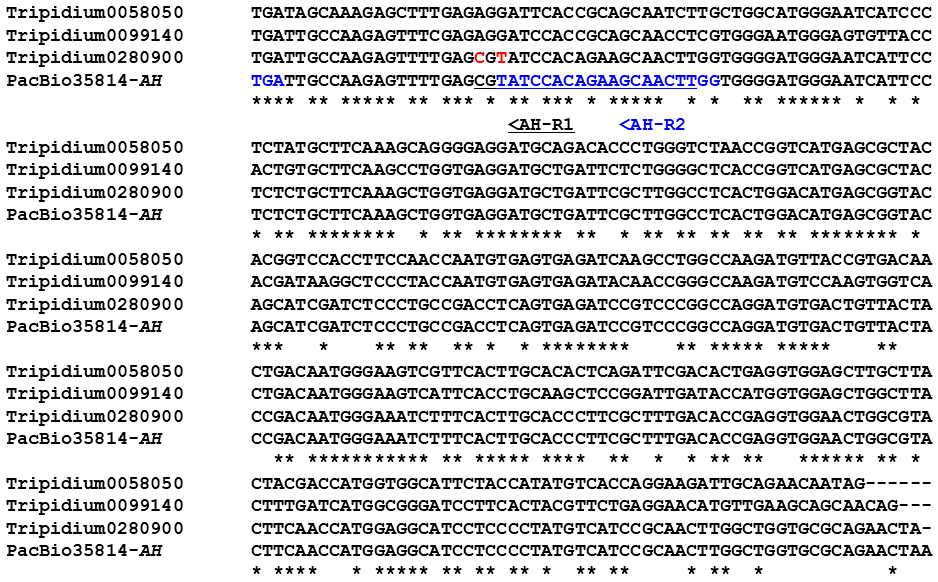
**

**Figure S18. The Sanger sequencing results of the PCR products amplified by the primers for the amplification of both the PacBio sequences of the 5 candidate reference genes, i.e., *Ubi4* (A), *Aldolase* (B), *BI1* (C), *8C* (D), and *AH* (E),** **and their most similar homologs in *T. ravennae*** **at the same time without cloning.** Dots underneath the consensus sequences indicate the mismatch between the PacBio sequence and its most similar homolog of each gene in the *T. ravennae* genome.


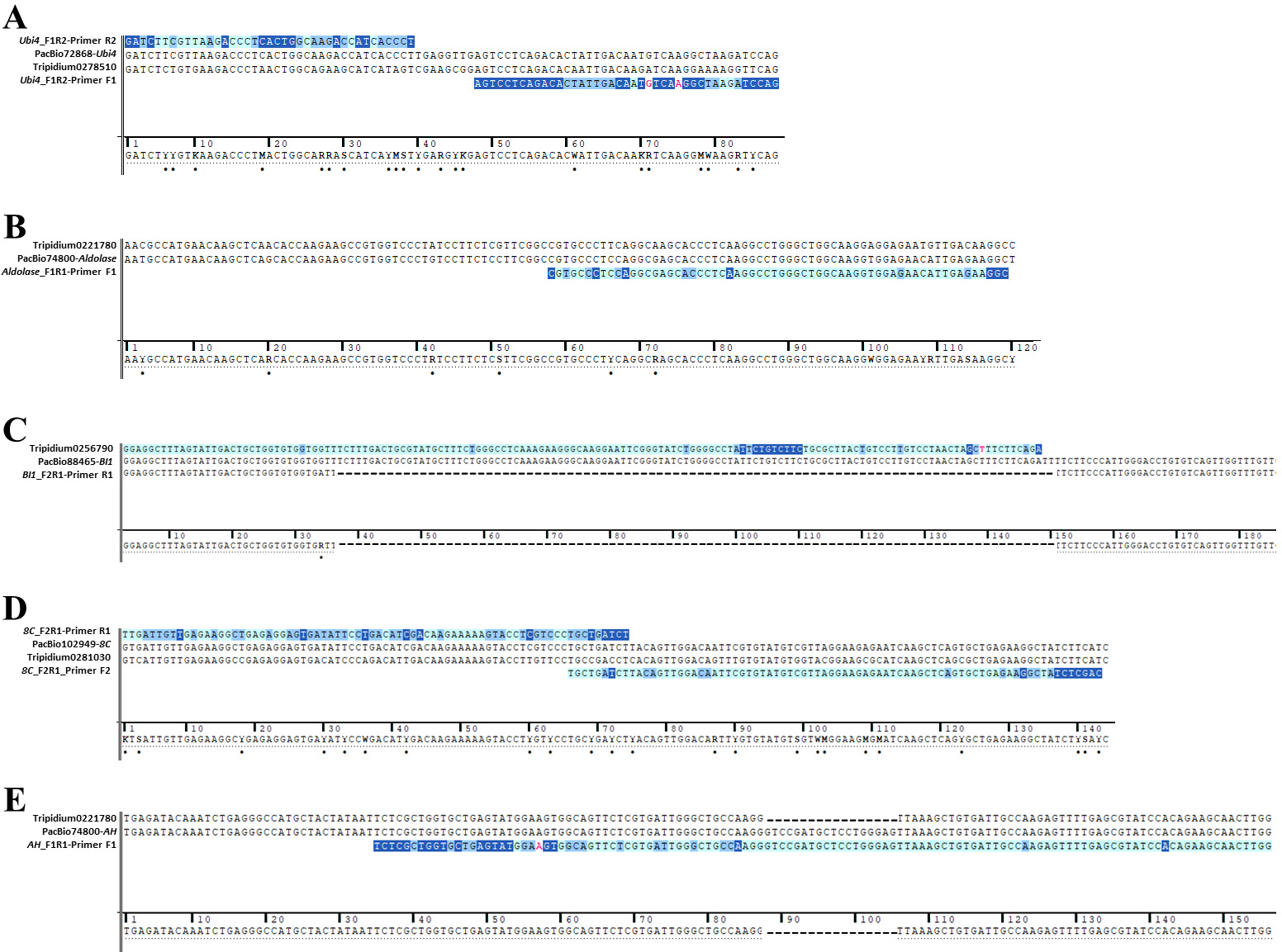


**Figure S19. Melting curves of all the 8 candidate reference genes in *T. ravennae*.** Gradient PCR with different annealing temperatures (56, 58, 60.6, 62.7℃) was conducted using each of the four primer pairs for each PacBio sequence. The single peak indicates the specificity of the primers used in qPCR amplification at 60.6℃ while the different peaks (the blue font circles) arose from PCR amplification at 56°C.

**
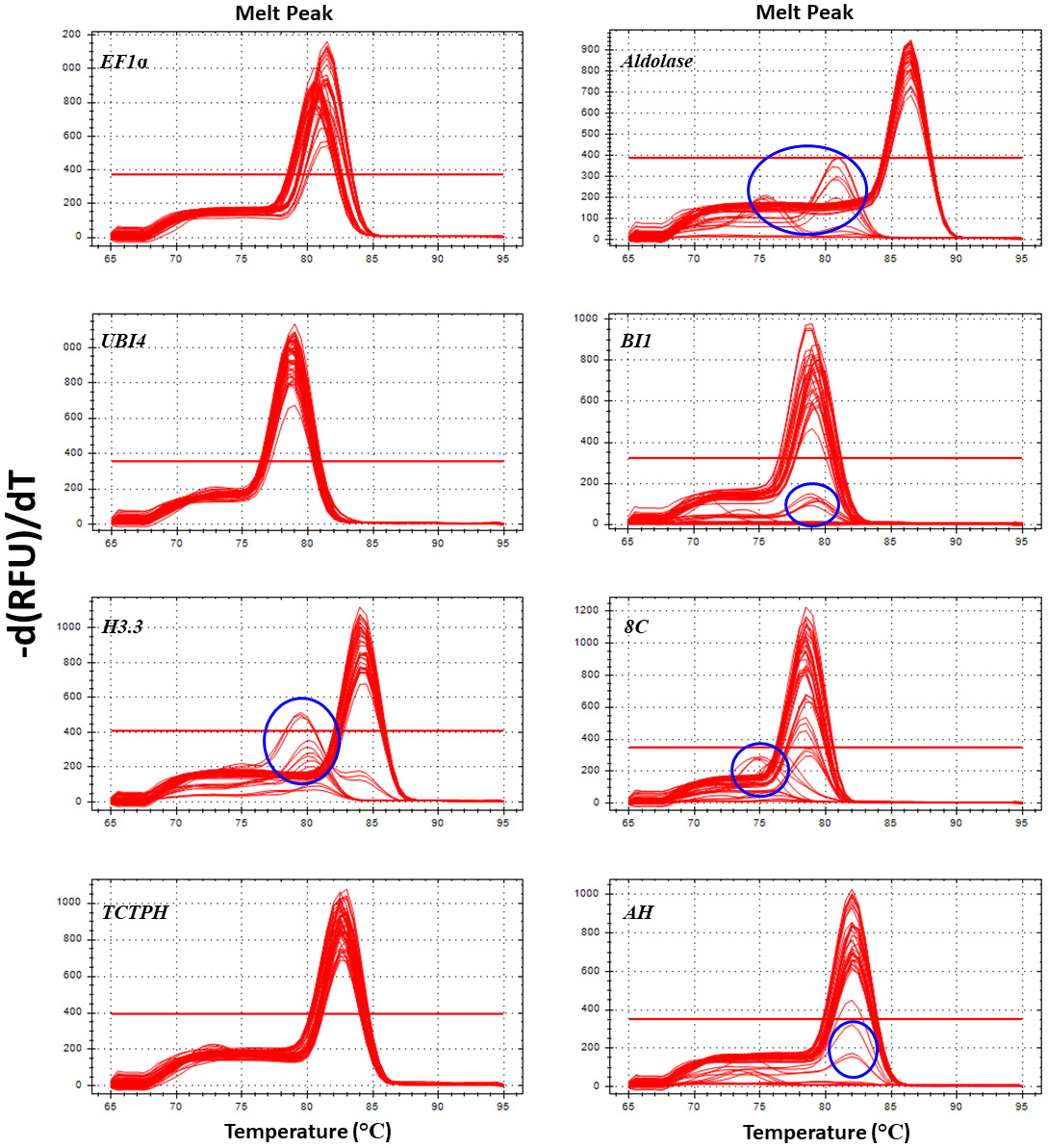
**

**Figure S20.** **Alignment of the** **deduced protein sequences of the soybean *Actin* (*Glyma.08G182200*) gene and its homologous sequences in soybean for qPCR primer design.**

**
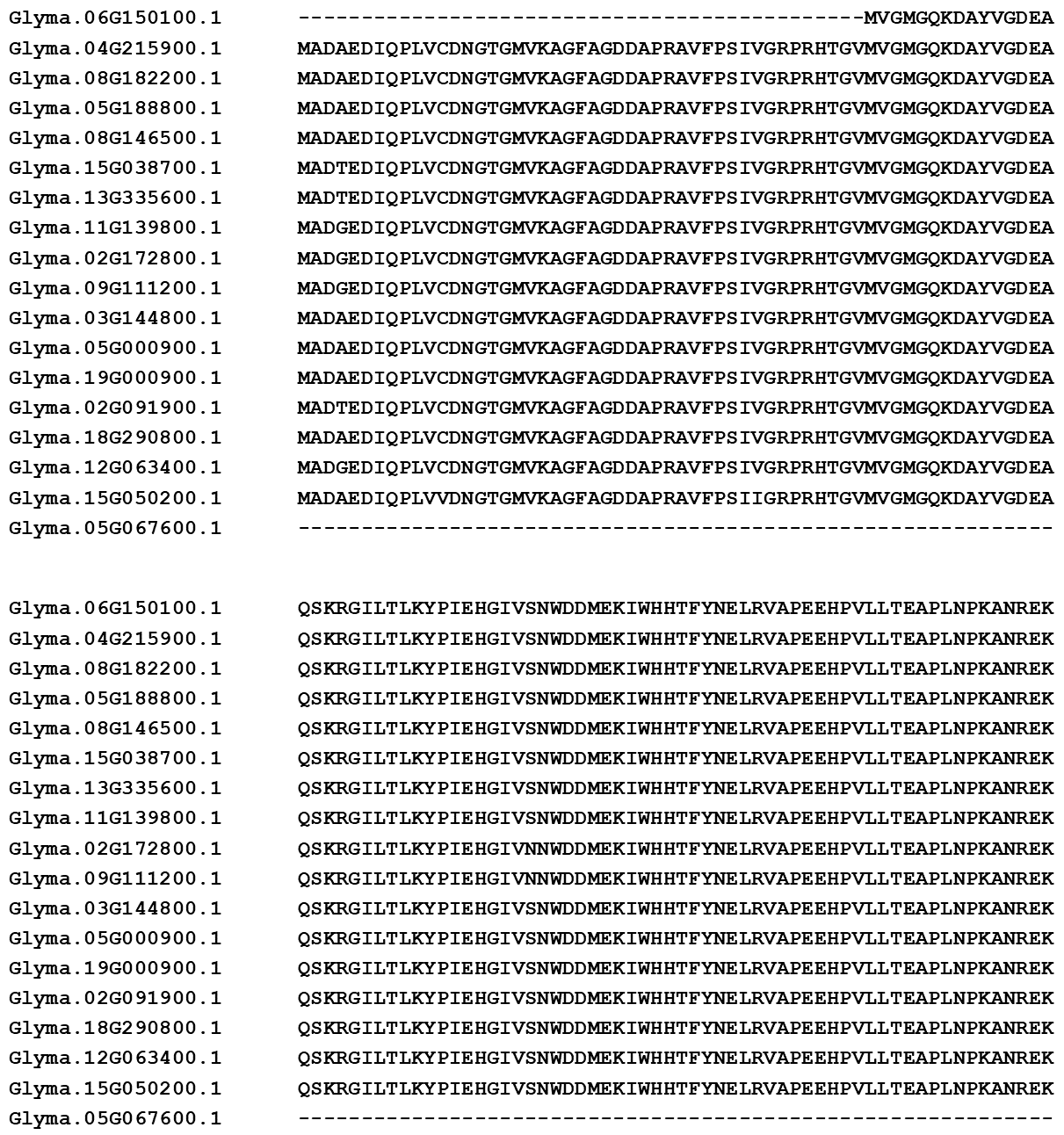
**

**Figure S20. (Cont.)**

**
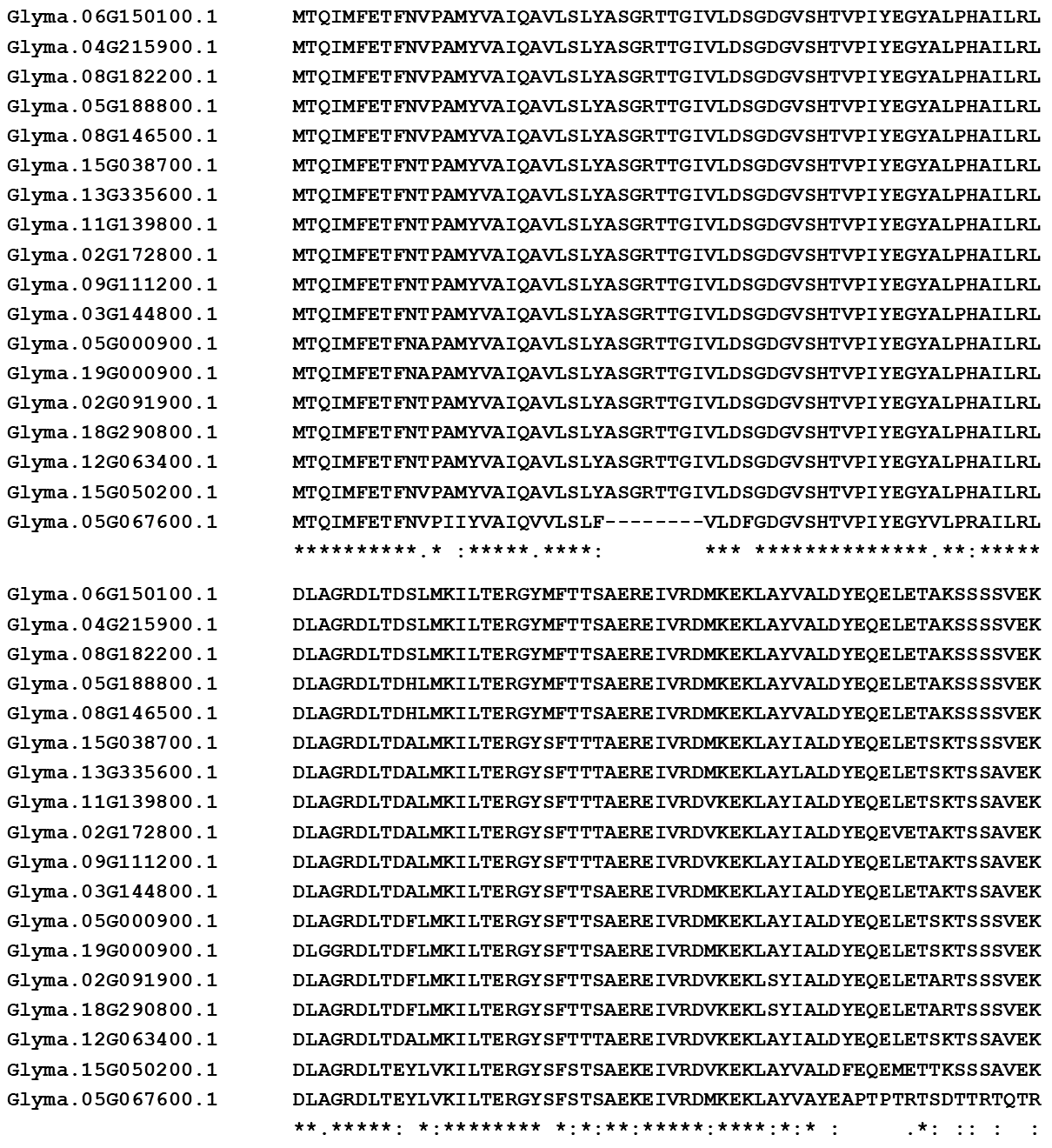
**

**Figure S20. (Cont.)**

**
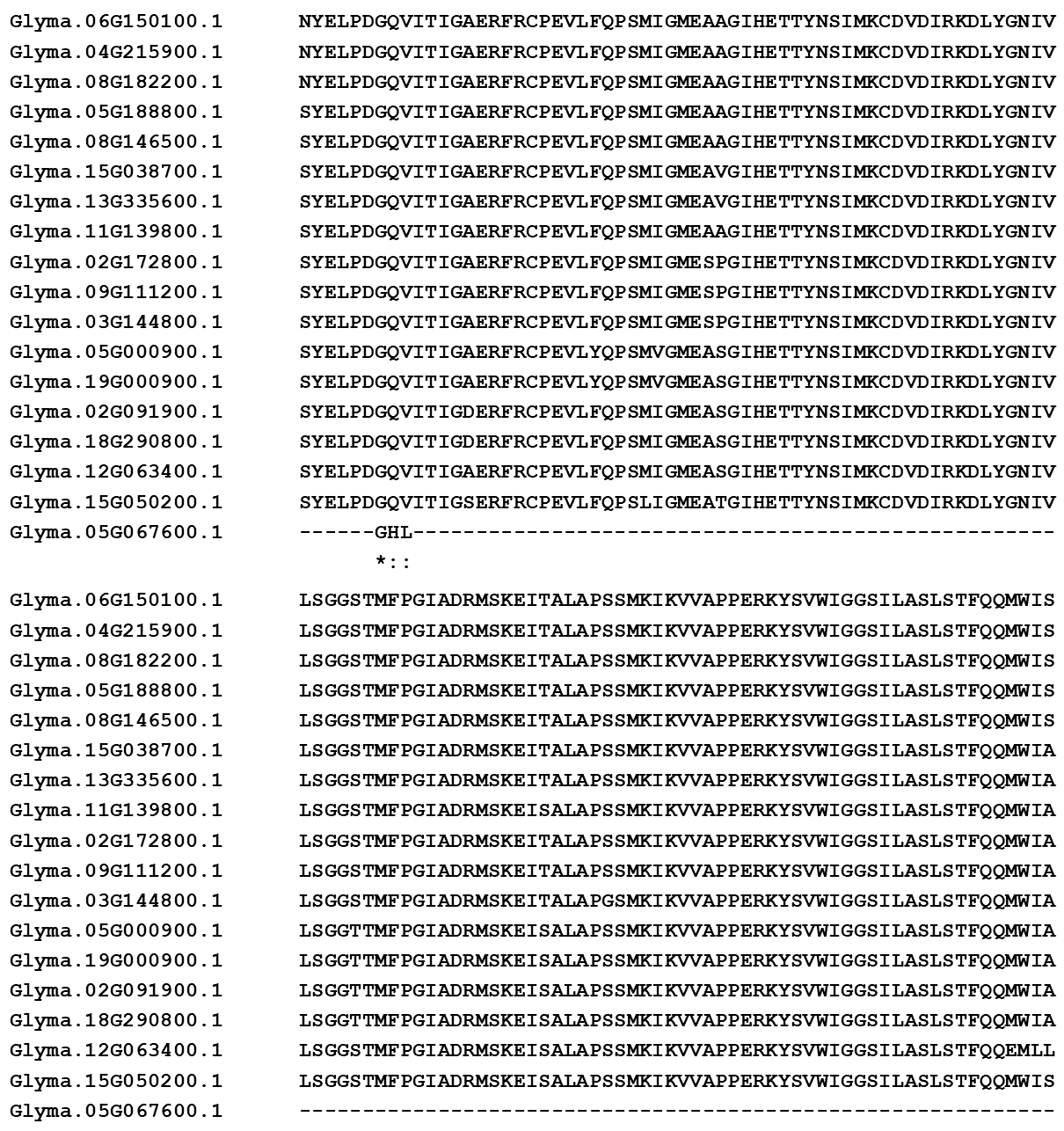
**

**Figure S20. (Cont.)**

**
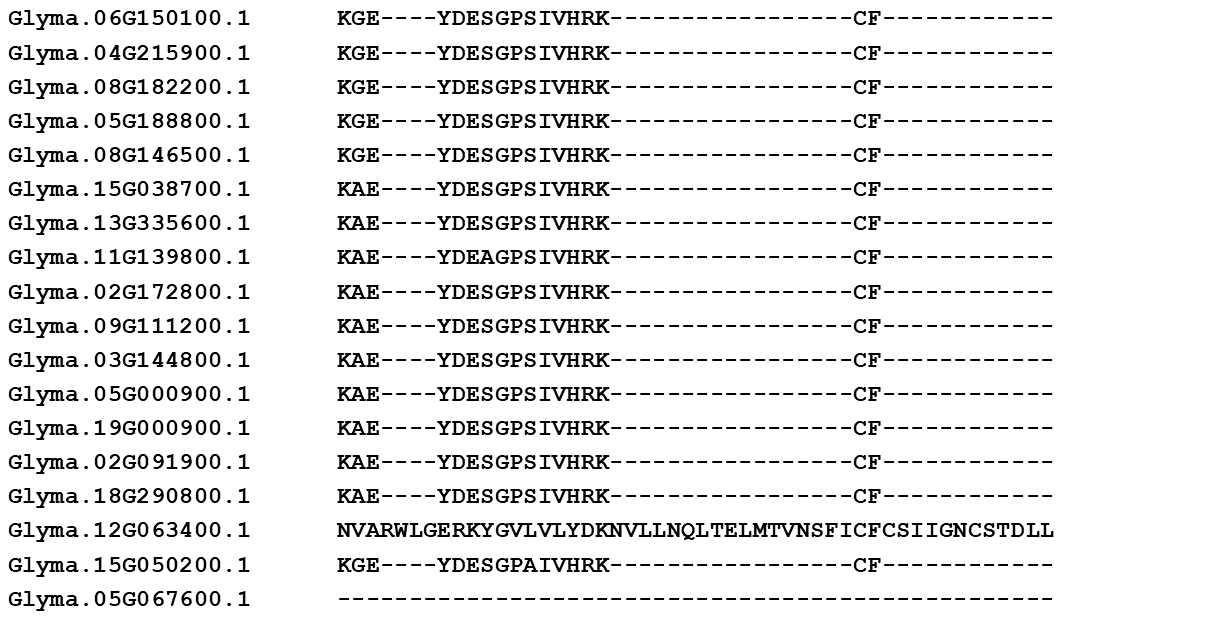
**

**Figure S21.** **Alignment of the** **deduced protein sequences of the soybean *cons4* (*Glyma.12G020500*) gene and its homologous sequences in soybean for qPCR primer design.**

**
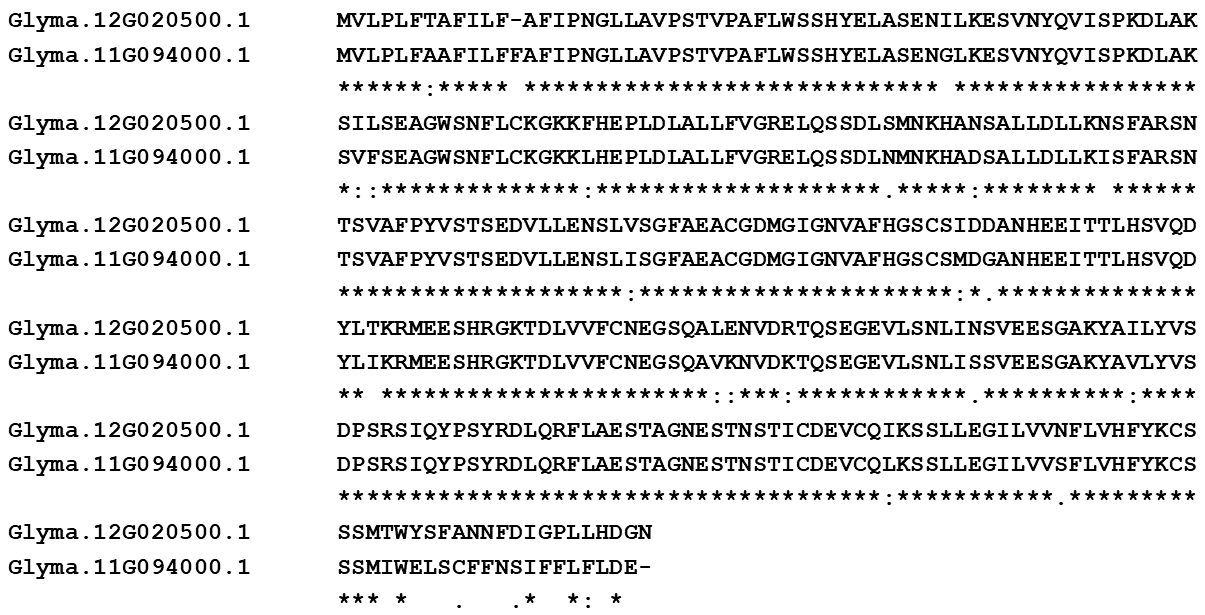
**

**Figure S22.** **Alignment of the** **deduced protein sequences of the soybean *cons6* (*Glyma.12G051100*) gene and its homologous sequences in soybean for qPCR primer design.**

**
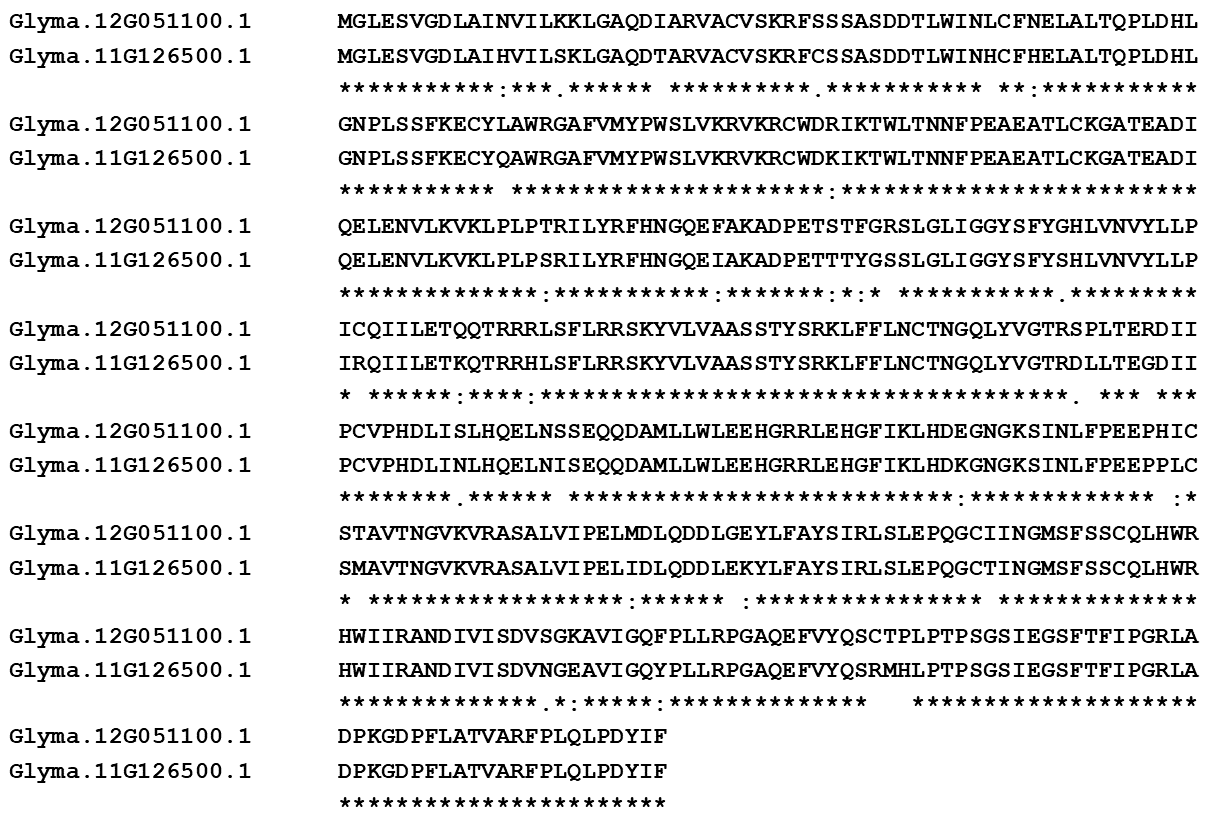
**

**Figure S23.** **Alignment of the** **deduced protein sequences of the soybean *Tubulin* (*Glyma.08G014200.1*) gene and its homologous sequences in soybean for qPCR primer design.**

**
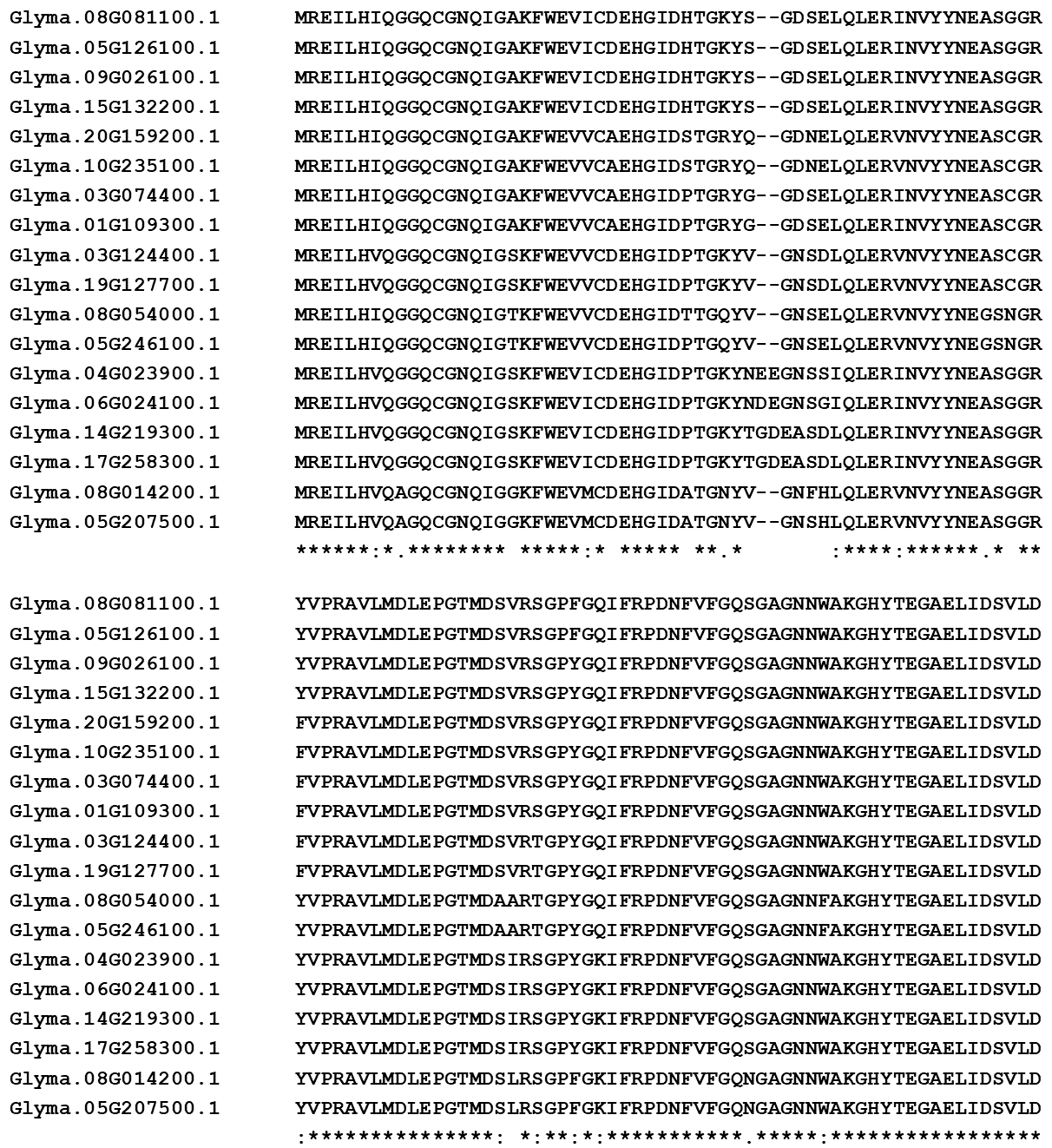
**

**Figure S23. (Cont.)**

**
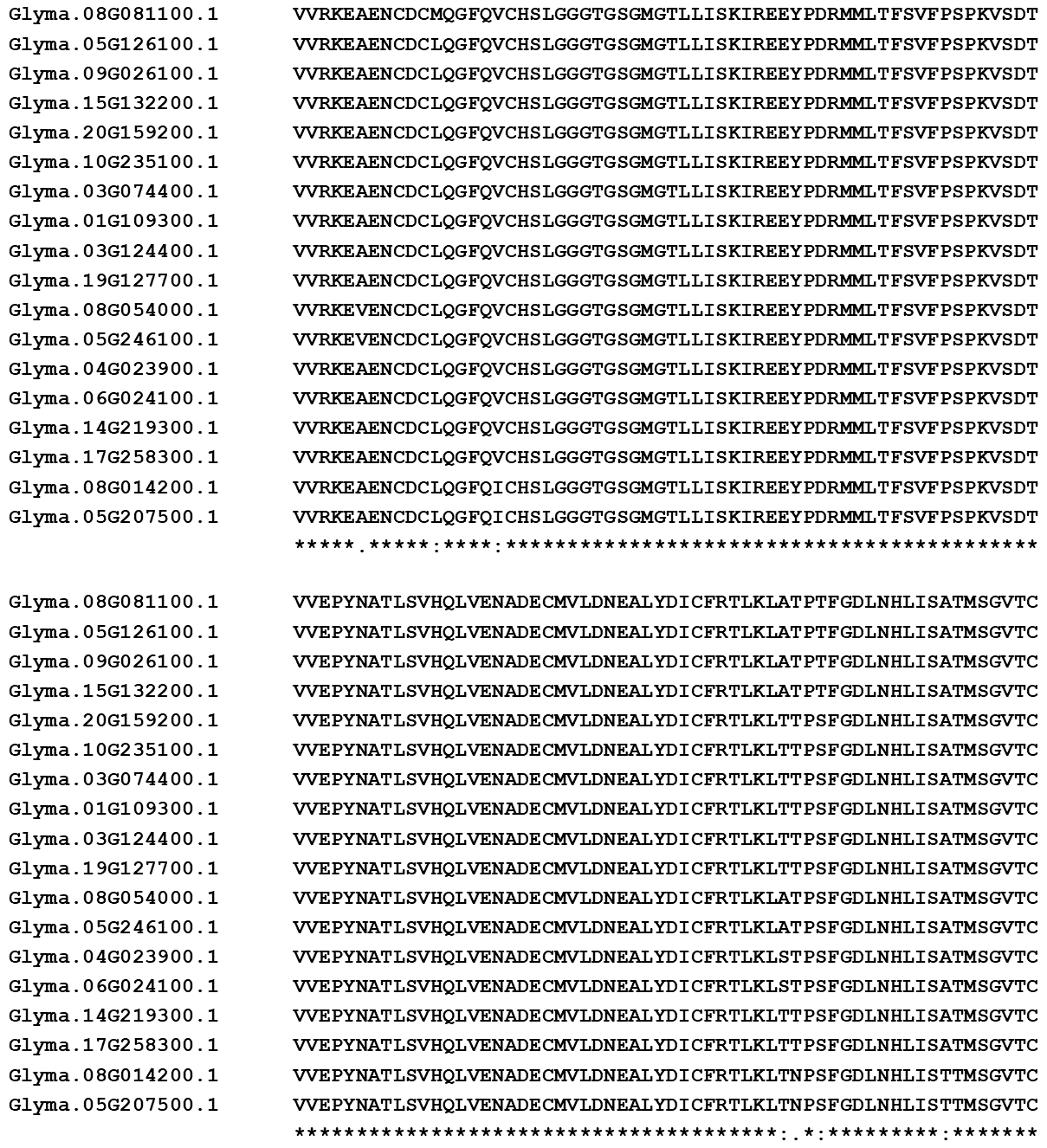
**

**Figure S23. (Cont.)**

**
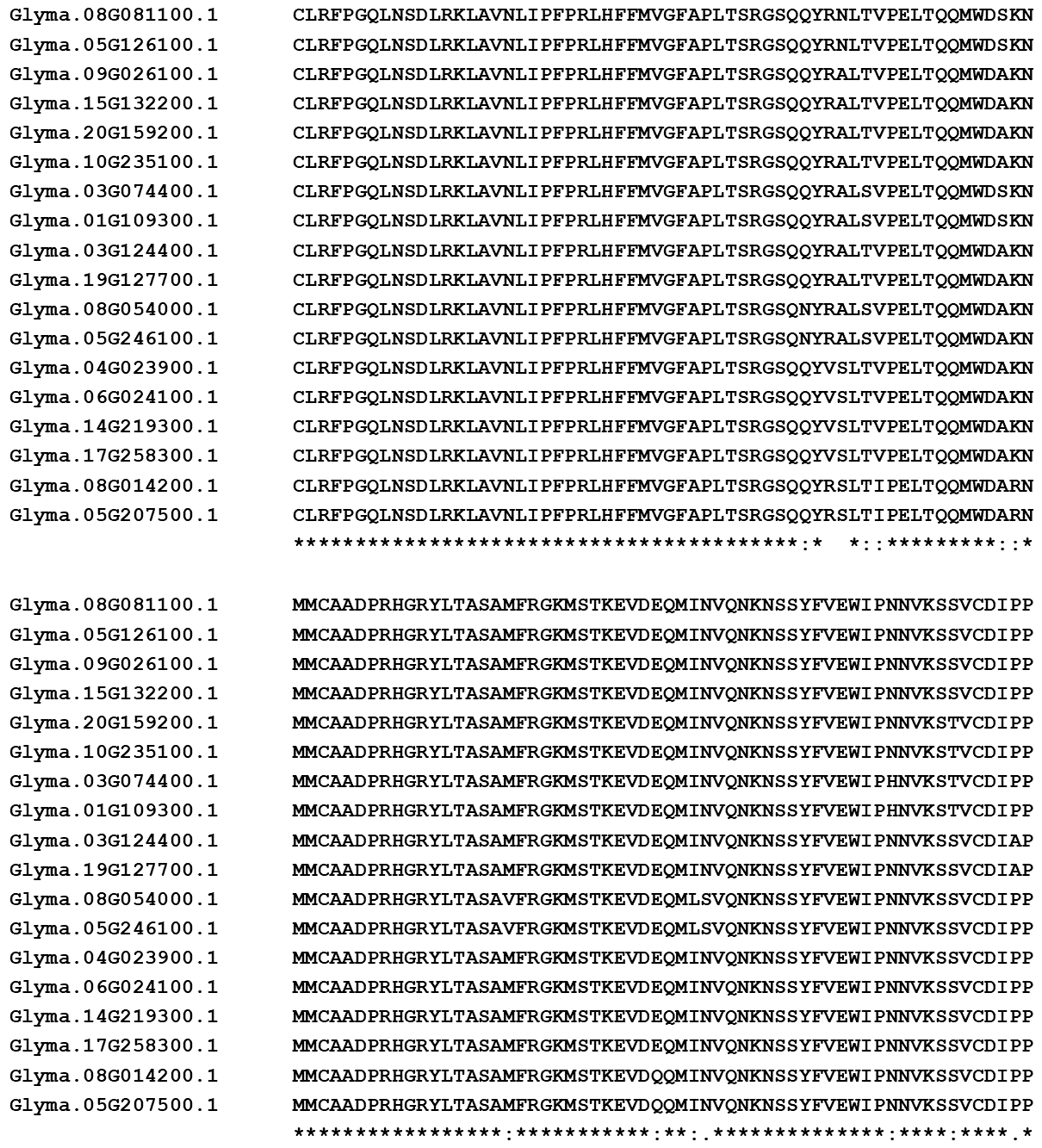
**

**Figure S23. (Cont.)**

**
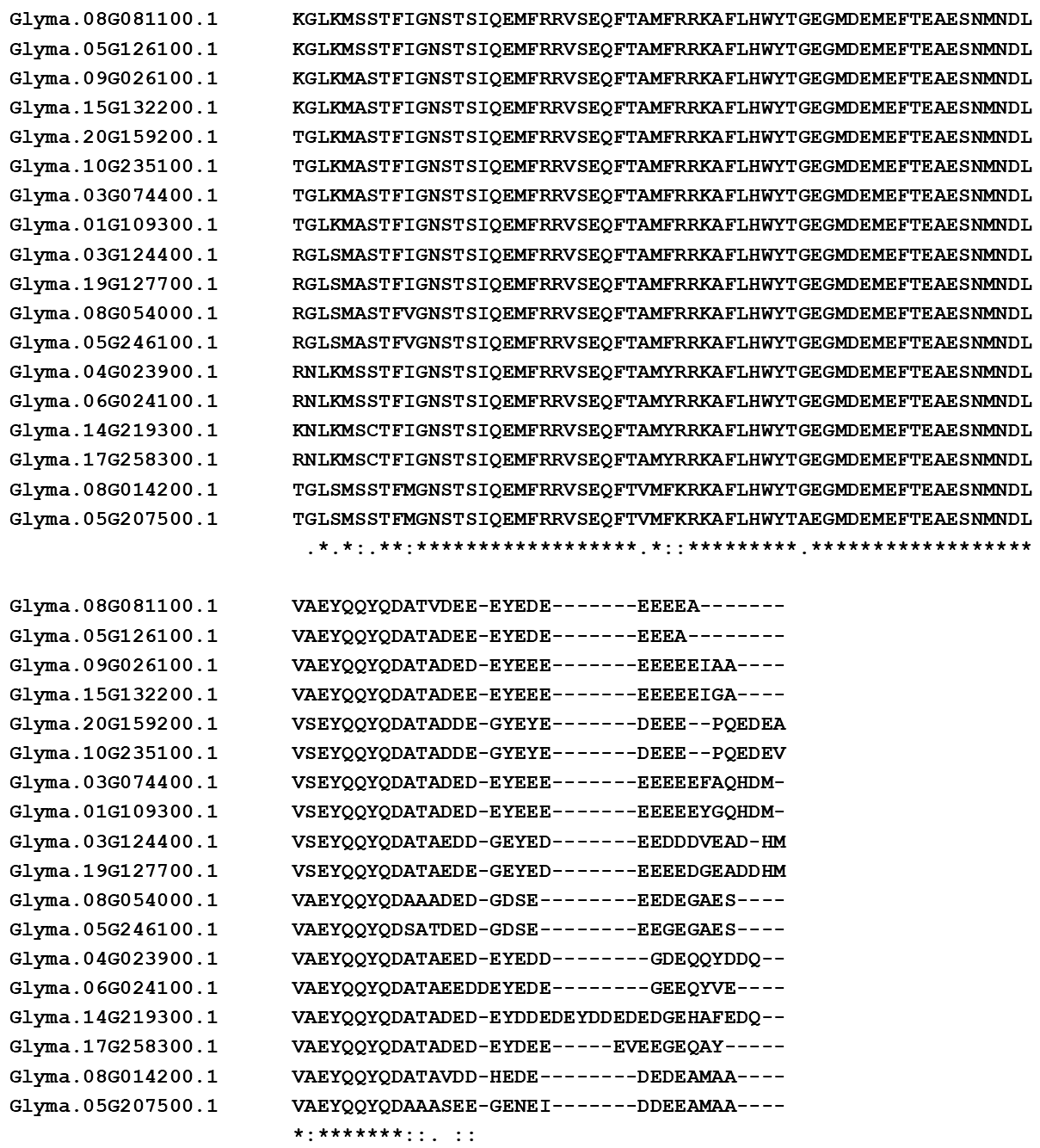
**

**Figure S24.** **Alignment of the** **deduced protein sequences of the soybean *FtsZ2-1* (*Glyma.19G194800*) and its homologous sequences in soybean for qPCR primer design.**

**
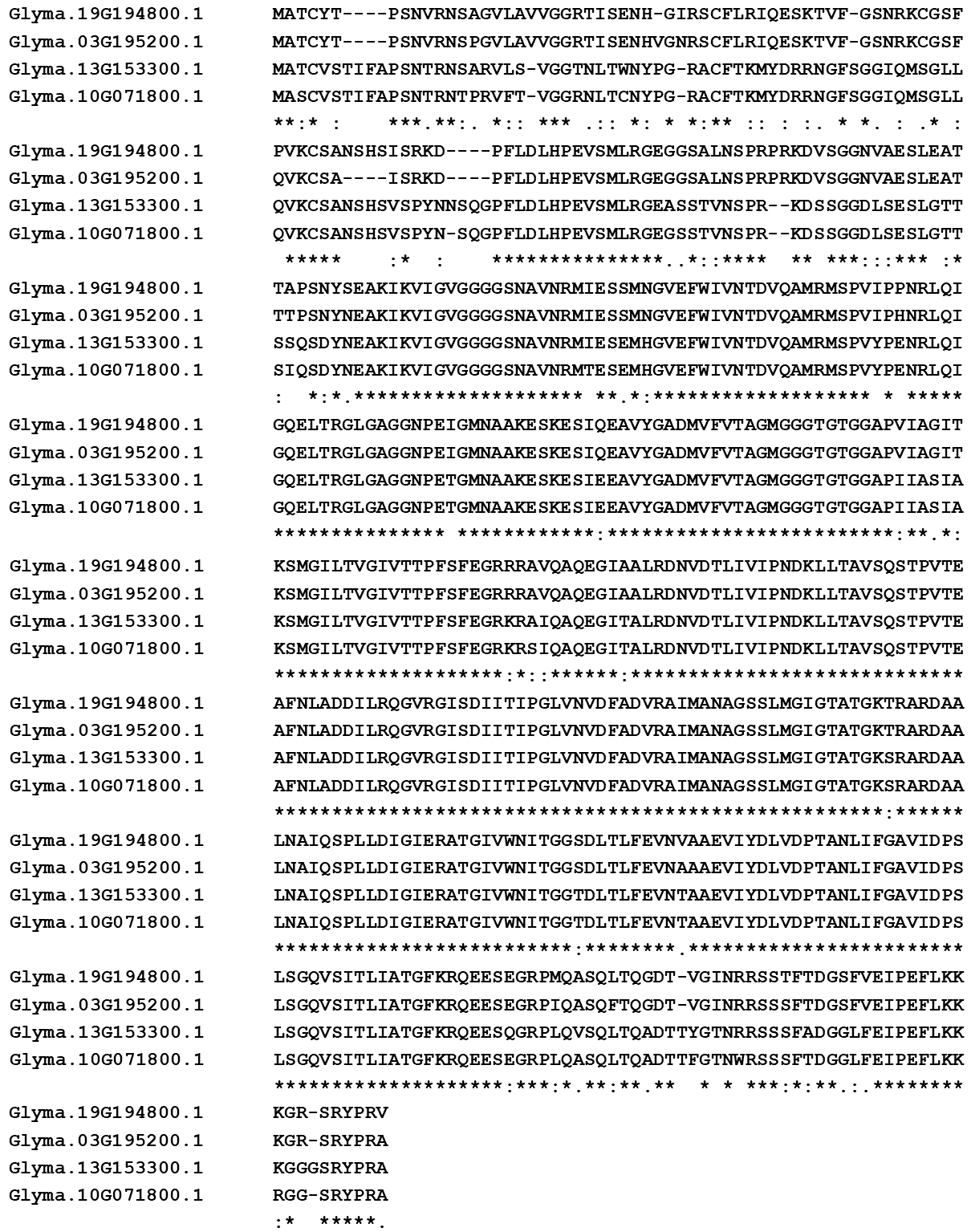
**

**Figure S25.** **Alignment of the** **deduced protein sequences of the soybean *TUBB* (*Glyma.04G023900*) gene and its homologous sequences in soybean for qPCR primer design.**


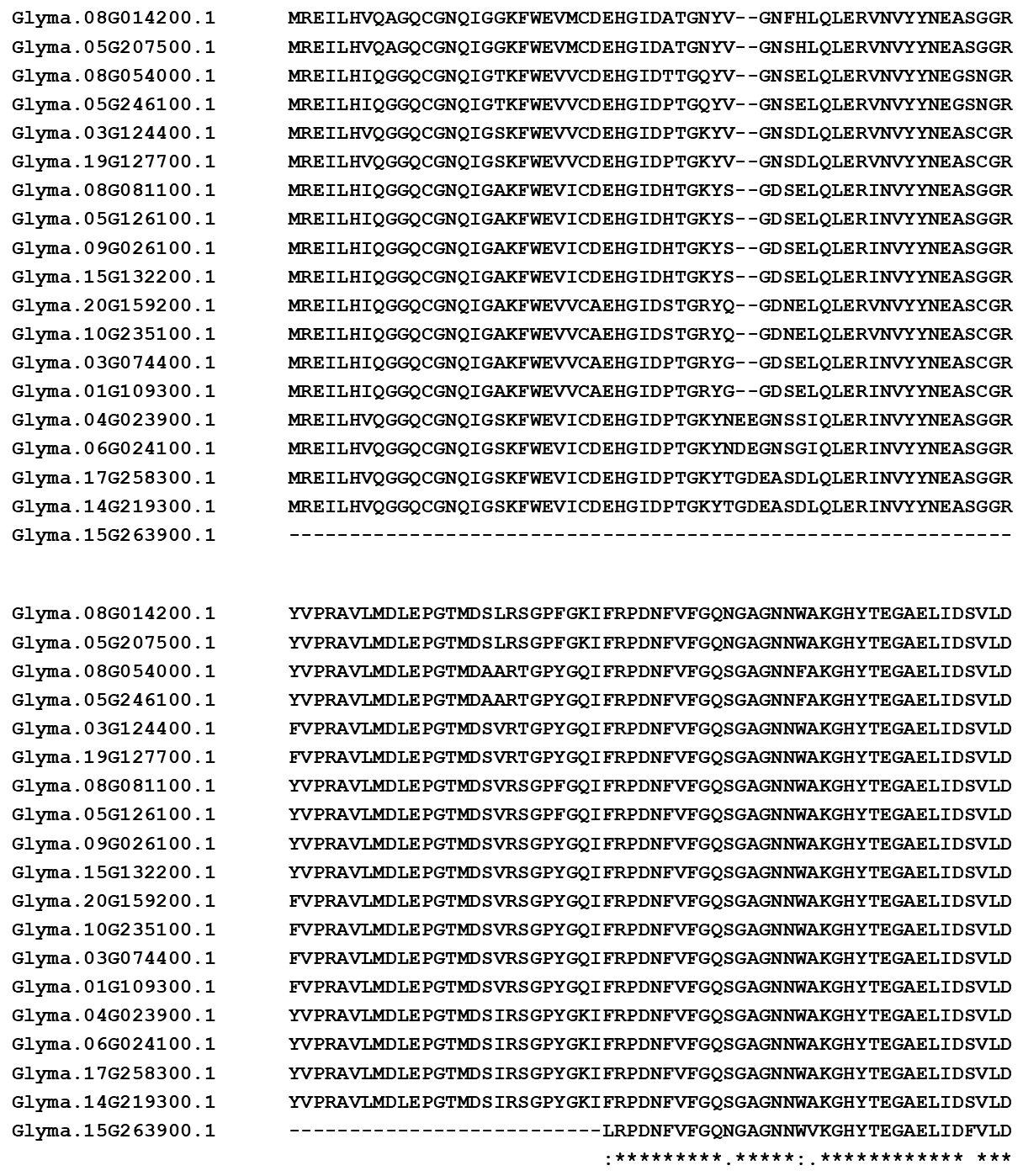


**Figure S25. (Cont.)**


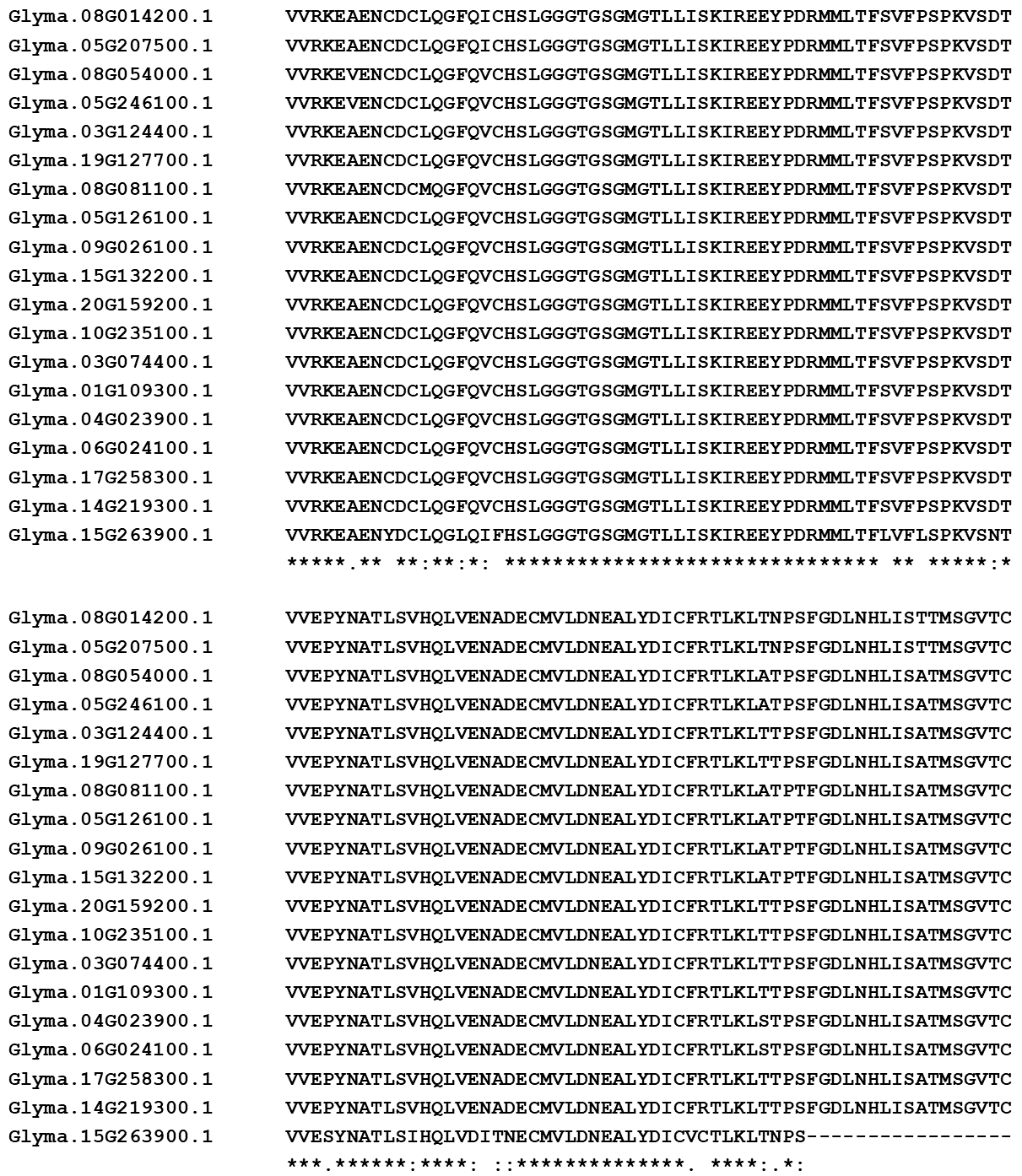


**Figure S25. (Cont.)**


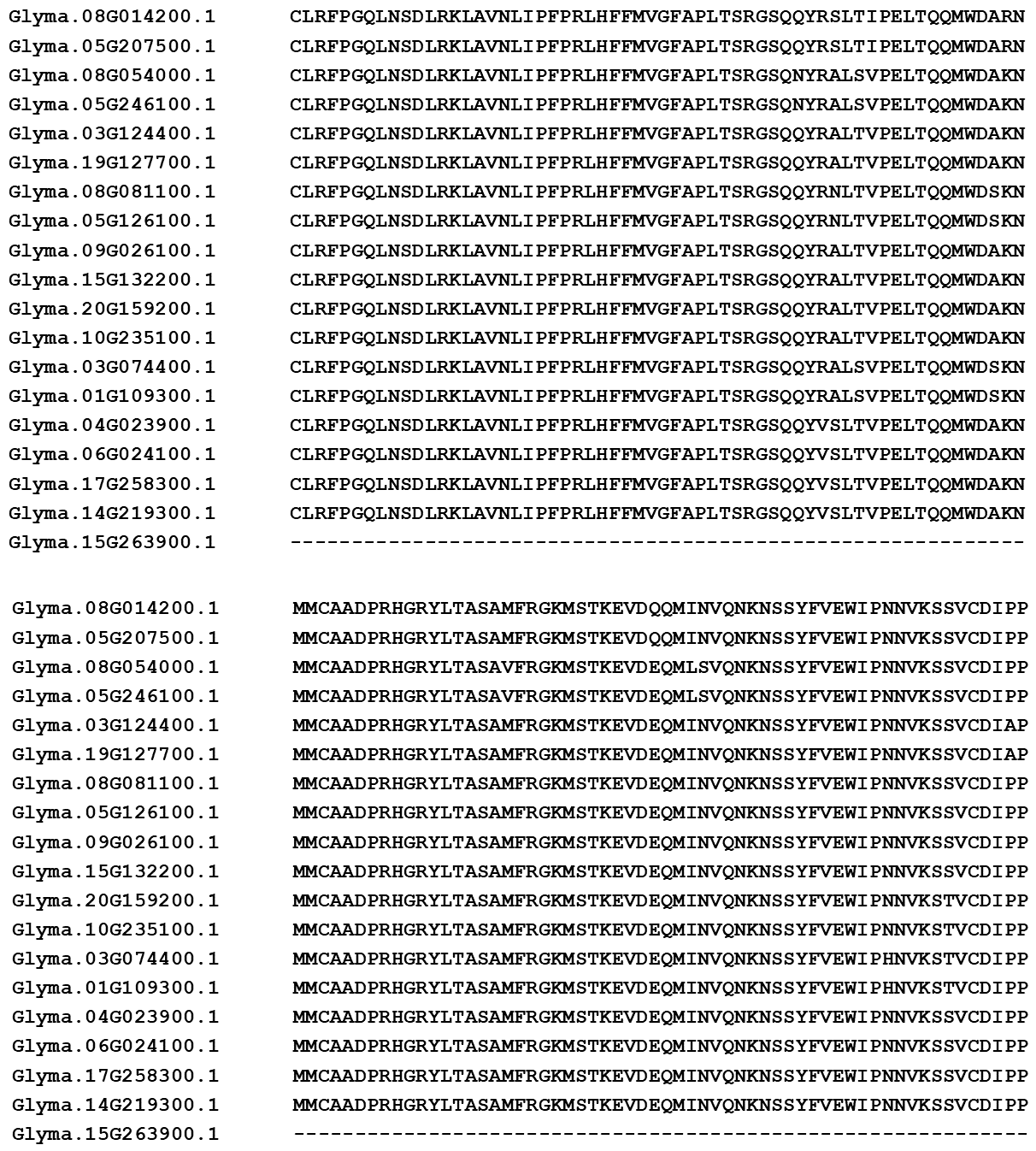


**Figure S25. (Cont.)**


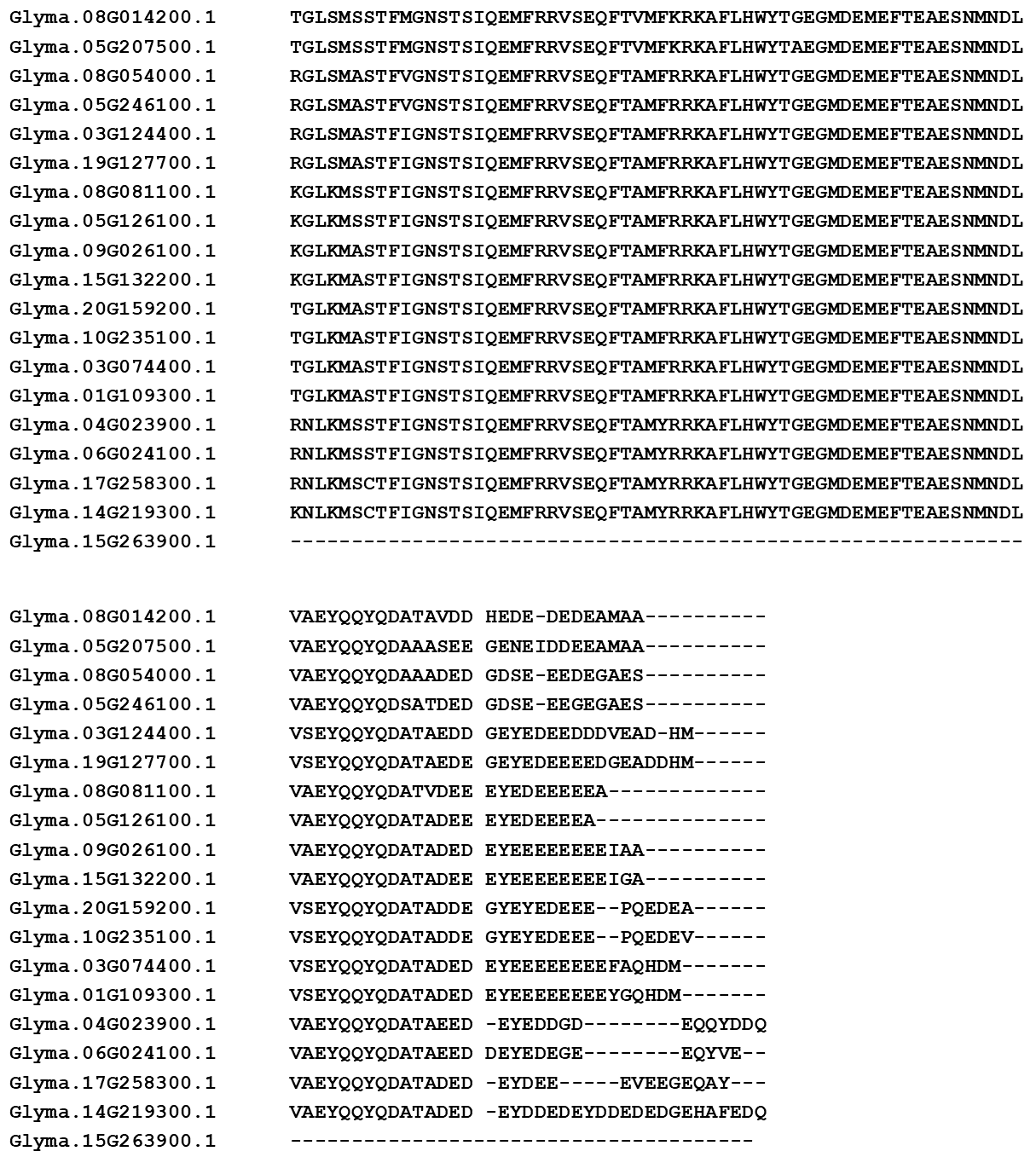


**Figure S26.** **Alignment of the** **cDNA sequences of the soybean *Actin* (*Glyma.08G182200*) gene and its homologous sequences in soybean for qPCR primer design.**

**
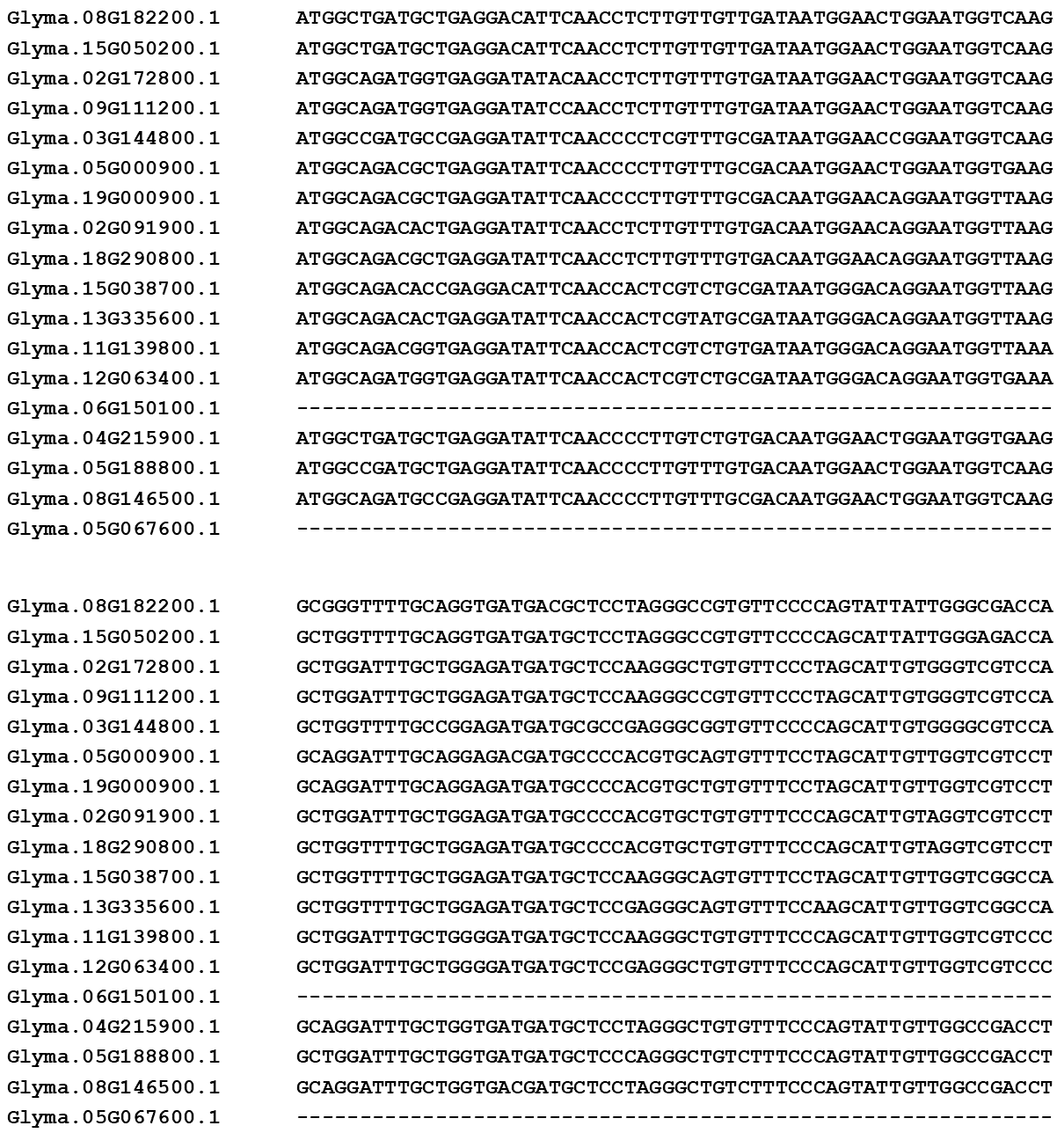
**

**Figure S26. (Cont.)**

**
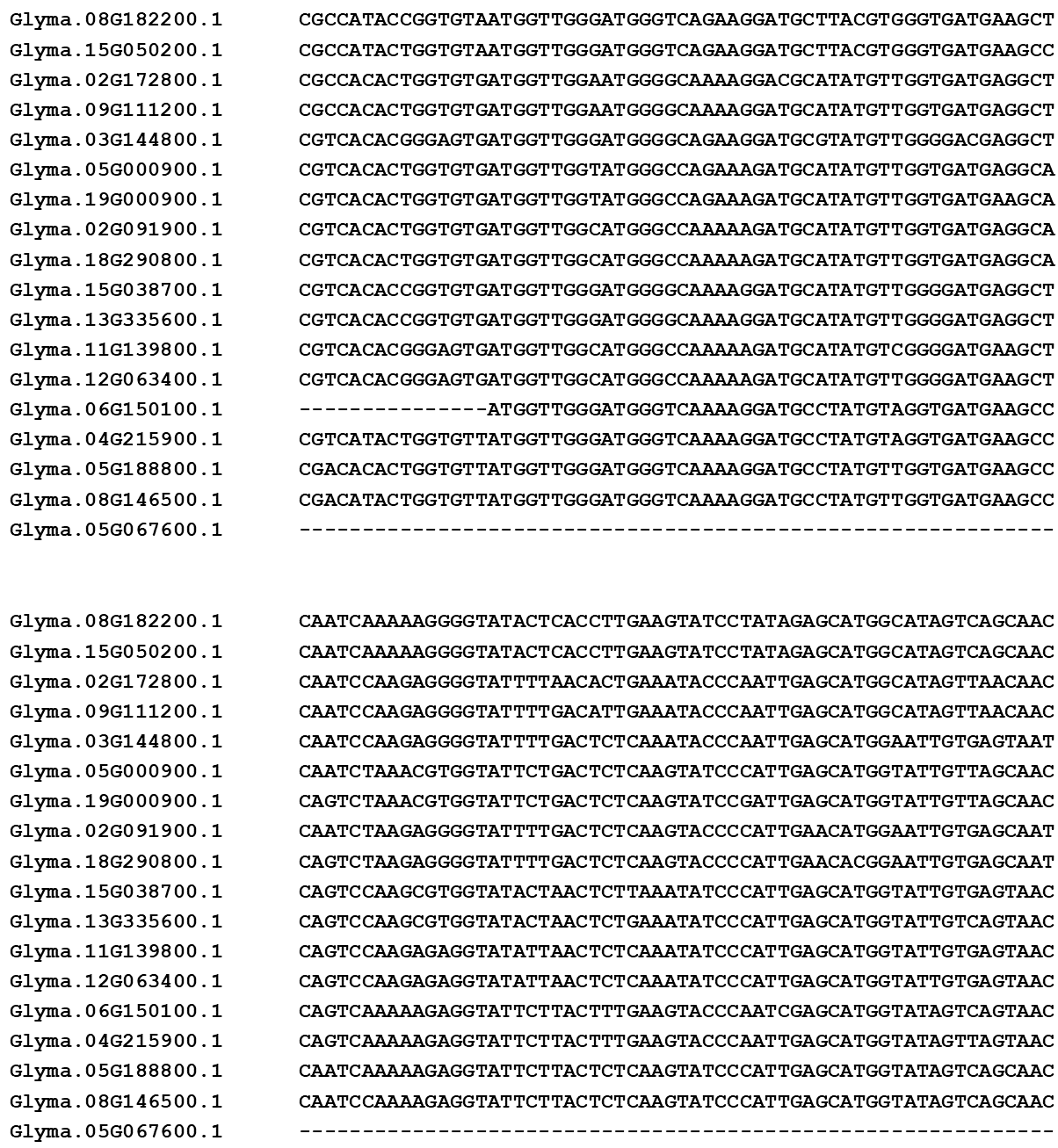
**

**Figure S26. (Cont.)**

**
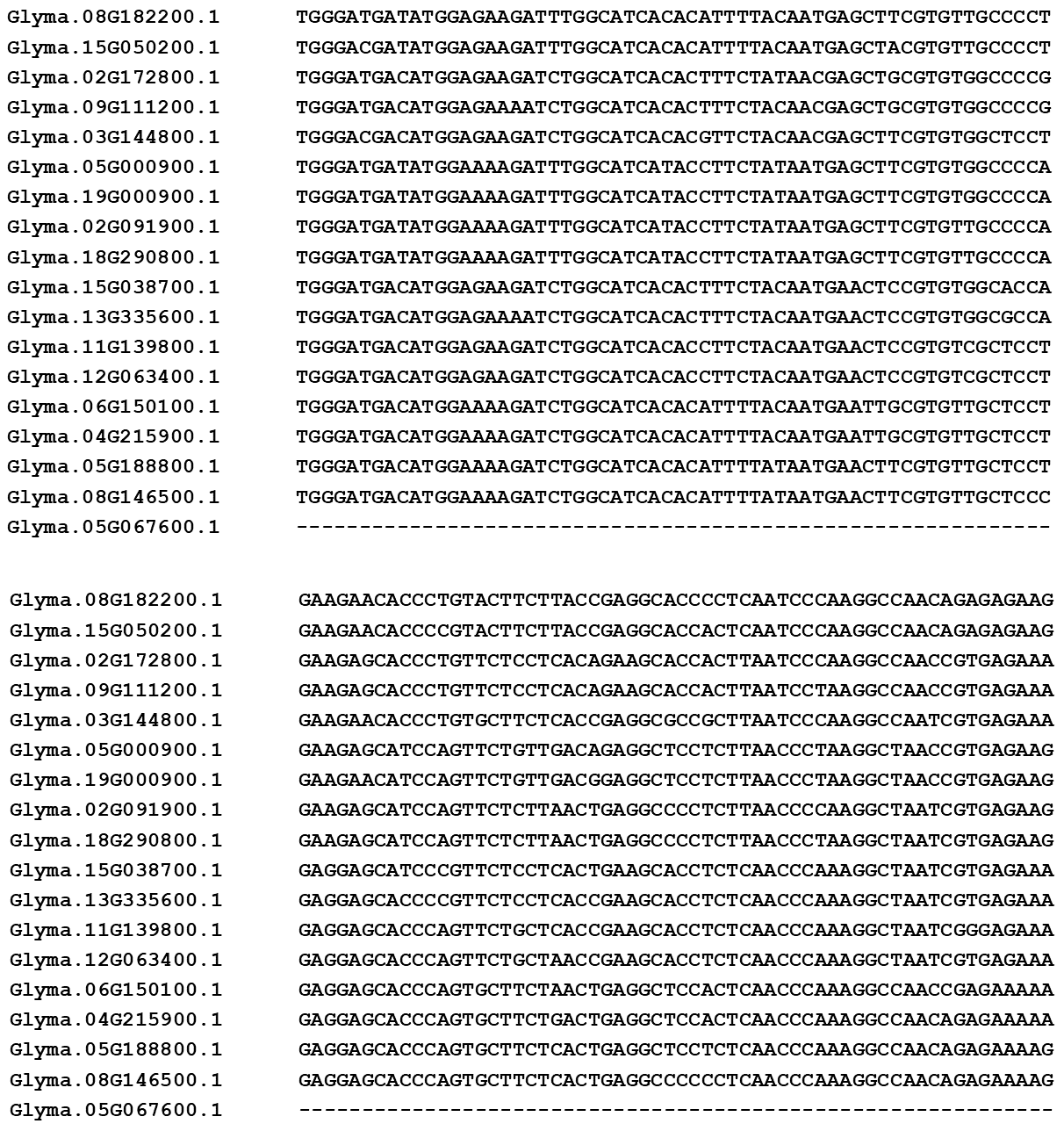
**

**Figure S26. (Cont.)**

**
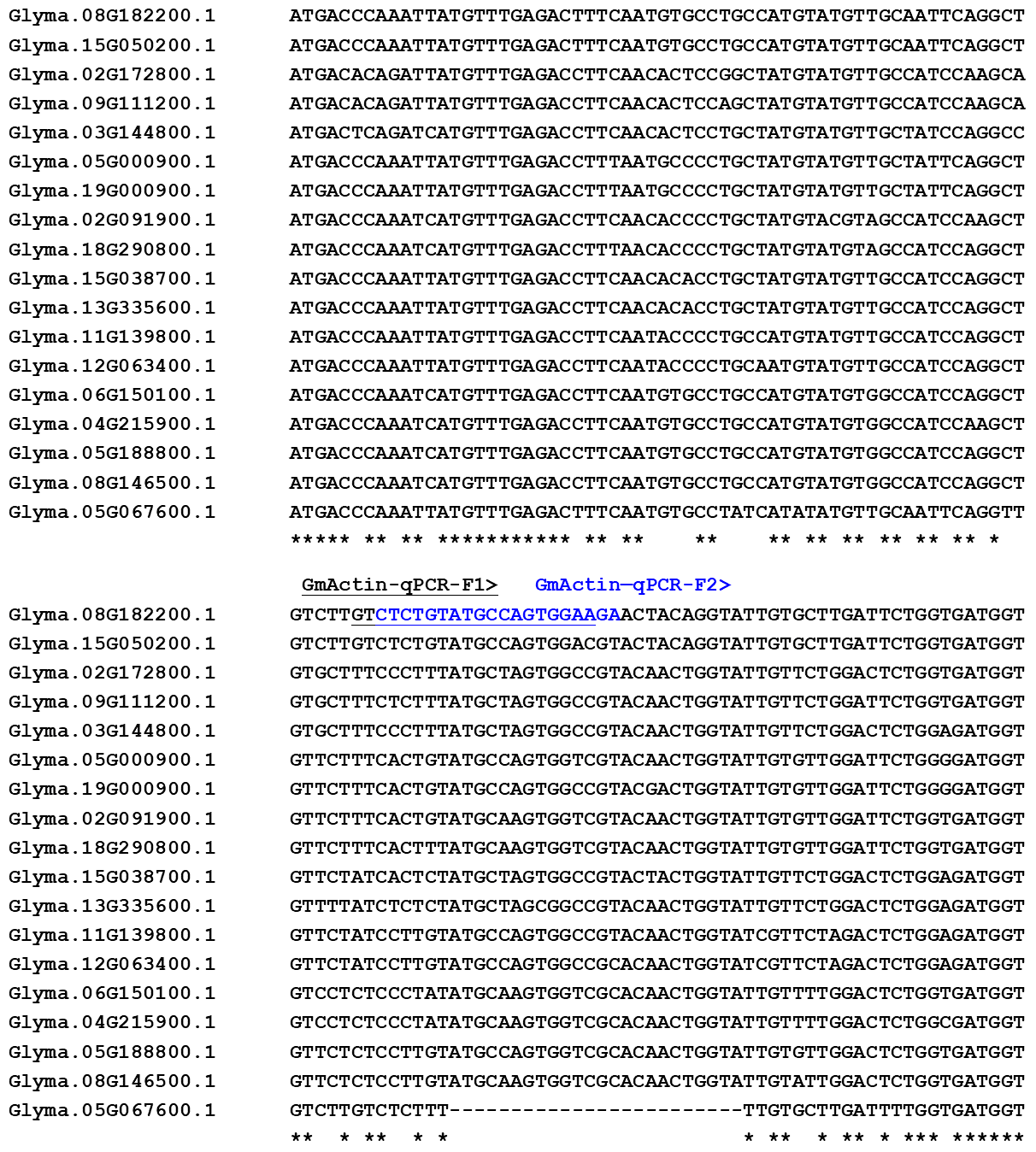
**

**Figure S26. (Cont.)**

**
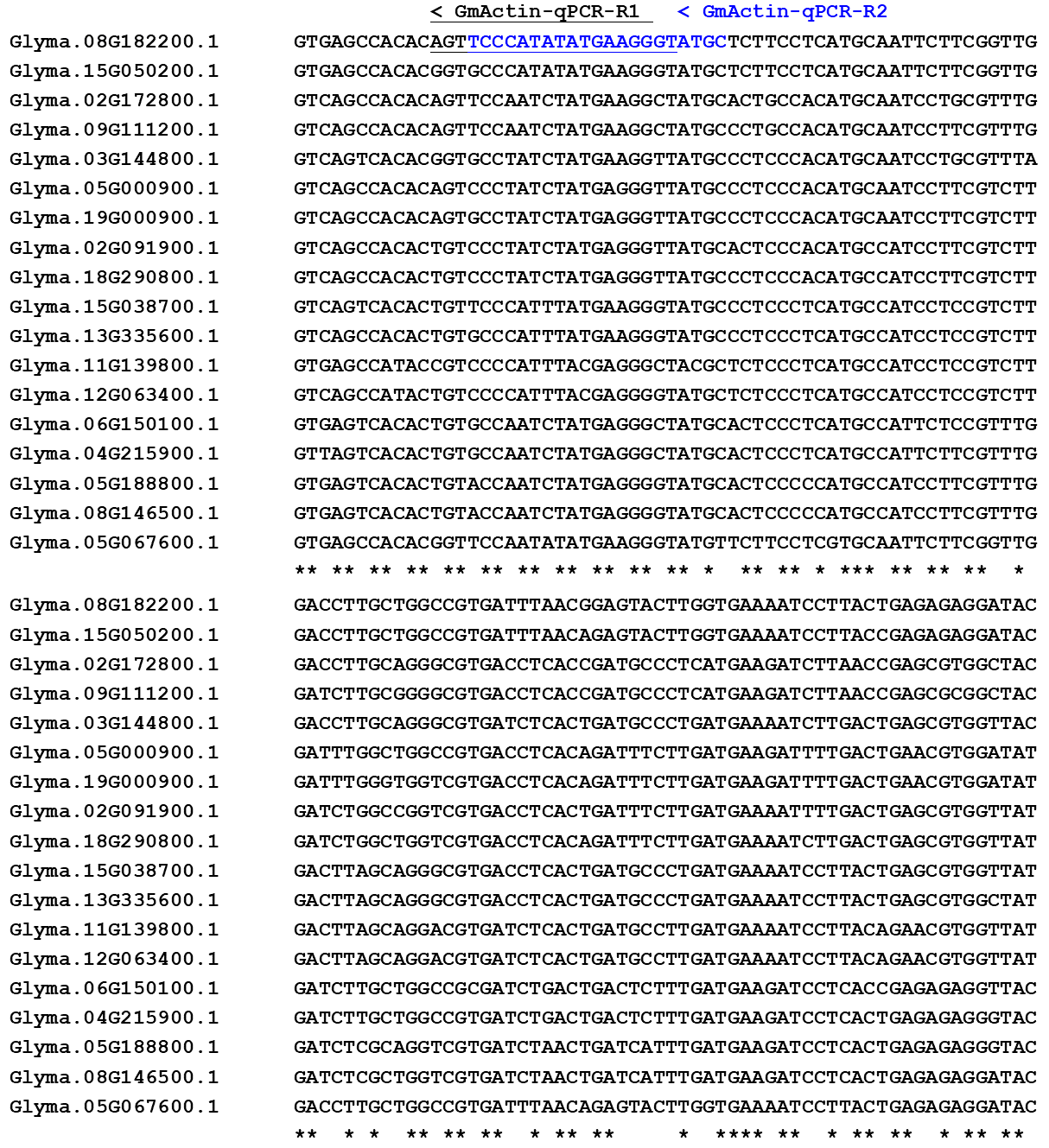
**

**Figure S26. (Cont.)**

**
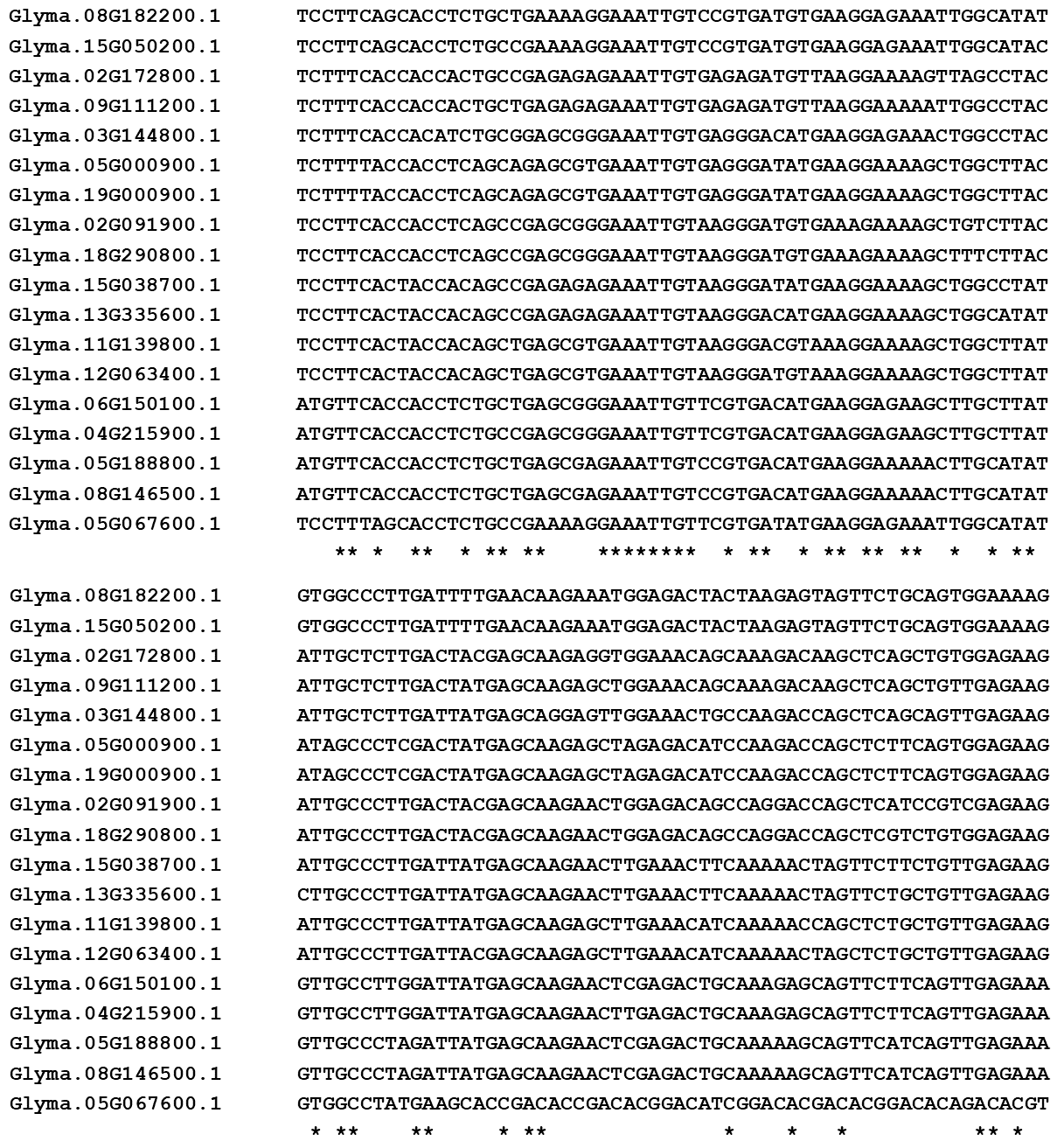
**

**Figure S26. (Cont.)**

**
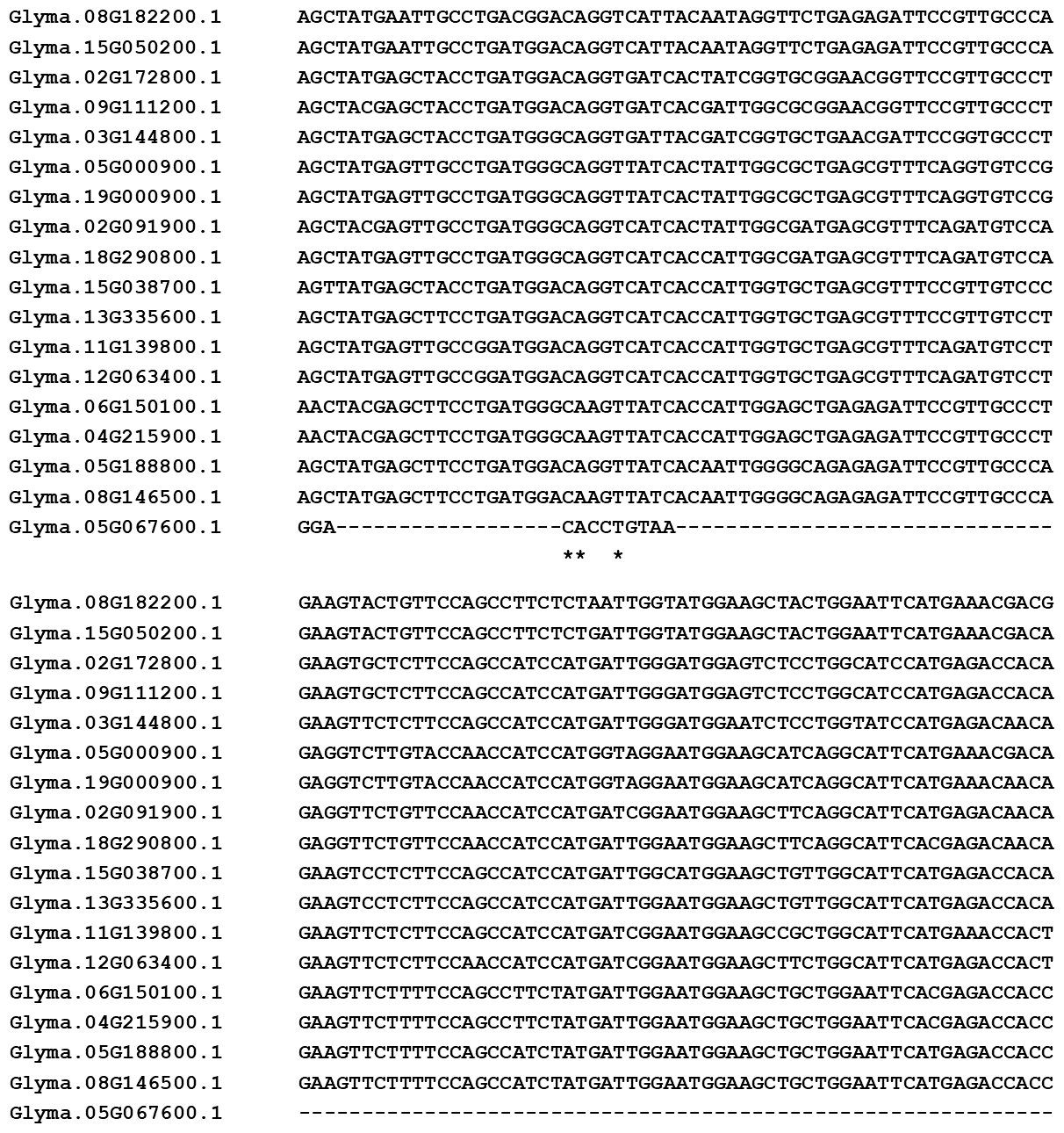
**

**Figure S26. (Cont.)**

**
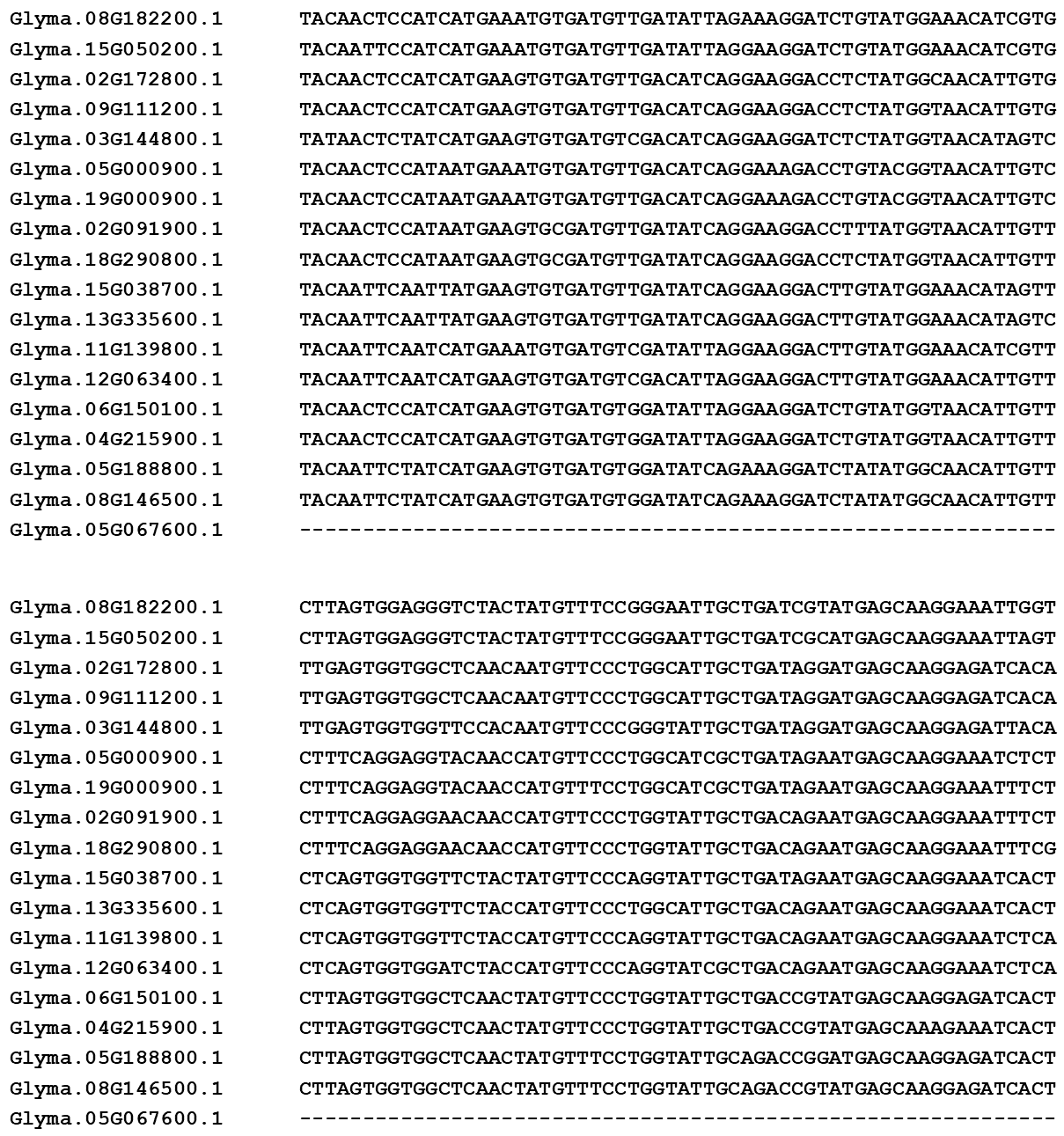
**

**Figure S26. (Cont.)**

**
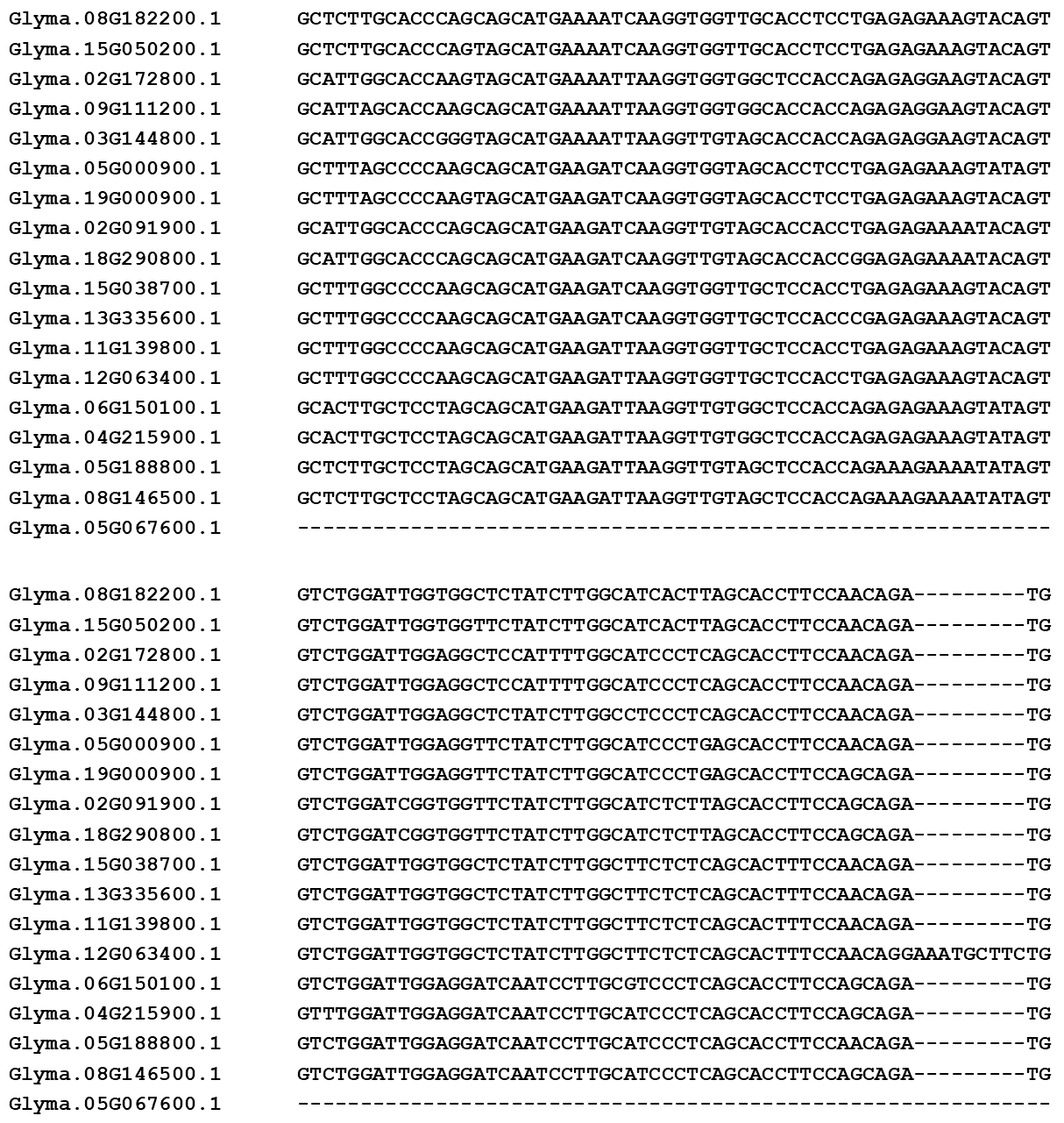
**

**Figure S26. (Cont.)**

**
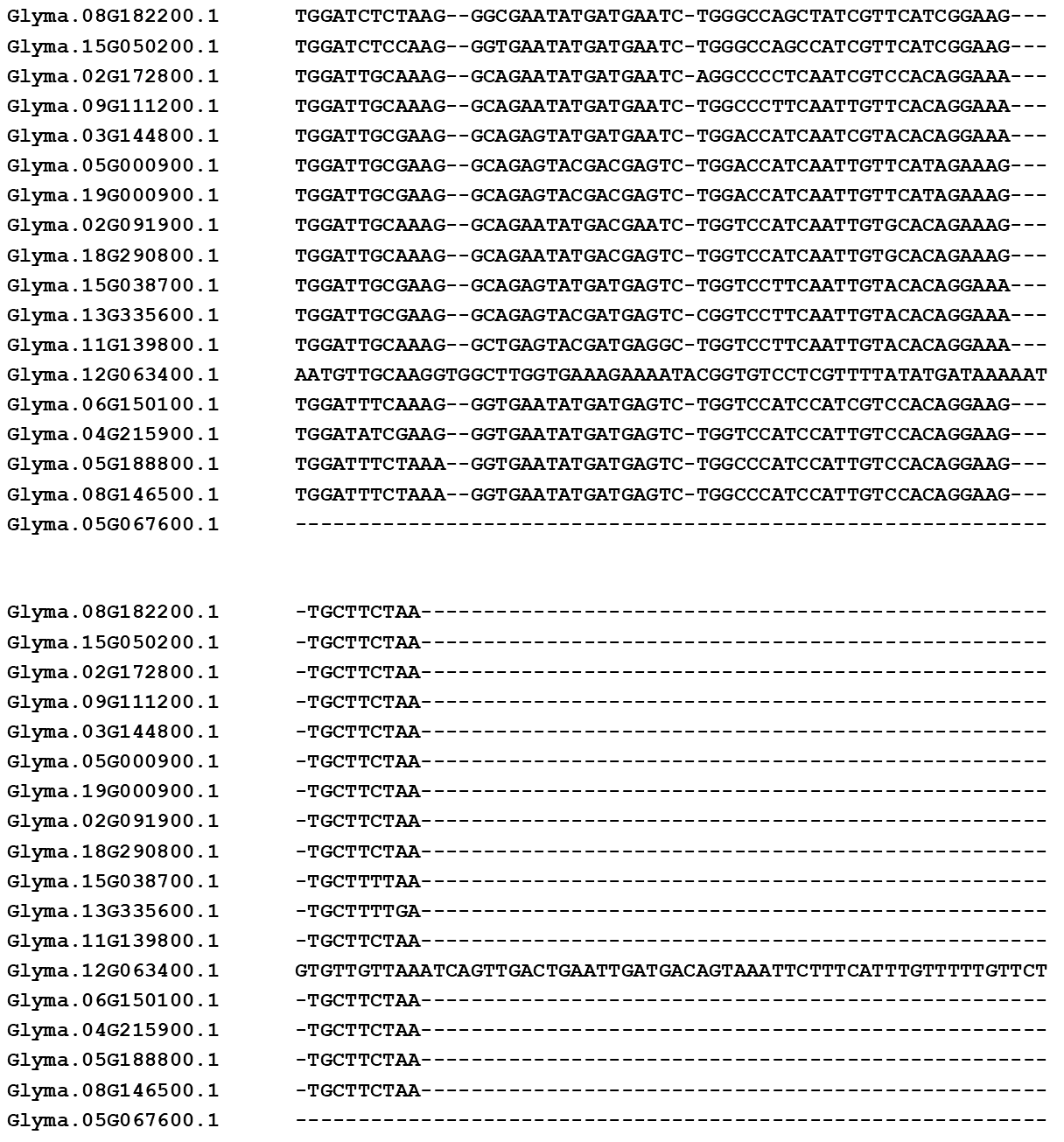
**

**Figure S26. (Cont.)**

**
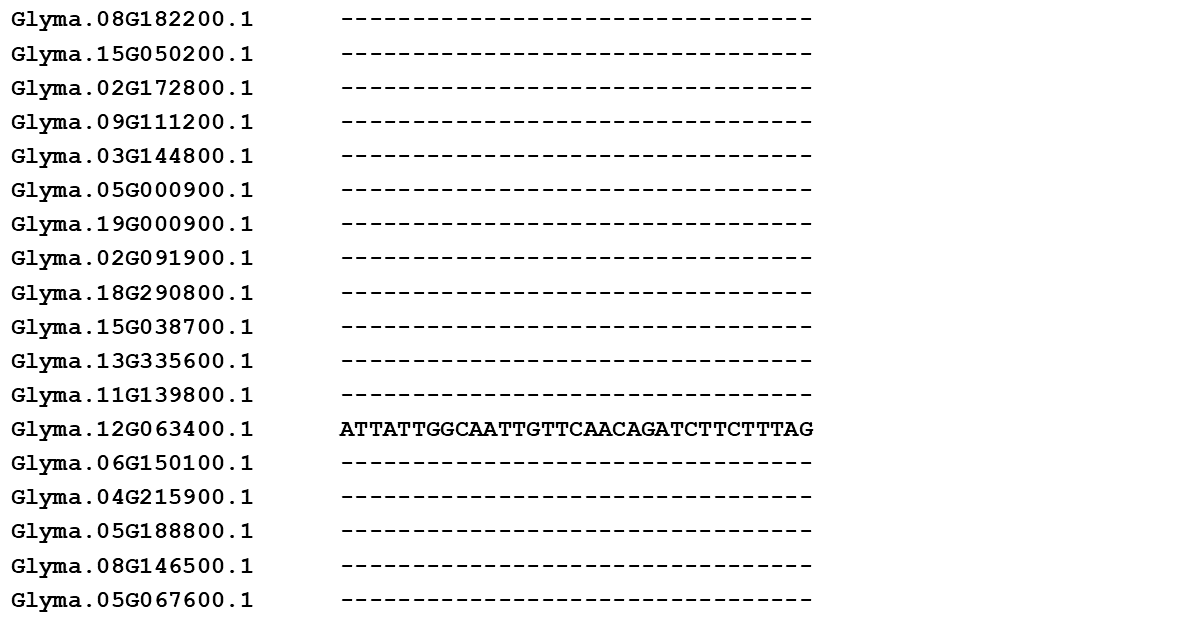
**

**Figure S27.** **Alignment of the** **cDNA sequences of the soybean *cons4* (*Glyma.12G020500*) gene and its homologous sequences in soybean for qPCR primer design.**

**
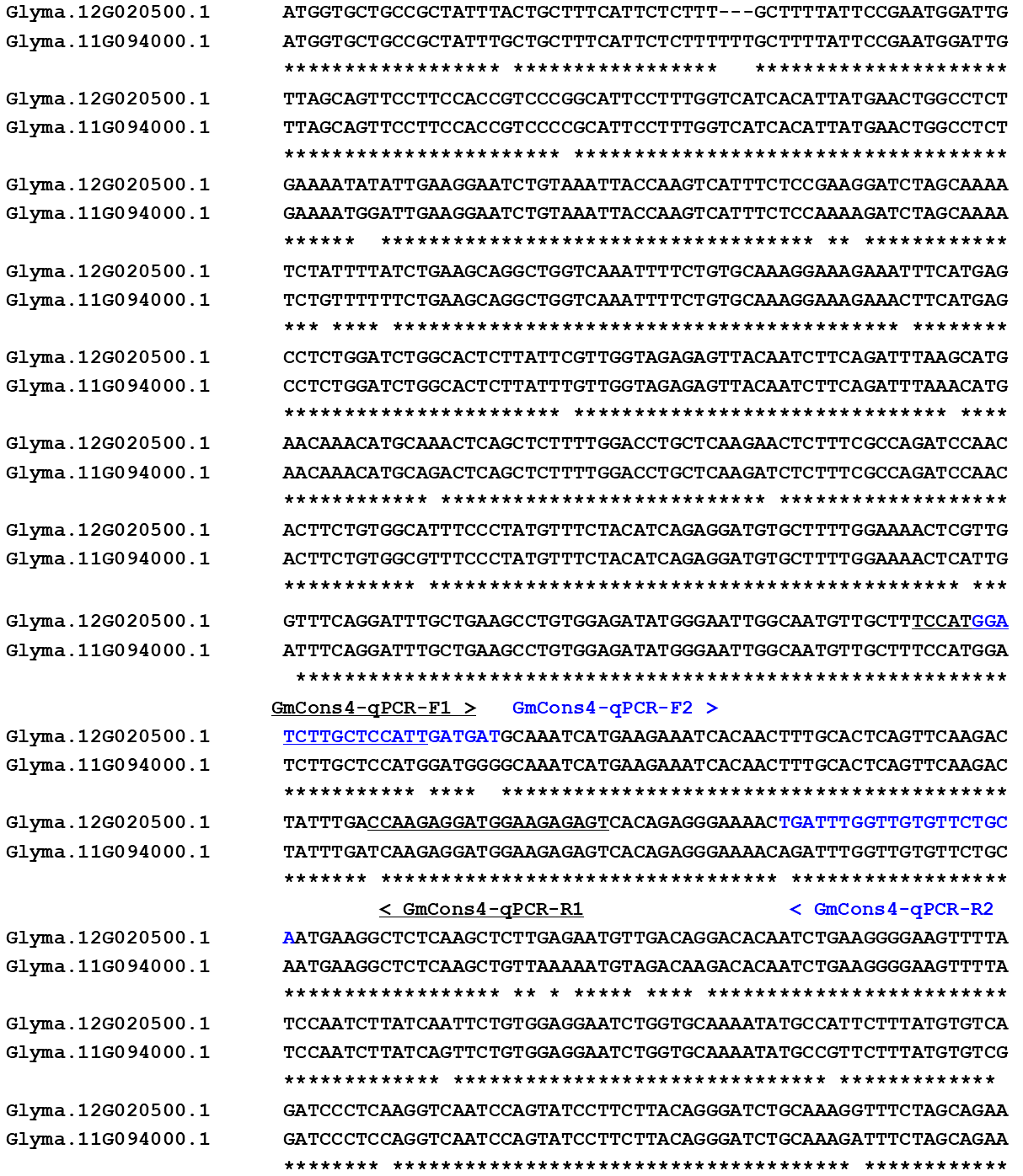
**

**Figure S27. (Cont.)**

**
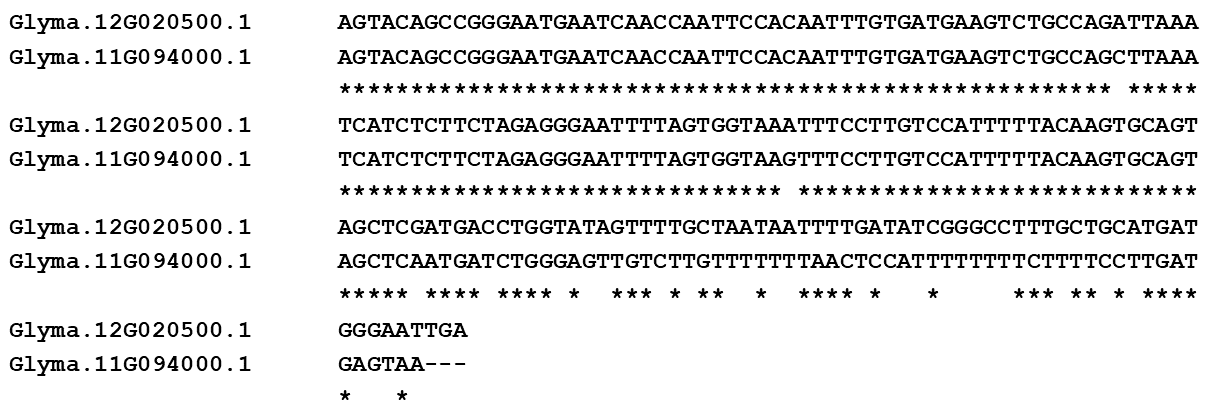
**

**Figure S28.** **Alignment of the** **cDNA sequences of the soybean *cons6* (*Glyma.12G051100*) gene and its homologous sequences in soybean for qPCR primer design.**

**
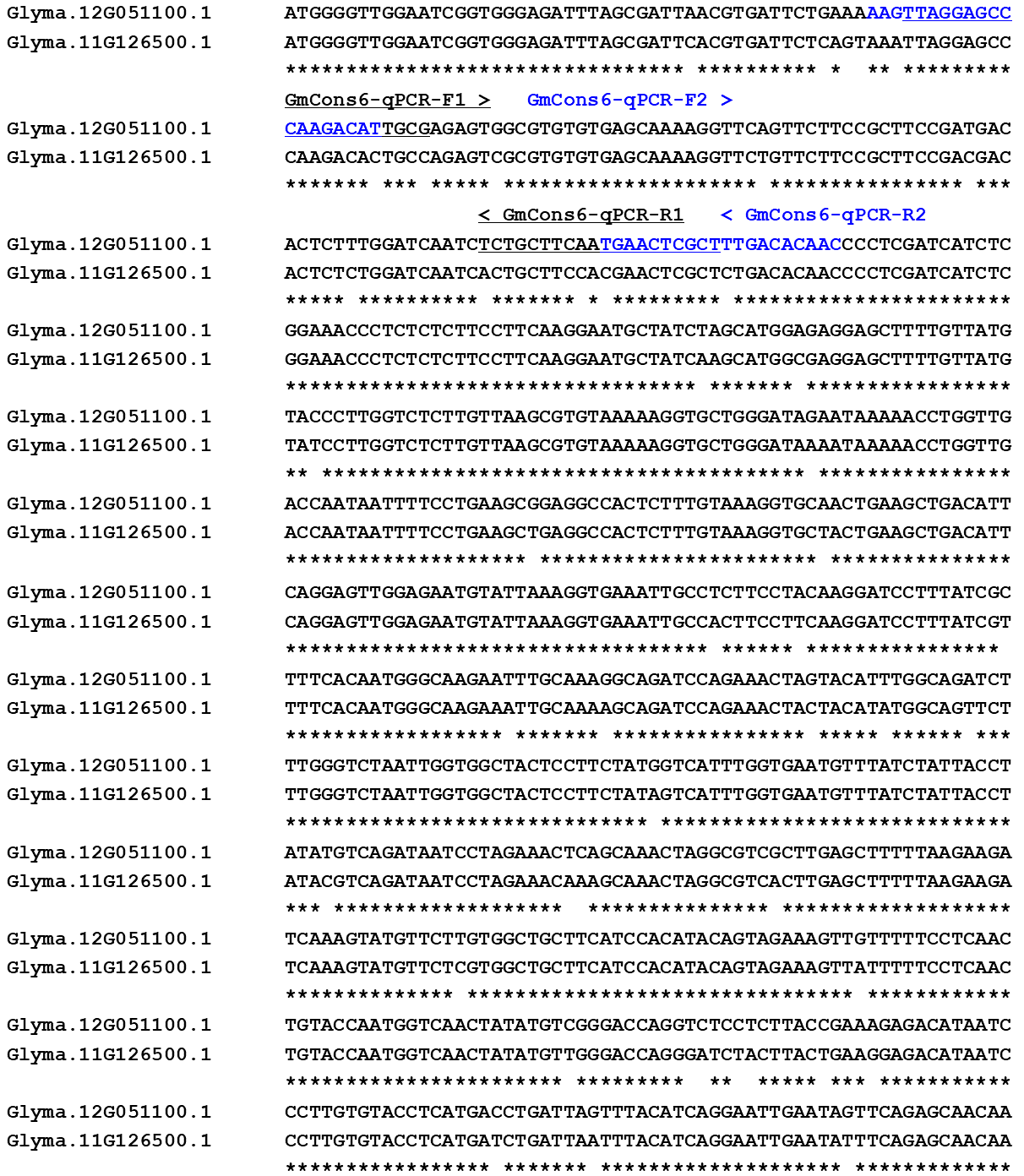
**

**Figure S28. (Cont.)**

**
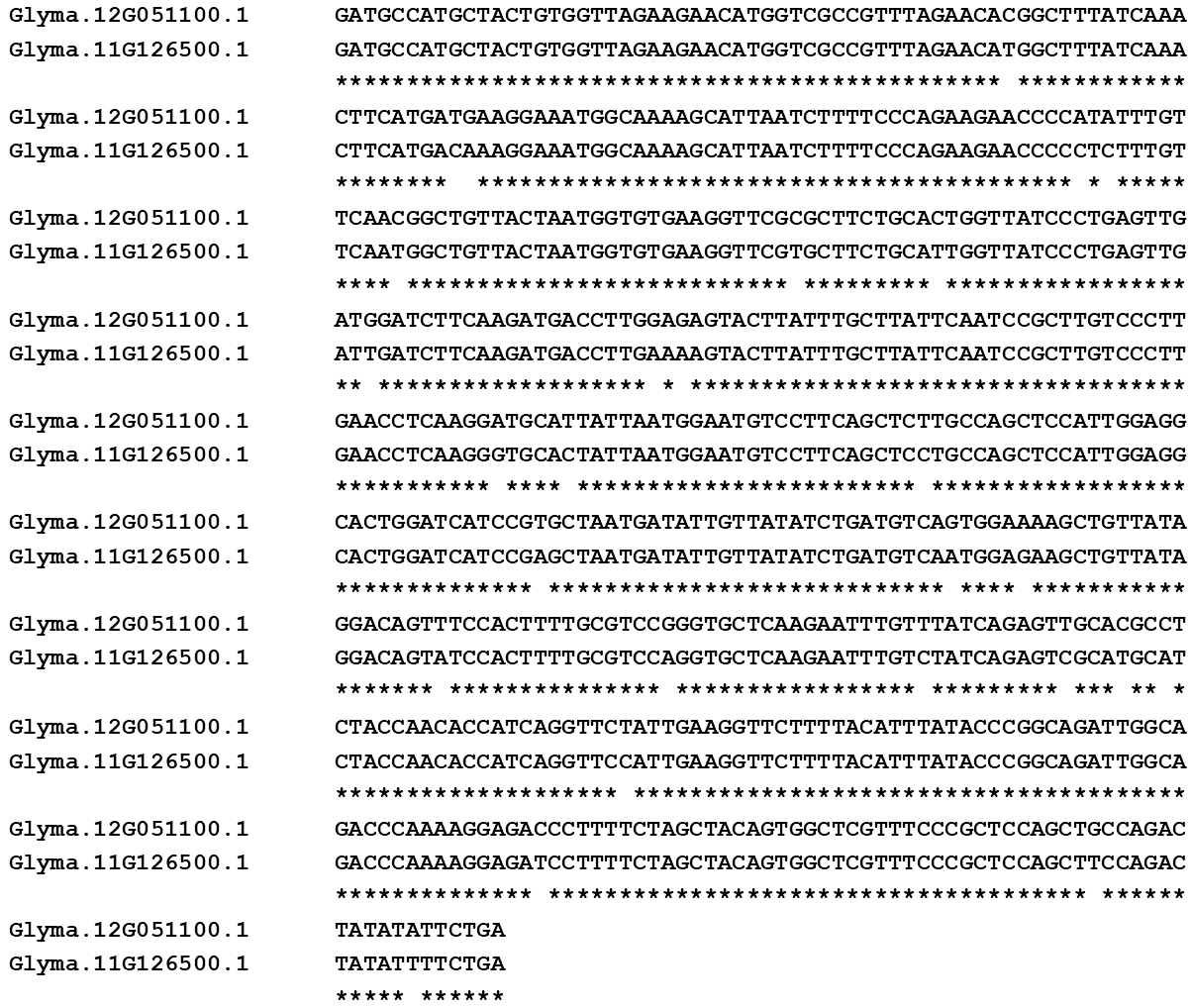
**

**Figure S29.** **Alignment of the** **cDNA sequences of the soybean *Tubulin* (*Glyma.08G014200*) gene and its homologous sequences in soybean for qPCR primer design.**

**
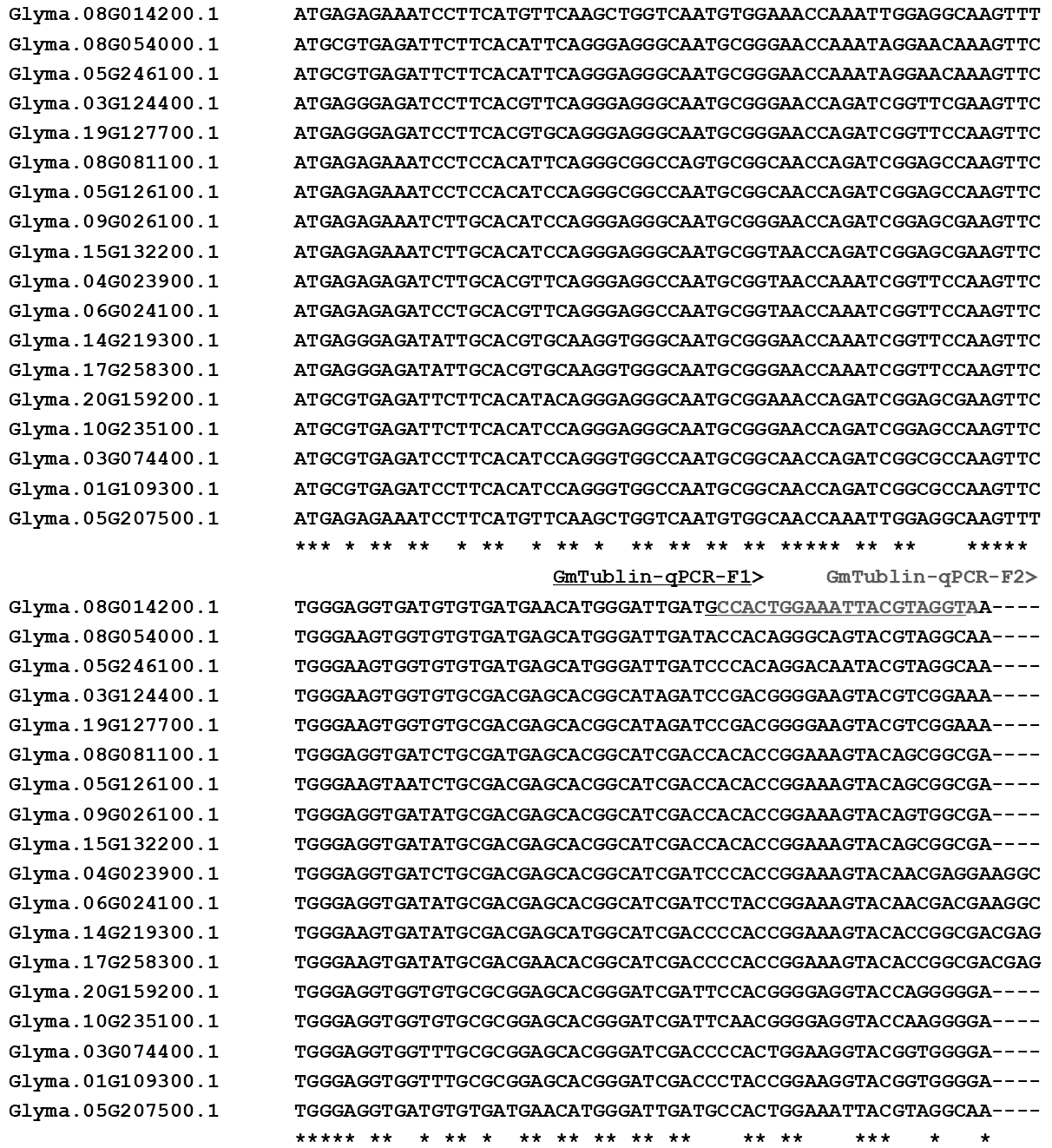
**

**Figure S29. (Cont.)**

**
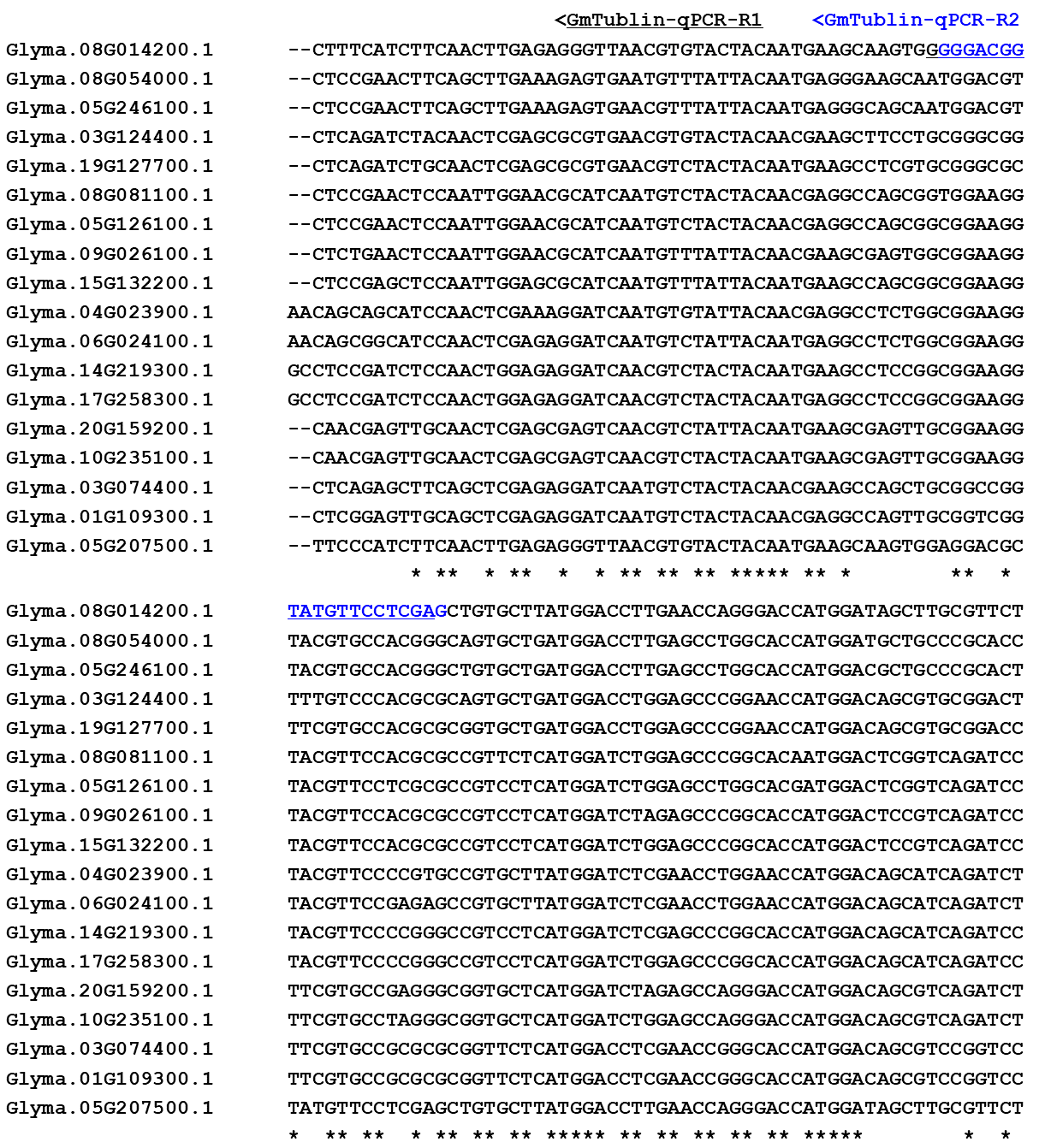
**

**Figure S29. (Cont.)**

**
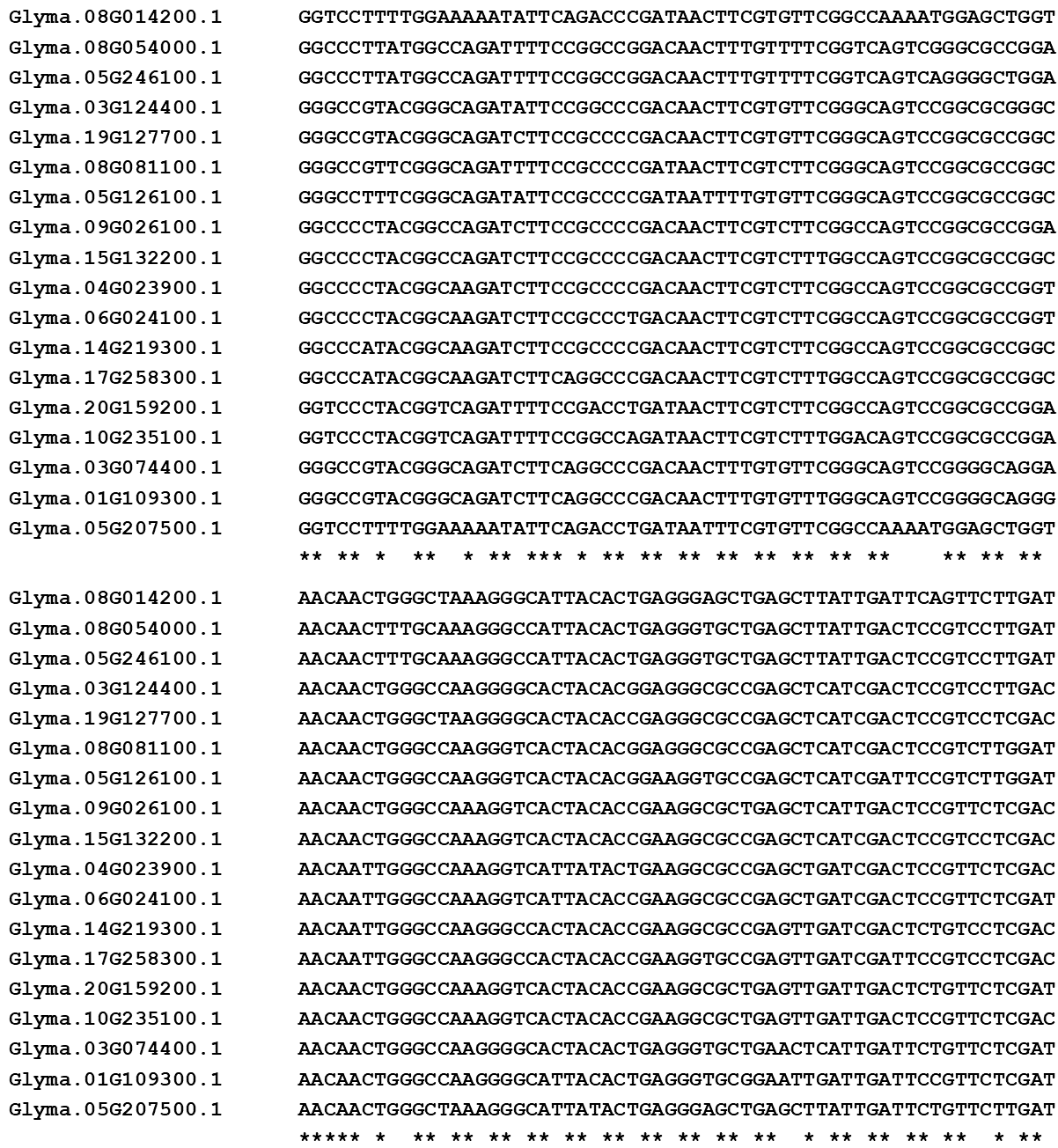
**

**Figure S29. (Cont.)**

**
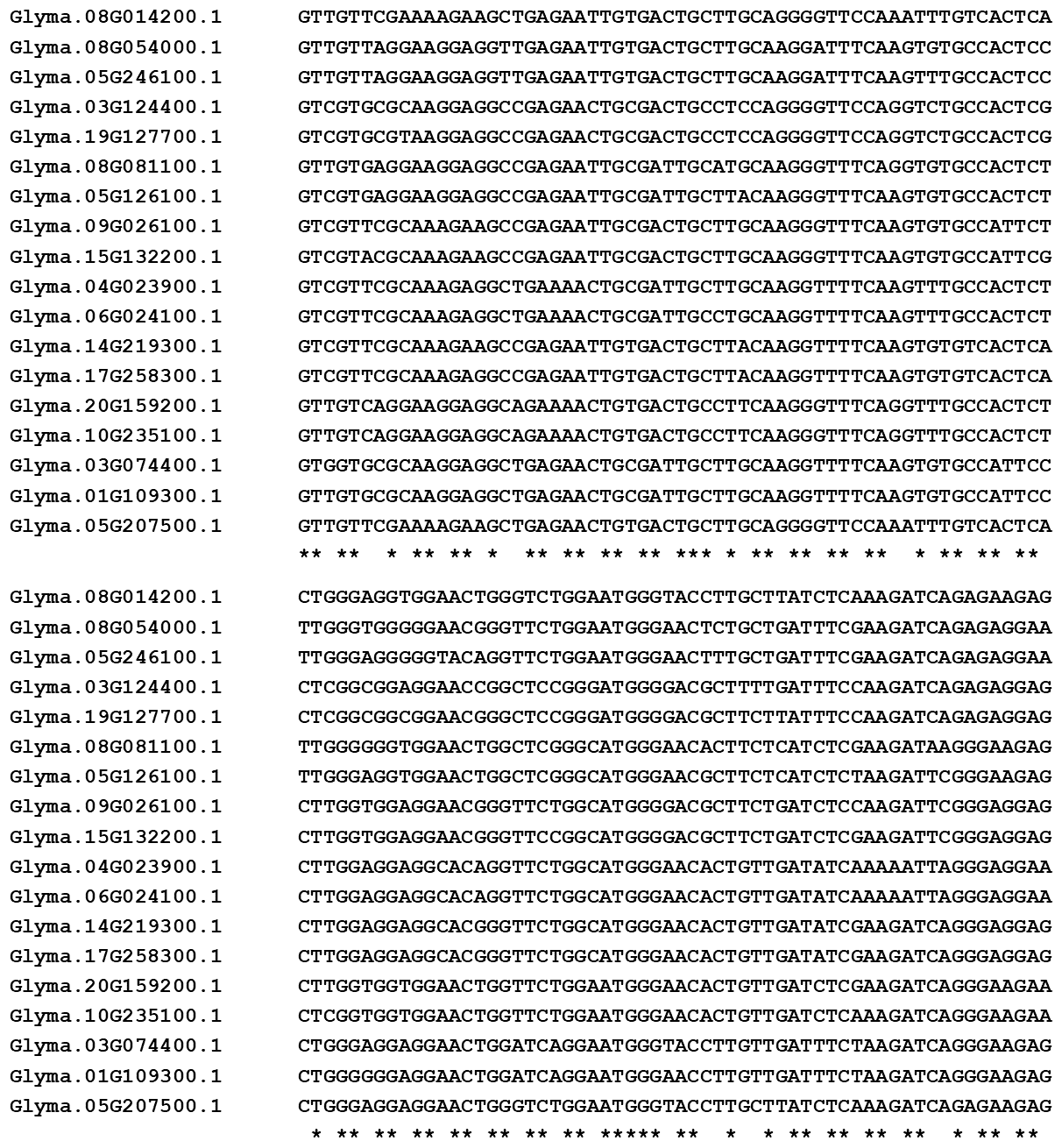
**

**Figure S29. (Cont.)**

**
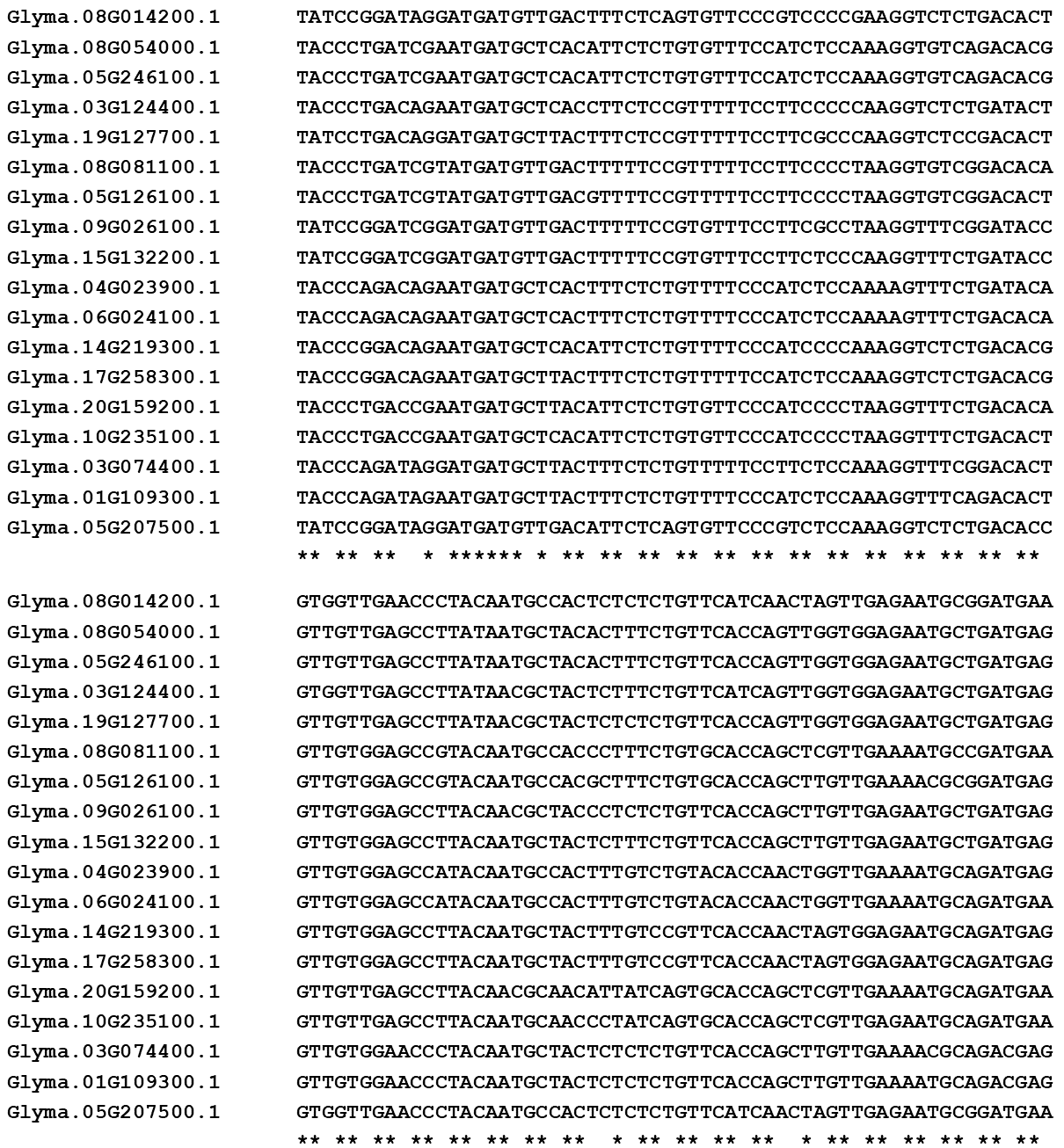
**

**Figure S29. (Cont.)**

**
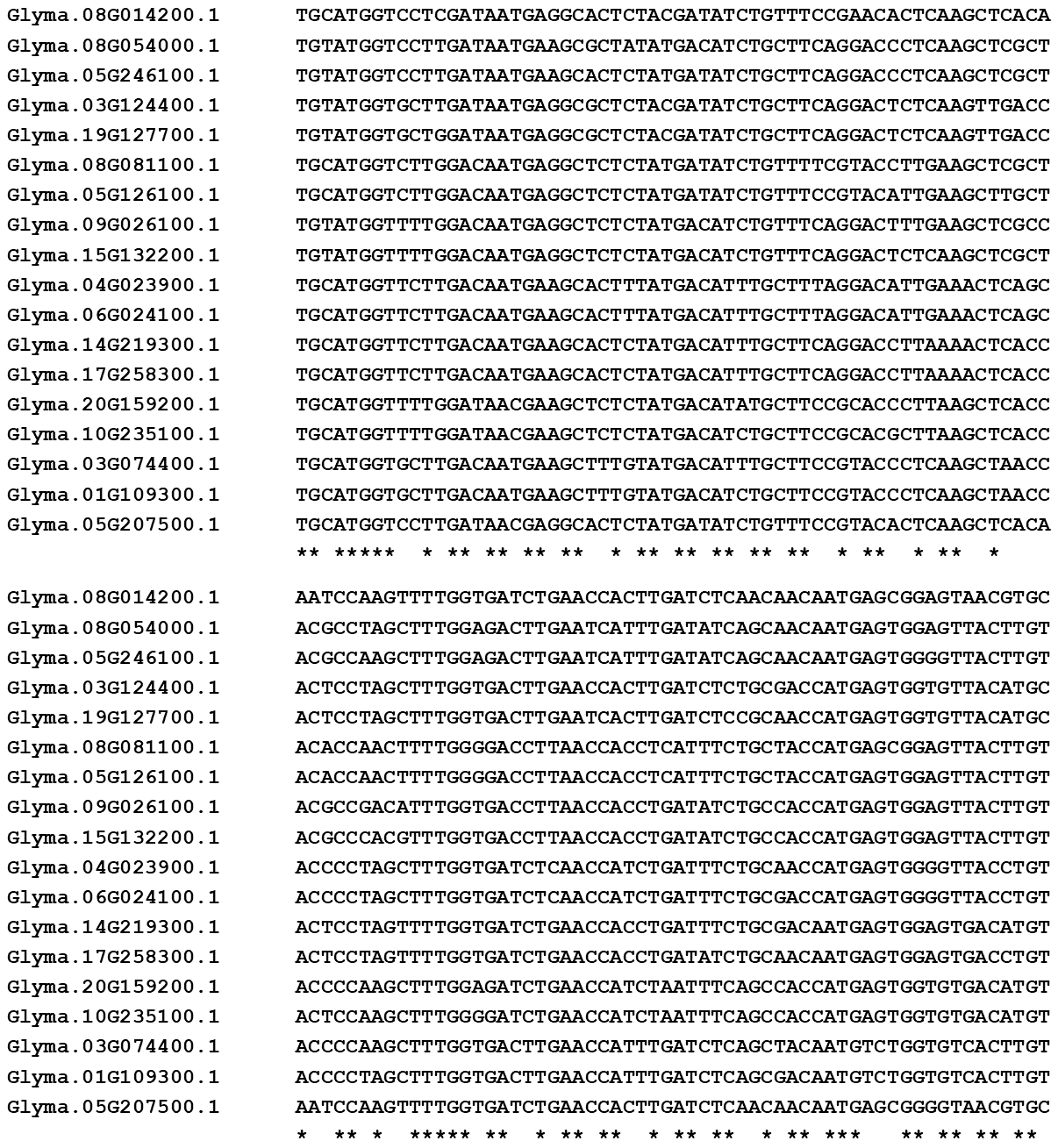
**

**Figure S29. (Cont.)**

**
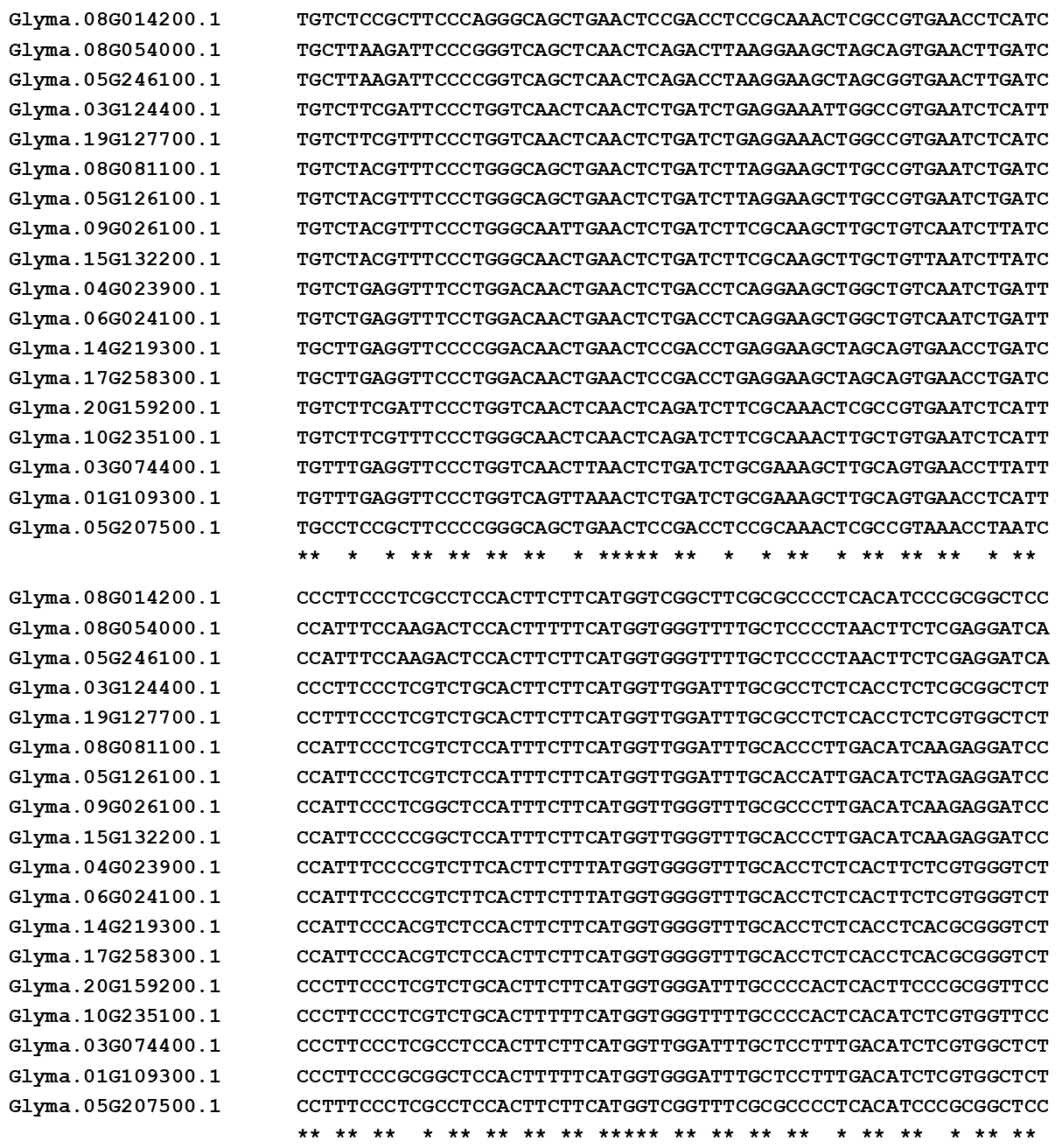
**

**Figure S29. (Cont.)**

**
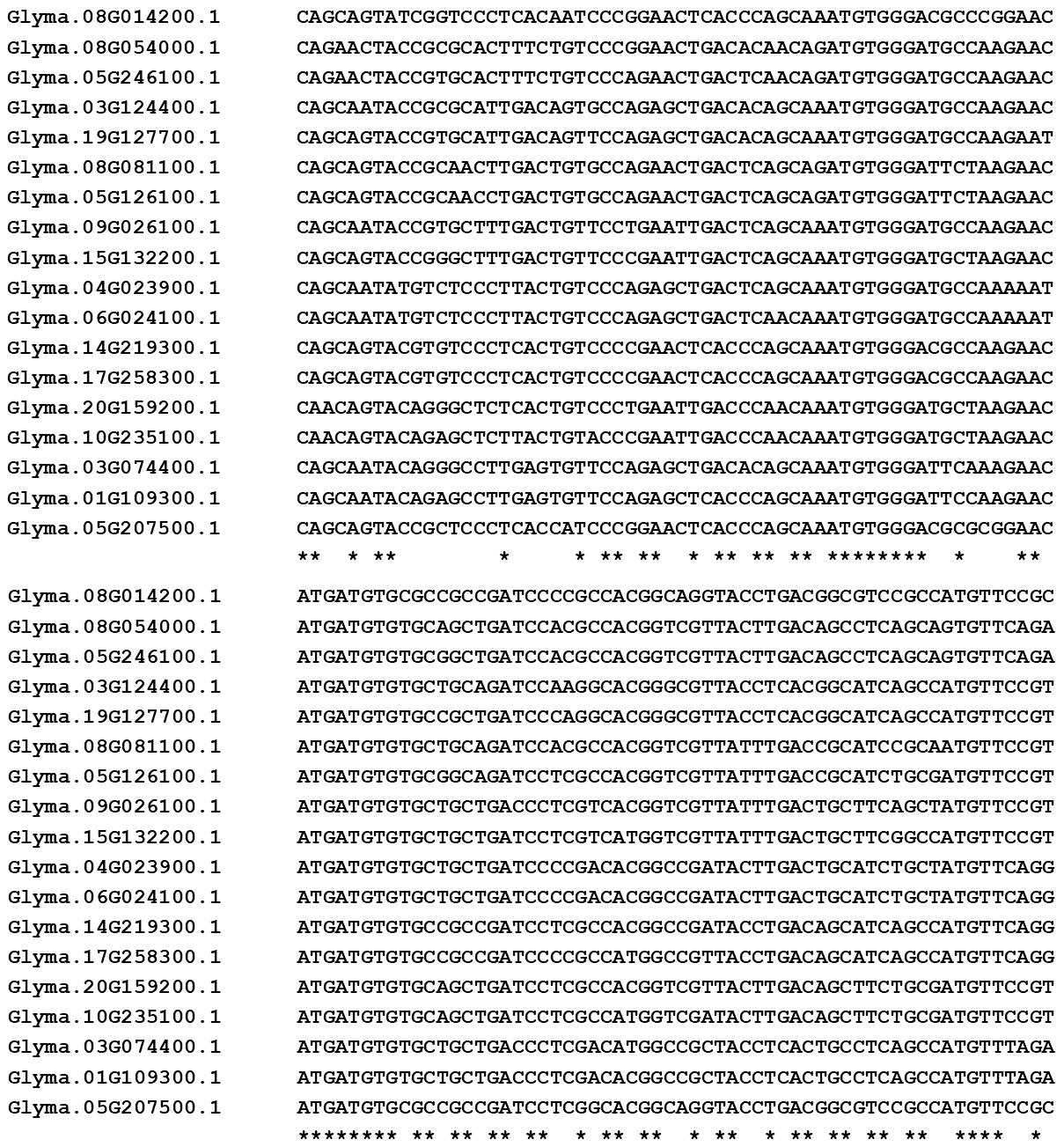
**

**Figure S29. (Cont.)**

**
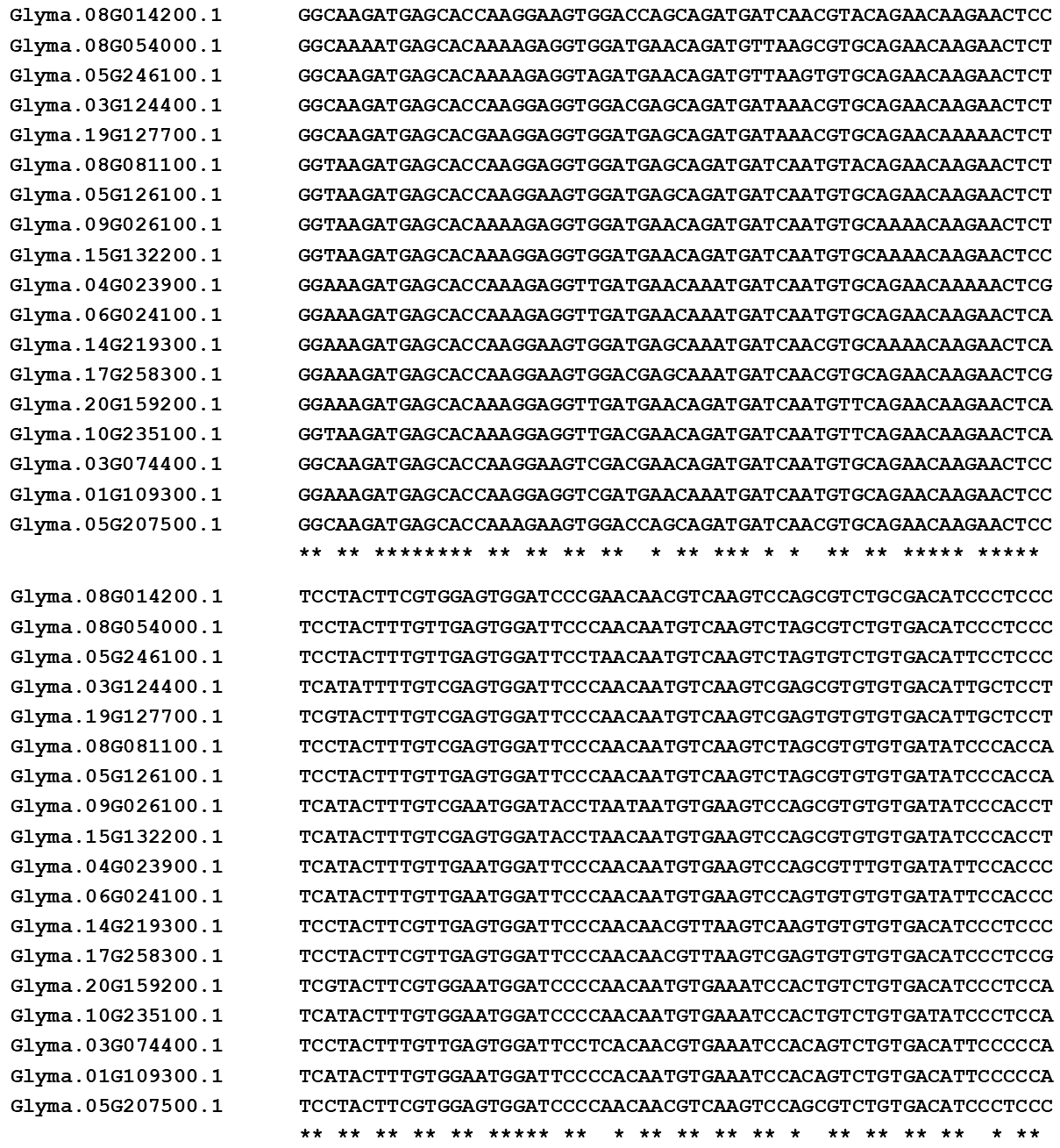
**

**Figure S29. (Cont.)**

**
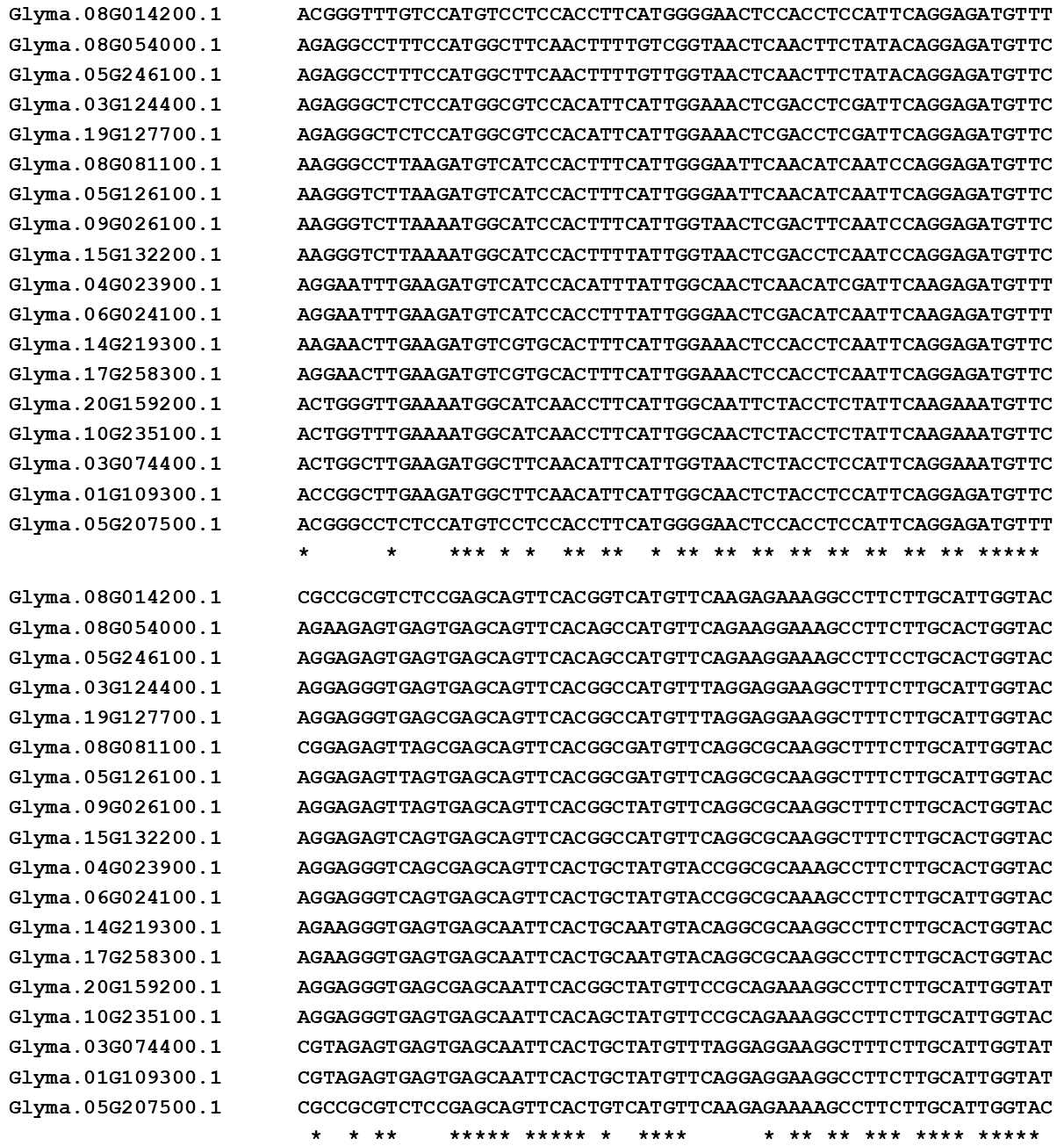
**

**Figure S29. (Cont.)**

**
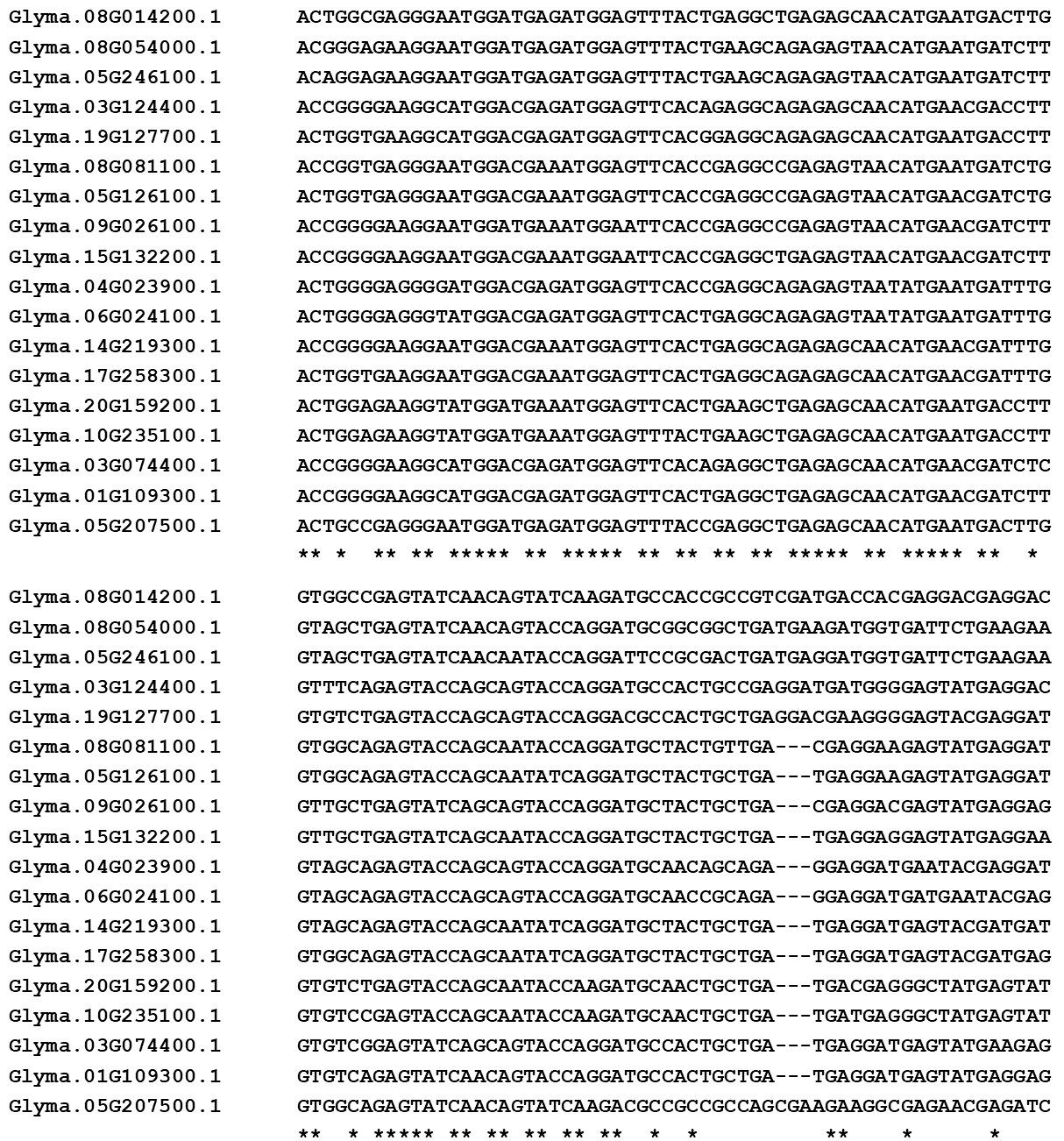
**

**Figure S29. (Cont.)**

**
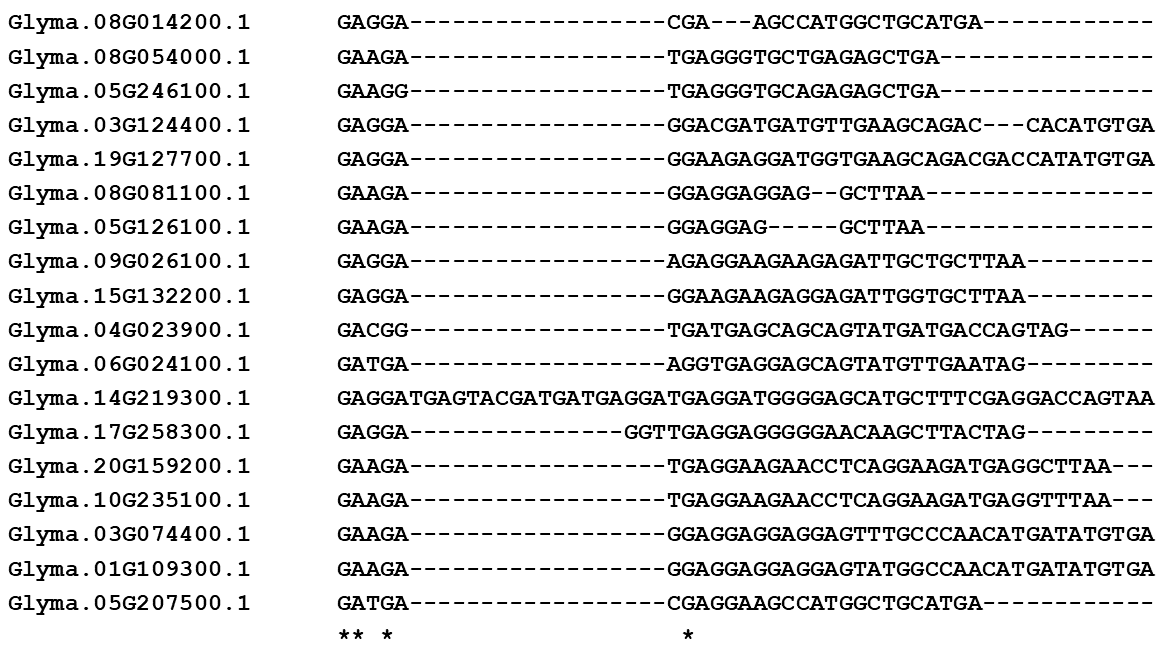
**

**Figure S30.** **Alignment of the** **cDNA sequences of the soybean *FtsZ2-1* (*Glyma.19G194800*) and its homologous sequences in soybean for qPCR primer design.**

**
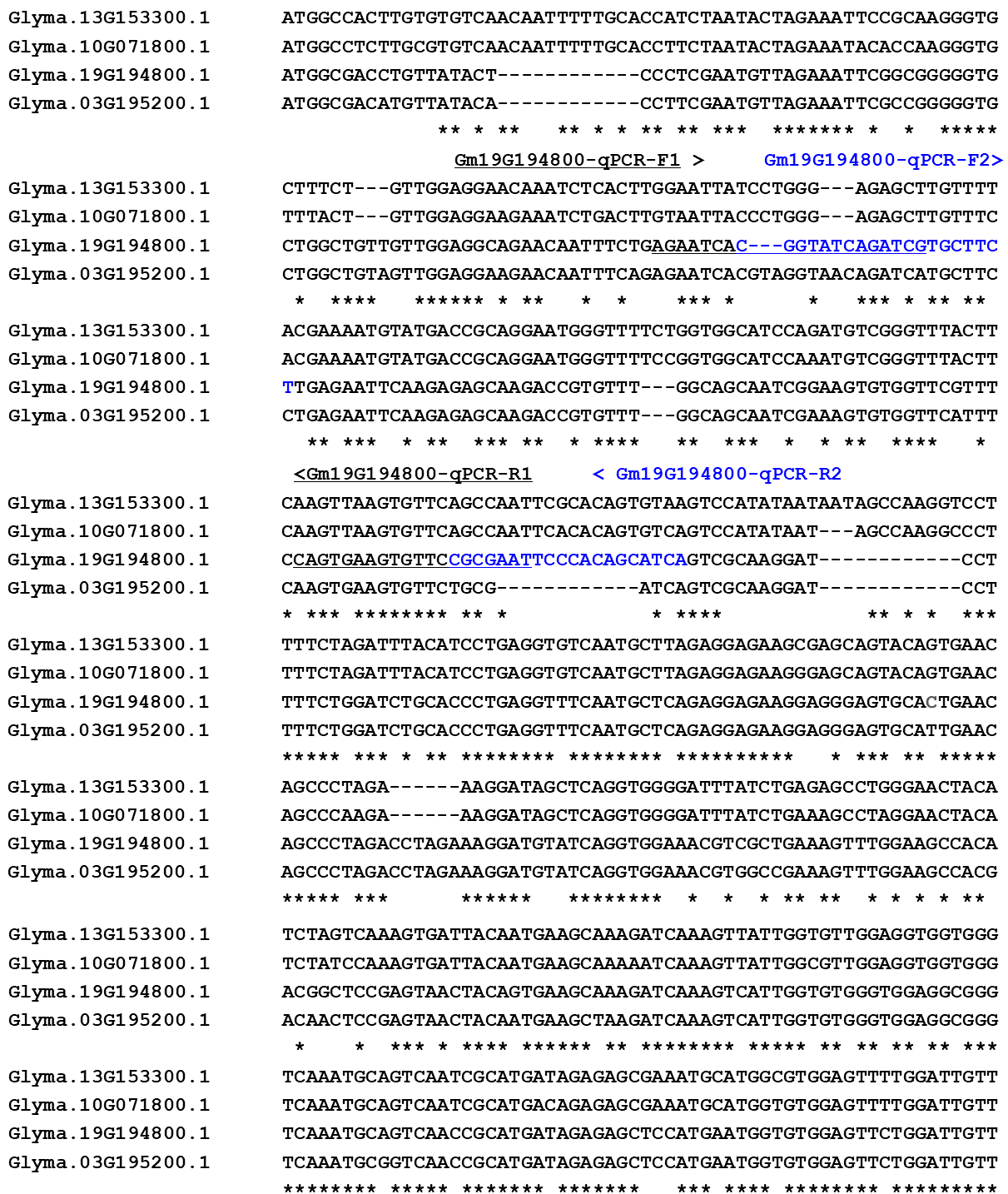
**

**Figure S30. (Cont.)**

**
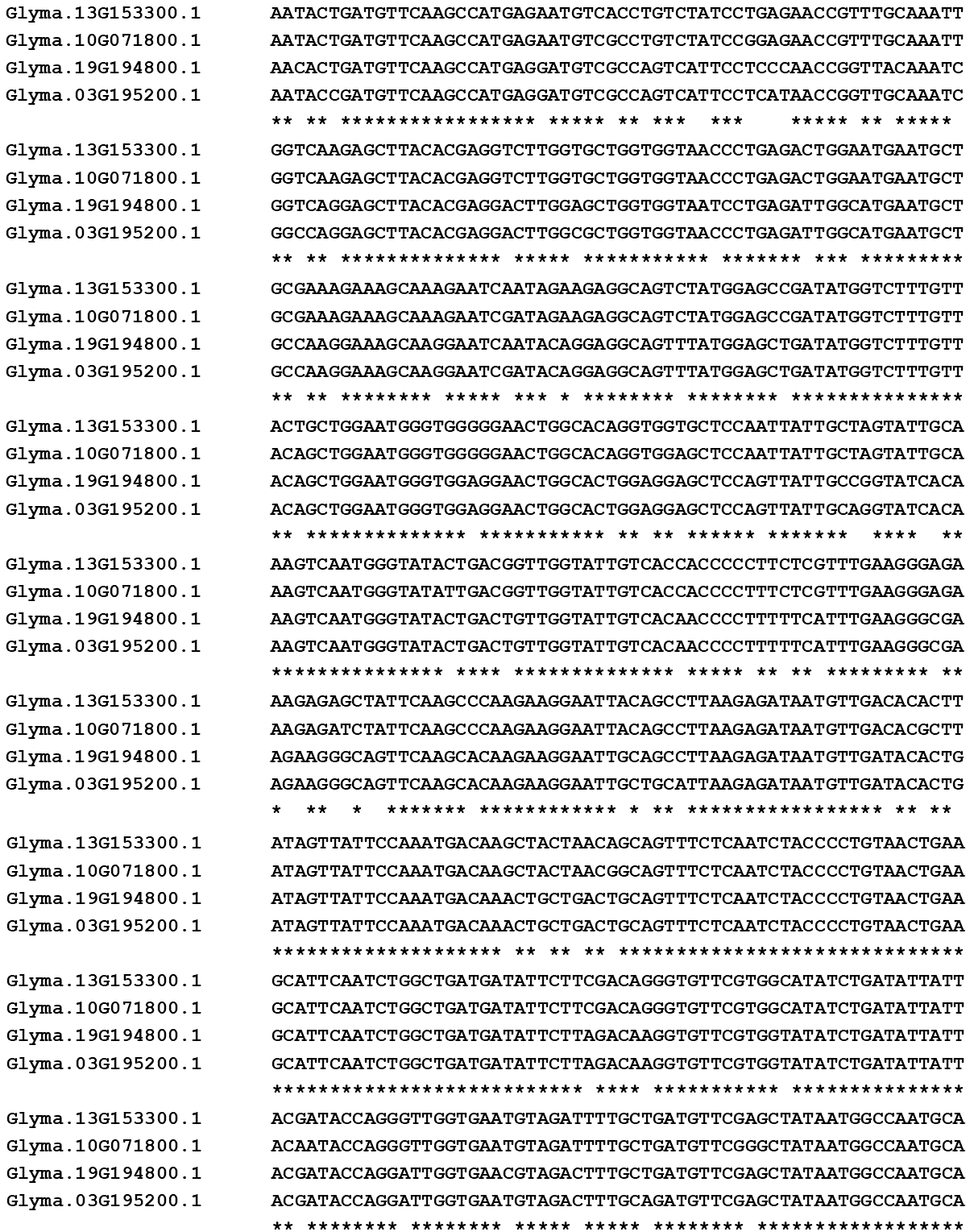
**

**Figure S30. (Cont.)**

**
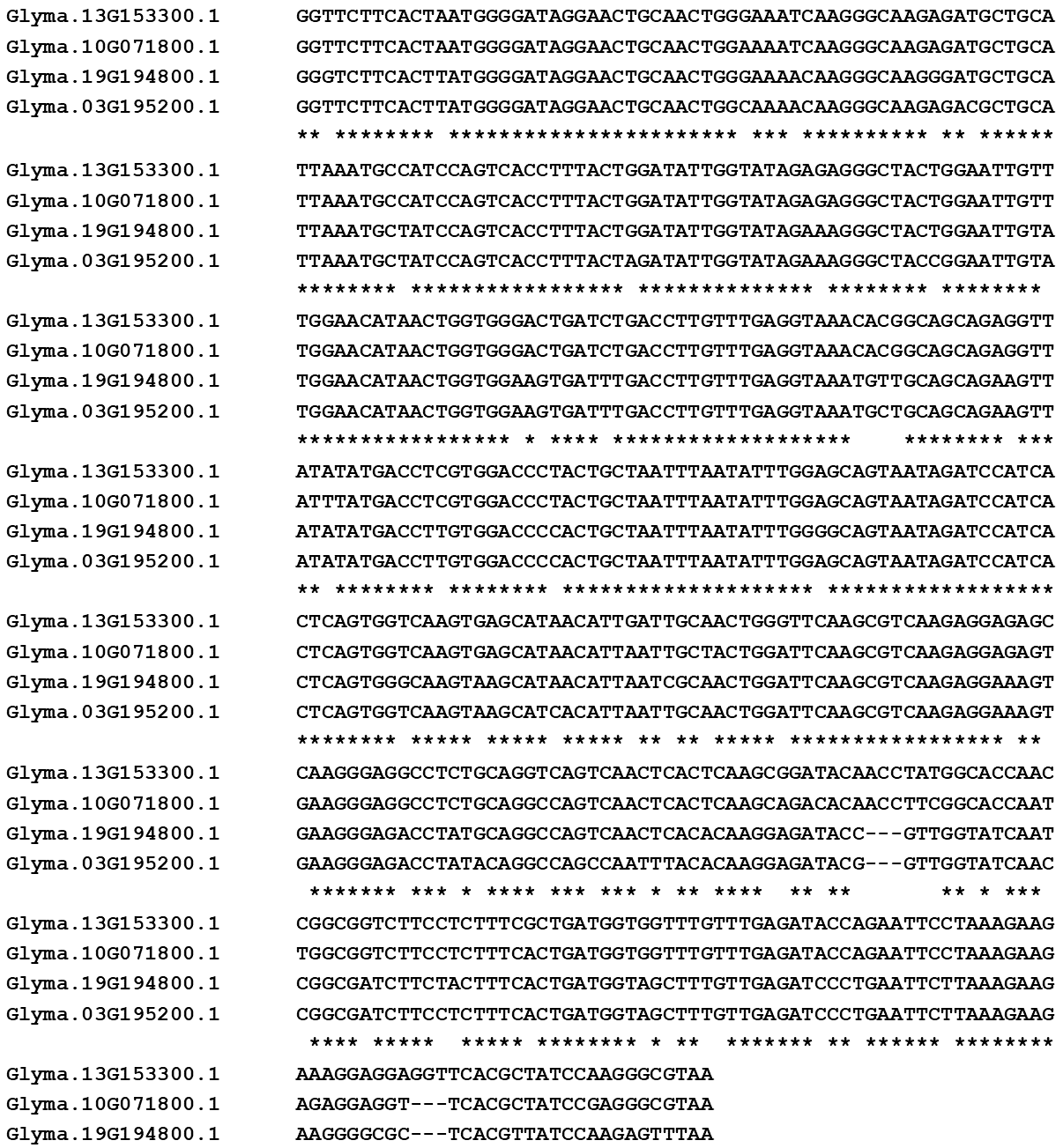
**

**Figure S31.** **Alignment of the** **cDNA sequences of the soybean *TUBB* (*Glyma.04G023900*) gene and its homologous sequences in soybean for qPCR primer design.**

**
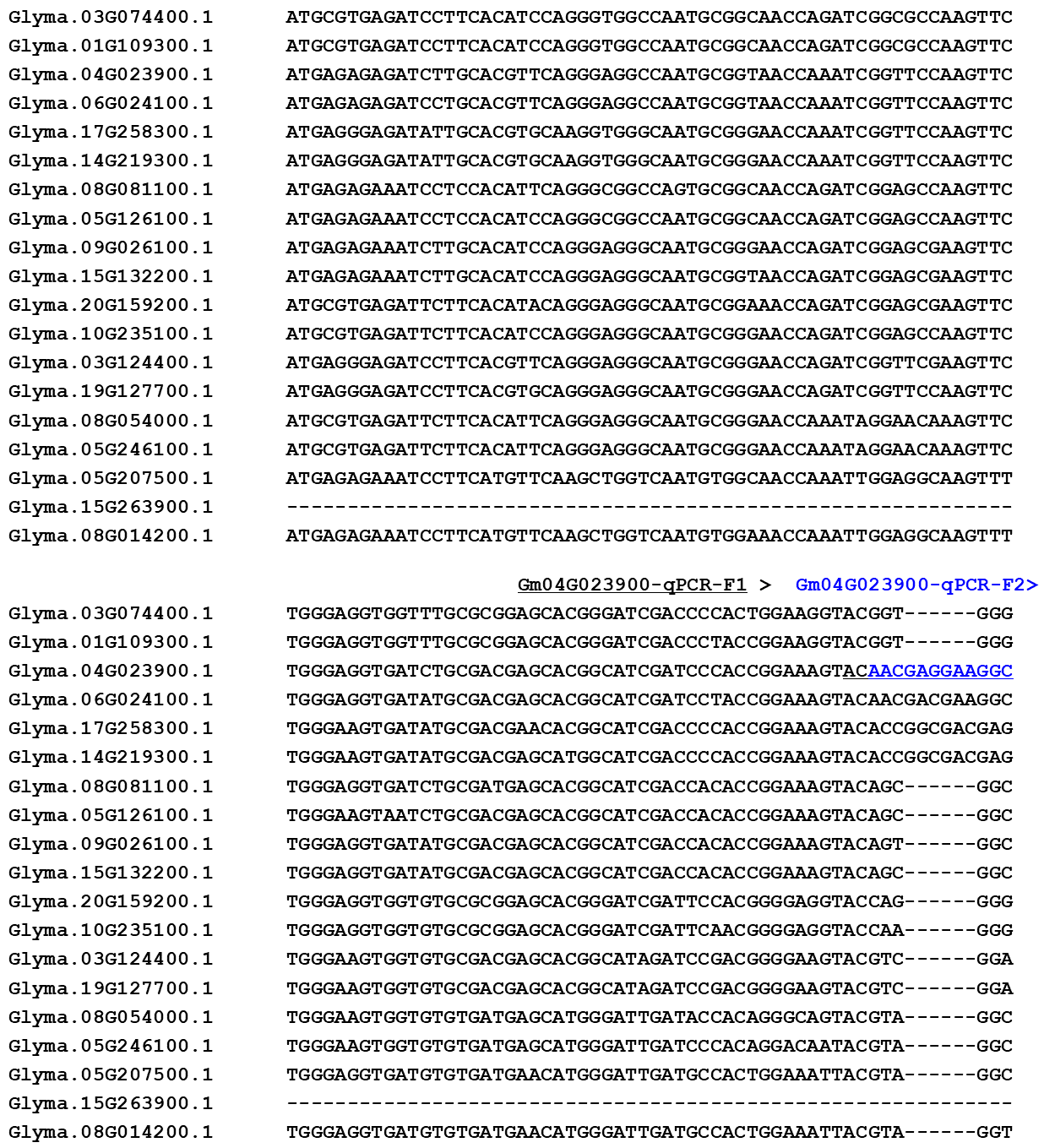
**

**Figure S31. (Cont.)**

**
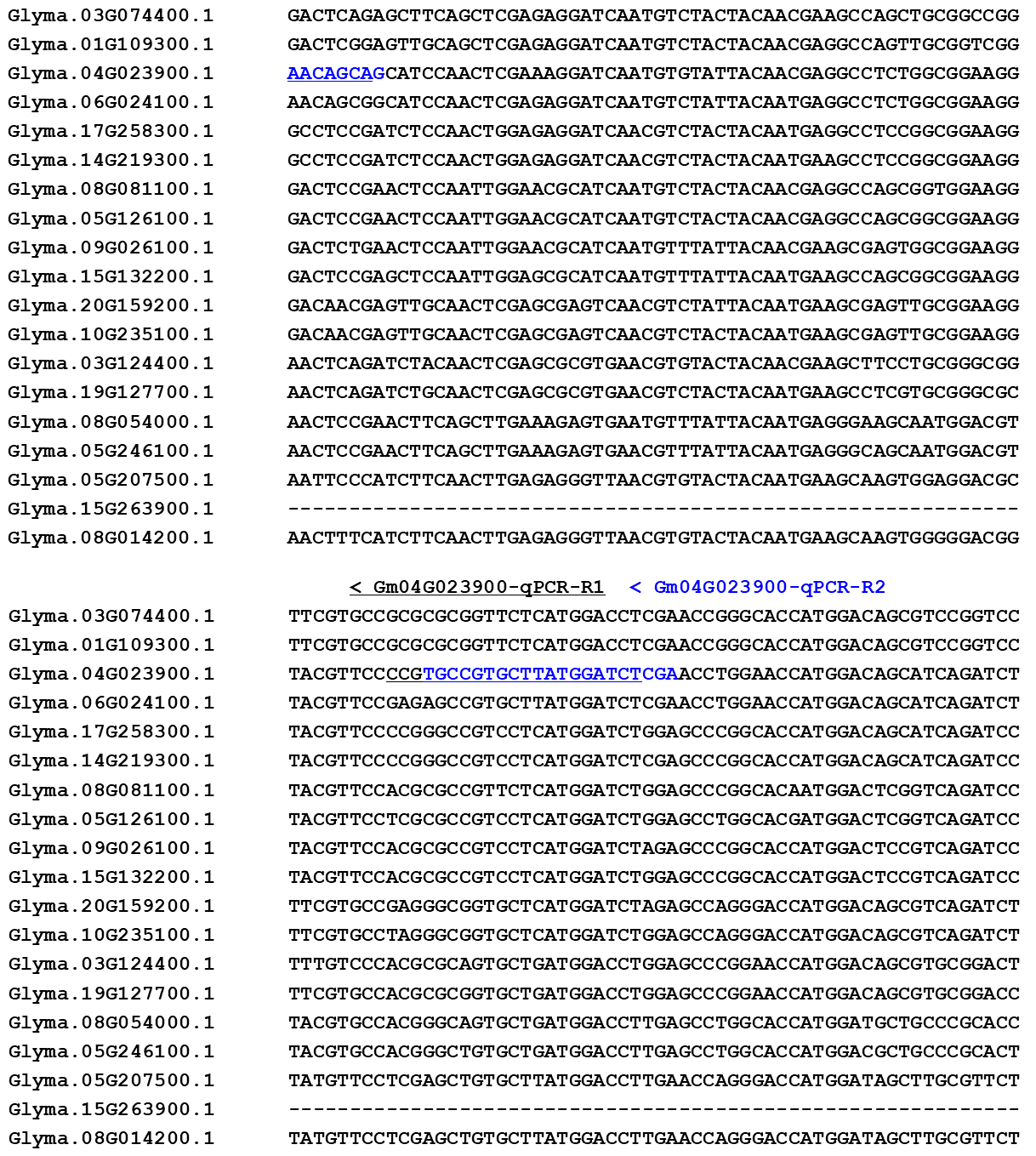
**

**Figure S31. (Cont.)**

**
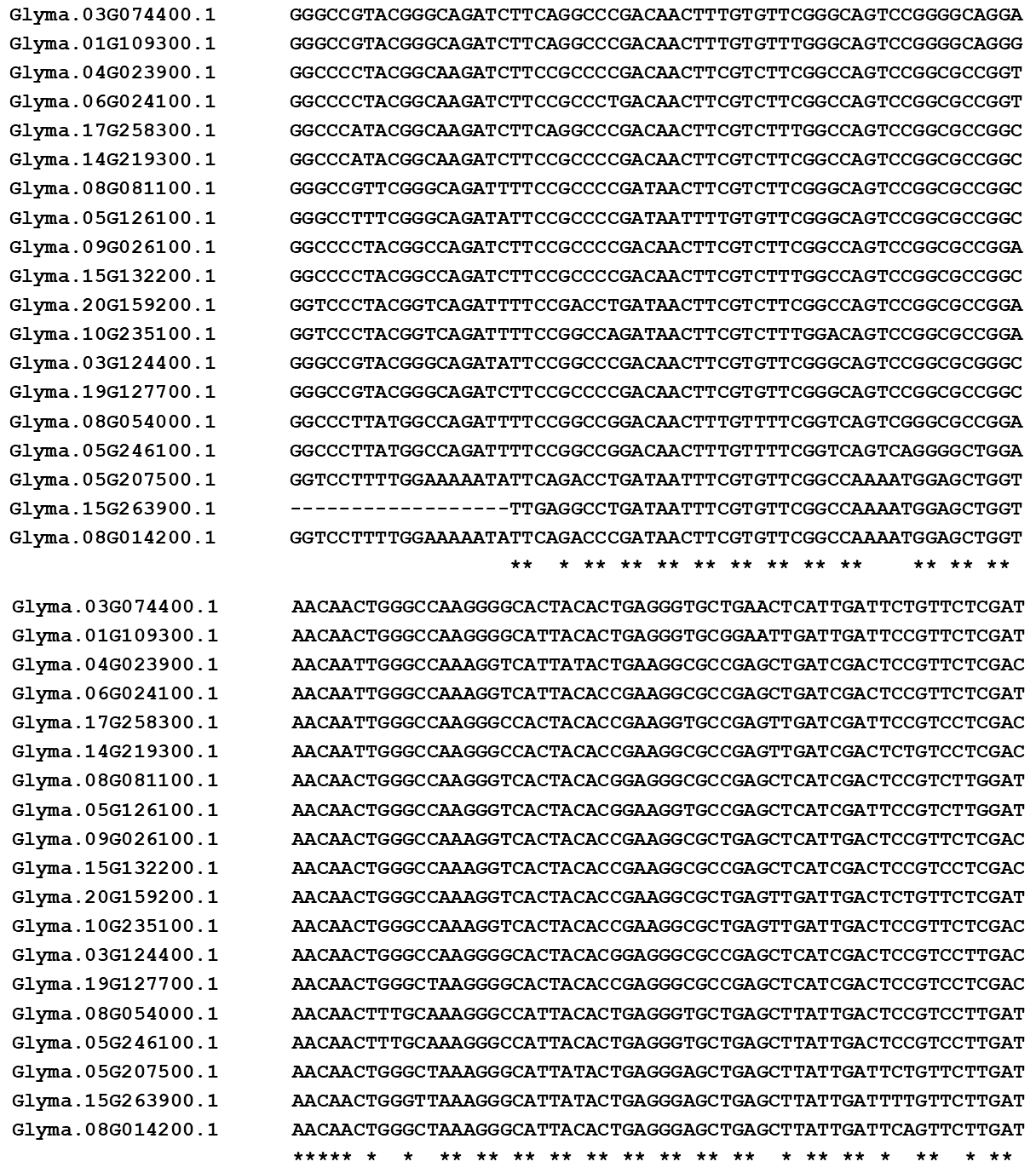
**

**Figure S31. (Cont.)**

**
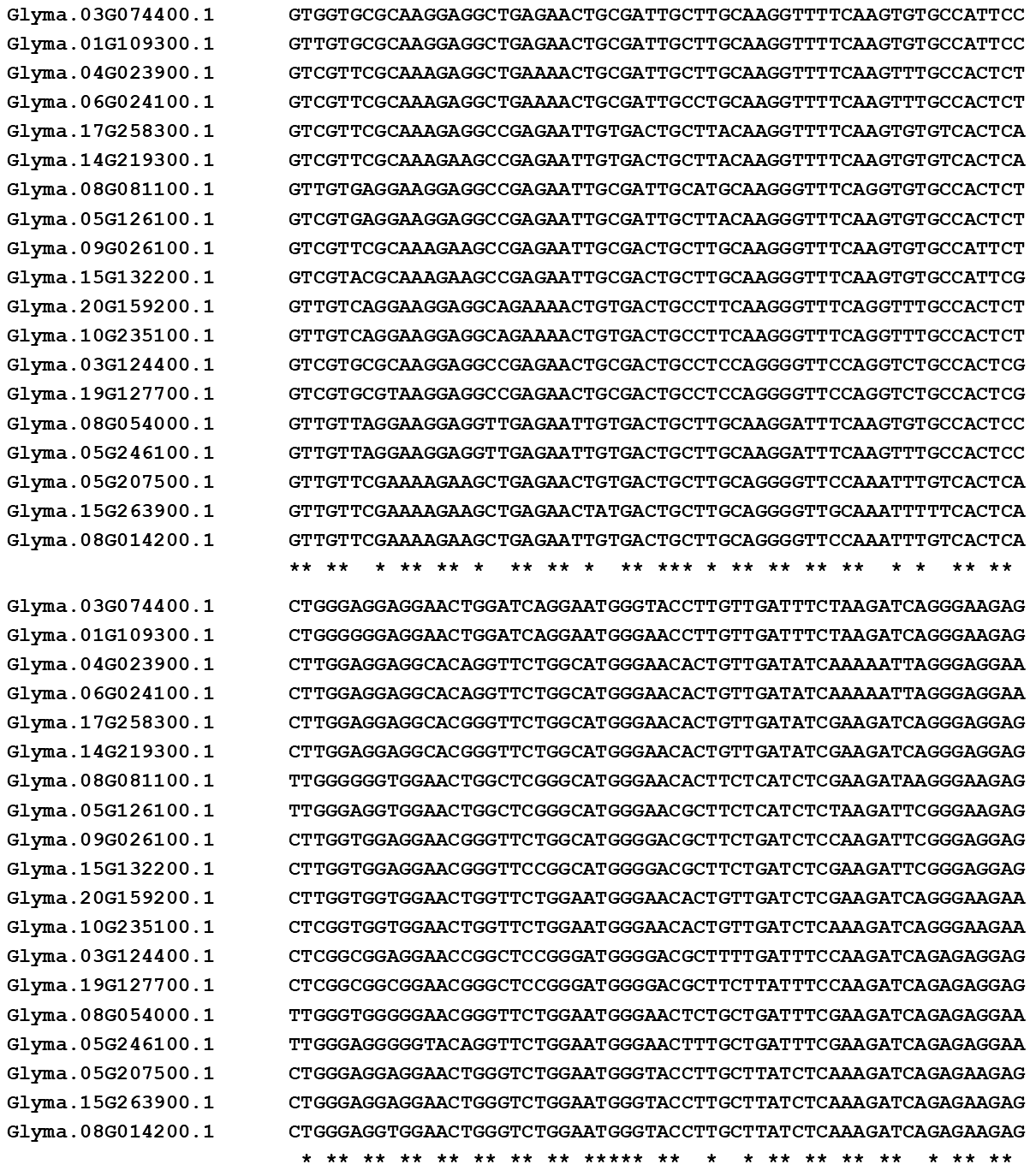
**

**Figure S31. (Cont.)**

**
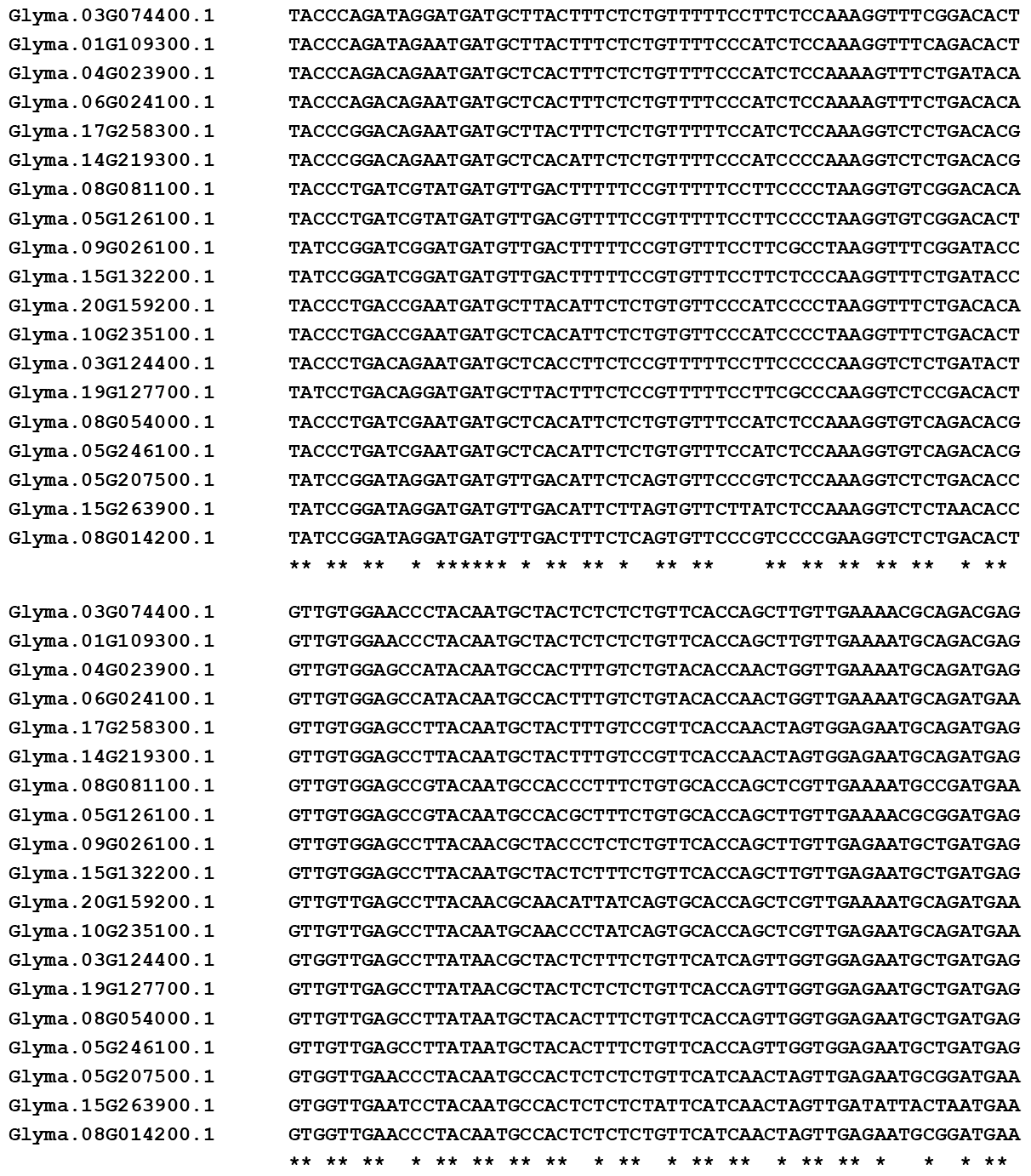
**

**Figure S31. (Cont.)**

**
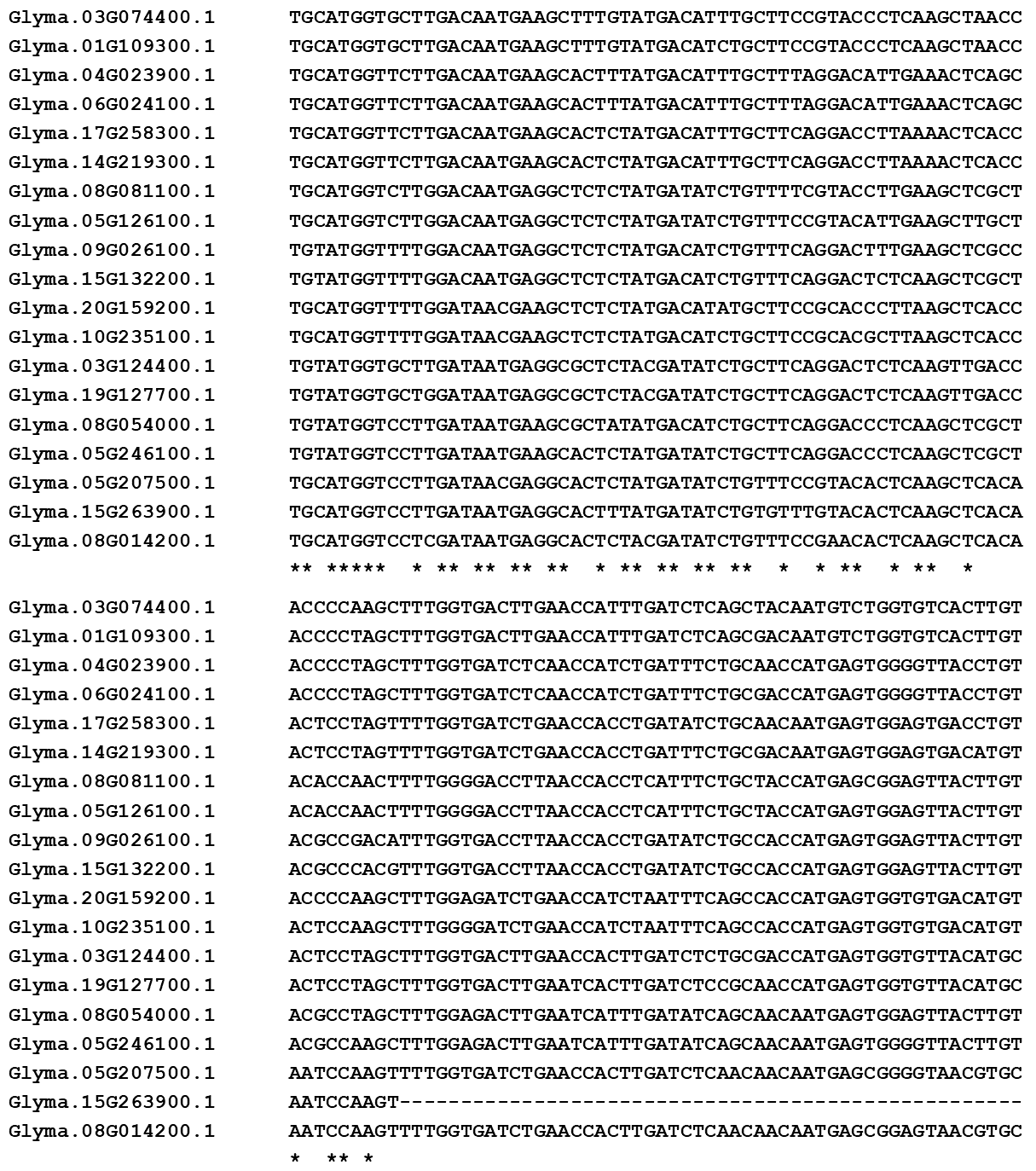
**

**Figure S31. (Cont.)**

**
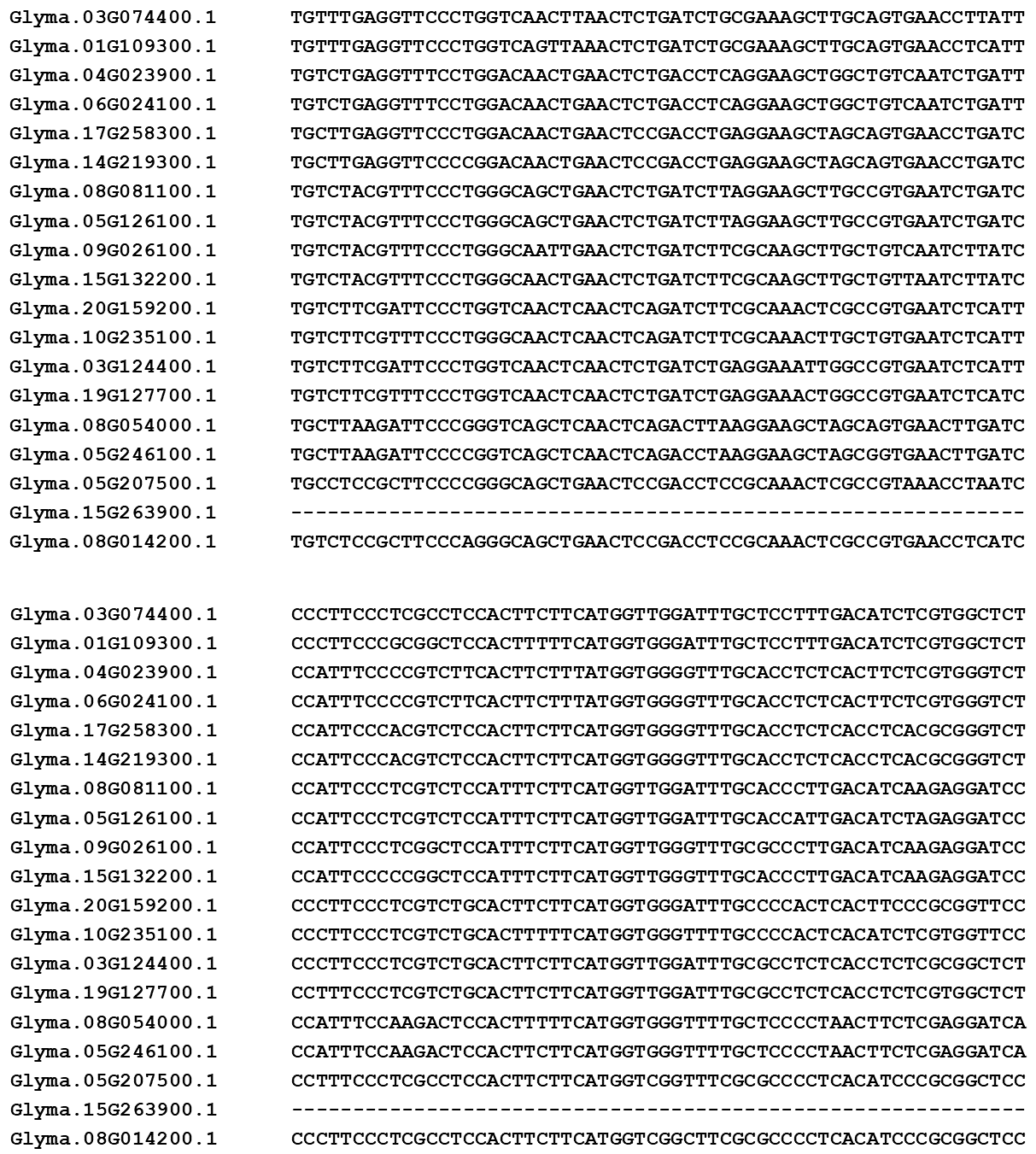
**

**Figure S31. (Cont.)**

**
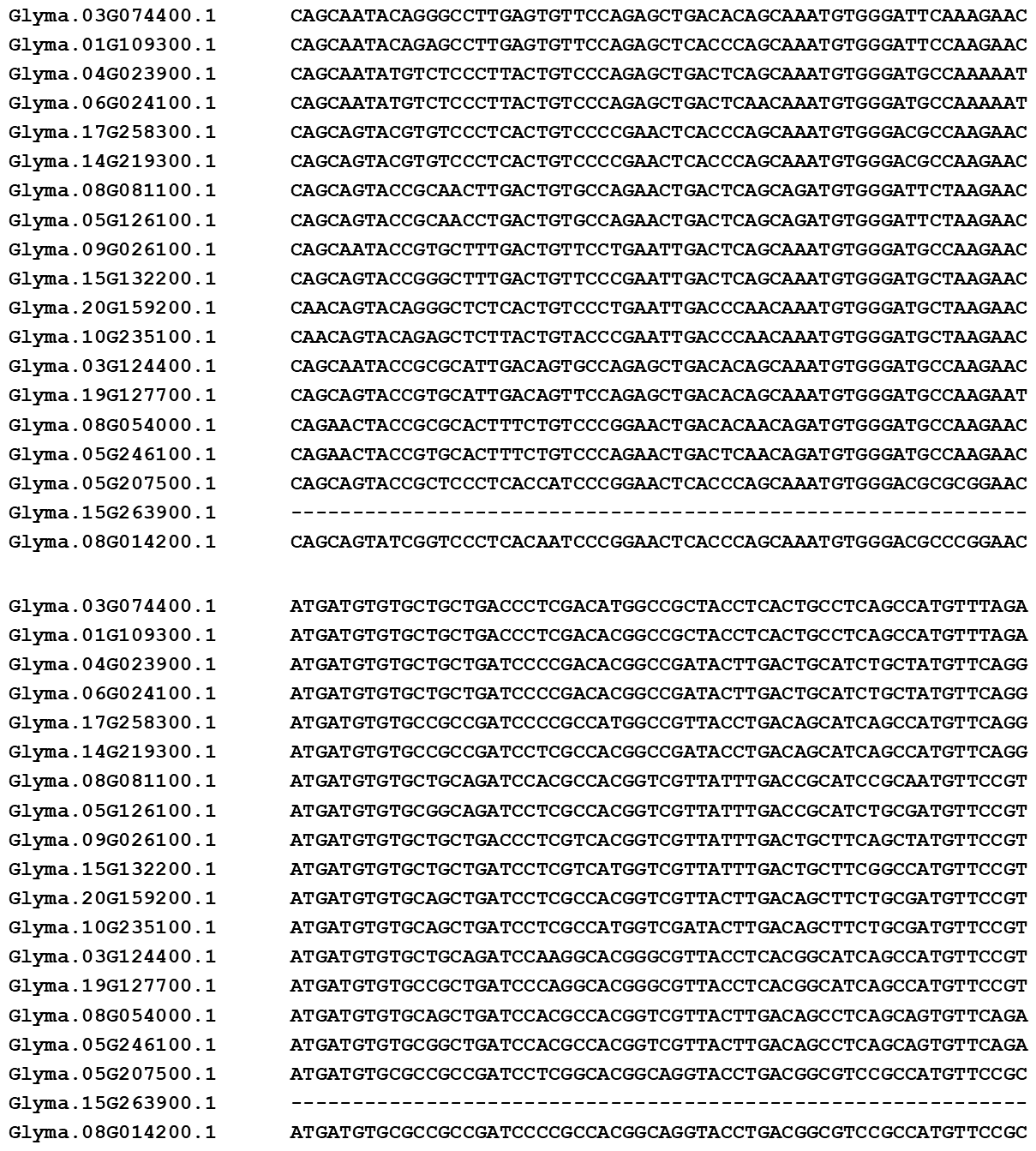
**

**Figure S31. (Cont.)**

**
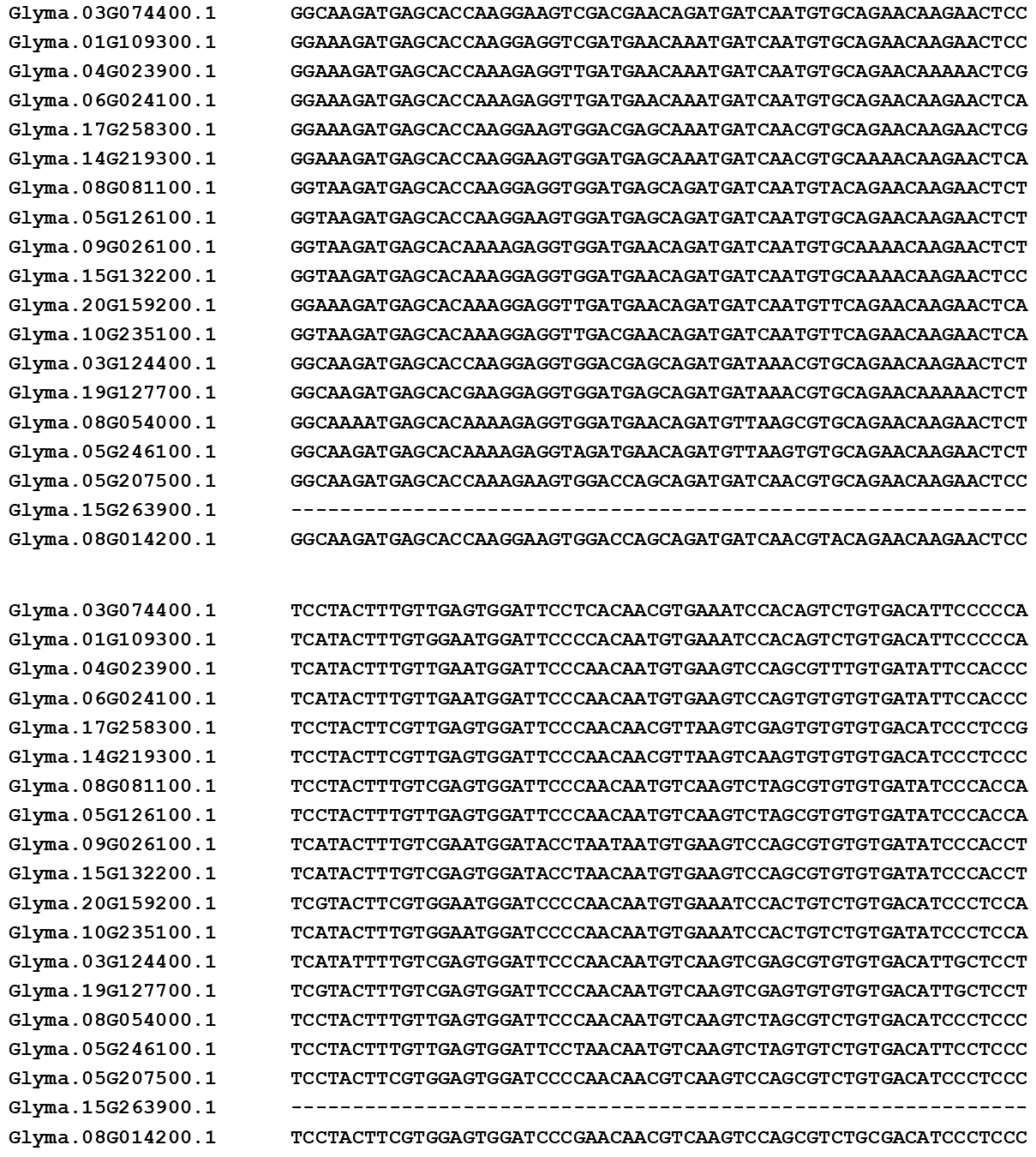
**

**Figure S31. (Cont.)**

**
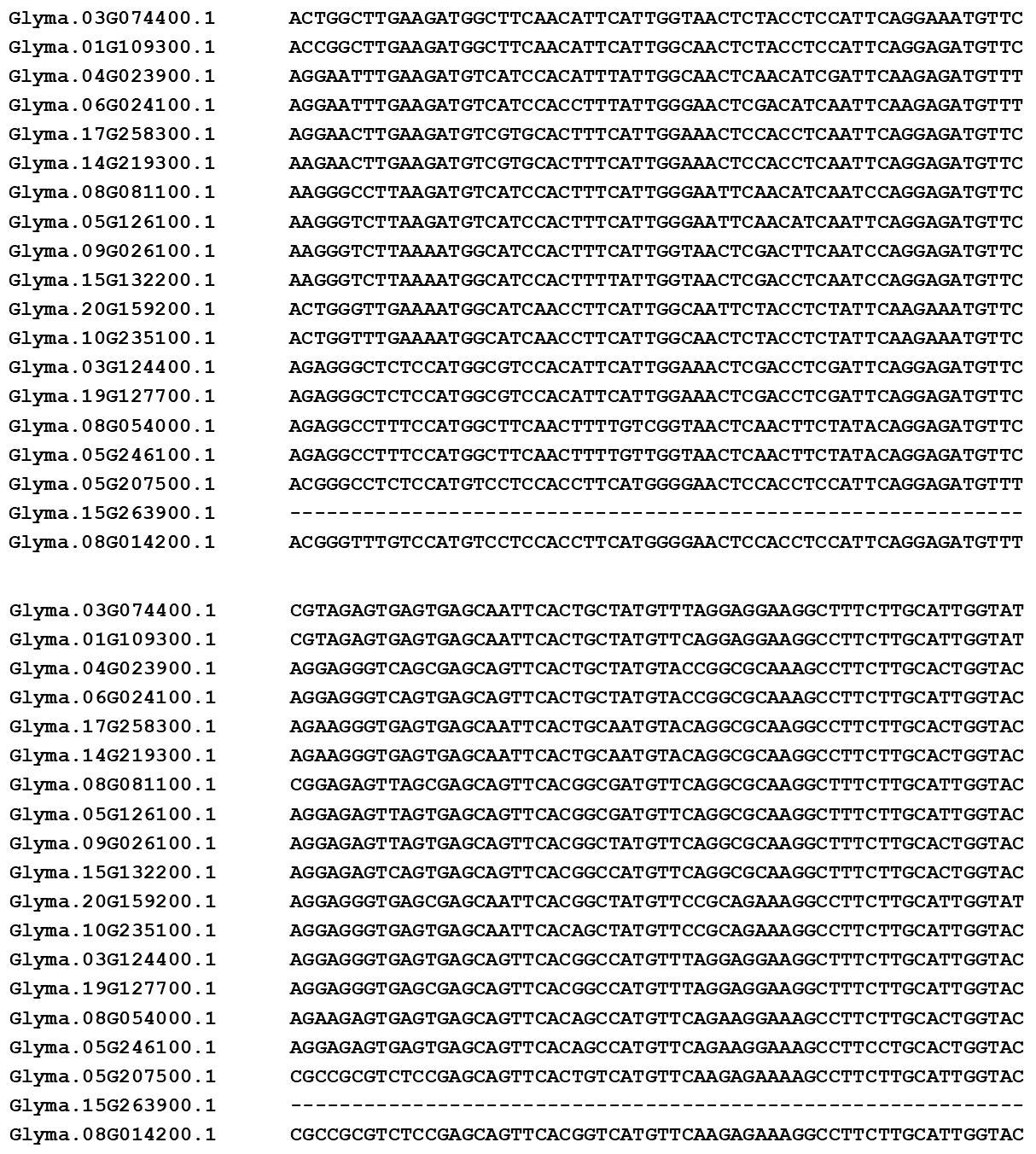
**

**Figure S31. (Cont.)**

**
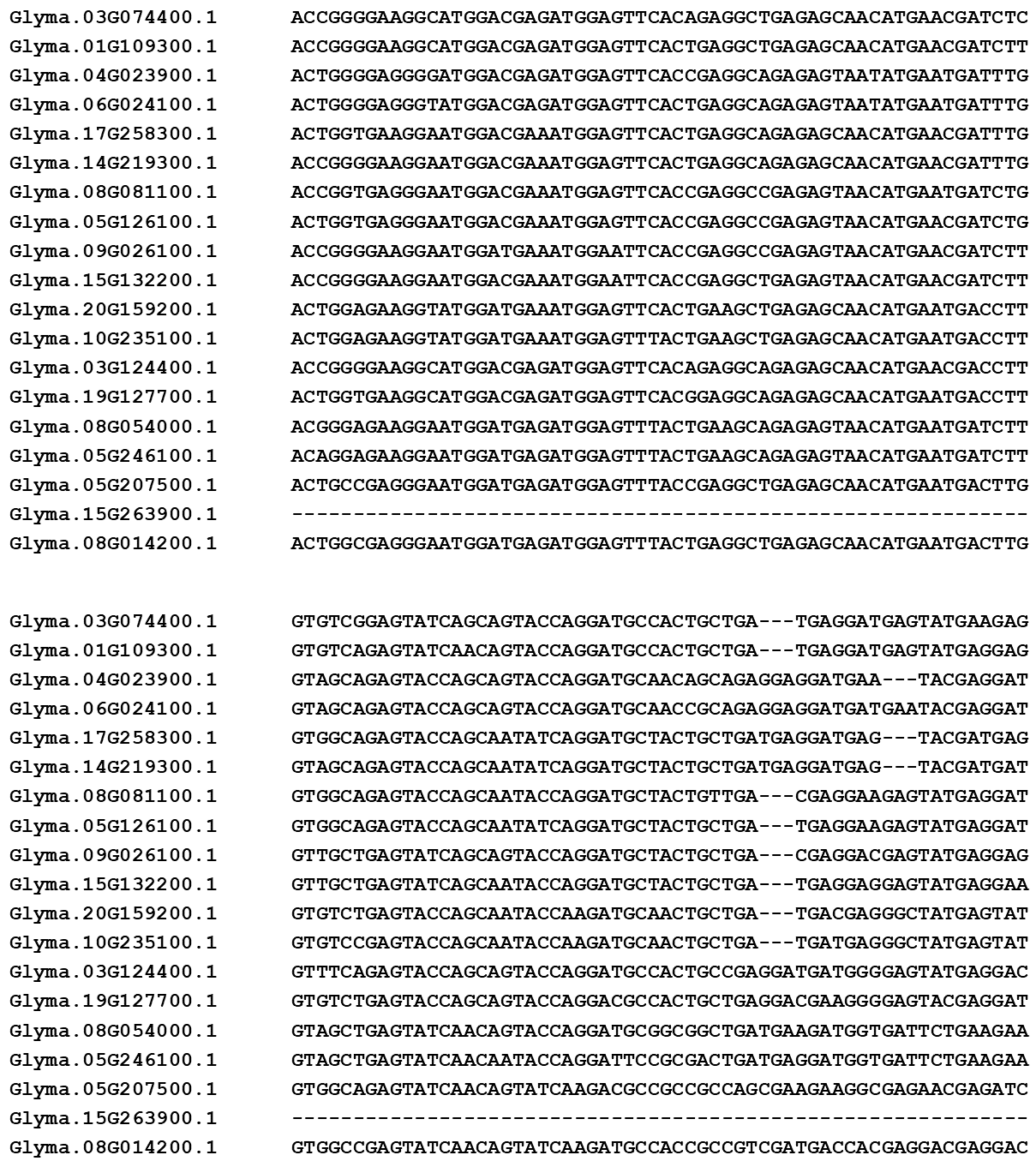
**

**Fig. S32. The plot of the averaged Ct values from three technical replicates against the Log (cDNA in ng/reaction) for optimization of qPCR conditions for the best primer pair of each of the six candidate reference genes in soybean.** The PCR efficiency (E; %) for each primer pair was calculated as E = (10^-1/A^ − 1) × 100. The cDNA concentration in 1:10, 1:20, 1:40, 1:80, and 1:160 dilutions was 5, 2.5, 1.25, 0.625, 0.3125 ng/µl, respectively, while the Log (cDNA in ng/reaction) for the 1:10, 1:20, 1:40, 1:80, and 1:160 dilutions were 0.69897, 0.39794, 0.09691, − 0.20412, and − 0.50515, respectively. The data from the lowest (or highest) one (or two) cDNA concentration might have been omitted in order to obtain R^2^ ≥ 0.99 and E = 100 ± 5% for the data from the remaining four (or three) consecutive cDNA concentrations for the best primer pair for each candidate gene. This served as the prerequisite for using the 2^−ΔΔCt^ method for data analysis.

**Fig. S33. Bacterial population recovered from soybean varieties Jack (top) and Williams 82 (W82, bottom) inoculated with a water control (Mock) or *Xanthomonas axonopodis* pv. *glycines* (Xag).** One-month-old soybean plants were spray inoculated with a bacterial suspension of Xag strain EB08 at a concentration of 1×10^8^ CFU/mL. At 0, 12, 24 and 120 hours post treatment (hpt) bacterial population sizes on extracted leaf discs were determined by serial dilution and plating. No bacteria were detected in the mock treated plants. Bacterial populations significantly changed within the first 12 hpt, determined by a one-way ANOVA (*F* = 453; *p* < .0001; *n* = 9) in Jack and (*F* = 247; *p* < .0001; *n* = 9) in W82. A post hoc Tukey test showed that there were no changes among bacterial population in both Jack and W82 across the different time points at *p* < .05.

**Fig. S34. The optimal number of reference genes for qPCR normalization in soybean detected by geNorm.** The pairwise variation (V_n/n+1_) of the six candidate reference genes under Xag treatment were calculated by the geNorm software, and the optimal number of reference genes was determined by the lowest number of genes with V_n_/V_n+1_ smaller than the threshold of 0.15.
